# Safety and Immunogenicity of an HIV-1 *Gag* DNA Vaccine with or without IL-12 and/or IL-15 Plasmid Cytokine Adjuvant in Healthy, HIV-1 Uninfected Adults

**DOI:** 10.1371/journal.pone.0029231

**Published:** 2012-01-05

**Authors:** Spyros A. Kalams, Scott Parker, Xia Jin, Marnie Elizaga, Barbara Metch, Maggie Wang, John Hural, Michael Lubeck, John Eldridge, Massimo Cardinali, William A. Blattner, Magda Sobieszczyk, Vinai Suriyanon, Artur Kalichman, David B. Weiner, Lindsey R. Baden, the NIAID HIV Vaccine Trials Network

**Affiliations:** 1 Division of Infectious Diseases, Department of Medicine, Department of Microbiology and Immunology, Vanderbilt University Medical Center, Nashville, Tennessee, United States of Ameica; 2 Division of Infectious Disease, Department of Medicine, University of Alabama Medical Center, Birmingham, Alabama, United States of America; 3 Department of Medicine, University of Rochester Medical Center, Rochester, New York, United States of America; 4 Vaccine and Infectious Disease Division, Fred Hutchinson Cancer Research Center, Seattle, Washington, United States of America; 5 National Institute of Allergy and Infectious Diseases, Bethesda, Maryland, United States of America; 6 Pfizer, Inc. Pearl River, New York, United States of America; 7 Profectus BioSciences, Inc. Tarrytown, New York, United States of America; 8 Institute of Human Virology, University of Maryland at Baltimore, Baltimore, Maryland, United States of America; 9 Columbia University School of Medicine, New York, New York, United States of America; 10 Chiang Mai Ram Hospital, Chiang Mai, Thailand; 11 Centro de Referencia e Treinamento em DST/AIDS, Sao Paulo, Brazil; 12 Department of Pathology, University of Pennsylvania, Philadelphia, Pennsylvania, United States of America; 13 Division of Infectious Disease, Brigham and Women's Hospital, Boston, Massachusetts, United States of America; INSERM, France

## Abstract

**Background:**

DNA vaccines are a promising approach to vaccination since they circumvent the problem of vector-induced immunity. DNA plasmid cytokine adjuvants have been shown to augment immune responses in small animals and in macaques.

**Methodology/Principal Findings:**

We performed two first in human HIV vaccine trials in the US, Brazil and Thailand of an RNA-optimized truncated HIV-1 *gag* gene (p37) DNA derived from strain HXB2 administered either alone or in combination with dose-escalation of IL-12 or IL-15 plasmid cytokine adjuvants. Vaccinations with both the HIV immunogen and cytokine adjuvant were generally well-tolerated and no significant vaccine-related adverse events were identified. A small number of subjects developed asymptomatic low titer antibodies to IL-12 or IL-15. Cellular immunogenicity following 3 and 4 vaccinations was poor, with response rates to *gag* of 4.9%/8.7% among vaccinees receiving *gag* DNA alone, 0%/11.5% among those receiving *gag* DNA+IL-15, and no responders among those receiving DNA+high dose (1500 ug) *IL-12* DNA. However, after three doses, 44.4% (4/9) of vaccinees receiving *gag* DNA and intermediate dose (500 ug) of *IL-12* DNA demonstrated a detectable cellular immune response.

**Conclusions/Significance:**

This combination of HIV *gag* DNA with plasmid cytokine adjuvants was well tolerated. There were minimal responses to HIV *gag* DNA alone, and no apparent augmentation with either IL-12 or IL-15 plasmid cytokine adjuvants. Despite the promise of DNA vaccines, newer formulations or methods of delivery will be required to increase their immunogenicity.

**Trial Registration:**

Clinicaltrials.gov NCT00115960 NCT00111605

## Introduction

DNA vaccines theoretically have potential to generate immune responses to common pathogens. The majority of HIV vaccines evaluated to date have relied on viral vectors such as vaccinia or adenovirus to deliver antigen [1]–[5]. DNA expresses the antigen of interest without the need for a vector delivery system; therefore this non-live vaccine approach circumvents the problem of vector-induced immune responses. In addition to the safety of this approach, DNA vaccines have the ability to induce cellular immune responses, which is in contrast to killed or subunit-based vaccines [6].

Several delivery methods for DNA vaccines have been tested in animals and in humans. The basic concept is the uptake of DNA into cells (skin, subcutaneous cells, muscle, and antigen presenting cells), where it reaches the nucleus via the host cellular machinery. Once there, gene transcription and protein production takes place. The cell provides the post-translational modifications to these proteins, that are then processed and presented in the context of HLA class I and class II molecules [1].

The major drawback of DNA vaccines has been a reduced level of immunogenicity in humans compared to animal models [7]–[10]. The first pre-clinical studies demonstrated that “naked” DNA could protect from virulent influenza [11]. While macaque and human studies have shown that DNA vaccines are not as immunogenic as vaccine vectors such as adenovirus [8], [12], combination prime-boost protocols with an HIV DNA priming regimen followed by adenovirus type 5-HIV DNA can increase the magnitude and qualitatively alter immune responses in macaques [13], [14] and humans [14]–[16]. A similar effect has been shown in a recent human trial of DNA priming followed by MVA boost [17]. However, some combination vaccines may be difficult to administer, thus methods to augment immunogenicity of DNA vaccines are desirable.

One approach to augment the immunogenicity of DNA is to combine the DNA plasmid of the gene of interest along with a plasmid cytokine adjuvant [18]. Two promising cytokine adjuvants include interleukin-12 [19] and interleukin-15 [20]. IL-12 has been shown to be a key cytokine for the induction of cellular immune responses. IL-12 is a p70 disulfide-linked heterodimer composed of two separately encoded subunits, a heavy chain of 40 kDa and a 35-kDa light chain that was originally cloned from B lymphoblastoid cell lines [21], [22]. Although p35 gene transcripts are rather ubiquitous, p40 transcripts are found exclusively in cells producing biologically active IL-12, which include monocytes, macrophages, dendritic cells (DCs), polymorphonuclear leukocytes (PMN), and B cells [23]. The importance of this cytokine is highlighted by the observation that genetic defects in IL-12 predispose to infections with intracellular pathogens such as salmonella and tuberculosis [24]–[26]. IL-15 is a glycoprotein of 14–15 kDA in size and is produced by monocytes, DCs and epithelial cells. It is a member of the common cytokine receptor γ-chain family, and was initially characterized as a T cell growth factor with similar in vitro properties as IL-2 [27]–[29]. IL-15 is important in the initial stimulation of the proliferation of activated T and B cells on antigenic stimulation, as well as the maintenance and activation of natural killer (NK) cells. Furthermore IL-15 inhibits IL-2 induced cell death and is an important cytokine for the development of long-lived memory T cell responses [30]–[32]. Here we summarize the first human trials combining vaccination with a plasmid expressing HIV-1 *gag* p37 and either an IL-12- or IL-15 expressing plasmid.

## Methods

### Study design

**Figure 1:**
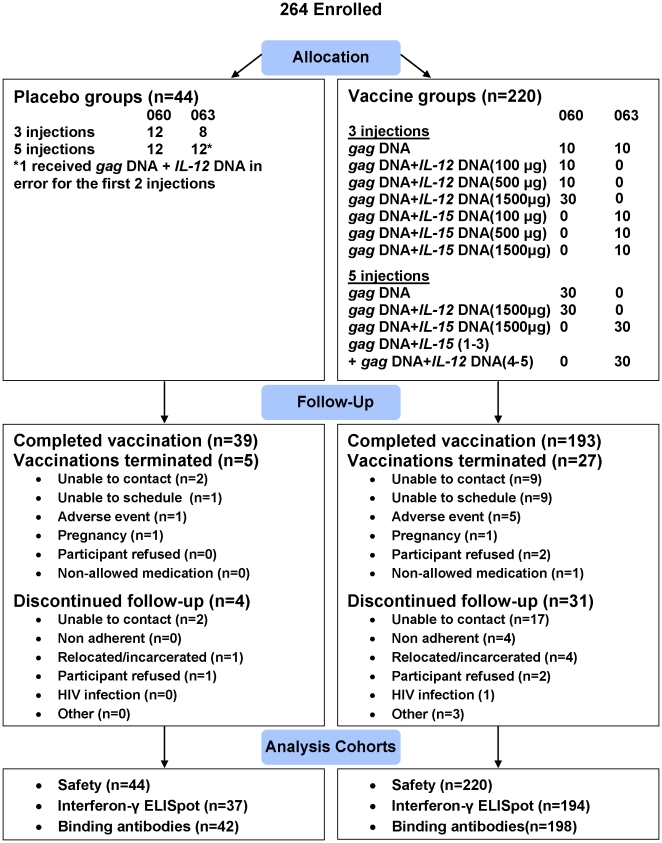
Allocation, follow-up, and analysis cohorts for HVTN 060 and HVTN 063.

**Figure 2:**
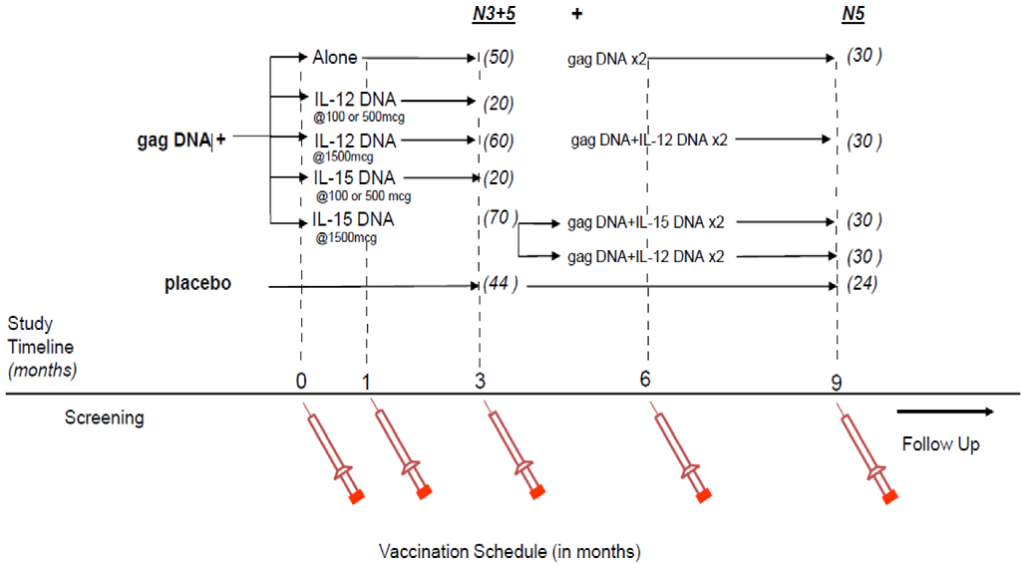
Study schema for HVTN 060/063. N3-5 – Number of participants randomized to either 3 dose or 5 dose regimen; N5 – Number of participants randomized to a 5 dose regimen.

The protocols for these trials and supporting CONSORT checklist are available as supporting information; see Checklist S1 and Protocols S1 and S2. HVTN 060 and 063 were both multicenter, randomized, placebo-controlled, double-blind Phase 1 trials conducted in the US, Brazil (063), and Thailand (060) (Figures 1 and 2).

### Participants

Study subjects were healthy HIV-1 uninfected adults (18 to 49 years old), who met eligibility criteria based on medical history, physical exam, and laboratory tests, including a CBC, serum creatinine, ALT, AST, CPK, alkaline phosphatase, and urinalysis. Volunteers were excluded for an allergy to amide-type local anesthetics. Participants provided signed informed consent in their native language. Reactogenicity and adverse events were graded based on the *HVTN Table for Grading Severity of Adverse Experiences*.

### Ethics

The studies were approved by the US Food and Drug Administration, the Recombinant DNA Advisory Committee (RAC), the National Health Surveillance Agency Brazil (ANVISA), the National Commission on Ethics in Research (CONEP) Brazil, the Thai Food and Drug Administration, the Committee for Research on Humans, Ministry of Public Health (Thailand), and study site institutional review boards and biosafety committees (Vanderbilt University Medical Center, Nashville; TN, University of Alabama Medical Center, Birmingham, AL; University of Rochester Medical Center, Rochester, NY; Fred Hutchinson Cancer Research Center, Seattle, WA, University of Maryland at Baltimore, Baltimore, MD; Columbia University School of Medicine, New York, NY; Chiang Mai Ram Hospital, Chiang Mai, Thailand; Centro de Referencia e Treinamento em DST/AIDS, Sao Paulo Brazil; University of Pennsylvania, Philadelphia, PA; and Brigham and Women's Hospital, Boston, Massachusetts).

### Interventions

Study participants were randomized to receive *gag* DNA alone (1500 ug), *gag* DNA + dose escalations of IL-12 plasmid (100, 500, or 1500 ug) (HVTN 060), or *gag* DNA + dose escalations of IL-15 plasmid (100, 500, or 1500 ug) (HVTN 063). In study 060, 30 individuals received an additional 2 vaccinations with *gag* DNA alone, and 30 individuals received 2 vaccinations with *gag* DNA+1500 ug of IL-12 plasmid. In study 063, 30 individuals received an additional 2 vaccinations with *gag* DNA+1500 ug of IL-15 plasmid, and 30 individuals received an additional 2 vaccinations with *gag* DNA+1500 ug of IL-12 plasmid. Vaccinations were given intramuscularly at months 0, 1, and 3, and months 6 and 9 where applicable (Figure 2).

### Study agents

The HIV-1 *gag* DNA vaccine is derived from strain HXB2. It was RNA optimized by introducing multiple silent point mutations within the coding region that disrupt endogenous inhibitory sequences that impede nuclear export thus allowing for high level Rev-independent expression [33]–[35]The plasmid backbone includes a eukaryotic gene expression unit that contains elements from the human cytomegalovirus (hCMV) immediate early promoter/enhancer and the bovine growth hormone (BGH) polyadenylation signal, a chimeric kanamycin resistance gene, and a pUC bacterial origin of replication. *In vitro* expression analyses this *HIV gag p37* DNA was performed in both African green monkey kidney (COS) and human rhabdomyosarcoma (RD) cells. Cells were transfected with 1 µg of the HIV *gag*-expressing DNA in the presence of a transfectant agent, Fugene (Roche); culture supernatants and cell lysates were examined for biological activity 48 hours later by assaying (ELISA) for HIV Gag. There was a significant enhancement in Gag expression (up to 200-fold) of the RNA-optimized *gag p37* over that observed with the original full-length HIV-1 *gag* DNA in a qualified assay [[[John, can you look at this section and modify? Is there a reference for the “qualified assay” part, or is this sufficient? SK]. The vaccine is formulated in 30 mM citrate buffer pH 6.5 containing 150 mM NaCl, 0.01% ethylenediamine tetraacetic acid (EDTA), and 0.25% bupivacaine- HCl.

The *IL-12* DNA adjuvant is a dual promoter expression plasmid which expresses the genes encoding human IL-12 proteins p35 and p40 under separate regulatory control. The p35 subunit is under the control of the hCMV promoter/enhancer and the SV40 (simian virus 40) polyadenylation signal. The p40 subunit is under the control of the SCMV (simian cytomegalovirus) promoter and the BGH (bovine growth hormone) polyadenylation signal. The plasmid contains a chimeric kanamycin resistance gene and a pUC bacterial origin of replication. The plasmid adjuvant is formulated in 30 mM citrate buffer pH 6.5 containing 150 mM NaCl, 0.01% ethylenediamine tetraacetic acid (EDTA), and 0.25% bupivacaine-HCl.

The *IL-15* DNA adjuvant encodes the human *IL-15* gene within a DNA plasmid expression vector. The *IL-15* gene is under the control of the hCMV promoter and the BGH polyadenylation signal. The plasmid also contains a chimeric kanamycin resistance gene and a ColE1 bacterial origin of replication. The *IL-15* gene has been optimized for high-level expression, with removal of the IL-15 signal peptide sequence in exchange for that of rhesus IL-15, and inclusion of Kozak sequences for efficient translation. The *IL-15* DNA is formulated in 30 mM citrate buffer pH 6.5 containing 150 mM NaCl, 0.01% EDTA and 0.25% bupivacaine-HCl.

Vaccine and plasmid cytokine aduvants were supplied by Wyeth, now Wyeth LLC and a wholly owned subsidiary of Pfizer Inc.

For both trials, the placebo was sodium chloride injection USP, 0.9%.

### Laboratory Assays

#### Evaluation for cytokine neutralizing antibodies

The presence of cytokine neutralizing antibody was detected by a reduction in the activity of a cytokine responsive cell line. HVTN 063 participants were tested for IL-15 antibodies either at baseline and 2 weeks following the 3^rd^ vaccination (3 dose groups) or 2 weeks following the 5^th^ vaccination (5 dose groups). For the IL-15 neutralization assay the CTLL-2 cell line is used, which proliferates in response to IL-15. To begin the assay, serum is mixed with an amount of IL-15 known to produce approximately 90% maximum proliferation. After 1 hour incubation of IL-15 and serum antibody, CTLL-2 cells are added to the mixture and cultured for 3 days after which time the proliferation of cells is measured. To measure proliferation, sodium 3,3′-[1(phenylamino)carbonyl]-3,4-tetrazolium]-3is(4-methoxy-6-nitro) benzene sulfonic acid hydrate is metabolized by live cells to form a colored product, which can be measured in ELISA plate readers. Thus, the presence of anti-IL-15 antibody would result in less colored product being formed. Each sample is serially diluted starting from neat serum and its concentration is calculated relative to a reference standard. The results of the assays are expressed in Neutralization units (NU) per ml. One NU is defined as the amount of antibody that neutralizes 1 International Unit (IU) of cytokine. The lower limit of quantitation is 11 NU/ml and a negative test is given a value of 6 NU

HVTN 060 participants were tested for IL-12 neutralization activity in serum at baseline and 2 weeks following the 1^st^, 2^nd^ and 3^rd^ vaccinations and 3.5 months after 3^rd^ vaccination (3 dose groups). In addition, those randomized to *gag* DNA + IL-15 (3 doses) + either IL-15 or IL-12 (2 doses) were tested 2 weeks following the 5^th^ vaccination. The IL-12 neutralization assay uses a natural killer cell line, NK92-MI, as the IL-12 responsive cell line. In this assay, the response of the NK92-MI cells to the presence of IL-12 is the secretion of IFN-γ, not cellular proliferation. The IFN-γ secreted in the culture supernatant is quantified in a sandwich ELISA using commercial reagents. The lower limit of quantitation for this assay was determined to be 8 NU/ml, and a negative test is given a value of 4 NU/ml. During clinical testing with this assay, high backgrounds in pre-vaccination serum were encountered such that results to approximately 30 NU/ml could be considered negative.

### Immunogenicity evaluations

#### Cellular assays

*Ex vivo* T cell responses were assessed using a validated IFN-γ ELISpot assay with a panel of two consensus Clade B *gag* peptide pools. The assays were performed using cryopreserved peripheral blood mononuclear cells. Specimens were excluded from analysis if: the participant was HIV-1 infected; the visit took place outside of the allowable visit window; cell viability was less than 66% upon thawing; the result was deemed unreliable by the lab, the mean of the medium-only wells was greater than 6 spot forming cells (SFC)/200,000 PBMC; the mean for the 3 positive control (PHA) wells was less than 400 SFC/200,000 PBMC; the mean for the negative control wells was greater than 20 SFC/200,000 PBMC; or results were available for fewer than 4 of the 6 negative control wells. Also, data for a specific peptide pool were excluded if: 1) there were results from fewer than 2 of the 3 experimental wells or 2) the ratio of the variance of the experimental wells to the median of the experimental wells +1 was greater than or equal to 25 SFC/200,000 PBMC.

#### Humoral assays

Serological tests for binding antibodies to p55 were assessed with a validated ELISA using single serum dilutions (1/100). Any of the time points that yielded positive results in the initial ELISA were subject to midpoint titration ELISA employing 6 serial dilutions of serum beginning at 1/100.

### Objectives

The primary objective of the study was to assess the safety, reactogenicity, and tolerability of *gag* DNA alone (1500 ug), *gag* DNA + dose escalations of IL-12 plasmid (100, 500, or 1500 ug) (HVTN 060), or *gag* DNA + dose escalations of IL-15 plasmid (100, 500, or 1500 ug) (HVTN 063) in healthy adults 18 to 49 years. Secondary objectives included the assessment of the immunogenicity of *gag* DNA alone or in combination with plasmid cytokine adjuvants.

### Outcomes

Initial safety assessment included visual inspection of the injection 30 minutes post injection, and subsequent safety assessments were performed at up to 11 visits depending on receipt of 3 vs 5 vaccinations (on days 14, 28, 42, 84, 98, 168, 182, 273, 287, 364, and 455). Reactogenicity and adverse events were graded based on the *HVTN Table for Grading Severity of Adverse Experiences*. *Ex vivo* T cell responses were assessed with a validated IFN-γ ELISpot assay using cryopreserved peripheral blood mononuclear cells (PBMC) from HVTN 060 samples obtained at baseline, 2 weeks post 3^rd^ vaccination, and 2 weeks post 4^th^ and 5^th^ vaccinations (5 dose groups only), and from 063 samples at baseline and 2 weeks post 3^rd^ vaccination (3 dose groups only), and 2 weeks post 4^th^ vaccination (5 dose groups only). Humoral assays from HVTN 060 and HVTN 063 samples were performed at baseline. Subsequent samples from HVTN 060 were performed 2 weeks post 3^rd^ vaccination (3 dose groups) and 2 weeks post 5^th^ vaccination (5 dose groups) and from 063 samples at 2 weeks post 3^rd^ vaccination (3 dose groups) and 2 weeks post 4^th^ vaccination (5 dose groups).

### Sample size and randomization

Groups in the *IL-12* (060) and *IL-15* (063) dose escalation phases, which received 3 vaccinations, were each randomized with a ratio of 10 vaccine to 2 placebo. Groups receiving products at the maximum dose with 5 vaccinations were randomized with a ratio of 30 vaccine to 6 placebo. Placebos were included primarily to maintain blinding, provide some safety reference data, and to provide controls for immunogenicity assays. Sample size was selected based on the ability to detect rare safety events since these were first in human trials. Within vaccine groups of size 30 (10), there was a 90% chance of observing at least 1 adverse event if the true rate of an event was at least 8% (21%).

Randomization was conducted independently for the two trials and stratified by country. Randomization assignments were computer generated by a centralized statistical and data management center and provided to site pharmacists. Participants, site staff other than the site pharmacists, laboratory personnel responsible for endpoint assays, and investigators were blinded as to treatment assignments during the conduct of the trial.

### Statistical methods

**Figure 3:**
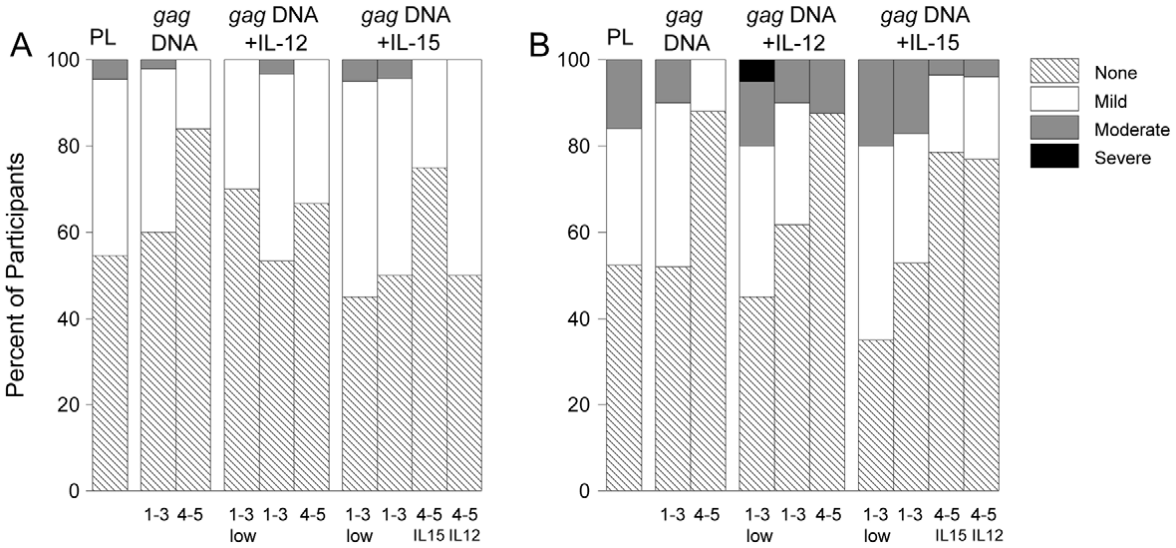
Severity of vaccine reactions. Panel A shows the maximum severity of pain or tenderness at the injection site and Panel B the maximum severity of systemic reactogenicity symptoms. Systemic symptoms include malaise and/or fatigue, myalgia, headache, nausea, vomiting, chills and arthralgia. Data for vaccinations 1–3 and for vaccinations 4–5 are combined. Data for adjuvants at a dose of 100 or 500 ug are also combined (labeled as low). Abbreviation PL = placebo.

All participants received at least one vaccination and are therefore included in the safety analyses. For vaccine reactions, the maximum severity of pain and/or tenderness and of systemic symptoms was calculated for study injections 1–3 and 4–5 separately. Systemic symptoms included malaise and/or fatigue, myalgia, headache, nausea, vomiting, chills and arthralgia. No statistically significant differences were observed for vaccine reaction symptoms between groups receiving 100 or 500 ug doses of either IL-12 or IL-15 adjuvant, so the two dose groups were combined for each adjuvant in Figure 3. Differences in the distribution of severity of vaccine reactions were assessed with exact Wilcoxon rank sum tests.

For the IFN-γ ELISpot assay, to determine a positive response to a specific peptide pool, the distribution free method of Moodie, et al [36] was used. This approach uses a bootstrap test to test the null hypothesis that the mean of the experimental wells was equal to twice the mean of the negative control wells, versus the alternative hypothesis that the experimental mean was greater than twice that of the negative control mean based on log_10_ transformed data. The method adjusted for the two peptide pools by calculating step-down maxT adjusted p-values. Peptide pools with adjusted one-sided p-values≤0.05 were declared positive. In addition to a significant p-value, the mean background-subtracted response for the peptide pool had to be >50 SFC/10^6^ PBMC for the peptide pool to be considered positive. The purpose of this criterion was to require a minimal demonstration of biological activity. If either of the peptide pools was positive, then the overall response was considered positive.

For the ELISA assay, response to an antigen was considered positive if the differences in optical densities between duplicate antigen-containing and non-antigen containing wells was >0.2. For the IL-12 and IL-15 neutralization assays, values greater than 30 and 11 NU/ml, respectively, are considered positive.

Immunogenicity analyses were based on intent-to-treat. Response rates were calculated based on evaluable data as listed under immunogenicity evaluations above. Two-sided 95% confidence intervals were calculated using the score test method [37]. Differences in response rates between groups were tested with Fisher exact tests. Statistical tests were two-sided and considered significant if P<0.05.

## Results

### Trial population

Between August 2005 and May 2007, the HIV Vaccine Trials Network enrolled 264 healthy HIV-1 uninfected adults from the US, Brazil and Thailand into two randomized, placebo controlled, double blinded HIV vaccine trials (HVTN 060 and HVTN 063) (220 to a vaccine regimen and 44 to placebo). As these 2 trials are quite similar in design and utilized the same vaccine products, we are presenting the combined data. In total, 20 participants were randomized to 3 doses and 30 to 5 doses of *gag* DNA alone; 10 each to 3 doses of *gag* DNA and *IL-12* or *IL-15* DNA at an adjuvant dose of either 100 or 500 ug; 30 to 3 doses and 30 to 5 doses of *gag* DNA+*IL-12* DNA at the maximum dose of 1500 ug ; 10 to 3 doses and 30 to 5 doses of *gag* DNA+*IL-15* DNA at the 1500 ug, 30 to 3 doses of *gag* DNA+*IL-15* DNA at the 1500 ug dose followed by 2 doses with *gag* DNA+*IL-12* DNA (1500 ug); and 20 to 3 doses and 24 to 5 doses of placebo (Figure 2). All vaccinations were administered intramuscularly in the deltoid.

More than half of participants were male (54%), with a median age of 31 (Table 1). Participants represented a mixture of racial/ethnic groups, with 54% being white non-Hispanic. Overall, 91% received 3 study injections and 85% of those randomized to 5 study injection completed their regimens (Table 2). The most common reasons for not completing vaccination were: the participant could not be contacted (n = 11; 4.2%), and the participant was unable to schedule the vaccination visit within the allowable visit window (n = 10; 3.8%). Site investigators discontinued vaccinations for 4 vaccine recipients due to adverse events deemed probably not related to vaccination (manic episode, suicide intention, decreased hemoglobin related to heavy menses, and new onset mild type II diabetes). Two participants refused to continue with vaccinations due to clinical events (one placebo participant with moderate abdominal pain deemed probably not related to vaccination and one vaccinee with mild elbow pain deemed possibly related).

**Table 1:** Participant characteristics.

		*N (%)*
Total enrolled		264
Country	US	240 (90.9%)
	Brazil	12 (4.5%)
	Thailand	12 (4.5%)
Sex	Male	142 (53.8%)
	Female	122 (46.2)
Race	Race	
	White - non-Hispanic	142 (53.8%)
	African American - non-Hispanic	63 (23.9%)
	Hispanic	32 (12.1%)
	Other	27 (10.2%)
Age	Median	31 years
	minimum, maximum	18, 50 years

**Table 2:** Number of vaccinations received.

	Vaccination Number				
	1st	2nd	3rd	4th	5th
Gag DNA	50/50 (100.0%)	46/50 (92.0%)	44/50 (88.0%)	25/30 (83.3%)	24/30 (80.0%)
Gag DNA+IL-12(100/500 mcg)	20/20 (100.0%)	20/20 (100.0%)	19/20 (95.0%)	NA	NA
Gag DNA+IL-12(1500 mcg)	60/60 (100.0%)	57/60 (95.0%)	53/60 (88.3%)	24/30 (80.0%)	24/30 (80.0%)
Gag DNA+IL-15(100/500 mcg)	20/20 (100.0%)	20/20 (100.0%)	20/20 (100.0%)	NA	NA
Gag DNA+IL-15(1500 mcg)	70/70 (100.0%)	69/70 (98.6%)	66/70 (94.3%)	28/30 (93.3%)	26/30 (86.7%)
+Gag DNA+IL-12(1500 mcg) boost at 4^th^ & 5^th^ vaccinations				26/30 (86.7%)	25/30 (83.3%)
Placebo	44/44 (100.0%)	41/44 (93.2%)	39/44 (88.6%)	23/24 (95.8%)	23/24 (95.8%)
Total	264/264 (100.0%)	253/264 (95.8%)	241/264 (91.3%)	126/144 (87.5%)	122/144 (84.7%)

Abbreviations: NA – not applicable.

For vaccinations 1, 2 and 3, the denominator is the number of persons randomized to receive either 3 or 5 study injections.

For vaccinations 4 and 5, the denominator is the number randomized to 5 study injections.

The numerator is the number of people who actually received the specified vaccination.

### Safety

The vaccines were well tolerated. Fifteen participants on vaccine arms and 5 receiving placebo had erythema and/or induration of 6 cm^2^ or less at the injection site. Overall, 45% of participants had mild and 3% moderate pain and/or tenderness at the injection site in the three days following vaccination. There were no statistically significant differences in severity between placebo and vaccine groups and no increase in severity with additional doses (Figure 3A). Half of participants had no systemic vaccine reactions, 34% had a maximum severity of mild, and 15% had a maximum severity of moderate (Figure 3B). One *gag* DNA+*IL-15* DNA participant had a severe headache on day 3 following the 2^nd^ vaccination, which was thought to be related to sinusitis, not vaccination. This participant received the 3^rd^ vaccination with no reactions reported. One participant randomized to placebo inadvertently received *gag* DNA+*IL-12* DNA at the first two study injections and placebos at the later 3 injections due to pharmacy errors. This participant had mild tenderness at the injection site following the 3^rd^ injection (1st placebo injection) and mild elevated SGOT deemed probably not related to study product 2 weeks after the 3^rd^ injection. This participant reported no other vaccine reactions and other adverse events were mild and not related to product (occupational thumb pain and a cold).

### Adverse Events

Thirty-nine (88.6%) of placebo subjects and 182 (82.7%) of vaccine subjects reported at least one AE. There were no Grade 3 or 4 AEs or SAEs related to study products. The vaccine and cytokine adjuvants did not have any apparent effects on hematologic parameters (complete blood count, CBC), CD4+ lymphocyte counts, or serum chemistries compared to placebo. AEs that were reported as “definitely related” or “probably related” to vaccination were mild in severity: 1 event each of injection site pruritis, injection site swelling, injection site pain, injection site papule, pyrexia, and injection site hematoma. AEs that were considered “possibly related” among vaccine recipients were mild except as noted: CPK increased (3 events, mild to moderate), hemoglobin decreased (3), AST increased (2), CD4 decreased (2) , neutropenia (moderate), myalgia (2), URI (2), dizziness (2), and 1 event each for chills (moderate), fatigue (moderate), HA (moderate), lymphocytes decreased (moderate), injection site papule, ALT increased, arthralgia, atrial fibrillation, back pain, hyperreflexia, nasal congestion, pharyngitis, proteinuria, rhinorrhea, sneezing, anorexia, apthous stomatitis, chest discomfort, abnormal UA, nausea, presyncope, metrorrhagia, and musculoskeletal pain.

Among those who received *gag* DNA+IL-12, two participants out of 68 tested for IL-12 antibody following the 1^st^ vaccination had positive results. One had a low level result of 32.3 NU/ml (a positive response was >30 NU/ml) that was not present at baseline, or following the 2^nd^ or 3^rd^ vaccination. The other had a result of 45.1 NU/ml but had also tested positive at baseline with a value of 71.4 NU/ml. None tested positive following the 2^nd^ vaccination (n = 61 tested) or following the 3^rd^ vaccination (n = 69 tested) and none who received 3 doses of *gag* DNA+IL-15 followed by *gag* DNA+IL-12 (n = 26 tested) tested positive after the 5^th^ vaccination. With regard to IL-15 antibody, two participants (n = 26 tested) who received *gag* DNA+IL-15 followed by *gag* DNA+IL-12 tested positive after the 5th vaccination (values of 11.5 and 17.7 NU/ml, a positive response was >11 NU/ml). On the *gag* DNA+IL-15 arms, none among 29 tested who received 3 doses tested positive after the 3^rd^ vaccination and none among 28 who received 5 doses tested positive after the 5^th^ vaccination. The presence of IL-12 antibody or IL-15 antibody was not associated with any adverse events.

### Immunogenicity

T-cell responses as measured by the Interferon-gamma (IFN-γ) ELISpot assay were minimal and of apparent short duration as none of those assayed post 5^th^ vaccination had a response (Table 3). No statistically significant differences were observed in pairwise comparisons of response rates for *gag* DNA alone to the groups receiving either IL-12 or IL-15 plasmid adjuvants following either the 3^rd^ or 4^th^ vaccinations. One participant who received *gag* DNA alone responded at both time points (background adjusted SFCs of 165 and 252/10^6^ PBMC, respectively). For those receiving 5 *gag* DNA+*IL-15* DNA vaccinations or those receiving 3 *gag* DNA+*IL-15* DNA followed by 2 *gag* DNA+IL-12 DNA vaccinations, assays were not performed following the 3^rd^ vaccination so comparisons to the post 5^th^ vaccination time point are not possible. Following three vaccinations, response rates were: 4.9% (2/41 subjects; 95% CI 1.3%, 16.1%) for *gag* DNA alone and 21.1% (4/19 subjects; 95% CI 8.5%, 43.3%) for *gag* DNA+*IL-12* DNA at either a 100 or 500 ug dose (There were 0/10 responders in the gag DNA+*IL-12 DNA* (100 ug) group, and 4/9 responders in the *gag* DNA+IL-12 (500 ug) group). No responses were observed for the *gag* DNA+1500 ug IL-12 group or for any of the *gag* DNA+IL-15 dose groups. Following four vaccinations, the *gag* DNA alone response rate was 8.7% (2/23 subjects; 95% CI 2.4%, 26.8%); for *gag* DNA+1500 ug IL-15, 11.5% (3/26 subjects; 4.0%, 29.0%) and in subjects receiving 3 *gag* DNA+IL-15 vaccinations followed by vaccination with *gag* DNA+IL-12, 3.6% (1/28 subjects; 0.6%, 17.7%). One placebo participant responded at baseline and post 3^rd^ vaccination (background adjusted SFCs/10^6^ PBMC 221 and 100, respectively). No responses were observed at baseline among the 132 vaccine recipients with assay results.

**Table 3:** IFN-γ ELISpot response.

	Post 3^rd^ vaccination		Post 4^th^ vaccination		Post 5^th^ vaccination
	Response rate (95% CI)	Background adjusted SFC/10^6^ PBMC for responders	Response rate (95% CI)	Background adjusted SFC/10^6^ PBMC for responders	Response rate (95% CI)
Gag DNA	2/41 = 4.9% (1.3%, 16.1%)	165, 178.5	2/23 = 8.7% (2.4%, 26.8%)	76.5, 252	0/21 = 0.0% (0.0%, 15.5%)
Gag DNA+IL-12 (100 mcg)	0/10 = 0% (0%, 27.8%)		NA		NA
Gag DNA+IL-12 (500 mcg)	4/9 = 44.4% (18.9%, 73.3%)	55, 57.5, 61.5, 103.5	NA		NA
Gag DNA+IL-12 (1500 mcg)	0/49 = 0.0% (0.0%, 7.3%)		0/24 = 0.0% (0.0%, 13.8%)		0/23 = 0.0% (0.0%, 14.3%)
Gag DNA+IL-15 (100/500 mcg)	0/18 = 0.0% (0.0%, 17.6%)		NA		NA
Gag DNA+IL-15 (1500 mcg)	0/6 = 0.0% (0.0%, 39.0%)		3/26 = 11.5% (4.0%, 29.0%)	61, 147.5, 195	ND
+Gag DNA+IL-12 (1500 mcg) at 4^th^ & 5^th^ vaccinations			1/28 = 3.6% (0.6%, 17.7%)	71.5	ND
Placebo	1/23 = 4.3% (0.8%, 21.0%)	100	0/24 = 0.0% (0.0%, 13.8%)		0/11 = 0.0% (0.0%, 25.9%)

Abbreviations: CI-confidence interval; SFC-spot forming cells; NA-not applicable for those randomized to 3 vaccinations; ND-assay not performed.

We also measured humoral immune responses to *gag*. One participant in the *gag* DNA+IL-15 followed by *gag* DNA+IL-12 arm had a low level ELISA response to p55 at baseline and post 4^th^ vaccination (background adjusted ODs 0.21 and 0.22). No participant developed a vaccine-induced anti-*gag* response.

## Discussion

These trials demonstrate the safety of an HIV-1 DNA vaccine administered in combination with plasmid cytokine adjuvants. Cytokines administered in protein form can cause severe side effects. IL-15 shares the common gamma chain with IL-2, and although IL-15 has never been given directly, IL-2 can cause side effects such as fever, malaise and hypotension [38]. The use of IL-12 protein administered intravenously at doses of 500–1000 ng/kg has been associated with stomatitis, elevation of liver enzymes, leukopenia, and death [39]–[41]. However, In pre-clinical toxicity studies, the use of plasmid cytokine adjuvants did not result in detectable elevations in cytokine levels systemically, and we saw no severe reactions related to vaccination in these trials, suggesting that any cytokine production was limited to the site of injection.

The low level of cellular immunogenicity in our study contrasts with previous findings in macaques. Shadeck et. al. immunized groups of 5 macaques with either low dose (1.5 mg) or high dose (5 mg) SIV *gag p39*, which was codon optimized in a manner similar to the *HIV gag p37* construct used in this human trial, with or without 1.5 mg or 5.0 mg of *IL-12* plasmid DNA. The administration of plasmid IL-12 at low dose in combination with low dose HIV DNA led to cellular immune responses in all animals, with a mean value 5-fold higher than macaques immunized with high dose SIV *gag* DNA alone. The increased magnitude of immune responses was associated with increased breadth of responses as measured by the number of individual overlapping peptides recognized by vaccinated animals. The ability of low dose pIL-12 in combination with SIV DNA to elicit responses superior to DNA alone results in a significant dose-sparing effect that allows lower doses of DNA to be used, or allows the incorporation of DNAs expressing additional HIV/SIV gene products [42]. In macaques, IL-15 administered as plasmid cytokine adjuvant with SIV *gag* DNA marginally increased the magnitude of antigen-specific interferon-gamma producing T cells compared with SIV *gag* DNA alone [43], [44]. It was not as potent as SIV DNA administered with pIL-12, and there was no significant difference in the magnitude of responses when pIL-15 was co-administered with pIL-12 [43]. However, cells from macaques primed with SIV *gag* DNA+pIL-15 show enhanced ability to proliferate in vitro compared to SIV *gag* DNA alone, suggesting that pIL-15 delivered as an adjuvant may qualitatively affect the vaccine-induced immune response [44].

Despite promising results in macaque studies of a similar SIV *gag* DNA at similar doses, we found limited immunogenicity after administration of the combination of HIV *gag* DNA with either IL-12 or IL-15 or both. For many vaccines, not limited to DNA, there is a marked difference in the magnitude of immune responses between macaques and humans [14], so immune responses elicited by vaccination may be quite different for unknown species-specific reasons. Other trials have noted a lack of *gag* responses after DNA vaccination with different *gag* DNA constructs from the one we tested in this trial, and this may reflect something inherently different about the human response to *gag* HIV DNA [8], [16]. We also may have been limited by the use of a truncated form of *gag*, which did not include several dominant HIV epitopes present in areas outside of p37 (http://www.hiv.lanl.gov/content/immunology/tables/ctl_summary.html) [45] and not incorporating other insert antigens which may be more immunogenic, such as envelope [8]. It is unikely that the lack of immunogenicity was due to a lack of expression of the *gag* HIV DNA. *In vitro* expression analyses of the HIV gag p37 DNA was performed in both African green monkey kidney (COS) and human rhabdomyosarcoma (RD) cells. There was significant enhancement in Gag expression (up to 200-fold) with the RNA-optimized *gag p37* over that observed with the original full-length HIV-1 *gag* DNA, and this was the reason for going forward with this particular study product. In macaque trials without plasmid cytokine adjuvant, higher doses of DNA improved the magnitude of immune responses, and higher concentrations of HIV DNA may improve immunogenicity in future trials [43].

Despite the theoretical advantages of DNA vaccination, and the apparent immunogenicity of these vaccines in animals, significant improvements will be necessary if these vaccines are to enter larger clinical trials as a standalone regimen. There have been some responses to HIV DNA, but mainly limited to Envelope responses [8], [16]. Recent studies have shown that while DNA alone is a very poor immunogen in humans, as part of a prime-boost strategy with Adenovirus or MVA, it can significantly increase the magnitude and quality of the immune response [16], [17]. The cytokine plasmid approach has been quite successful in macaques, and here too, more concentrated versions of these plasmid cytokine adjuvants or more careful timing of cytokine administration may be required to induce robust responses in humans. Other adjuvants currently in testing include TLR agonists, or other cytokine plasmids such as IL-28 as recently published by Morrow et. al. [19].

Another approach toward DNA vaccination may rely on the delivery method of DNA. Recent studies have shown electroporation to be highly efficient at inducing immune responses in animal models, with up to 40-fold higher frequencies of cytokine-producing T cells [46], [47]. In a recent macaque study comparing 4 doses of SIV DNA vaccination delivered via in vivo electroporation to 3 doses of Ad5-SIV vaccination, peak immune responses to SIV *gag* were 2.5 fold higher, and peak responses to SIV *pol* 5.5 fold higher in the DNA-EP vaccinated group [48]. In addition to the higher magnitudes of responses as measured by IFN-γ ELISpot, SIV-DNA delivered via EP induced immune responses were more “polyfunctional” than those induced by Ad5-SIV as measured by antigen-specific secretion of TNF-α, Mip-1α, and IL-2, along with enhanced antigen-specific proliferation [48]. A recent small trial in humans showed HIV DNA delivered via IM electroporation to be safe and more immunogenic than DNA delivered via standard IM vaccination [29].

These data suggest that IL-12 and IL-15 are safe to give, but offered little ability to augment cellular immune responses in this format. The results of human vaccine trials currently in progress with DNA delivered via electroporation, with and without plasmid cytokine adjuvant, will help us determine whether DNA vaccination can induce cellular immune responses either alone or as part of a prime-boost regimen.

## Supporting Information

Checklist S1 **CONSORT checklist.**
(DOC)

Protocol S1 **Trial Protocol HVTN 060.**
(PDF)

Protocol S2 **Trial Protocol HVTN 063.**
(PDF)
