# Supplementary material for: Safety and Immunogenicity of an HIV-1 Gag DNA Vaccine with or without IL-12 and/or IL-15 Plasmid Cytokine Adjuvant in Healthy, HIV-1 Uninfected Adults
Source: PLoS One. 2012 Jan 5;7(1):e29231. doi: 10.1371/journal.pone.0029231 (PMC3252307; doi:10.1371/journal.pone.0029231)
Supplement: Protocol S1 — Trial Protocol HVTN 060. (PDF) [file pone.0029231.s002.pdf]

HIV VACCINE
TRIALS NETWORK

PROTOCOL
HVTN 060

A Phase I clinical trial to evaluate the safety and immunogenicity of an HIV-1 *gag* DNA vaccine with or without *IL-12* DNA adjuvant, boosted with homologous plasmids or with HIV CTL multiepitope peptide vaccine / RC529-SE plus GM-CSF, in healthy, HIV-1 uninfected adult participants

CLINICAL TRIAL SPONSORED BY

Division of AIDS (DAIDS)
National Institute of Allergy and Infectious Diseases (NIAID)
National Institutes of Health (NIH)
Department of Health and Human Services (DHHS)
Bethesda, Maryland, USA

BB IND [#] HELD BY DAIDS

VACCINE PROVIDED BY

Wyeth Vaccines Research
Pearl River, New York, USA

March 22, 2005

HVTN 060, Version 1, FINAL

This page is intentionally blank

Brief contents

Detailed contents 4
Tables and figures 8
Schema 9
Summary 10
Introduction 13
Ethical considerations 14

Study products

1 Products background 16
2 Study product descriptions 22
3 Preclinical studies 27
4 Clinical studies 39
5 Summary 41

Study design

6 Study objectives 44
7 Study type, study population, and eligibility criteria 45
8 Safety and immunogenicity evaluations 48
9 Statistical considerations 53

Study operations

10 Protocol conduct 62
11 Informed consent 63
12 Procedures 65
13 Study product preparation and administration 67
14 Safety monitoring and review 76
References 84
Protocol history 91
Protocol team 92

Appendices

Appendix A: Sample informed consent form 94
Appendix B: Laboratory procedures for Part A 115
Appendix C: Laboratory procedures for Part B 116
Appendix D: Procedures at HVTU 117

Detailed contents

Brief contents ... 3
Detailed contents ... 4
Tables and figures ... 8
Schema ... 9
Summary ... 10
Introduction ... 13
Ethical considerations ... 14

Study products

1 Products background ... 16
1.1 Plasmid DNA vaccination ... 17
1.2 DNA-based HIV vaccines in humans ... 18
1.3 Cytokine-augmented DNA vaccination ... 18
1.4 IL-12 background ... 19
1.5 Recombinant human IL-12 (rhIL-12) protein ... 20
1.5.1 Clinical studies of rhIL-12 protein in therapeutic settings ... 20
1.5.2 Clinical studies of rhIL-12 protein as vaccine adjuvant ... 20
1.5.3 *IL-12* DNA compared to rhIL-12 protein ... 21
1.6 *IL-12* DNA plasmid as a vaccine adjuvant ... 21
2 Study product descriptions ... 22
2.1 HIV-1 *gag* DNA vaccine ... 22
2.2 *IL-12* DNA adjuvant ... 22
2.3 HIV CTL MEP vaccine ... 23
2.3.1 HIV CTL MEP ... 23
2.3.2 RC529-SE adjuvant ... 24
2.4 GM-CSF as a vaccine adjuvant ... 25
2.4.1 Antibodies to GM-CSF ... 25
2.5 Placebo ... 26
3 Preclinical studies ... 27
3.1 Preclinical safety studies ... 27
3.1.1 Preclinical safety studies of *gag* DNA ... 27
3.1.2 Preclinical safety studies of *gag* DNA and *IL-12* DNA ... 27
3.1.2.1 Toxicology study of murine *IL-12* DNA alone or in combination with HIV-1 *gag* DNA in mice ... 29
3.1.2.2 Toxicology study of *IL-12* DNA alone or in combination with HIV-1 *gag* DNA in New Zealand white rabbits ... 30
3.1.2.3 Biodistribution analysis of *IL-12* DNA and *gag* DNA in rabbits ... 30
3.1.2.4 Integration analysis of *IL-12* DNA and *gag* DNA in rabbits ... 30
3.1.2.5 Safety of rhesus *IL-12* DNA plasmid in rhesus macaques ... 31
3.1.3 Preclinical safety studies of CTL MEP vaccine ... 31
3.1.3.1 Toxicology study of HIV CTL MEP vaccine in New Zealand white rabbits ... 31
3.1.3.2 HIV CTP MEP and SIV CTL MEP vaccine in nonhuman primates ... 31
3.2 Preclinical immunogenicity studies ... 31
3.2.1 Preclinical immunogenicity studies of SIV *gag* DNA alone and with rhesus *IL-12* plasmid DNA in rhesus macaques ... 31

3.2.2 Preclinical immunogenicity studies of HIV CTL MEP vaccine ... 33
3.2.2.1 Immunogenicity of HIV CTL MEP in HLA-A*0201 transgenic mice ... 33
3.2.2.2 Nonhuman primate studies with related peptide vaccines ... 33
3.2.2.3 Study of HIV CTL MEP and HIV DNA vaccines in cynomolgus macaques ... 35

4 Clinical studies ... 39
4.1 HIV-1 *gag* DNA vaccine ... 39
4.2 *IL-12* DNA adjuvant ... 39
4.3 HIV CTL MEP / RC529-SE vaccine ... 39
4.4 Clinical study of an HIV peptide vaccine: AVEG 020 ... 40

5 Summary ... 41
5.1 Rationale for trial design ... 41
5.2 HVTN 056 and timing of initiation of HVTN 060 ... 41
5.3 Plans for future product development and testing ... 42

Study design

6 Study objectives ... 44
6.1 Primary objectives ... 44
6.2 Secondary objectives ... 44

7 Study type, study population, and eligibility criteria ... 45

8 Safety and immunogenicity evaluations ... 48
8.1 Considerations for trial start ... 48
8.2 Initial safety evaluation ... 48
8.3 Safety considerations for dose escalation ... 48
8.4 Safety evaluation for moving from Part A to Part B ... 48
8.5 Other safety considerations ... 49
8.5.1 Exclusion of participants with egg allergy (Part B only) ... 49
8.5.2 Exclusion of participants with yeast allergy (Part B only) ... 49
8.5.3 Exclusion of participants with allergy to amide type local anesthetics ... 49
8.6 Distinguishing intercurrent HIV infection from vaccine-induced positive serology ... 49
8.7 Immunogenicity evaluations ... 50
8.7.1 Humoral immunogenicity studies (HVTN) ... 50
8.7.1.1 Binding antibodies by ELISA (Part B only) ... 50
8.7.2 Cellular immunogenicity studies for Part A (HVTN) ... 50
8.7.2.1 IFN-γ ELISpot ... 50
8.7.2.2 Intracellular cytokine staining (ICS) ... 50
8.7.3 Cellular immunogenicity studies for Part B (HVTN) ... 51
8.7.3.1 IFN-γ ELISpot ... 51
8.7.3.2 Intracellular cytokine staining (ICS) for Part B ... 51
8.7.4 Ancillary studies ... 51
8.7.4.1 Humoral immunogenicity studies for Part A (Wyeth) ... 51
8.7.4.2 Humoral immunogenicity studies for Part B (Wyeth) ... 51
8.7.4.3 Cellular immunogenicity studies for Part A and Part B (Wyeth) ... 51
8.7.4.4 T-cell repertoire studies for Part B (Vanderbilt University) ... 52
8.8 HLA typing ... 52

9 Statistical considerations ... 53
9.1 Overview ... 53
9.2 Objectives ... 53
9.3 Endpoints ... 53
9.3.1 Safety ... 53

9.3.2 Immunogenicity 53
9.3.3 Social impacts 53
9.4 Accrual and sample size 53
9.4.1 Sample size calculations for safety 54
9.4.2 Sample size calculations for immunogenicity 55
9.5 Statistical analysis 56
9.5.1 Analysis variables 56
9.5.2 Baseline comparability 56
9.5.3 Safety analysis 57
9.5.4 Immunogenicity analysis 57
9.5.4.1 Missing data 58
9.5.5 Social impact analysis 58
9.5.6 Analyses prior to end of study 58
9.6 Randomization of treatment assignments 59

Study operations

10 Protocol conduct 62
11 Informed consent 63
11.1 Screening consent form 63
11.2 Protocol-specific consent form 63
11.3 Assessment of understanding 63
12 Procedures 65
12.1 Pre-enrollment procedures 65
12.2 Post-enrollment procedures 66
12.3 Total blood volumes 66
12.4 Laboratory procedures 66
13 Study product preparation and administration 67
13.1 Schema and vaccine regimen 67
13.2 Study product formulation and preparation 69
13.2.1 HIV *gag* DNA Plasmid (HIV-1 *gag* DNA Vaccine, GENEVAX *gag-2962*) 69
13.2.2 *IL-12* DNA (*IL-12* adjuvant, GENEVAX *IL-12*-6285 Part A and GENEVAX *IL-12*-4532 Part B) 70
13.2.3 HIV CTL Peptides (HIV CTL Multi-Epitope Peptide, HIV Th/CTL Peptides, HIV CTL MEP, MEP) 70
13.2.4 RC529-SE (labeled as RC-529 SE 500 mcg/mL in 10% Squalene) 70
13.2.5 Peptide Diluent (Diluent for HIV CTL Peptides, Sodium Succinate diluent) 70
13.2.6 rGM-CSF (GM-CSF, Leukine®) 70
13.2.7 Placebo (Sodium Chloride Injection USP, 0.9%, NaCl 0.9%) 70
13.2.8 Preparation of HIV *gag* DNA Plasmid 1500 mcg or Placebo (Groups 1 and 5) 71
13.2.9 Preparation of HIV *gag* DNA Plasmid 1500 mcg / *IL-12* DNA 100 mcg or Placebo (Group 2) 71
13.2.10 Preparation of HIV *gag* DNA Plasmid 1500 mcg / *IL-12* DNA 500 mcg or Placebo (Group 3) 72
13.2.11 Preparation of HIV *gag* DNA Plasmid 1500 mcg / IL-12 DNA 1500 mcg or Placebo (Group 4) 72
13.2.12 Preparation of HIV *gag* DNA Plasmid 1500 mcg / IL-12 DNA 1500 mcg or Placebo (Groups 6 and 7) 73
13.2.13 Preparation of MEP 1000mcg / RC529-SE 50mcg / GM-CSF 250 mcg or Placebo (Group 7) 74
13.2.14 Pharmacy procedures to preserve blinding 74

13.3 Study product administration 74
13.3.1 Administration of vaccinations 75
13.4 Study product acquisition 75
13.5 Pharmacy records 75
14 Safety monitoring and review 76
14.1 Assessing reactogenicity 76
14.2 Grading adverse events 76
14.3 Adverse events reporting and safety pause rules 76
14.3.1 Follow up and resolution after a safety pause 78
14.4 Expedited adverse event reporting 78
14.4.1 EAE reporting level 79
14.4.2 Study products for expedited reporting to DAIDS 79
14.4.3 Grading severity of events 79
14.4.4 EAE reporting periods 79
14.4.5 Biosafety concerns: Recombinant DNA Advisory Committee and Institutional Biosafety Committee review and reporting 79
14.5 Participant departure from schedule of clinic visits or vaccinations 80
14.5.1 Delaying vaccinations for a participant 80
14.5.2 Stopping vaccinations for a participant 80
14.5.3 Participant termination from the study 81
14.6 Study termination (for all participants) 81
14.7 HVTN review of cumulative safety data 81
14.7.1 Daily review 82
14.7.2 Weekly review 82
14.7.3 Quarterly review 82
14.7.4 Safety Monitoring Board review 82
14.7.5 Review for dose escalation 83
14.7.6 Review for advancing from Part A to Part B 83
References 84
Protocol history 91
Protocol team 92

Appendices

Appendix A: Sample informed consent form 94
Appendix B: Laboratory procedures for Part A 115
Appendix C: Laboratory procedures for Part B 116
Appendix D: Procedures at HVTU 117

Tables and figures

Figure 1-1 Coordination of product trials 17
Table 2-1 Structural differences between *gag* plasmids APL-400-047 and GENEVAX® *gag*-2962 plasmid....22
Table 2-2 Sequence of Th/CTL peptides 23
Table 2-3 Epitopes and allele restriction of peptides A, B, C, and J....24
Table 3-1 Summary of toxicology studies with human *IL-12* DNA....28
Table 3-2 IFN-γ ELISpot responses (per 10^6 cells) in monkeys immunized with SIV *gag* DNA and rhesus *IL-12* DNA....32
Table 3-3 Study design of rhesus macaque study 34
Table 3-4 Percent p11C-tetramer staining and functional p11C-specific CTL responses after 14-day *in vitro* peptide p11C stimulation 34
Table 3-5 Study design: evaluation of HIV CTL MEP and HIV DNA vaccine in cynomolgus macaque 35
Figure 3-1 Total IFN-γ response to vaccine antigen in macaques primed with plasmid, and boosted with peptide (Group 2)....36
Figure 3-2 Total IFN-γ response to vaccine antigen in macaques primed with peptide and boosted with plasmid (Group 1)....37
Figure 3-3 Total IFN-γ response to vaccine antigen from animals co-administered peptide plus plasmid for prime and boost immunizations (Group 3)....37
Table 4-1 HVTN 056: Adverse events at least possibly related to investigational product, all injections grouped (blinded data), N=52....40
Table 7-1 Study inclusion criteria....46
Table 7-2 Study exclusion criteria 47
Table 9-1 Probability (%) of response for different safety scenarios 54
Table 9-2 Exact 95% 2-sided confidence intervals for response rates based on observing a particular rate of responses in the vaccinees (n=30) in Part B. 55
Table 9-3 Minimum detectable differences in response rates between 2 groups (of size 30)....55
Table 9-4 Probability of 90% (80%) correct selection of regimen with highest immunology response (n=30 per group) among 3 groups 56
Table 12-1 Pre-enrollment procedures....65
Table 12-2 Post-enrollment procedures 66
Table 14-1 Schedule of reactogenicity assessments 76
Table 14-2 Adverse experience notification and safety pause rules for Part A 77
Table 14-3 Adverse experience notification and safety pause rules for Part B 78
Protocol history and modifications 91

Schema

Study products

gag DNA: HIV-1 *gag* DNA vaccine at 1500 mcg per dose (formulated with bupivacaine)

IL-12 DNA: *IL-12* DNA adjuvant at 100, 500 and 1500 mcg per dose (formulated with bupivacaine)

CTL MEP vaccine: CTL multiepitope peptide vaccine (HIV CTL MEP) at 1000 mcg total dose (250 mcg per peptide × 4 peptides), including RC529-SE adjuvant at 50 mcg per dose and Granulocyte-macrophage colony-stimulating factor (GM-CSF) cytokine adjuvant at 250 mcg per dose. *Note: future references to CTL MEP vaccine include adjuvants.*

Placebo: Sodium chloride injection USP, 0.9%

Administration: *gag* DNA or placebo: 0.75 mL injected intramuscularly in the deltoid by needle and syringe (Groups 1, 5)

gag DNA + *IL-12* DNA or placebo: 0.8 mL (Group 2), 1 mL (Group 3), or 1.5 mL (Groups 4, 6, 7) injected intramuscularly in the deltoid by needle and syringe.

CTL MEP/RC529-SE/GM-CSF or placebo: 1 mL injected intramuscularly in the deltoid by needle and syringe (Group 7)

					Vaccination schedule in months (days)				
		Dose (mcg)			Priming			Boosting	
Groups	N	gag	IL-12	CTL MEP	0 (0)	1 (28)	3 (84)	6 (168)	9 (273)
Part A									
1	10	1500	—	—	*gag* DNA	*gag* DNA	*gag* DNA	—	—
	2	—	—	—	placebo	placebo	placebo	—	—
2	10	1500	100	—	*gag* DNA + *IL-12* DNA	*gag* DNA + *IL-12* DNA	*gag* DNA + *IL-12* DNA	—	—
	2	—	—	—	placebo	placebo	placebo	—	—
3	10	1500	500	—	*gag* DNA + *IL-12* DNA	*gag* DNA + *IL-12* DNA	*gag* DNA + *IL-12* DNA	—	—
	2	—	—	—	placebo	placebo	placebo	—	—
4	10	1500	1500	—	*gag* DNA + *IL-12* DNA	*gag* DNA + *IL-12* DNA	*gag* DNA + *IL-12* DNA	—	—
	2	—	—	—	placebo	placebo	placebo	—	—
Part B									
5	30	1500	—	—	*gag* DNA	*gag* DNA	*gag* DNA	*gag* DNA	*gag* DNA
	6	—	—	—	placebo	placebo	placebo	placebo	placebo
6	30	1500	1500	—	*gag* DNA + *IL-12* DNA	*gag* DNA + *IL-12* DNA	*gag* DNA + *IL-12* DNA	*gag* DNA + *IL-12* DNA	*gag* DNA + *IL-12* DNA
	6	—	—	—	placebo	placebo	placebo	placebo	placebo
7	30	1500	1500	1000	*gag* DNA + *IL-12* DNA	*gag* DNA + *IL-12* DNA	*gag* DNA + *IL-12* DNA	CTL MEP	CTL MEP
	6	—	—		placebo	placebo	placebo	placebo	placebo
Total	48 (Part A) + 108 (Part B) = 156								

Enrollment in Groups 1 and 2 will occur simultaneously. See Section 8.2 regarding initial safety evaluation.

Enrollment in Groups 3 and 4 will be sequential. See Section 8.3 for dose escalation criteria.

Enrollment in Groups 5, 6, and 7 will occur simultaneously. See Section 8.4 regarding safety evaluation for moving from Part A to Part B.

Summary

Title

A Phase I clinical trial to evaluate the safety and immunogenicity of an HIV-1 *gag* DNA vaccine with or without *IL-12* DNA adjuvant, boosted with homologous plasmids or with HIV CTL multiepitope peptide vaccine / RC529-SE plus GM-CSF, in healthy, HIV-1 uninfected adult participants

Participants

Healthy HIV-1 uninfected adult participants (18 to 50 years old)

Number of participants

Part A: 48 (40 vaccinees, 8 placebo recipients)

Part B: 108 (90 vaccinees, 18 placebo recipients)

Total: 156 (130 vaccinees, 26 placebo recipients)

Primary objectives

Part A

- To evaluate the safety and tolerability of intramuscular administration of HIV-1 *gag* DNA vaccine
- To evaluate the safety and tolerability of intramuscular administration of HIV-1 *gag* DNA vaccine plus *IL-12* DNA adjuvant (at escalating doses of 100 mcg, 500 mcg, and 1500 mcg)

Part B

- To further evaluate the safety and tolerability of intramuscular administration of HIV-1 *gag* DNA vaccine following a priming series and booster vaccinations with homologous plasmid
- To further evaluate the safety and tolerability of intramuscular administration of HIV-1 *gag* DNA vaccine plus *IL-12* DNA adjuvant as a priming series followed by booster vaccinations with homologous plasmids or HIV CTL MEP/RC529-SE/GM-CSF

Study product descriptions

HIV-1 gag *DNA vaccine*

The HIV-1 *gag* DNA vaccine contains an RNA-optimized truncated *gag* gene (p37) derived from strain HXB2. The plasmid backbone includes a eukaryotic gene expression unit that contains elements from the human cytomegalovirus (hCMV) immediate early promoter/enhancer and the bovine growth hormone (BGH) polyadenylation signal, a chimeric kanamycin resistance gene, and a pUC bacterial origin of replication. The vaccine is formulated in 30 mM citrate buffer pH 6.5 containing 150 mM NaCl, 0.01% ethylenediamine tetraacetic acid (EDTA), and 0.25% bupivacaine-HCl.

IL-12 *DNA adjuvant*

The *IL-12* DNA adjuvant is a dual promoter expression plasmid which expresses the genes encoding human IL-12 proteins p35 and p40 under separate regulatory control. The p35 subunit is under the control of the hCMV promoter/enhancer and the SV40 (simian virus 40) polyadenylation signal. The p40 subunit is under the control of the SCMV (simian cytomegalovirus) promoter and the BGH (bovine growth hormone) polyadenylation signal. The plasmid contains a chimeric kanamycin resistance gene and a pUC bacterial origin of replication. The plasmid adjuvant is formulated in 30

mM citrate buffer pH 6.5 containing 150 mM NaCl, 0.01% ethylenediamine tetraacetic acid (EDTA), and 0.25% bupivacaine-HCl.

HIV CTL MEP vaccine

The HIV CTL multiepitope peptide (HIV CTL MEP) is a mixture of 4 peptides. Each hybrid peptide contains 1 of 3 different HIV T helper epitopes (derived from *env* or *gag*) in combination with 1 of 4 different CTL "hot spots," regions of Clade B Gag and Nef proteins that contain multiple overlapping CTL epitopes. The tetravalent peptides are formulated at 625 mcg/mL of each peptide in 3% mannitol and 12.5 mM succinic acid, pH2. The lyophilized preparation is reconstituted with a sodium succinate diluent. The MEP is given with RC529-SE adjuvant and granulocyte-macrophage colony-stimulating factor (GM-CSF). The combination of HIV CTL MEP and RC529-SE and GM-CSF is referred to as the HIV CTL MEP vaccine.

Placebo

Sodium Chloride Injection USP, 0.9%

Study design

Multicenter, randomized, placebo-controlled, double-blind trial

Study duration

Part A: 9 months per participant

Part B: 15 months per participant

Safety monitoring

HVTN 060 Protocol Safety Review Team

HVTN Safety Monitoring Board

Vaccine provider

Wyeth Vaccines Research (Pearl River, New York, USA)

Sponsor

Division of AIDS (DAIDS), National Institute of Allergy and Infectious Diseases (NIAID), National Institutes of Health (NIH), Department of Health and Human Services (DHHS) (Bethesda, Maryland, USA)

IND holder

DAIDS, NIH

Study sites

US and non-US HIV Vaccine Trials Units (HVTUs) to be selected contingent on site preparedness

HVTN Core Operations

HVTN Vaccine Leadership Group/Core Operations Center, Fred Hutchinson Cancer Research Center (FHCRC) (Seattle, Washington, USA)

Statistical and data management center

Statistical Center for HIV/AIDS Research and Prevention (SCHARP), FHCRC

Central laboratories

Duke University Medical Center, *Durham, North Carolina, USA*

FHCRC/University of Washington, *Seattle, Washington, USA*

Viral and Rickettsial Disease Laboratory, *Richmond, California, USA*

South Africa Immunology Laboratory and National Institute for Communicable Disease, *Johannesburg, South Africa*

Protocol leadership

Chair: Spyros Kalams MD, *Vanderbilt University, Nashville, Tennessee, USA*

Co-Chair: Scott Parker MD, University of Alabama at Birmingham, *Birmingham, Alabama, USA*

Clinical Trials Physicians: Marnie Elizaga MD, HVTN Core, *Seattle, Washington, USA*

Statistician: Barbara Metch MS, SCHARP, *Seattle, Washington, USA*

DAIDS Medical Officer: Maria Allende MD, DAIDS, *Bethesda, Maryland, USA*

Introduction

The ongoing worldwide epidemic of the human immunodeficiency virus type 1 (HIV-1) remains one of the major global health challenges. HIV-1 causes the acquired immunodeficiency syndrome (AIDS), which is responsible for tremendous human suffering and economic loss throughout the world. Currently, over 39 million people are living with HIV-1 infection [1]. Without treatment, it is likely that nearly all of these will die of AIDS in the next 2 decades.

Since 1996, potent new antiretroviral therapies, including combination regimens with protease inhibitors, have created the possibility that HIV-1 infection might become a chronic, manageable disease among individuals with access to these medications. In the US, AIDS deaths are down to 18,000 per year as a result of the new antiretrovirals [2]. However, for the developing world, where over 98% of the nearly 5 million annual incident HIV-1 infections occur [1], it is unlikely that these drugs will be widely accessible, due to many logistical challenges associated with their use.

Globally, 13,000 new infections occur each day. More than 3 million AIDS deaths occur per year [1], and nearly 20 million have died since the HIV epidemic began [3]. AIDS has become the leading infectious disease killer, the fourth leading cause of death overall. In severely affected countries, life expectancy has fallen by more than 10 years [1]. AIDS is the leading killer in Africa, with over 25 million Africans living with HIV/AIDS. Sub-Saharan Africa has been affected most; in 7 Sub-Saharan African countries, over 22 million adults (aged 15-49) are living with HIV/AIDS [3]. For example, in Botswana, 37.3% of adults aged 15 to 49 are infected with HIV, while in South Africa more than 25% of women in antenatal clinics are infected [3].

After sub-Saharan Africa and Asia, Latin America is the region most severely affected by HIV infection. The HIV epidemic in Latin America reflects diverse transmission patterns: in Andean countries HIV is most often transmitted sexually, primarily among men who have sex with men (MSM), while in Brazil, Uruguay and Argentina a significant proportion (39% of AIDS cases in Argentina) of HIV transmission occurs through injection drug use (IDU). In all countries of the Andean Region, MSM account for a substantial proportion of HIV infections and comprise a “bridge” group for spread into the heterosexual population due to the high frequency of bisexuality. HIV incidence is higher among high-risk MSM in Brazil and Peru compared to most U.S. populations [4-9].

The need for better education, better treatment access, better prevention programs, and better prevention technologies is therefore clear. Specifically, the need for a safe, effective, and affordable HIV-1 vaccine is paramount [10,11]. The ideal HIV-1 vaccine for global use should meet several of the following criteria:

- proven safety in healthy HIV-uninfected persons
- induction of long-lasting HIV-specific cell-mediated and humoral immunity capable of conferring protection against HIV
- tolerability
- potential for production in sufficient quantity to meet global needs
- affordability
- stability during distribution and storage

Ethical considerations

Multiple candidate HIV vaccines will need to be studied simultaneously in different populations around the world before a successful HIV preventive vaccine is found. It is critical that universally accepted ethical guidelines are followed at all sites involved in the conduct of these clinical trials. The HVTN has addressed ethical concerns in the following ways:

- HVTN trials are designed and conducted to enhance the knowledge base necessary to find a preventive vaccine, with methodology that is scientifically rigorous and valid, and in accordance with Good Clinical Practice (GCP) guidelines.
- HVTN scientists and protocol team members incorporate the philosophies underlying major codes, declarations, and other guidance documents relevant to human subject research into the design and conduct of HIV vaccine clinical trials.
- HVTN scientists and protocol team members are committed to substantive community input into the planning, conduct, and follow up of the research which will help ensure that locally appropriate cultural and linguistic needs of study populations are met.
- The HVTN advocates that all HVTN sites should develop a plan for the care and treatment of participants who develop HIV infection during a trial. This plan should be formulated by representatives of host countries, communities from which potential trial participants will be drawn, sponsors, and the HVTN.
- Prior to implementation, HVTN trials are rigorously reviewed by both local and national regulatory bodies, in addition to scientists who have no involvement with the trial under consideration.
- The HVTN recognizes the importance of institutional review and values the role of in-country Institutional Review Boards (IRBs) and Institutional Ethics Committees (IECs) as custodians responsible for ensuring the ethical conduct of research in the local setting.
- The HVTN provides training so that all participating sites similarly ensure fair subject selection, protect the privacy of research subjects, and obtain meaningful informed consent.

STUDY PRODUCTS

1 Products background

Despite the major strides that have been made in HIV therapy with the advent of potent antiretroviral drugs, these medications are quite expensive and are still not readily available for the vast majority of infected individuals worldwide. Even when the medications are available, the associated long-term toxicities and the frequent emergence of drug-resistance mutations can complicate therapy, making the formulation of effective vaccines imperative. Although it is unclear at present which vaccine-induced immune responses will protect against infection, the evidence to date suggests that vaccine-induced cellular immune responses are able to control viremia and prevent disease progression in animal infection models. The ability to measure immune responses has also advanced markedly over the past few years and will allow investigators to more accurately measure the immunogenicity of vaccine constructs, and correlate the magnitude and breadth of these responses with protection.

Cytotoxic T lymphocytes (CTL) are generated early during the course of acute HIV-1 infection. After infection of CD4+ T cells, viral proteins that are generated in the cytosol are degraded and presented as epitopic peptides (usually 9 to 11 amino acids in length) on the cell surface complexed to HLA Class I molecules. CTL recognize infected cells through the interaction of the T cell receptor (TCR) with the HLA-epitope complex. This occurs prior to the assembly of progeny virions, a process that takes approximately 2.6 days. During this time, an infected cell is vulnerable to attack by CTL, and if eliminated at this time, progeny virus will not be released [12,13]. CTL are also able to mediate an antiviral effect through the elaboration of soluble factors that inhibit viral replication. These include the chemokines RANTES, MIP-1alpha and MIP-1beta, as well as other factors not yet fully defined. The release of these factors occurs when the TCR recognizes an infected cell. In fact, these factors are released concurrently with the mobilization of the cell's cytolytic machinery when an infected cell is recognized [14], and this likely has an important effect on the microenvironment of the infected cell. RANTES, MIP-1alpha and MIP-1beta have been shown to inhibit HIV infection of cells by competing with the virus for chemokine coreceptors present on the cell surface that are necessary for viral entry. Although the exact contribution of each of these two mechanisms toward suppression of HIV infection *in vivo* is not clear, it is likely that they act synergistically to contain cell-to-cell spread of HIV.

The generation of cytotoxic T lymphocyte responses after exposure to viral pathogens involves a complex relationship between antigen presenting cells and T cells. Chemokines such as MIP-1alpha and MIP-1beta (among others) recruit T and B cells to sites of inflammation, where dendritic cells interact with naïve T cells [15]. These interactions lead to CD40L upregulation on CD4+ T cells, which leads to upregulation of CD40 [16], costimulatory molecules , and the release of cytokines such as IL-12 and IL-15 from mature dendritic cells [17]. This series of events has been shown to be fundamental to the generation of high quality and high magnitude CD4+ Th and CD8+ CTL responses.

Wyeth Research is pursuing development of a combination HIV vaccine consisting of CTL multiepitope peptide (HIV CTL MEP) and facilitated DNA technology (HIV-1 *gag* DNA + *IL-12* DNA) platforms. The HIV CTL MEP and *gag* DNA + *IL-12* DNA vaccine approaches are being examined in integrated Phase I prime/boost studies. In HVTN 060, priming with *gag* DNA + *IL-12* DNA will be followed by boost vaccination(s) with HIV CTL MEP/RC529-SE/GM-CSF or with *gag* DNA + *IL-12* DNA, or gag DNA only. HVTN 061 will examine the converse strategy of boosting HIV CTL MEP / RC529-SE vaccinees from HVTN 056 with HIV CTL MEP/ RC529-SE, HIV CTL MEP/RC529-SE plus GM-CSF

or *gag* DNA + *IL-12* DNA. The 3 studies are presented diagrammatically in Figure 1-1. Detailed descriptions of the individual components of the vaccine are presented in Section 2. Here we provide additional background information supporting the use of the *gag* DNA + *IL-12* DNA.

Figure 1-1 Coordination of product trials

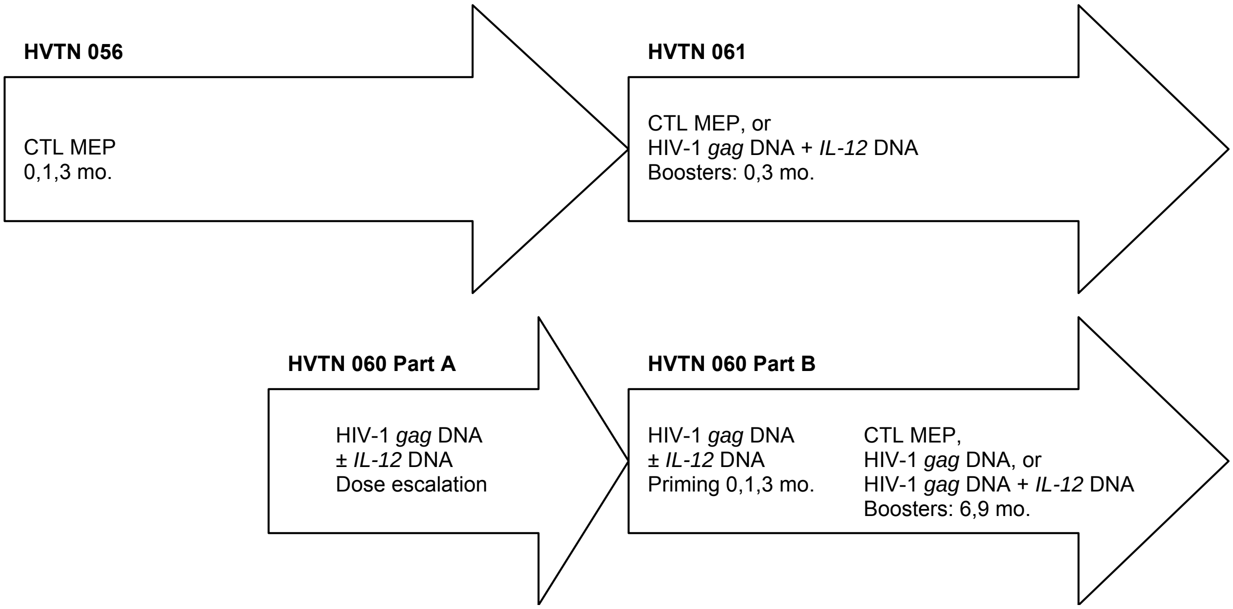

Note: HVTN 056 and 061 peptides are tested with and without GM-CSF

1.1 Plasmid DNA vaccination

Plasmid DNA vaccination is a novel vaccination modality that has been shown to elicit both humoral and cellular immune responses in animal models. DNA vaccines are also simple and inexpensive to construct, readily produced in large quantities, and stable for long periods of time. If DNA vaccination proves to be efficacious, production and delivery to individuals in developing nations may be more economically and logistically feasible than with other types of vaccines.

Plasmid DNA vaccination involves the administration of purified plasmid DNA encoding an antigen. Plasmid DNA is typically injected into skeletal muscle or, alternatively, inoculated as plasmid-coated beads by gene gun into the epidermis. The protein is expressed in

transfected mammalian cells, including macrophages and dendritic cells; enters into both the MHC Class I and Class II processing pathways; and elicits strong and persistent humoral and cellular immune responses [18-21].

Results of DNA vaccine clinical trials for a number of infectious diseases have been reported. A Phase I study of a malaria gene-based DNA vaccine demonstrated that CTLs and IFN-alpha–producing T lymphocytes could be induced in human participants [22]. In a Phase I trial in 20 healthy adult participants to test the safety, tolerability, and immune responses to a DNA vaccine for malaria (containing a backbone very similar to that being proposed in the current study) there were no severe or serious adverse experiences and no significant clinical or laboratory findings [23]. Anti-dsDNA antibodies were not detected. Furthermore, CTLs reactive with the expressed *Plasmodium falciparum* circumsporozoite (*Pf*CSP) protein were observed, although no antigen-specific antibodies were detected. In a Phase I study of a DNA vaccine encoding hepatitis B surface antigen, the vaccine was delivered by the gold bead particle acceleration PowderJect XR1 gene gun into 6 hepatitis B seronegative subjects and 1 seropositive subject. Although none of the seronegative subjects developed primary immune responses in the form of antibodies, the vaccine was well tolerated with minimal, mostly local reactions [24]. Overall, DNA vaccination in humans is a promising vaccination modality that has achieved a good safety record but has not yet achieved its full potential in terms of immunogenicity.

DNA vaccines have shown partial efficacy in viral challenge studies in nonhuman primates. SIV *env* DNA vaccination of rhesus monkeys reduced viral loads following challenge with the virulent SIVmac251 isolate in rhesus monkeys [25]. Furthermore, SIV *gag* DNA vaccination of rhesus monkeys led to the development of potent CTL responses and reduced viral loads following intravenous challenge with the pathogenic SIVmn E660 virus [26].

These and other nonhuman primate studies [27,28] have prompted clinical trials of DNA-based HIV vaccines.

1.2 DNA-based HIV vaccines in humans

The first clinical trial of a DNA vaccine in HIV seropositive individuals was conducted by Wyeth (Apollon) and evaluated the intramuscular injection of a DNA vaccine expressing the Env and Rev proteins. This study provided extensive safety data and revealed no significant adverse clinical or laboratory findings in any of the subjects. In particular, there was no evidence of antinuclear antibody formation, muscle enzyme elevation, or changes in blood pressure and pulse [29].

After encouraging results from this preliminary study, a blinded Phase I study of the Env- and Rev-encoding DNA vaccine (GENEVAX HIV, Apollon) was undertaken in healthy, uninfected subjects [30]. The vaccine was given at a dose of 100 mcg, 300 mcg or 1000 mcg by intramuscular injection. Participants demonstrated modest but sporadic T lymphocyte immune responses, but the conclusions were limited by the small number of subjects (6 per group). In both studies, the plasmid was administered with bupivacaine to enhance DNA uptake, gene expression, and immune responses. Results of the study suggested that further investigations should be undertaken with higher doses and enhancements with different formulation or combination strategies.

1.3 Cytokine-augmented DNA vaccination

The safety and partial efficacy of DNA vaccination in nonhuman primate studies has led to a number of approaches to augment the efficacy of this vaccine modality. One such strategy

involves the boosting of a DNA-primed immune response with a recombinant live vector. A second promising strategy involves the coadministration of plasmid-encoded immunomodulator molecules. Augmentation of DNA vaccine–elicited HIV-specific cellular immune responses in mice has been reported by a number of laboratories using plasmids expressing GM-CSF, IL-2, CD40L, IL-12, IL-15, B7-2, ICAM-1, LFA-3, RANTES, MIP-1 alpha, or MCP-1 [31-35].

Cytokines are molecules secreted mainly by bone marrow–derived cells that act in an autocrine or paracrine manner to induce a specific response in cells expressing the appropriate cytokine receptor. Recent vaccine strategies have attempted to incorporate cytokine-expressing plasmids with the viral genes of interest in efforts to elicit cell-mediated immune responses. Initial murine studies have shown that both GM-CSF and *IL-12* plasmids were able to enhance CTL responses to a peptide-based vaccine [36]. Subsequent studies evaluating plasmids encoding GM-CSF or *IL-12* have shown enhancement of CTL responses [37-39], and protection from disease after vaccinia challenge [40]. While there are few published studies evaluating vaccine immunogenicity in primates, van der Meide *et al.* found that recombinant *IL-12* augmented cellular immune responses in a macaque model [41].

These published studies, in combination with the preliminary results summarized in this protocol, provide the rationale to pursue HIV *gag* and *IL-12* plasmid–based vaccines in this first in human study to determine whether DNA encoding IL-12 is safe, and whether it provides an adjuvant effect designed to augment HIV-specific CTL responses.

1.4 IL-12 background

IL-12 was first described in 1989 as a cytokine capable of stimulating natural killer (NK) cell and cytotoxic lymphocyte maturation [42-44]. IL-12 is a heterodimeric cytokine composed of a heavy chain of 40 kDa and a light chain of 35 kDa that are covalently linked by a disulfide bond [45].

IL-12 is produced primarily by phagocytic cells, including monocytes, macrophages, and neutrophils [46]. To lesser degrees, dendritic cells, keratinocytes, and nonmucosal mast cells also possess the ability to produce IL-12. Both a T cell independent pathway (exposure to microbes or their components) or a T cell dependent pathway (interaction of CD40 ligand on activated T cells with CD40 on IL-12 producing cells) can stimulate IL-12 production [47]. Both intracellular and extracellular pathogens are capable of inducing IL-12 production from phagocytic cells. In addition, the gp120 glycoprotein of HIV has been shown to stimulate IL-12 production [48].

The major cells influenced by IL-12 are T cells and NK cells, resulting in cytokine production, proliferation of activated T lymphocytes, and enhanced NK cell proliferation [42,49-52]. The cytolytic activity of cytotoxic T and NK cells is enhanced by IL-12. IL-12 is required for the optimal proliferation of mitogen- or antigen-stimulated T cells. IL-12 increases IFN-γ production from NK and T cells, enhances NK/lymphokine-activated killer cell cytotoxicity, and promotes Th1 immune responses [52,53].

IL-12 is important in influencing the differentiation of Th1 cells from uncommitted precursor cells in response to signals derived from the innate immune system and inhibiting Th2 cell differentiation. Once Th1 cells develop, IL-12 further enhances the Th1 response by promoting activity of this cell subset.

IL-12 stimulates cellular immune responses and thus may serve to stimulate HIV-specific immunity or enhance general cell-mediated immune responses. Both these actions may improve immune responses to HIV vaccines. The capacity of IL-12 to promote Th1 responses

has led to interest in its role as a vaccine adjuvant for diseases requiring cellular immune responses. In a mouse model of Leishmaniasis, a protective Th1 cell response was induced by vaccination with parasite extracts in combination with IL-12. NK cell activation and IFN-γ production were seen in vaccinated mice that protected normally susceptible mice from parasite challenge [54]. Other studies in mice have also demonstrated the development of protective immune responses to a *Mycobacterium tuberculosis* subunit vaccine, to doses of heat-killed *Listeria monocytogenes* or listerial antigen preparations, and to an acellular pertussis vaccine when given with IL-12 [55-57]. In each of these studies Th1 responses to the vaccine were amplified by the addition of IL-12.

IL-12 may also exert effects on antibody responses. In mice, IL-12 administration has led to a shift in the isotype of antigen specific antibodies. IFN-alpha promoted the differentiation of B cells to produce cytophilic antibodies in mice (IgG2a, IgG2b, IgG3) and decreased expression of IgG1, IgE, and IgA. IL-12 as a vaccine adjuvant may modulate the type of antibody response toward those isotypes that are protective [58,59].

1.5 Recombinant human IL-12 (rhIL-12) protein

1.5.1 Clinical studies of rhIL-12 protein in therapeutic settings

Although human clinical trial data are not available for the *IL-12* DNA adjuvant, recombinant human IL-12 (rhIL-12) protein has been used extensively in various therapeutic and prophylactic settings. Over 1100 subjects have received 1 or more doses of rhIL-12 given in therapeutic trials for oncology, HIV disease, hepatitis C virus infection, and hepatitis B virus infection, as well as for malaria and asthma.

For reference purposes, the Investigator's Brochure for Recombinant Human IL-12 will be provided as an attachment in the *IL-12* plasmid Investigator's Brochure. Details of this clinical experience are not provided in this review.

1.5.2 Clinical studies of rhIL-12 protein as vaccine adjuvant

Several studies have demonstrated the potential of rhIL-12 as an adjuvant to specifically modulate the characteristics of a developing antibody response by enhancing expression of isotypes associated with efficacy in infectious diseases and suppressing others that may be associated with pathology. In addition, they suggest the potential for the use of IL-12 to modify the antibody response at specific sites to modulate the characteristics of an ongoing immune response. IL-12 has also been shown to enhance the absolute levels of antigen-specific antibody in serum. The mechanism for enhanced antibody production remains to be determined but may reflect expansion of T helper cells and enhanced cytokine expression. Wyeth has conducted a number of clinical studies with rhIL-12 as a vaccine adjuvant as described in the Investigator's Brochure. Safety and tolerability data from these studies support the safety of up to 2 mcg of rhIL-12 and the likely tolerability of this dose. Those studies also revealed that rhIL-12 had a modest stimulatory effect when given with T cell dependent vaccines. At doses higher than 4 mcg of rhIL-12, an unacceptable level of adverse experiences was noted. These included headache, injection site pain, myalgia, fever, asthenia, lymphadenopathy, and other symptoms; please refer to the Investigator's Brochure for more details of these reactions. In light of these adverse experiences, rhIL-12 is being pursued only at levels of 2 mcg or below as a vaccine adjuvant. In this study, rhIL-12 is not being used, and the safety of *IL-12* DNA will be assessed.

1.5.3 *IL-12* DNA compared to rhIL-12 protein

In comparative studies in mice using *IL-12* DNA therapy and IL-12 protein therapy against various tumors, *IL-12* DNA therapy proved to be as efficient as the IL-12 protein therapy, and induced far less toxic side effects [60]. Preclinical studies conducted using intratumoral *IL-12* DNA therapy showed that this treatment can induce a striking anti-tumor response in various murine tumor models, including melanoma, sarcoma and adenocarcinoma [60-63]. The lack of severe side effects of *IL-12* DNA therapy in this animal model suggested that it may be a safe alternative to IL-12 protein therapy for certain human cancers.

1.6 *IL-12* DNA plasmid as a vaccine adjuvant

Modulation of the immune response by coadministration of cytokine plasmids is one of the most promising approaches under investigation aimed at enhancing the immunogenicity of DNA vaccines. Coinjection of cytokine plasmids has been shown by several laboratories to enhance immune responses. Seminal studies of *IL-12* DNA combined with HIV DNA vaccines were performed in rodents by the Weiner Laboratory [33]. These studies demonstrated a dramatic increase in specific CTL activity when a *gag/pol* plasmid or an *env* plasmid was coadministered with an *IL-12* plasmid, as compared with results in animals receiving *env* or *gag/pol* plasmids alone. The molecular adjuvant activity of several Th1 cytokines (GM-CSF, IL-2, IL-12, IL-15, and IL-18) was then evaluated in mice in a subsequent study by the Weiner group [64]. This study revealed that the *IL-12* plasmid was the best driver of MHC-restricted CD8+ CTL activity. Codelivery of *IL-12* DNA and HIV DNA vaccines was also evaluated by the Weiner Laboratory in chimpanzees [65]. DNA immunogens were administered at 500 mcg/plasmid at weeks 0, 5 ,9 and 15. A chimpanzee receiving *gag/pol* and *IL-12* plasmid immunizations exhibited enhanced antigen-specific responses to multiple antigens at multiple timepoints, as compared with a chimpanzee that did not receive *IL-12* plasmid injections.

The use of plasmid *IL-12* as a molecular adjuvant has also been evaluated by other investigators. *IL-12* plasmid was shown to function effectively as an adjuvant in a murine plasmodium model [66], a Hepatitis C murine study [67], influenza virus model [68] and Feline leukemia virus model (FLV) [69]. Other investigators have reported an absence of adjuvant effect for *IL-12* plasmid when coadministered with DNA vaccines for leishmaniasis [70], influenza virus NP [71], and LaCrosse Virus [68]. Wyeth Research has evaluated *IL-12* DNA and *gag* DNA administration in 20 macaques and has not observed any adverse events related to *IL-12* DNA administration. Coadministration of *IL-12* DNA resulted in substantially enhanced cellular immune responses (see Section 3.2.1). In summary, the use of *IL-12* plasmid DNA as a molecular adjuvant has resulted in enhancement of cellular immune responses in a number of studies and has been safe. Taken together, the preclinical data support the evaluation of this adjuvant in human vaccine trials.

2 Study product descriptions

2.1 HIV-1 *gag* DNA vaccine

The GENEVAX® *gag*-2962 plasmid is highly purified, supercoiled, plasmid DNA containing an RNA-optimized truncated *gag* gene (p37) inserted into the DNA plasmid expression vector WLV-001M. The HIV-1 *gag* gene is derived from strain HXB2 but has been RNA-optimized by inactivating inhibitory sequences that allows high level Rev independent expression of the *gag* gene. The WLV-001M plasmid backbone consists of 3 genetic units. The first is a eukaryotic gene expression unit that contains genetic elements from the human cytomegalovirus (hCMV) immediate early promoter/enhancer and the bovine growth hormone (BGH) polyadenylation signal. The second component is a chimeric kanamycin resistance gene *(km^r)* that confers resistance to a limited number of aminoglycosides while enabling selection of bacteria containing the km^r plasmid. The third component is a pUC bacterial origin of replication (ori) that is required for the propagation of the plasmid during fermentation of bacteria.

The GENEVAX® *gag*-2962 plasmid is a second-generation Gag-expressing plasmid that was derived from *gag* plasmid APL-400-047 which was previously evaluated in the AVEG 031 clinical trial. GENEVAX® *gag*-2962 plasmid differs in structure from predecessor plasmid APL-400-047 as described in Table 2-1.

The vaccine is formulated in 30 mM citrate buffer pH 6.5 containing 150 mM NaCl, 0.01% ethylenediamine tetraacetic acid (EDTA), and 0.25% bupivacaine-HCl. Studies have shown that formulation of bupivacaine at a 0.25% concentration with plasmid DNA at levels of 800 mcg or higher results in quantitative association (100%) of bupivacaine with DNA [72]. Thus, as the gag, *IL-12* and *IL-15* DNA vaccine/molecular adjuvants are formulated at 2 mg/mL, bupivacaine in these formulated preparations is complexed with plasmid DNA and is not found in significant levels in an uncomplexed form as a free molecule.

Table 2-1 Structural differences between *gag* plasmids APL-400-047 and GENEVAX® *gag*-2962 plasmid

APL-400-047	GENEVAX® gag-2962 plasmid
Codes for both gag and pol genes from HIV-1 (HXB2 strain)	Codes for RNA optimized truncated gag (p37) from HIV-1 (HXB2 strain)
Presence of RSV enhancer	RSV enhancer has been removed for increased expression
SV40 poly A	BGH poly A
Absence of 5′ untranslated region of IE gene of hCMV	Incorporation of 5′ untranslated sequences from the IE gene of hCMV to enhance the promoter activity
Origin of replication sequence for low-copy plasmid	Single base change in the origin of replication sequence for high copy plasmid

In vitro expression analysis of GENEVAX® *gag*-2962 plasmid in mammalian cells demonstrated a significant enhancement in *gag* expression by GENEVAX® *gag*-2962 plasmid (up to 200-fold) over that observed with APL-400-047. GENEVAX® *gag*-2962 is formulated with 0.25% bupivacaine as a facilitating agent for DNA uptake.

2.2 *IL-12* DNA adjuvant

IL-12-6285 molecular adjuvant plasmid is a dual promoter expression plasmid consisting of 6259 nucleotides. It contains 2 cistrons that express the genes encoding human IL-12 proteins p35 and p40 under separate regulatory control. The p35 subunit is under the control of the hCMV promoter/enhancer and the SV40 (simian virus 40) polyadenylation signal whereas

the p40 subunit is under the control of the SCMV (simian cytomegalovirus) promoter and the BGH (bovine growth hormone) polyadenylation signal. The plasmid backbone of *IL-12*-6285 is identical to that of the GENEVAX® *gag*-2962 plasmid, as described above, except that it contains a single nucleotide difference in the origin of replication.

Note that *IL-12*-6285 DNA will be used for Part A of the present study. For Part B, *IL-12*-4532 will be used, based on plasmid. However, since sufficient *IL-12*-6285 is available to support only Part A of the study, a second lot of *IL-12* DNA, *IL-12*-4532, will be used to support Part B of this study. *IL-12*-4532 is identical to *IL-12*-6285 except for a single nucleotide change in the noncoding origin of replication region of the plasmid (identical plasmid backbone as that used for *gag*-2962). This nucleotide substitution is associated with an enhanced ability to produce the plasmid, and does not alter gene expression. These DNA plasmids will also be formulated with 0.25% bupivacaine as for the *gag* DNA vaccine.

2.3 HIV CTL MEP vaccine

2.3.1 HIV CTL MEP

The HIV CTL MEP consists of 4 CTL multiepitope peptides designated A, B, C, and J. These peptides range in length from 27 to 47 amino acids Table 2-2. Each hybrid peptide contains 1 of 3 different HIV T helper epitopes (and terminal amino acids sequence derived from Env or Gag) in combination with 1 of 4 different CTL "hot spots." All Gag CTL epitopes and CD4 epitopes in HIV CTL MEP are derived from similar sequences found in GENEVAX® *gag*-2962. The CTL "hot spot" peptides are regions of Clade B Gag and Nef proteins that contain multiple overlapping CTL epitopes. These "hot spots" were selected for use in the HIV CTL MEP vaccine as this combination of epitopes is restricted by 19 HLA types, providing theoretical coverage for 95% African Americans, 97% Thais, 98% Caucasian Americans, and 99% Native Americans. A partial listing of the CTL epitopes and their restricting HLA alleles is provided in Table 2-3.

Table 2-2 Sequence of Th/CTL peptides

**Peptide A**	H-Lys-Gln-Ile-Ile-Asn-Met-Trp-Gln-Glu-Val-Gly-Lys-Ala-Met-Tyr-Ala-Lys-Ala-Phe-Ser-Pro-Glu-Val-Ile-Pro-Met-Phe-OH
**Peptide B**	H-Tyr-Lys-Arg-Trp-Ile-Ile-Leu-Gly-Leu-Asn-Lys-Ile-Val-Arg-Met-Tyr-Ser-Asn-Pro-Pro-Ile-Pro-Val-Gly-Glu-Ile-Tyr-Lys-Arg-Trp-Ile-Ile-Leu-Gly-Leu-Asn-Lys-Ile-Val-Arg-Met-Tyr-Ser-Pro-Thr-Ser-Ile-OH
**Peptide C**	H-Asp-Arg-Val-Ile-Glu-Val-Val-Gln-Gly-Ala-Tyr-Arg-Ala-Ile-Leu-Val-Gly-Phe-Pro-Val-Arg-Pro-Gln-Val-Pro-Leu-Arg-Met-Thr-Tyr-Lys-OH
**Peptide J**	H-Lys-Gln-Ile-Ile-Asn-Met-Trp-Gln-Val-Val-Gly-Lys-Ala-Met-Tyr-Ala-Gly-Gln-Met-Val-His-Gln-Ala-Ile-Ser-Pro-Arg-Thr-Leu-Asn-Ala-Trp-Val-Lys-Val-Val-OH

Table 2-3 Epitopes and allele restriction of peptides A, B, C, and J

Allele	Sequence	Peptide
A01	VGFPVTPQVPLRMT	C
**A02**	KQIINMWQEVGKAMY	A
	**QVPLRPMTYK**	C
	**RVIEVLQRA**	C
	**TLNAWVKVV**	J
**A03**	**QVPLRPMTYK**	C
A05	QAISPRTLNAW	J
A11	**VPLRPMTYK**	C
A24	**IYKRWIIL**	B
A33	KRWIILGLNKIVMRY	B
B02	QAISPRTL	J
**B07**	**FPVTPQVPL**	C
	**TPQVPLRPM**	C
	**SPRTLNAWV**	J
**B08**	**EIYKRWII**	B
	VGFPVTPQVPLRMT	C
B14	GQMVHQAISPRTLNAWVKVV	J
B27	**KRWIILGLNK**	B
	**QVPLRPMTYK**	B
	**ISPRTLNAW**	B
B35	PPIPVGDIY	B
	**FPVRPQVPL**	C
	**VPLRPMTY**	C
B52	RMYSPTSII	B
B57	KAFSPEVI	A
	**ISPRTLNAW**	J
Bw62	LGLNKIVRMY	B

CTL epitopes in bold are those included in the vaccine for which tetramer reagents have been developed. Peptides A, B, and J contain epitopes from *gag*; peptide C contains epitopes from *nef*.

2.3.2 RC529-SE adjuvant

RC529-SE is a chemically synthesized analog of monophosphoryl lipid A (MPL) manufactured by Corixa Corporation (Seattle, WA). This analog shares strong adjuvant properties with MPL but exhibits reduced reactogenicity relative to MPL in rabbits and nonhuman primates. It is a member of the aminoalkylglucosamine phosphate family and possesses a monoglucosamine backbone with 3 fatty acyl side chains. It is solubilized in the oil phase of a stable oil-in-water emulsion. The SE emulsion contains egg-derived phosphatidyl choline as a stabilizer. The adjuvant activity of RC529-SE is attributed primarily to its ability to activate antigen-presenting cells and induce a cytokine cascade. Through the activation of these cells, vaccine antigens are more readily phagocytosed, processed, and presented. Animal studies demonstrate that RC529-SE and MPL exhibit a similar adjuvant profile and generate a strong Th1 immune response profile. RC529-SE, as part of the HIV CTL MEP vaccine, has been given to 24 volunteers to date in the Phase I trial, HVTN 056 (see Section 4.3). A Hepatitis B vaccine containing RC529 (without the SE emulsion) has recently been approved for use in humans in Argentina [73].

2.4 GM-CSF as a vaccine adjuvant

GM-CSF is a pleiotropic cytokine that enhances immune responses by attracting macrophages and triggering their maturation, thus resulting in increased antigen presentation. Several lines of evidence suggest that GM-CSF might be an effective adjuvant for a peptide vaccine. It induces Class II expression and activation of macrophages; it enhances DC1 maturation and migration; it promotes the influx of inflammatory cells locally, and has effects on maturation of hematopoietic progenitors in bone marrow [74-77]. GM-CSF has been evaluated as an adjuvant for viral vaccines in several Phase I trials. Its use as an adjuvant for hepatitis B vaccination in healthy individuals was reported by Hasan [78]. Individuals were given recombinant hepatitis B vaccine by intramuscular injection at 0, 1, and 6 months. The injections of GM-CSF were extremely well tolerated. Only a few moderate and no severe reactions were noted and there was little difference between adverse experiences seen in the GM-CSF and placebo arms of the trial. Although a trend towards an increase in protective titers was seen in the GM-CSF–treated group at some timepoints, the differences were not statistically significant. In another hepatitis B vaccine study conducted in healthy individuals, GM-CSF was safely administered at lower doses (20 and 40 mcg) when given by the IM, subcutaneous (SC) and intradermal routes. GM-CSF was safely administered by the SC route at a dose of 250 mcg to cancer patients when used as an adjuvant for influenza vaccine administration [79]. Additionally, the 250 mcg dose was administered safely by the SC route to healthy individuals given diphtheria toxoid, influenza or hepatitis A vaccines [80]. A number of studies have also evaluated SC administration of varying doses of GM-CSF in hemodialysis patients receiving Hepatitis B vaccine [81-85]. Enhanced Hepatitis B vaccine immunogenicity was observed in some trials. The GM-CSF vaccine was well-tolerated in all of the studies. None of the above studies evaluated anti-GM-CSF antibody responses.

GM-CSF has also been used as a vaccine adjuvant over a range of doses in several cancer vaccine studies. Weber *et al.* [86] recently reported its use in a multipeptide melanoma vaccine trial in melanoma patients. The study showed a trend for GM-CSF to modestly increase the immune response to the melanoma peptides. No meaningful differences were noted in the overall toxicities between the peptide/IFA (Incomplete Freund's Adjuvant) and peptide/IFA–GM-CSF arms of the trial. The toxicity of the peptide/IFA vaccine with GM-CSF was modest, transient, and was not related to the administration of GM-CSF. Similar safety profiles have been reported in other melanoma and colorectal carcinoma vaccine trials using GM-CSF as a vaccine adjuvant [87,88]. Taken together, the results show enhanced vaccine immunogenicity in some studies, but the vaccine/adjuvant combinations were uniformly well tolerated and safe.

GM-CSF is currently used as an adjuvant with the CTL MEP vaccine in protocol HVTN 056. See Section 4.3 for a summary of the current clinical data.

2.4.1 Antibodies to GM-CSF

Induction of GM-CSF antibody responses has been reported in cancer patients receiving recombinant GM-CSF therapies [89-93]. Serum samples collected before and after GM-CSF treatment from 214 patients with a variety of underlying diseases have been examined for the presence of antibodies. Neutralizing antibodies were detected in 5 of 214 (2.3%) after receiving GM-CSF by continuous IV infusion (3 patients) or subcutaneously (2 patients) for 28 to 84 days in multiple courses. All 5 patients had impaired hematopoiesis before the administration of GM-CSF and consequently the effect of the development of anti-GM-CSF antibodies on normal hematopoiesis could not be assessed. Neutralizing antibody responses to GM-CSF have been observed in some individuals and the presence of this response has been associated with decreased efficacy of GM-CSF administration. It is noteworthy,

however, that such responses are induced only after administration of high doses of recombinant GM-CSF over an extended period of time. It is of interest to note that follow-up of cancer patients in these studies has shown that anti-GM-CSF antibodies do not affect WBC levels. In addition, anti-GM-CSF antibody responses have been noted to disappear gradually after discontinuation of GM-CSF therapy [92].

Other studies have provided additional information relevant to the safety of anti-GM-CSF antibody responses. Investigators have recently observed that 94% to 100% of commercially available human immunoglobulin preparations contain anti-GM-CSF neutralizing antibody activity [94,95] and that plasma from 0.3% of normal healthy plasma donors possess neutralizing antibodies to GM-CSF [94]. Individuals with idiopathic pulmonary alveolar proteinosis (I-PAP), a rare lung condition, have been recently reported to possess significant levels of anti-GM-CSF antibodies [96]. Administration of GM-CSF to patients with I-PAP in one study was shown to reverse the condition in 43% of individuals [97]. It is clear, however, that many normal healthy individuals possess anti-GM-CSF neutralizing antibody titers and do not exhibit this clinical condition.

In summary, the administration of recombinant GM-CSF may result in the induction of binding and/or neutralizing antibodies in a subset of treated individuals. However, as anti-GM-CSF antibodies are found in healthy, untreated individuals, the clinical significance of these antibodies is unclear. In this study, the potential induction of binding and neutralizing anti-GM-CSF antibodies will be assessed.

2.5 Placebo

The placebo for all products in this trial will be sodium chloride injection USP, 0.9%.

3 Preclinical studies

3.1 Preclinical safety studies

3.1.1 Preclinical safety studies of *gag* DNA

Several toxicology studies conducted in macaques, white rabbits and mice suggest that *gag* DNA is well tolerated (Section 3.1.2).

During multiple dose administration of SIV *gag* plasmid DNA, with or without rhesus *IL-12* DNA, monitoring of hematologic parameters in macaques demonstrated values that were similar to those of controls and that remained within the normal range. In addition, studies in mice and rabbits given multiple doses of *gag* DNA, with or without *IL-12* DNA, showed no product-related clinical signs and no effect on body weight, food consumption, body temperature or clinical pathology parameters. The biodistribution of *gag* DNA was similar to that of the precursor plasmid APL 400-047, which has exhibited a favorable safety profile in a human trial [98]. According to these animal studies, *gag* DNA appears to be well tolerated and elicits immunogenic responses that may be enhanced by coadministration with an adjuvant.

3.1.2 Preclinical safety studies of *gag* DNA and *IL-12* DNA

Brief descriptions of the preclinical toxicology studies are provided in Table 3-1. Additional detail is provided in the Investigator's Brochure. Note that toxicology studies were conducted with plasmid *gag*-3339 which differs from plasmid *gag*-2962 by a single nucleotide difference in the origin of replication (noncoding). This nucleotide difference is associated with increased levels of plasmid production but does not effect levels of *gag* gene expression. Similarly, toxicology studies were conducted with plasmid *IL-12*-6285. Overall, *gag* DNA and *IL-12* DNA have been shown to be safe and well tolerated in mice, rabbits, and nonhuman primates.

Table 3-1 Summary of toxicology studies with human *IL-12* DNA

Study	Regimen	Methods/ Results
Biodistribution analysis in rabbits of *gag*-2962 combined with GENEVAX *IL-12*-6285<br><br>(NOTE: This study included another arm in which rabbits received GENEVAX *IL-15*-1696 co-administered with gag-2962. For these results see the GENEVAX *IL-15*-1696 IB, section 4.3.1).	30 rabbits (N=15/sex) were administered a mixture of GENEVAX *IL-12*-6285 with *gag*-2962 (1500 µg each) in a single bolus IM dose<br><br>Ten rabbits (N=5/sex) were sacrificed at each of three time-points: Days 3, 60, and 94.	Tissues collected on Days 3, 60, and 94 post-dosing. TaqMan® real-time PCR assays isolated genomic DNA from bone marrow, brain, heart, injection site skin, quadriceps muscle, kidney, liver, lung, mesenteric lymph node, gonads, spleen, and thymus.<br>Day 3 plasmid levels: high levels of *gag* and *IL-12* plasmids in injection site skin (*gag* plasmid mean: 6.0 x 10^6 copies /µg of genomic DNA, 10/10 animals; *IL-12* plasmid mean: 2.9 x 10^6 copies /µg of genomic DNA, 10/10 animals). Muscle also contained significant levels of plasmid (*gag* plasmid mean: 1.3 x 10^6 copies /µg of genomic DNA, 10/10 animals; *IL-12* plasmid mean: 1.5 x 10^5 copies /µg of genomic DNA, 10/10 animals). Plasmid levels in other tissues near or < assay LLOQ (100 copies/µg of genomic DNA) throughout study.<br>Day 60 plasmid levels: high levels of *gag* and *IL-12* plasmids at injection site skin of female rabbits (*gag* plasmid mean: 3.8 x 10^6 copies /µg of genomic DNA, 5/5 animals; *IL-12* plasmid mean: 1.4 x 10^6 copies /µg of genomic DNA, 5/5 animals). Decreased levels of *gag* and *IL-12* plasmids in injection site of male skin (*gag* plasmid mean: 2.2 x 10^4 copies /µg of genomic DNA, 5/5 animals; IL-12 plasmid mean: 7.8 x 10^3 copies /µg of genomic DNA, 5/5 animals). Muscle contained little or no plasmid (*gag* plasmid mean: 464 copies /µg of genomic DNA, 2/10 animals; *IL-12* plasmid 18 copies /µg of genomic DNA, 1/10 animals).<br>Day 94 plasmid levels: Reduced but significant levels of *gag* and *IL-12* plasmids at injection site skin of female rabbits (*gag* plasmid mean: 2.6 x 10^5 copies /µg of genomic DNA, 5/5 animals; *IL-12* plasmid mean: 1.1 x 10^5 copies /µg of genomic DNA, 5/5 animals). Muscle contained no or low levels of plasmid (*gag* plasmid mean: 3.4 x10^3 copies /µg of genomic DNA, 3/10 animals; *IL-12* plasmid 1.1 x 10^3 copies /µg of genomic DNA, 4/10 animals).<br><br>Integration analysis of Day 94 injection site skin (analysis performed by BioReliance). Host cell DNAs from injection site skin from 6 rabbits showing high *IL-12* and *gag* plasmid copy numbers on biodistribution study at Day 94 (*IL-12*/ *gag* copies/µg of genomic DNA: 239,217/ 643,736; 174,984/ 401,172; 537, 816/ 1,244,799; 14,481/ 33,083; 12,067/ 33,225; 1900/ 3,973) and from 2 control animal were extracted and purified by alternating TAE and TBE agarose gels (4 gel purifications). Q-PCR analyses on purified DNA were performed to detect possible presence of integrated *gag* or *IL-12* plasmid sequence. Q-PCR results indicated that the six high copy samples for *gag* and *IL-12* plasmids were less than the assay LLOQ (<100 copies/ plasmid), indicating no evidence of plasmid integration in these samples for either *gag* or *IL-12* sequence.

Study	Regimen	Methods/ Results
10-wk toxicity study with 4-wk recovery in Crl:CD-1® (ICR) BR mice<br>(N=60 per group, 30 M, 30 F)	1: *gag*-3339 + murine *IL-12* DNA (100µg IM per dose for each);<br>2: murine *IL-12* DNA alone (100µg IM);<br>3: Control vehicle (bupivacaine).<br>4 IM bolus injections, one dose on days 1, 22, 43, 64)	10 animals per sex per group: euthanized 14 days after 1st dose and 2 days after 4th dose. Remaining 10 in each group: 4-week compound/vehicle-free period.<br>No test article-related clinical signs; no test article-related effects on body weight, food consumption, clinical pathology parameters, organ weight, or macroscopic or microscopic changes. Injection site changes in controls and treated animals related to injection procedure or vehicle components.
10-wk toxicity study with 4-wk recovery in male (M) and female (F) New Zealand white rabbits<br>(N = 60; 20 per group, 10 M, 10 F)	1: GENEVAX *IL-12*-6285+ *gag*-3339 (1500µg per dose, each plasmid),<br>2: GENEVAX *IL-12*-6285 alone (1500µg per dose),<br>3: control vehicle (bupivacaine)<br>4 IM bolus injections, one dose on days 1, 22, 43, 64)	(5 per sex per group: euthanized:<br>2 days post final dose; 30 days post final dose to assess compound/ vehicle-free recovery)<br>No test article-related clinical signs; no effects on body weight, food consumption, body temperature; no test article-related clinical pathology abnormalities, no organ weight, macroscopic or microscopic changes; injection site irritation (lymphohistiocytic inflammation, slight to marked hemorrhage, muscle necrosis) in all groups related to injection procedure or vehicle components; inflammation and muscle necrosis resolved at recovery necropsy 4 weeks post final dose.
Hematologic safety study with different dose levels of SIV *gag* DNA and rhesus *IL-12* DNA in male rhesus macaques<br>N=25 (5 per group)	1: *gag*+*IL-12* DNAs, 1.5 mg each<br>2: *gag* alone, 1.5 mg<br>3: *gag*+*IL-12* DNAs, 5.0 mg each<br>4: *gag* DNA alone, 5.0 mg<br>5: unvaccinated controls<br>Dose administered IM at weeks 0, 4 and 8.	Analysis of white blood cells, hemoglobin, platelets, red blood cells, % lymphocytes demonstrated mean values similar to controls and within normal range.
Hematologic safety study with SIV *gag* DNA and rhesus *IL-12* DNA and/or rhesus *IL-15* DNA in rhesus macaques	1. *SIV gag* DNA alone, 1500 µg<br>2. SIV *gag* DNA + *IL-12* DNA, 1500 µg each<br>3. SIV *gag* DNA + *IL-15* DNA, 1500 µg each<br>4. SIV *gag* DNA + *IL-12* DNA + *IL-15* DNA, 1500 µg each<br>5. controls<br>N=25 (5 per group)<br>Dose administered IM at weeks 0, 4, 8, 29.	Analysis of white blood cells, hemoglobin, platelets, red blood cells, % lymphocytes demonstrated mean values similar to controls with no significant changes from baseline over the study course in all treatment groups.<br>No significant weight loss and no adverse events related to general appearance, appetite, injection site, or behavior were observed.<br>No detection of anti-IL-15 antibody in any group of macaques receiving DNA vaccine immunization

3.1.2.1 Toxicology study of murine *IL-12* DNA alone or in combination with HIV-1 *gag* DNA in mice

This study was designed to assess the toxicity of 4 intramuscular bolus injections of murine *IL-12* DNA alone (100 mcg), or combined with *gag* DNA (100 mcg), in mice. Administration was well tolerated. There were no product-related clinical signs and no product-related effects on body weight, food consumption, clinical pathology parameters, organ weight or macroscopic or microscopic changes. Injection site reactions were not different between the *IL-12* DNA, *gag* DNA plus *IL-12* DNA, and control (vehicle) injections.

3.1.2.2 Toxicology study of *IL-12* DNA alone or in combination with HIV-1 *gag* DNA in New Zealand white rabbits

The purpose of this study was to evaluate the toxicity of 4 intramuscular injections of *IL-12* DNA, 1500 mcg, administered alone or in combination with *gag* DNA, 1500 mcg, in New Zealand white rabbits. Administration of *IL-12* DNA alone or *gag* DNA/*IL-12* DNA was well tolerated. There were no product-related effects on body weight, food consumption or body temperature and no indication of injection site irritation at the recovery necropsy evaluation (day 30 post final dose). There were no product-related effects on clinical pathology parameters, and no organ weight or macroscopic or microscopic changes were noted at 30 days post final dose.

3.1.2.3 Biodistribution analysis of *IL-12* DNA and *gag* DNA in rabbits

Rabbits received a single intramuscular injection of 1500 mcg each of *IL-12* DNA and *gag* DNA. Tissues were collected on Days 2, 30, 60 and 94 to determine plasmid distribution. Tissues were sent to Exploratory Drug Safety in Andover, Massachusetts, USA, where a previously developed TaqMan® assay was used to detect and quantify amounts of *IL-12* plasmid and *gag* plasmid in the rabbit tissues.

On Day 3 post-dosing, high levels of *gag* and *IL-12* plasmids were observed in injection site skin (*gag* plasmid mean: 6.0×10^6 copies/mcg of genomic DNA, 10/10 animals; *IL-12* plasmid mean: 2.9×10^6 copies/mcg of genomic DNA, 10/10 animals). Muscle also contained significant levels of plasmid (*gag* plasmid mean: 1.3×10^6 copies/mcg of genomic DNA, 10/10 animals; *IL-12* plasmid mean: $1.5 \text{ x } 10^5$ copies/mcg of genomic DNA, 10/10 animals). Plasmid levels in other tissues near or less than the assay LLOQ (100 copies/mcg of genomic DNA) throughout study.

On Day 60 high levels of *gag* and *IL-12* plasmids were observed at the injection site skin of female rabbits only (*gag* plasmid mean: 3.8×10^6 copies/mcg of genomic DNA, 5/5 animals; *IL-12* plasmid mean: 1.4×10^6 copies/mcg of genomic DNA, 5/5 animals). In the injection site skin of male rabbits, decreased levels of *gag* and *IL-12* plasmids were observed (*gag* plasmid mean: 2.2×10^4 copies/mcg of genomic DNA, 5/5 animals; *IL-12* plasmid mean: $7.8 \text{ x } 10^3$ copies/mcg of genomic DNA, 5/5 animals). Muscle from males and females contained little or no plasmid (*gag* plasmid mean: 464 copies/mcg of genomic DNA, 2/10 animals; *IL-12* plasmid 18 copies/mcg of genomic DNA, 1/10 animals).

By Day 94 post dosing, reduced levels of *gag* and *IL-12* plasmids were observed at the injection site skin of female rabbits (*gag* plasmid mean: 2.6×10^5 copies/mcg of genomic DNA, 5/5 animals; *IL-12* plasmid mean: 1.1×10^5 copies/mcg of genomic DNA, 5/5 animals). In a few animals of both sexes, muscle contained no or low levels of plasmids (*gag* plasmid mean: 3.4×10^3 copies/mcg of genomic DNA, 3/10 animals; *IL-12* plasmid 1.1×10^3 copies/mcg of genomic DNA, 4/10 animals). Muscle of 7 animals contained no *gag* plasmid, while 6 animals had no *IL-12* plasmid in muscle. Results indicated that *gag* and *IL-12* plasmids did not persist at significant levels in the tissues examined, except for injection site skin.

3.1.2.4 Integration analysis of *IL-12* DNA and *gag* DNA in rabbits

Discussions were subsequently held with the FDA regarding possible concerns over plasmid integration in injection site skin samples possessing high copy number. The FDA recommended that Wyeth conduct an integration analysis of the high copy number injection site skin samples at Day 94 and further recommended specific tissue samples for analysis. Wyeth subsequently contracted with BioReliance to perform the integration analysis. The analysis evaluated host cell DNA extracted from Day 94 injection site skin obtained from six

rabbit showing high *IL-12/gag* plasmid copy numbers (239,217/ 643,736; 174,984/ 401,172; 537, 816/ 1,244,799; 14,481/ 33,083; 12,067/ 33,225; 1900/ 3,973 *IL-12/gag* plasmid copies/µg of genomic DNA) and from skin obtained from two negative control rabbits. Host cell DNAs were extracted and then purified by alternating TAE and TBE agarose gels by 4 gel purifications. Two independent Q-PCR analyses on purified DNA were then performed to detect possible presence of integrated *gag* or *IL-12* plasmid sequence. Result s of the analysis failed to produce evidence for integration of either *gag* or *IL-12* plasmid sequence as *gag* and *IL-12* Q-PCR values from the six high copy test samples were less than the assay LLOQ (<100 copies).

3.1.2.5 Safety of rhesus *IL-12* DNA plasmid in rhesus macaques

To date 20 rhesus macaques have been coimmunized with either 15 mg (three 5 mg doses at 0, 4 and 8 weeks) or 4.5 mg (three 1.5 mg doses at 0, 4 and 8 weeks) of rhesus *IL-12* DNA and no obvious adverse effects have been observed. IL-12 serum levels did not increase following plasmid administration and anti-IL-12 antibodies were not detected following vaccination. In addition, white blood cell count, hemoglobin, platelet count, red blood cell count and percent of lymphocytes were monitored biweekly for 12 weeks, and at Weeks 18 and 20 post inoculation. All evaluations revealed values within the normal range over the period studied.

3.1.3 Preclinical safety studies of CTL MEP vaccine

3.1.3.1 Toxicology study of HIV CTL MEP vaccine in New Zealand white rabbits

A study to evaluate the toxicity of a series of 4 intramuscular doses of the 4 CTL peptides contained in the HIV CTL MEP vaccine administered with RC529-SE adjuvant alone or in combination with GM-CSF was carried out in New Zealand white rabbits. There were no HIV CTL MEP vaccine–related issues with mortality, clinical signs, effects on body weight, food consumption, body temperature, injection site scoring, hematology and clinical chemistry parameters, organ weights or macroscopic observations, or microscopic observations that were considered adjuvant related. Slight to moderate injection site reactions were observed. Immunogenicity and efficacy were not tested. A more detailed description of this study is provided in the Investigator's Brochure. Based on these results, there was no observed adverse effect level of the 4 HIV CTL MEPs when administered to New Zealand white rabbits at a dose of 250 mcg/dose (1 mg total) in combination with RC529-SE (50 mcg/dose) and GM-CSF (250 mcg/dose).

3.1.3.2 HIV CTP MEP and SIV CTL MEP vaccine in nonhuman primates

Similar peptides have undergone evaluation for both safety and immunogenicity in nonhuman primates. In addition, HIV CTL MEP and *gag* + *IL-12* DNA have been tested in cynomolgus monkeys in an exploratory study of the suitability of the model to evaluate the human CTL epitopes contained in the vaccine (See Section 3.2.2.2 and 3.2.2.3 for a description of these results).

3.2 Preclinical immunogenicity studies

3.2.1 Preclinical immunogenicity studies of SIV *gag* DNA alone and with rhesus *IL-12* plasmid DNA in rhesus macaques

Four groups of 5 rhesus macaques were vaccinated intramuscularly (0, 4, 8 weeks) with 1.5 or 5 mg of SIV *gag* DNA, either with or without rhesus *IL-12* DNA at 1.5 and 5 mg, respectively. Robust (>1:1000) serum antibody responses to SIV Gag were observed in 8/10

animals receiving *IL-12* DNA (high 5 mg or low 1.5 mg dose) compared to 3/10 animals in the groups that did not receive *IL-12* DNA. IFN-γ ELISpot assay to overlapping SIV *gag* peptide pools was also performed to measure cellular immune responses. Moderate numbers of IFN-γ expressing cells (mean 300 SFU/million cells) were detected in macaques vaccinated with 1.5 or 5 mg SIV *gag* DNA alone by Week 10, compared to high numbers of IFN-γ expressing cells (mean 1400 SFU/million cells) in groups receiving *IL-12* DNA (see Table 3-2). IFN-γ ELISpot responses were similar with either dose level of *IL-12* DNA.

Table 3-2 IFN-γ ELISpot responses (per 10^6 cells) in monkeys immunized with SIV *gag* DNA and rhesus *IL-12* DNA

	Week					
Vaccine	2	4	6	8	10	20
1.5 mg SIV *gag* DNA + 1.5 mg IL-12 DNA	41	65	768	718	2098	928
	158	40	405	1200	425	89
	135	9	690	193	2948	948
	62	23	1436	450	1115	249
	69	9	500	0	135	456
Mean	93	29	760	292	1344	534
1.5 mg SIV *gag* DNA	11	23	228	160	278	50
	13	0	119	128	240	251
	25	3	10	0	28	19
	9	0	110	63	321	101
	79	36	25	15	411	178
Mean	27	12	98	73	256	120
5 mg SIV *gag* DNA + 5 mg IL-12 DNA	129	404	1190	473	1796	978
	1	118	534	150	538	159
	65	168	966	333	1373	499
	0	348	640	83	725	768
	196	289	1175	218	2733	431
Mean	78	265	901	251	1433	567
5 mg SIV *gag* DNA	10	24	43	0	392	71
	101	54	36	43	255	32
	115	495	421	110	726	281
	15	5	336	305	271	207
	25	236	233	0	48	142
Mean	53	163	214	92	338	147
Controls	36	0	0	15	68	0
	45	33	0	0	85	29
	113	96	0	88	13	5
	88	9	0	8	0	19
	46	0	0	0	0	4
Mean	66	28	0	22	33	11

The humoral and cellular immune responses elicited by a RNA optimized SIV *gag* DNA construct were substantially enhanced by coimmunization with rhesus *IL-12* expressing plasmid DNA

The above animals subsequently received intravenous SHIV 89.6P challenge (30 MID50). Preliminary results following challenge have indicated that animals receiving *IL-12* and *gag* plasmids demonstrated decreased levels of peak viremia, lower viral setpoints, and slower rate of CD4+ T cell decline (data not shown).

3.2.2 Preclinical immunogenicity studies of HIV CTL MEP vaccine

Several nonclinical studies were carried out to evaluate the immunogenic properties of the HIV CTL MEP vaccine. These included evaluation of antigen processing of the CTL epitopes in the vaccine peptides, nonhuman primate studies that evaluated the adjuvant properties of vaccine adjuvants RC529-SE and GM-CSF, and an immunogenicity study of HIV CTL MEP in transgenic mice. In addition, HIV CTL MEP and *gag* + *IL-12* DNA have been tested in cynomolgus monkeys in an exploratory study of the suitability of the model to evaluate the human CTL epitopes contained in the vaccine.

3.2.2.1 Immunogenicity of HIV CTL MEP in HLA-A*0201 transgenic mice

This study was undertaken to formally demonstrate that CTL epitopes contained within the HIV CTL MEP vaccine can be properly processed and presented to the immune system for CD8+ CTL induction. A mouse strain derived from C57BL/6 mice that expresses the human HLA-A*0201 Class I gene was used in order to allow the evaluation of immune responses characteristic of human, rather than murine, T cells. Mice were injected with a vaccine formulation consistent with the formulation proposed for human clinical evaluation in this trial. Using MHC Class I tetramer staining and IFN-γ ELISpot assays, it was demonstrated that a subset of mice generated an HLA-A*0201-restricted cellular immune response to an A*0201-restricted epitope within the CTL MEP by tetramer analysis. This study indicates that the CTL epitopes contained within the CTL MEP are effectively presented to the immune system for the induction of cytotoxic T cells in this transgenic mouse model.

3.2.2.2 Nonhuman primate studies with related peptide vaccines

HIV C4-V3 peptide vaccine administered in RC529-SE in combination with GM-CSF adjuvant in cynomolgus macaques

The safety and immunogenicity of an HIV peptide vaccine administered in RC529-SE combined with GM-CSF adjuvant was evaluated in cynomolgus macaques. The hybrid peptide used in this study consisted of the HIV-1 Env-derived peptide, C4(E9V)-$V3_{89.6P}$. The GM-CSF adjuvant employed in this primate study was recombinant human GM-CSF produced in yeast (Leukine®, 250 mcg) and was coadministered with RC529-SE (50 mcg). A group of 4 cynomolgus macaques was immunized 5 times (weeks 0, 4, 8, 18 and 23) by the intramuscular route with peptide (1000 mcg) plus RC529-SE combined with GM-CSF. The control group consisted of 4 animals that received peptide alone administered by the intranasal route. Serum samples from all animals were obtained immediately prior to and at 1 and 2 weeks after each vaccination.

The C4(E9V)-$V3_{89.6P}$ peptide was well tolerated when coadministered with RC529-SE plus GM-CSF by intramuscular injection. No adverse injection site reactivities or increases in body temperature readings were noted. Peptide administered alone was similarly well tolerated.

This study demonstrated that a peptide immunogen coadministered with RC529-SE combined with GM-CSF adjuvant can be safely administered multiple times. In addition, RC529-SE/GM-CSF adjuvant was shown to enhance antibody responses relative to a control both in serum and in several mucosal secretions (nasal, cervicovaginal, and rectal). Thus, both the safety and immunogenicity of hybrid peptide in combination with RC529-SE/GM-CSF were demonstrated in this model.

SIV peptide vaccine administered in IFA, or in RC529-SE with GM-CSF adjuvant in cynomolgus macaques

This study was designed to compare the safety and immunogenicity of peptide vaccine adjuvanted with IFA to the vaccine formulated with RC529-SE/GM-CSF (Table 3-3). The peptide examined in this study was an SIV *env*-derived T-helper / SIV *gag* CTL peptide conjugate (ST1-p11C,12-mer; RQIINTWHKVGKNVYL-EGCTPYDINQML). A total of 4 macaques were used for the study, all of which were Mamu-A01+.

Group 1 animals received 0.5 mL of the Th-CTL peptide ST1-p11C, 12-mer (1.0 mg/mL) combined with 0.5 mL IFA in a total volume of 1.0 mL. The group 2 animals received 0.5 mL of ST1-p11C, 12-mer (1 mg/mL) combined with 250 mcg of human GM-CSF, 50 mcg of RC529-SE (final oil concentration of 1%) in a total volume of 1 mL. All macaques were immunized by intramuscular injection on a schedule of 0, 4, and 8 weeks.

Table 3-3 Study design of rhesus macaque study

Group #	# Animals	Animal ID	Vaccine	Adjuvant	Route	# Doses
1	2 Mamu-A01+	95x009<br>93x021	ST1-p11C,12mer	IFA	IM	3
2	2 Mamu-A01+	98n002<br>98n008	ST1-p11C,12mer	RC529-SE/GM-CSF	IM	3

The ST1-p11C + IFA formulation was associated with significant injection site reactivity. One animal (93x021) developed a 1.5 cm sized abscess at the site of injection 2 weeks after the second immunization. The other animal (95x009) also developed a 2 cm sized abscess at the site of injection 2 weeks after the third immunization. This abscess broke through the skin and required dressing. In contrast, ST1-p11C + RC529-SE combined with GM-CSF was not associated with injection site reactions or systemic symptoms. Administration of the ST1-p11C + RC529-SE/GM-CSF formulation was associated with vomiting in 2 animals possibly related to administration of anesthetic to the animals. While the anesthetic (ketamine) is known to be associated with vomiting, no other cases of vomiting were noted over the course of the study.

Immunogenicity results for the 4 animals in this study are provided in Table 3-4.

Table 3-4 Percent p11C-tetramer staining and functional p11C-specific CTL responses after 14-day *in vitro* peptide p11C stimulation

		Timepoint (relative to immunizations); assay									
		Week 0 (Pre)		Week 5 (1 wk post 2d)		Week 6 (2 wk post 2d)		Week 9 (1 wk post 3d)		Week 10 (2 wk post 3d)	
Adjuvant	Animal	TS	CTL	TS	CTL	TS	CTL	TS	CTL	TS	CTL
IFA	95x009	0.03	0.6	0.23	nd	0.27	0.0	0.76	3.0	0.72	0.0
	93x021	0.03	0.0	34.31	66.3	15.15	4.69	4.90	7.3	nd	nd
RC529-SE/ GM-CSF	98n002	0.21	0.0	nd	nd	0.73	nd	0.07	5.7	0.39	0.0
	98n008	0.16	0.0	nd	nd	5.84	nd	2.24	11.4	5.00	0.0

Immunization with vaccine ST1-p11C + adjuvant as listed.

TS: Tetramer staining. Reported as the percentage of CD3+CD8+ cultured cells staining positive with the p11C-tetramer. Tetramer responses ≥1% were considered positive.

CTL: Standard chromium release CTL assay. Reported as the percent p11C-specific lysis (minus background) at an effector to target ratio (E:T) of 20:1. Peptide-specific lysis of ≥10% was considered positive.

nd: Not determined.

No data available for Week 8 (4 weeks post second immunization).

In summary of this section, the ST1-p11C peptide vaccine adjuvanted with RC529-SE/GM-CSF was well tolerated in the 2 animals tested. In contrast, significant injection site reactivity was noted in the 2 animals immunized with vaccine adjuvanted with IFA. The severe local reactions observed in this study were similar to local reactions observed in individuals immunized with peptide/IFA in the AVEG 020 study in humans. ST1-p11C peptide vaccine administered with IFA was capable of inducing a potent p11C-specific cellular immune response in 1 of 2 macaques tested. ST1-p11C peptide vaccine administered with RC529-SE combined with GM-CSF was also capable of inducing a p11C-specific cellular immune response in 1 of 2 macaques tested, but required *in vitro* amplification before the response was detected. These data indicate that peptide administered with RC529-SE/GM-CSF has an improved tolerability profile compared with the peptide/IFA combination and is capable of inducing CD8+ cellular immune responses.

3.2.2.3 Study of HIV CTL MEP and HIV DNA vaccines in cynomolgus macaques

An exploratory study was conducted to investigate immune responses in cynomolgus monkeys immunized with the Wyeth human clinical vaccines consisting of the HIV CTL MEP and the HIV DNA vaccine (*gag*-2962 + rhesus *IL-12* DNA). This represents the first evaluation of HIV CTL MEP in non-human primates. The study was designed to evaluate cellular immune responses to each vaccine following a 3-dose priming sequence and following a 3-dose booster immunization with heterologous vaccine. An arm of the study evaluated simultaneous administration of CTL MEP and HIV DNA vaccine to optimally assess immune recognition of the vaccines in this new model. The primary objective of the study was to determine whether the cynomolgus monkey represented a useful model for evaluating the Wyeth HIV vaccines based on human HIV CTL epitopes. A secondary objective was to investigate the two heterologous prime-boost regimens.

Also, as this study represents the first study in which non human primates received both HIV CTL MEP and HIV DNA vaccines, it provides an initial safety assessment of animals immunized with both vaccines. The study design is presented in Table 3-5.

Table 3-5 Study design: evaluation of HIV CTL MEP and HIV DNA vaccine in cynomolgus macaque

Group	Priming vaccine (weeks 0, 4, 8)	Booster vaccine (weeks 20, 24, 28)	Delivery
1 (n=4)	HIV CTL MEP (1 mg)<br>+ RC-529 (50 mcg)<br>+ GM-CSF (250 mcg)	*gag*-2962 + rhesus *IL-12* DNA (1.5 mg each)	IM
2 (n=4)	*gag*-2962<br>+ rhesus *IL-12* DNA<br>(1.5 mg each)	HIV CTL MEP (1 mg)<br>+ RC-529 (50 mcg)<br>+ GM-CSF (250 mcg)	IM
3 (n=6)	*gag*-2962+ rhesus *IL-12* DN A (1.5 mg each)<br>+ HIV CTL MEP (1 mg)<br>+ RC-529 (50 mcg)<br>+ GM-CSF (250 mcg)	*gag*-2962 + rhesus *IL-12* DNA (1.5 mg each)<br>+ HIV CTL MEP (1 mg)<br>+ RC-529 (50 mcg)<br>+ GM-CSF (250 mcg)	IM
4 (n=4)	unimmunized	unimmunized	unimmunized

ELISpot responses are shown in Figures 3-1 to 3-3. The results obtained following the priming immunizations (weeks 0, 4, 8) show that the plasmid alone group (Group 2, Figure 3-1) and plasmid plus peptide group (Group 3, Figure 3-3) exhibited significant ELISpot

activity. However, the peptide alone group (Group 1, Figure 3-2) showed either no response or minimal response two weeks following the third immunization. This finding indicates that the human HIV CTL epitopes contained in HIV CTL MEP are poorly recognized by cynomolgus monkeys when presented as peptide immunogens.

Although monkeys administered HIV CTL MEP in a priming series initially exhibited minimal cellular immune response, when these animals were then administered HIV DNA vaccine in booster series, they showed substantial responses by two weeks following the second and third booster (eg, week 30: 1771 SFU/million PBMC) (Figure 3-2). These responses were substantially higher than responses observed in Group 2 monkeys two weeks after receiving the third priming dose (e.g., week 10: average peak response of 683 SFU/million, Figure 3-1). Even though cynomolgus monkeys represent a weak model for HIV CTL MEP vaccine studies, the model provides scientific rationale supporting evaluation of peptide prime/DNA boost vaccine immunization regimen in humans. The observation that weak priming responses by peptide in this model enhance subsequent immunizations with plasmid vaccine may be an indication that peptide priming in humans may result in more pronounced booster responses to plasmid vaccine than observed in this study.

Figure 3-1 Total IFN-γ response to vaccine antigen in macaques primed with plasmid, and boosted with peptide (Group 2)

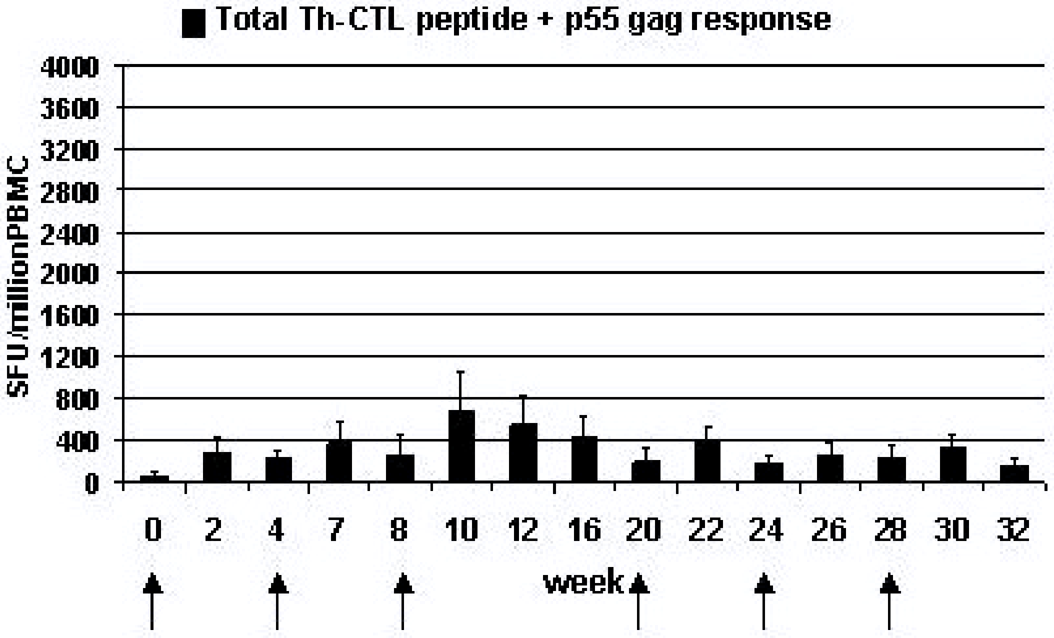

Figure 3-2 Total IFN-γ response to vaccine antigen in macaques primed with peptide and boosted with plasmid (Group 1)

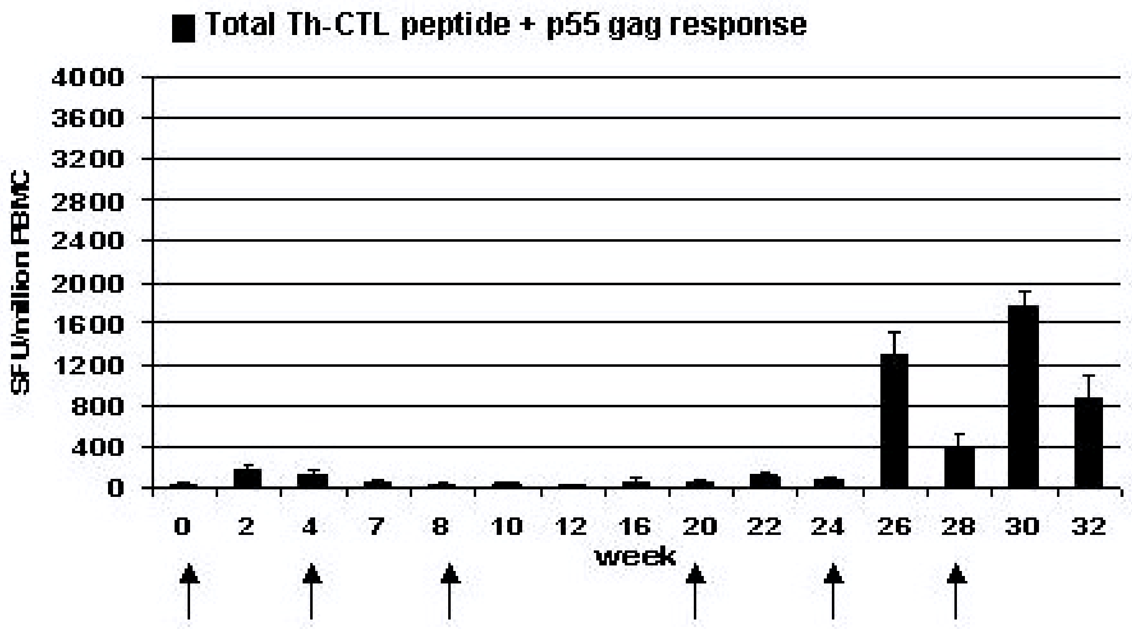

Figure 3-3 Total IFN-γ response to vaccine antigen from animals co-administered peptide plus plasmid for prime and boost immunizations (Group 3)

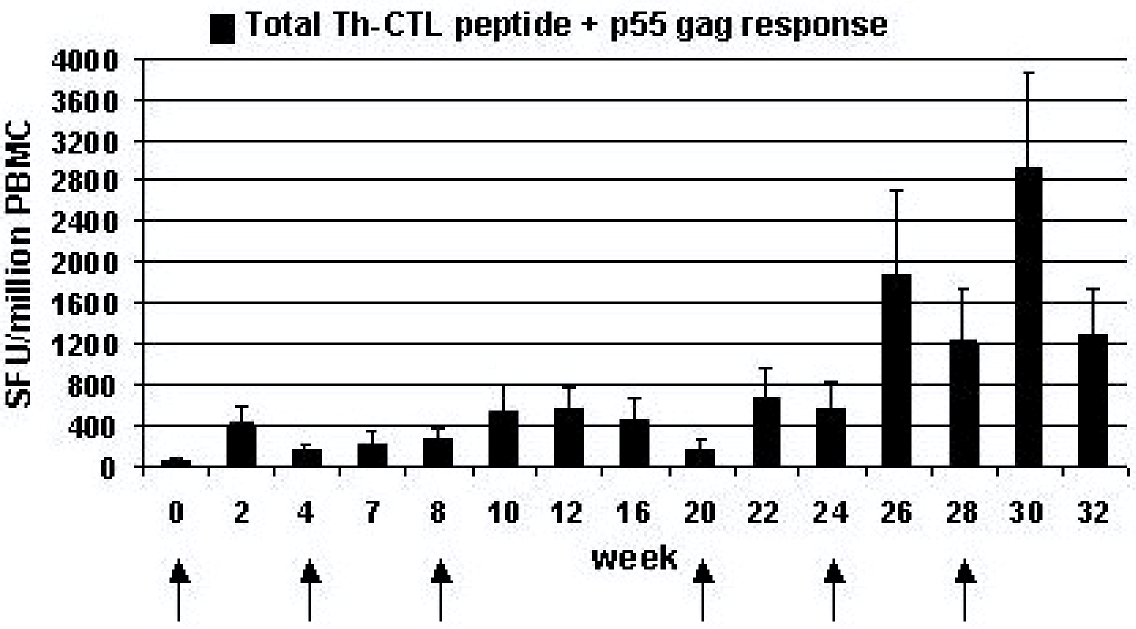

Figure 3-1 demonstrates that Group 2 animals exhibited minimal cellular responses following the HIV CTL MEP booster immunizations. These data are consistent with the poor peptide recognition exhibited by Group 1 animals following peptide priming (Figure 3-2). The results do not necessarily predict responses in humans immunized by an analogous vaccination regimen. The potential effectiveness of this vaccination strategy in humans will need to be directly assessed in clinical testing.

Co-immunization with the peptide plus plasmid (Group 3) resulted in elevated cellular responses relative to responses seen with Group 1 and Group 2 monkeys (Figure 3-3). The combined peptide plus plasmid dosage form may potentially be a more widely acceptable and practical vaccination regimen.

Importantly, the two heterologous prime/boost regimens, together with the regimen examining combined administration of plasmid plus peptide over six immunizations, did not result in serious local or systemic reactions or evidence of any adverse events in immunized monkeys.

4 Clinical studies

4.1 HIV-1 *gag* DNA vaccine

The HIV-1 *gag* DNA vaccine (GENEVAX® *gag*-2962) has not yet been evaluated in humans.

4.2 *IL-12* DNA adjuvant

The *IL-12* DNA vaccine adjuvants (GENEVAX® *IL-12*-6285 and GENEVAX® *IL-12*-4532) have not yet been evaluated in humans.

4.3 HIV CTL MEP / RC529-SE vaccine

Clinical evaluation of the CTL MEP vaccine with and without GM-CSF has been initiated in HVTN 056 in April 2004. As of February 7, 2005, Part A of the study is fully enrolled, and the 24 participants have all completed their injection series. An additional 28 participants have received at least one injection in Part B. Most participants have reported mild or moderate injection site pain or tenderness following vaccination (Table 4-1). Two participants have reported severe pain following their first vaccinations. In one case the pain was mild by day 2 and resolved by day 3. In the other case, the pain was associated with severe tenderness, and symptoms were mild by day 3. Two participants reported severe fatigue/malaise following their first vaccinations, which resolved within 24 hours in one case, and in another case improved to mild by day 3. These Grade 3 events have been reported to DAIDS according to the reporting requirements in the *DAIDS Serious Adverse Experience Reporting Manual for HVTN, November 15, 2000*. No SAEs have occurred (as defined by ICH Guidelines or 21 CFR 312.32). One other participant reported moderate sternal chest pain, possibly related to vaccine, which resolved overnight with ibuprofen and has not recurred. With subsequent doses, reactogenicity overall does not appear to be increasing in intensity or frequency, and may be diminishing. Study assignments remain blinded. All available safety data from HVTN 056 will be evaluated prior to the initiation of the booster vaccination series in Part B of HVTN 060 using the CTL MEP vaccine.

Table 4-1 HVTN 056: Adverse events at least possibly related to investigational product, all injections grouped (blinded data), N=52

Adverse event	Mild	Moderate	Severe	Total
Pain and/or tenderness (injection site)	17	28	2	47
Erythema and/or induration (injection site)	13	0	0	13
Fatigue/malaise	11	14	2	27
Myalgia	14	5	0	19
Headache	18	3	0	21
Nausea	10	1	0	11
Chills	7	2	0	9
Arthralgia	10	2	0	12
Temperature	5	0	0	5
Dizziness	4	0	0	4
Pharyngitis	1	0	0	1
Nasal congestion	1	0	0	1
Chest pain	0	1	0	1
Muscle tightness	1	0	0	1
Injection site swelling	2	0	0	2
Injection site hemorrhage (bruise)	1	0	0	1
Hyperbilirubinemia	1	0	0	1
Maculopapular rash	1	0	0	1
Erythematous lesions	1	0	0	1

4.4 Clinical study of an HIV peptide vaccine: AVEG 020

Although HIV CTL MEP / RC529-SE has not yet been evaluated in humans, a related HIV peptide vaccine formulated with IFA was evaluated in low-risk volunteers in the AIDS Vaccine Evaluation Group clinical trial AVEG 020 [98]. This vaccine consisted of a mixture of hybrid C4-V3 peptides representing 4 different Clade B HIV strains, each of which contained a unique CTL epitope restricted by the HLA-B7 allele. Early in the trial, unacceptable levels of reactogenicity were observed. Vaccination site reactions, including formation of sterile abscesses, were noted in a few volunteers after 1 or 2 doses of peptide + IFA and included sterile abscess formation. Flu-like symptoms were noted also in IFA-peptide recipients in this trial. In response to these observations the trial was closed to accrual and no further vaccinations were given.

Detectable cellular immune responses were detected in a subset of participants after 1 or 2 vaccinations. Two individuals possessing the HLA-B7 phenotype had a detectable HIV-specific CD8+ CTL response. Six of the HLA-B7+ participants received 2 doses, 3 each from the low and high dose groups. Positive responses were observed in one individual from each of the two dose levels. In summary, the data from AVEG 020 demonstrated that IFA was not a suitable adjuvant for this peptide-based HIV vaccine. This observation was confirmed by the findings of Toledo *et al.* in a separate human clinical trial using a similar adjuvant [99]. However, AVEG 020 also provided proof-of-concept data to justify further clinical development of HIV CTL MEP vaccines.

5 Summary

5.1 Rationale for trial design

Wyeth is pursuing a mixed modality CTL vaccine approach that consists of peptide and DNA technology platforms. These platforms were selected based on their complementing strengths. The HIV CTL MEP vaccine component may be most effective at inducing a balanced immune response to dominant and subdominant CTL epitopes (breadth) but may lack the ability to generate robust CTL responses. On the other hand, the SIV *gag* + rhesus *IL-12* DNA vaccine regimen has been shown in primates to induce robust CTL responses (ELISpot), but plasmid vaccines currently lack the ability to generate broad CTL responses.

It is anticipated that a combination prime/boost approach will build on the strengths of each technology and provide the desired breadth and robustness of cellular immune responses that are required to confer protective immunity. However, the optimal vaccination regimen for inducing broad and robust CTL responses is not known. A key objective in the early clinical development program is to determine which prime/boost vaccination regimen (peptide/plasmid or plasmid/peptide) will generate a CTL response possessing optimal breadth, depth, and magnitude.

As this is the first trial to investigate plasmid *IL-12*, the trial begins with a cautious dose escalation of *IL-12* DNA in Part A. There is a *gag* alone arm to judge whether there are safety issues with this particular *gag* plasmid. The 10:2 randomization ratio is standard for Part A of HVTN Phase I studies, an approach developed by SCHARP for the HVTN. This design is powered to observe safety and toxicity outcomes if they are likely (prevalence ≥20%). Two placebo recipients are included in each group to maintain blinding and the combined placebo groups will provide limited safety information.

Part B represents a regimen optimization/selection design. Each group consists of 30 vaccinees and 6 placebo recipients, who are included in each group to maintain blinding. The pooled placebo groups (n = 18) will provide safety information and will be used to calculate false positive rates for immunogenicity assays. Although samples for immunogenicity assays will be collected during Part A, these samples will only be analyzed if immune responses are detected during Part B of the trial. Statistical properties for safety and immunogenicity analyses are provided in Section 9.

5.2 HVTN 056 and timing of initiation of HVTN 060

HVTN 056, entitled "A Phase I clinical trial to evaluate the safety and immunogenicity of a CTL multi-epitope peptide HIV vaccine formulated with RC529-SE, with or without GM-CSF, in healthy, HIV-1 uninfected adult participants," initiated enrollment in April 2004. HVTN 060 is anticipated to begin enrollment in Q2, 2005. All available safety data from Part A of HVTN 056 will be evaluated prior to administration of HIV CTL MEP / RC529-SE boosting in HVTN 060. HVTN 061 is designed to follow HVTN 060, and will evaluate booster doses of HIV CTL MEP / RC529-SE or *gag* DNA + *IL-12* DNA given to recipients of HIV CTL MEP / RC529-SE from HVTN 056. Thus, these 3 HVTN protocols (056, 060, and 061) are closely interrelated. The temporal relationship of the present protocol to HVTN 056 and HVTN 061 is outlined in Figure 1-1.

5.3 Plans for future product development and testing

The current HIV-1 *gag* DNA vaccine represents a prototype plasmid vaccine and as such, contains only the p37 portion of the Gag molecule. The next generation plasmid vaccine will contain the full *gag* gene sequence as well as several other HIV genes. With regard to the HIV CTL MEP program, Wyeth is pursuing development of additional peptide components representing additional CTL "hot spots" located in a broader selection of HIV proteins. It is anticipated that the final vaccine regimen will combine the use of additional HIV genes in the DNA component with additional MEP peptides.

STUDY DESIGN

6 Study objectives

6.1 Primary objectives

Part A

- To evaluate the safety and tolerability of intramuscular administration of HIV-1 *gag* DNA vaccine
- To evaluate the safety and tolerability of intramuscular administration of HIV-1 gag DNA vaccine plus IL-12 DNA adjuvant (at escalating doses of 100 mcg, 500 mcg, and 1500 mcg)

Part B

- To further evaluate the safety and tolerability of intramuscular administration of HIV-1 *gag* DNA vaccine following a priming series and booster vaccinations with homologous plasmid
- To further evaluate the safety and tolerability of intramuscular administration of HIV-1 *gag* DNA vaccine plus *IL-12* DNA adjuvant as a priming series followed by booster vaccinations with homologous plasmids or HIV CTL MEP/RC529-SE/GM-CSF

6.2 Secondary objectives

Parts A and B

- To evaluate the immunogenicity of intramuscular administration of HIV-1 *gag* DNA vaccine. HIV-specific responses will be assessed using IFN-γ ELISpot and intracellular cytokine staining.
- To evaluate the immunogenicity of intramuscular administration of HIV-1 *gag* DNA vaccine plus *IL-12* DNA adjuvant as a priming series followed by booster vaccinations with homologous plasmids or HIV CTL MEP/RC529-SE/GM-CSF. HIV-specific responses will be assessed using IFN-γ ELISpot and intracellular cytokine staining.

Social impacts

- To evaluate the social impacts of trial participation

7 Study type, study population, and eligibility criteria

The study is a Phase I multicenter, randomized, placebo-controlled, double-blind trial to evaluate the safety and immunogenicity of HIV-1 *gag* DNA vaccine alone and in combination with *IL-12* DNA adjuvant as a 3 dose priming series. Due to differences in injection volumes, study participants and study staff cannot be blinded to study group assignment. The study is double-blinded regarding vaccine study product versus placebo administration. Participants in Part B will also receive 2 booster vaccinations either with the above plasmids or with HIV CTL MEP vaccine or with placebo. Participants will be recruited and screened. Those determined to be eligible, based on the inclusion and exclusion criteria (see Table 7-1 and Table 7-2), will be enrolled in the study and followed for a total period of 9 months in Part A and 15 months in Part B.

Participants will receive priming series vaccinations at Days 0, 28, and 84 (Months 0, 1 and 3). Booster vaccinations for participants in Part B will be administered at Days 168 and 273 (Months 6 and 9). All vaccinations will be administered by intramuscular injection in the deltoid using needle/syringe in the outpatient setting.

Participants will be healthy HIV-1-uninfected (seronegative) adults who comprehend the purpose of the study and have provided written informed consent.

See Table 7-1 for inclusion criteria and Table 7-2 for exclusion criteria. Final eligibility determination will depend on results of laboratory tests, medical history, physical examinations, and answers to the self-administered and/or interview questions.

See Table 12-1 for screening procedures.

Table 7-1 Study inclusion criteria

Note: Investigators should always use good clinical judgment in considering a volunteer's overall fitness for trial participation. Some volunteers might not be appropriate for enrollment even if they meet all inclusion /exclusion criteria because medical, psychiatric, or social conditions might make evaluation of safety and / or immunogenicity difficult.

General

Age: 18 to 50 years

Access to a participating HVTU and willingness to be followed for the planned duration of the study

Assessment of understanding: Complete a questionnaire prior to first vaccination; verbalize understanding of all questions answered incorrectly

Willingness to receive HIV test results

Informed consent: Be able and willing to provide informed consent

Health: Be in good general health as shown by medical history, physical exam, and screening laboratory tests performed within 56 days of enrollment.

Laboratory

Hemoglobin ≥ sex-specific institutional lower limit of normal and at least 11.0 g/dL for women, 13.0 g/dL for men

WBC count = 3,300 to 12,000 cells/mm^3

Total lymphocyte count ≥ 800 cells/mm^3

Remaining differential either within institutional normal range or accompanied by site physician approval

Platelets = 125,000 to 550,000/mm^3

Chemistry panel

Part A: ALT, AST, alkaline phosphatase, and creatinine values do not exceed institutional upper limit of normal, and CPK value does not exceed 2 times the institutional upper limit of normal;

Part B: ALT, AST, and alkaline phosphatase do not exceed 1.25 times the institutional upper limit of normal; creatinine does not exceed institutional upper limit of normal, and CPK value does not exceed 2 times the institutional upper limit of normal.

Negative HIV blood test. US participants must have a negative FDA-approved ELISA test. Non-US sites will use locally available and locally approved assays.

Negative Hepatitis B surface antigen (HBsAg)

Negative anti–Hepatitis C virus antibodies (anti-HCV), or negative HCV PCR if the anti-HCV is positive

Normal urine:

- Negative urine glucose, and
- Negative or trace urine protein, and
- Negative or trace urine hemoglobin (if trace hemoglobin is present on dipstick, a microscopic urinalysis is required to exclude participants with counts greater than the institutional normal range)

Additional inclusion criteria for female participants

Negative serum or urine β-HCG pregnancy test performed on the day of initial vaccination prior to vaccination

Reproductive status: a female participant must:

- agree to consistently use contraception for at least 21 days prior to enrollment until the last protocol visit, for sexual activity that could lead to pregnancy. Contraception is defined as using any of the following methods:
  - condoms (male or female) with or without a spermicide
  - diaphragm or cervical cap with spermicide
  - intrauterine device (IUD)
  - hormonal contraception
  - successful vasectomy in the male partner (considered successful if a woman reports that a male partner has [1] microscopic documentation of azoospermia, or [2] a vasectomy more than 2 years ago with no resultant pregnancy despite sexual activity post-vasectomy)
- or not be of reproductive potential, such as having reached menopause (no menses for one year) or having undergone hysterectomy, bilateral oophorectomy, or tubal ligation.
- agree not to seek pregnancy through alternative methods such as artificial insemination or in vitro fertilization until last protocol visit.

Table 7-2 Study exclusion criteria

Participant has received any of the following substances:

HIV vaccine(s) in a prior HIV vaccine trial. *For potential participants who have received control/placebo in an HIV vaccine trial, documentation of the identity of the study control/placebo must be provided to the Protocol Safety Team, who will determine eligibility on a case-by-case basis.*

Immunosuppressive medications within 168 days before first vaccination, e.g., oral/parenteral corticosteroids, and/or cytotoxic medications. *Not excluded: A participant using any of the following is not excluded: (1)corticosteroid nasal spray for allergic rhinitis; (2) topical corticosteroids for mild, uncomplicated dermatitis*

Blood products within 120 days before first vaccination

Immunoglobulin within 60 days before first vaccination

Live attenuated vaccines within 30 days before first vaccination

Investigational research agents within 30 days before first vaccination

Medically indicated subunit or killed vaccines, e.g., influenza within 14 days, pneumococcal within 14 days, or allergy treatment with antigen injections within 30 days prior to initial study vaccine administration

Current anti-TB prophylaxis or therapy

Participant has a clinically significant medical condition, physical examination findings, clinically significant abnormal laboratory results, or past medical history with clinically significant implications for current health. A clinically significant condition or process includes but is not limited to:

- a process that would affect the immune response
- a process that would require medication that affects the immune response
- any contraindication to repeated injections or blood draws
- a condition that requires active medical intervention or monitoring to avert grave danger to the participant's health or well-being during the study period
- a condition or process in which signs or symptoms could be confused with reactions to vaccine
- any condition specifically listed among the exclusion criteria below

Any medical, psychiatric, or social condition, or occupational or other responsibility that, in the judgment of the investigator, would interfere with, or serve as a contraindication to, protocol adherence, assessment of safety or reactogenicity, or a participant's ability to give informed consent

Diagnosed allergies to egg products (Part B only)

Diagnosed allergies to yeast-derived products (Part B only)

Diagnosed allergies to amide-type local anesthetics [(bupivacaine (Marcaine), lidocaine (Xylocaine), mepivacaine (Polocaine/Carbocaine), etidocaine (Duranest), prilocaine (Citanest, EMLA cream)].

Serious adverse reactions to vaccines including anaphylaxis and related symptoms such as hives, respiratory difficulty, angioedema, and/or abdominal pain. *Not excluded: A participant who had a nonanaphylactic adverse reaction to pertussis vaccine as a child.*

Autoimmune disease

Immunodeficiency

Active syphilis infection. *Not excluded: Syphilis fully treated over 6 months ago.*

Asthma that is greater than mild in severity. Specifically excluded are participants with any of the following within the past year:

- daily symptoms
- daily use of short acting $beta_2$ agonists
- frequent exacerbations of symptoms that interfere with daily activity
- use of moderate to high dose inhaled corticosteroids (e.g., more than the equivalent of 250 mcg fluticasone, 400 mcg budesonide, 500 mcg beclomethasone, or 1000 mcg triamcinolone/flunisolide, as a daily dose) or theophylline
- emergent care, urgent care, hospitalization or intubation for asthma

Diabetes mellitus type I or type II, including cases controlled with diet alone. *Not excluded: history of isolated gestational diabetes.*

Thyroid disease or thyroidectomy requiring medication during the last 12 months.

Angioedema within the last 3 years if episodes are considered serious or have required medication within the last 2 years.

Hypertension that is not well controlled by medication, or BP≥ 150/100 (either or both values) at enrollment.

BMI ≥ 40; or, BMI ≥ 35 with more than one of the following: age >45, BPs ≥ 140/90, smoking known hyperlipidemia. A person with BMI over 35 should be asked about other contributory health risks.

Bleeding disorder diagnosed by a doctor, e.g., factor deficiency, coagulopathy, or platelet disorder requiring special precautions

Malignancy *Not excluded: A participant with a surgical excision and subsequent observation period that in the investigator's estimation has a reasonable assurance of sustained cure and/or is unlikely to recur during the period of the study.*

Seizure disorder *Not excluded: A participant with a history of seizure who has not required medications or had seizure for 3 years.*

Asplenia: any condition resulting in the removal of the spleen or absence of a functional spleen

Psychiatric condition that precludes compliance with the protocol. Specifically excluded are persons with any of the following:

- psychoses within the past 3 years
- ongoing risk for suicide
- history of suicide attempt or gesture within the past 3 years

Female participants: Participant is pregnant and/or breast feeding, or planning to become pregnant during the period of study participation

8 Safety and immunogenicity evaluations

8.1 Considerations for trial start

No considerations for trial start have been identified.

8.2 Initial safety evaluation

For Part A, enrollment for Groups 1 and 2 (i.e., enrollment across all participating HVTUs) will be restricted to a maximum of 1 participant per day per group and restricted to US sites until 5 participants per group have been enrolled. The HVTN 060 Protocol Safety Review Team will review the safety and reactogenicity data reported for the first 72 hours post-vaccination on each of these 10 participants and will determine whether it is safe to proceed with full enrollment in these groups. If enrollment proceeds in the US following the safety review, then enrollment in these groups may also be initiated at sites outside the US.

Enrollment for each subsequent dose group in Part A (Group 3, then Group 4) across all participating HVTUs will be restricted to a maximum of 1 participant per day and restricted to US sites until 5 participants have been enrolled. The HVTN 060 Protocol Safety Review Team will review the safety and reactogenicity data reported for the first 72 hours post-vaccination on each of these 5 participants and will determine whether it is safe to proceed with full enrollment in those groups. If enrollment proceeds in the US following the safety review, then enrollment in that group may also be initiated at sites outside the US.

8.3 Safety considerations for dose escalation

In addition to monitoring participant safety throughout the study period, the HVTN 060 Protocol Safety Review Team will review cumulative safety data available on all participants in each group in Part A (Groups 1 and 2 are done simultaneously) for up to and including the 2 week visit after the second vaccination to determine whether dose escalation may occur (see Section 14.7.5).

The Protocol Safety Review Team may consult ad hoc with the HVTN Safety Monitoring Board (SMB) for these evaluations.

8.4 Safety evaluation for moving from Part A to Part B

The HVTN 060 Protocol Safety Review Team will review all cumulative safety data available on the HIV CTL MEP vaccine from HVTN 056 prior to initiation of enrollment in HVTN 060 Part B. At a minimum, this data review will include data for up to and including the two week post vaccination visit after the second vaccination, for all subjects enrolled in HVTN 056 Part A.

In addition to monitoring participant safety throughout the study period, the HVTN 060 Protocol Safety Review Team will review all cumulative safety data available from Groups 1 through 4 for up to and including 2 weeks after the second vaccination (Day 42) of Group 4. Based on the assessment of this safety data, the HVTN 060 Protocol Safety Review Team will make a decision regarding the appropriateness of moving to Part B as well as regarding the recommended (maximum safe and tolerated) dose to be used in Part B. At the request of the PSRT, the HVTN SMB may perform an additional ad hoc unblinded review of this safety data to make the final determination based on safety for proceeding to Part B.

8.5 Other safety considerations

8.5.1 Exclusion of participants with egg allergy (Part B only)

As lecithin contained in the RC529-SE adjuvant is derived from eggs, individuals with allergy to egg (based on a self-reported medical history) will be excluded from enrollment in Part B of this study.

8.5.2 Exclusion of participants with yeast allergy (Part B only)

Because recombinant human GM-CSF is produced in yeast, individuals with allergy to yeast (based on a self-reported medical history) will be excluded from enrollment in Part B of this study.

8.5.3 Exclusion of participants with allergy to amide type local anesthetics

The HIV-1 *gag* DNA vaccine and *IL-12* DNA adjuvant are formulated in bupivacaine. Individuals with allergy to amide type local anesthetics (based on a self-reported medical history) will be excluded from enrollment in this study.

8.6 Distinguishing intercurrent HIV infection from vaccine-induced positive serology

The study product may elicit an antibody response to HIV proteins. Therefore, vaccine-induced positive serology may occur in this study. Several precautionary measures will be taken to distinguish intercurrent HIV infection from vaccine-induced positive serology :

- Participants will be counseled frequently during the trial on avoidance of HIV infection.
- Participants will be counseled on the risks of seeking HIV testing outside of the network during study participation, and discouraged from doing so.
- Participants will have clinical evaluations at visits specified in Appendix D. Signs or symptoms of an acute HIV infection syndrome, an intercurrent illness consistent with HIV-1 infection, or probable HIV exposure would prompt a diagnostic work-up per the standard HVTN algorithm to determine HIV infection.
  - Diagnostic HIV-1 ELISAs will be performed from blood draws at multiple time points throughout the trial (see Appendices B and C).
  - For US sites the HIV diagnostic laboratory is the Viral and Rickettsial Disease Laboratory, Richmond, California, USA. For non-US sites, local labs may perform HIV diagnostic algorithms (following HVTN SOP) with pre-approval from the HVTN Laboratory Operations Division.
  - If intercurrent HIV-1 infection is suspected or positive test results are observed post-vaccination, the Laboratory Program or approved diagnostic laboratory will proceed with the HVTN algorithm to distinguish vaccine-induced antibody responses from actual HIV infection.
- Continued follow-up will identify subsequent HIV infections or address concerns in participants whose HIV-1 ELISA is positive or indeterminate at the end of the study. All participants who have positive or indeterminate HIV-1 serology at the last study visit (as measured by the Abbott HIV 1, 2 kit or other standard anti-HIV antibody screening test used by blood banks) will be offered follow-up HIV-1 diagnostic testing (HIV-1 ELISA, Western blot, PCR) periodically and free of charge as medically/socially indicated

(approximately every 6 months). This follow-up will be available until the ELISA/Western blot pattern no longer yields positive or indeterminate results or until HIV infection is confirmed.

- Potential participants identified as being HIV infected during screening and participants who become HIV infected during the study will be referred for medical treatment and management of the HIV infection. These individuals will also be referred to appropriate ongoing clinical trials or observation studies.

8.7 Immunogenicity evaluations

The ability of the vaccine to induce humoral responses and/or epitope-specific CD8+ and CD4+ T cell responses will be evaluated by the methods described below. For all assays, cryopreserved specimens from additional timepoints of immunological interest, as indicated in Appendix B and Appendix C, may be tested if positive responses are detected at the primary immunogenicity timepoint.

One of the goals in assaying additional time points (if positive responses are detected at primary immunogenicity time points) would be to examine the whether effector T cells generated in the presence of IL-12 DNA adjuvant improves memory T cell responses.

8.7.1 Humoral immunogenicity studies (HVTN)

8.7.1.1 Binding antibodies by ELISA (Part B only)

Binding antibodies to commercially available Gag will be assessed at the HVTN Laboratory Program by ELISA using single serum dilutions (1/50 or 1/100) on samples from all study participants taken at baseline and 2 weeks after the fourth and fifth vaccination visits. Any of the time points that yield positive results, defined as an OD of ≥ 0.2, in the initial ELISA may be subject to endpoint titration ELISA employing 6 (2-7-fold) serial dilutions of serum beginning at a 1/50 or 1/100. Additional ELISA may be performed in all participants using cryopreserved samples if positive responses are observed at the primary immunogenicity time point (see Appendix C).

8.7.2 Cellular immunogenicity studies for Part A (HVTN)

8.7.2.1 IFN-γ ELISpot

Bulk T cell responses will be assessed by IFN-γ ELISpot using cryopreserved peripheral blood mononuclear cells (PBMC) stimulated overnight with synthetic peptide pools that span the proteins encoded by the vaccine constructs. ELISpot assays will be performed at baseline and 2 weeks post final (third) vaccination. Additional time points may be assayed if positive responses are observed at the primary immunogenicity time point (see Appendix B). Responses will be reported as number of spot forming cells per 10^6 cells/well recognizing any specific peptide pool.

8.7.2.2 Intracellular cytokine staining (ICS)

Flow cytometry will be used to examine HIV-specific CD4+ and CD8+ T cell responses using ICS following stimulation with synthetic HIV peptides that span the proteins encoded by the vaccine construct. ICS assays will be performed at baseline and 2 weeks post final (third) vaccination visit. Additional time points may be assayed if positive responses are observed at the primary immunogenicity time point(see Appendix B). Responses will be reported as percentages of CD4+ or CD8+ T cells recognizing any specific peptide pool.

8.7.3 Cellular immunogenicity studies for Part B (HVTN)

8.7.3.1 IFN-γ ELISpot

Bulk T cell responses will be assessed by IFN-γ ELISpot using cryopreserved PBMC stimulated overnight with synthetic peptide pools that span the proteins encoded by the vaccine constructs. ELISpot assays will be performed at baseline and ELISpot assays will be performed at baseline and at two weeks post final (fifth) vaccination. Additional time points may be assayed if positive responses are observed at the primary immunogenicity time point (see Appendix C). Responses will be reported as number of spot forming cells per 10^6 cells/well recognizing any specific peptide pool.

8.7.3.2 Intracellular cytokine staining (ICS) for Part B

Flow cytometry will be used to examine HIV-specific CD4+ and CD8+ T cell responses using ICS following stimulation with synthetic HIV peptides that span the proteins encoded by the vaccine construct. ICS assays will be performed at baseline and ELISpot assays will be performed at baseline and at two weeks post final (fifth) vaccination. Additional time points may be assayed if positive responses are observed at the primary immunogenicity time point (see Appendix C). Responses will be reported as percentages of CD4+ or CD8+ T cells recognizing any specific peptide pool.

8.7.4 Ancillary studies

Cryopreserved samples may be used by HVTN to perform additional ELISpot and ICS assays to support standardization and validation of these assays, and to evaluate additional immunological assays of interest. These assays may include, but are not limited to, fine epitope mapping by flow cytometry or ELISpot, or flow cytometric tetramer analysis.

8.7.4.1 Humoral immunogenicity studies for Part A (Wyeth)

Evaluation of binding antibody production against IL-12 will be performed on cryopreserved samples stored at the CSR at baseline, at the 2 weeks post-vaccination visits, and at the final study visit.

8.7.4.2 Humoral immunogenicity studies for Part B (Wyeth)

Evaluation of binding antibody production against IL-12 will be performed on cryopreserved samples stored at the CSR at baseline; 2 weeks post first, second and third vaccination visits; and at the final study visit.

Evaluation of binding and neutralizing antibody production against GM-CSF will be performed on cryopreserved samples stored at the Central Specimen Repository (CSR) at baseline and 2 weeks post-fourth and fifth vaccination visits, and at the final study visit.

Neutralizing antibodies to GM-CSF will be assessed by a TF-1 cell proliferation assay, which evaluates the neutralization of GM-CSF biological activity by detecting decreased proliferation of a GM-CSF-dependent cell line (TF-1), using samples obtained at baseline; 2 weeks post fourth and fifth vaccination visits; and at the final study visit. Evaluations will be performed only on samples with demonstrated binding antibody by ELISA.

8.7.4.3 Cellular immunogenicity studies for Part A and Part B (Wyeth)

Tetramer-binding assays will be performed at Wyeth on cryopreserved PBMC stored at the CSR on samples from Part A at baseline and 2 weeks post third immunization, and on samples from Part B at baseline, 2 weeks post third, fourth and fifth vaccination visits and at

the final visit. These analyses will use a panel of tetramers that represent peptides from the HIV CTL MEP in the context of the appropriate HLA molecule (See Table 2-2). The HIV CTL MEP consists of 4 CTL multiepitope peptides designated A, B, C, and J. These peptides range in length from 27 to 47 amino acids**.** Each hybrid peptide contains 1 of 3 different HIV T helper epitopes (and terminal amino acids sequence derived from Env or Gag) in combination with 1 of 4 different CTL "hot spots." All Gag CTL epitopes and CD4 + T cell epitopes in HIV CTL MEP are derived from similar sequences found in GENEVAX® gag-2962. The CTL "hot *spot"* peptides are regions of Clade B Gag and Nef proteins that contain multiple overlapping CTL epitopes. These "hot spots" were selected for use in the HIV CTL MEP vaccine as this combination of epitopes is restricted by 19 HLA types, providing theoretical coverage for 95% African Americans, 97% Thais, 98% Caucasian Americans, and 99% Native Americans. A partial listing of the CTL epitopes and their restricting HLA alleles is provided in Table 2-2 and Table 2-3.

8.7.4.4 T-cell repertoire studies for Part B (Vanderbilt University)

HIV epitope-specific T-cell receptor (TCR) repertoires will be evaluated in vaccinees with detectable CD8+ T-cell responses. Tetramer-positive T cells from cryopreserved PBMC will be sorted and subjected to TCR analysis by PCR [100]. The breadth of TCR repertoires in vaccinees will be compared to matched, HLA-typed controls of HIV+ subjects at different stages of HIV disease. These subjects include a cohort of subjects with control of viremia (10 subjects with viral loads <50 copies off ART, and an additional 20 subjects with viral loads <1,000 copies off ART), actively followed by Dr. Kalams at Vanderbilt University Medical Center. These subjects have signed an informed consent, approved by the Vanderbilt University IRB, for an observational study (NIH-funded) to identify potential correlates of control of HIV viremia.

8.8 HLA typing

Molecular HLA typing may be performed on enrolled participants using cryopreserved PBMC collected at baseline, initially in participants who demonstrate vaccine-induced T-cell responses at post-vaccination time points. Other participants (including placebo recipients) may be HLA-typed to support future studies of immunological interest at the discretion of the protocol chair and the HVTN Laboratory Program. These assays may include, but are not limited to, fine epitope mapping by flow cytometry or ELISpot, or flow cytometric tetramer analysis.

9 Statistical considerations

9.1 Overview

This study is a multicenter, randomized, placebo-controlled, double-blind trial. The data analysis will evaluate safety and immunogenicity data of the study groups.

9.2 Objectives

The primary objective of this trial concerns safety; the secondary objectives concern immunogenicity and social impacts.

9.3 Endpoints

9.3.1 Safety

Assessment of product safety will include clinical observation and monitoring of hematological and chemical parameters. Safety will be evaluated by monitoring participants for local and systemic adverse reactions after each injection and for 9 months after the first injection in Part A and for 15 months after the first injection in Part B. Section 14 describes the safety monitoring plan and reports.

The following parameters will be assessed:

- Local reactogenicity signs and symptoms
- Systemic reactogenicity signs and symptoms
- Laboratory measures of safety
- Adverse and serious adverse experiences

9.3.2 Immunogenicity

Immunogenicity endpoints are:

- HIV-specific cellular responses assessed by IFN-γ ELISpot assays
- HIV-specific cellular responses as assessed by intracellular cytokine staining (ICS)

9.3.3 Social impacts

Social impact variables include any negative experiences or problems the participant experienced due to his/her participation in this study. The following social impacts will be followed during the course of the study: social, travel, work, school, health care, life insurance, health insurance, housing, military and any additional impacts identified by a participant.

9.4 Accrual and sample size

Recruitment will target 156 healthy, HIV-uninfected adult participants.

Part A will consist of 4 groups. Groups 1 and 2 will be enrolled simultaneously with participants randomized to receive either *gag* DNA or placebo (Group 1), or *gag* DNA plus *IL-12* DNA (100 mcg) or placebo (Group 2) in a (10:2) ratio within each group. Groups 3 and

4 will proceed sequentially according to dose escalation rules outlined in Sections 8.3 and 14.7.5. Groups 3 and 4 will each enroll participants randomized to receive either *gag* DNA plus *IL-12* DNA (maximum tolerated dose, MTD) or placebo in a (10:2) ratio.

Part B will consist of 3 groups (Groups 5, 6, 7) and will follow Part A according to rules outlined in Section 8.4. Groups 5, 6, and 7 will be enrolled simultaneously and randomized to receive either *gag* DNA (1500 mcg) for both prime and booster vaccinations or placebo (Group 5), or *gag* DNA plus *IL-12* DNA (MTD) for both prime and booster vaccinations or placebo (Group 6), or *gag* DNA plus *IL-12* DNA (MTD) for prime and HIV CTL MEP for booster vaccinations or placebo (Group 7), each group with a (30:6) ratio for vaccine to placebo.

Note that study design blinds participants and site staff to vaccine or placebo arms of each group, but participants will not be blinded as to which group they are in.

9.4.1 Sample size calculations for safety

The goal of the safety evaluation for this study is to identify safety concerns associated with administration. Sample size calculations for safety are expressed in terms of the ability to detect rare events (e.g. serious adverse events (SAEs).

The ability of the study to identify serious adverse experiences can be expressed by the maximum true rate of events that would be unlikely to be observed and the minimum true rate of events that would very likely be observed.

Specifically, for each vaccine arm of Part A (n=10), there is a 90% chance of observing at least 1 serious adverse experience if the true rate of such an event is at least 21%; there is a 90% chance that we would not observe at least 1 serious adverse experience if the true rate was no more than 1%.

For each vaccine arm of Part B (n=30), there is a 90% chance of observing at least 1 serious adverse experience if the true rate of such an event is at least 8%; there is a 90% chance that we would not observe at least 1 serious adverse experience if the true rate was no more than 0.35%.

Probabilities of observing 0 or 2 or more serious adverse experiences among groups of size 10 and 30 are presented in Table 9-1 for a range of possible true event rates. These calculations provide a more complete picture of the sensitivity of this study design to identify potential safety problems with the vaccine.

Table 9-1 Probability (%) of response for different safety scenarios

True event rate (%)	Pr(0/10)	Pr(2+/10)	Pr(0/30)	Pr(2+/30)
1.0	90.4	0.4	74.0	3.6
3.4	70.0	4.6	34.3	28.3
5.0	59.9	8.6	21.5	44.6
10.0	34.9	26.4	4.2	81.6
20.0	10.7	62.4	0.1	98.9
30.0	2.8	85.1	<0.1	>99.9
40.0	0.6	95.4	<0.1	>99.9
50.0	<0.1	98.9	<0.1	>99.9
60.0	<0.1	99.8	<0.1	>99.9

If none of the 130 participants receiving the vaccine experience serious adverse experiences to the vaccine, the exact 95% 2-sided upper confidence bound for the rate of such reactions in the population is 2.8%. Restricted to any of the vaccine arms (n=10) in Part A, the exact 95% 2-sided upper confidence bound for this rate is 31%. Restricted to any of the vaccine arms (n=30) in Part B, the exact 95% 2-sided upper confidence bound for this rate is 12%.

9.4.2 Sample size calculations for immunogenicity

In Part A, the precision with which response rates can be estimated, based on a sample of 10 vaccines, is limited. The standard error of the estimated response rates depends on the true underlying response rate but can be bounded by 0.16 (= $\sqrt{0.5 \times 0.5 / n}$). Thus, the width of a 95% confidence interval for the response rate in any 1 arm will be no greater than 0.63 (i.e., ±1.96 x 0.16).] In Part B, based on n=30, the standard error of the estimated response rates can be bounded by 0.09 (= $\sqrt{0.5 \times 0.5 / n}$). Thus, the width of a 95% confidence interval for the response rate in any 1 arm will be no greater than 0.35 (i.e., ±1.96 x 0.09).]

Table 1-2 below displays exact confidence intervals for several possible observed rates of response in Part B.

Table 9-2 Exact 95% 2-sided confidence intervals for response rates based on observing a particular rate of responses in the vaccinees (n=30) in Part B.

Observed response rate(%)	Confidence interval (n=30)
50	[31, 69]
60	[41, 77]
70	[51, 85]
80	[61, 92]
90	[73, 98]

In Part B, there is limited power for a formal comparison of immunogenicity response rates between vaccine groups of size n=30 and, hence, formal comparisons were not listed in the objectives. For illustration, we include power calculations for comparison of response rates between vaccine groups (n=30) that can be distinguished with statistical power of 80% and 90% for an exact 2-sided test with a Type I error rate of 0.05 in Table 9-3. Note the sizes of the differences the trial is powered to detect are very large.

Table 9-3 Minimum detectable differences in response rates between 2 groups (of size 30)

	Minimum detectable response rate 2nd group (%)	
True response rate 1st group (%)	80% power	90% power
10	44	49
20	58	63
30	69	74
40	78	83
50	86	90
60	93	96
70	98	>99

An alternative to formal comparisons of groups in Part B is to rank the vaccine groups by their immune response rates. We assess the reliability of this study to select the best group

with respect to the magnitude of their response rates. See Table 9-4 below. Each line in the table shows the results of N=40,000 simulated datasets generated using two (best/ next best) different binomial probabilities for 3 groups of size 30, with the best (highest) response probability used to generate data for one group and the next best response probability used to generate data for the remaining 2 groups. These simulations illustrate the probability of correctly selecting the highest response group 90% (or 80%) of the time [101] given the assumed parameters (best response probability, difference between best and next best).

Table 9-4 Probability of 90% (80%) correct selection of regimen with highest immunology response (n=30 per group) among 3 groups

Next Best response probability (%)	Best response probability (%)	Difference
10	26 (21)	16 (11)
20	39 (34)	19 (14)
30	50 (45)	20 (15)
40	61 (55)	21 (15)
50	70 (65)	20 (15)

9.5 Statistical analysis

All data from enrolled participants will be analyzed according to the initial randomization assignment regardless of how many vaccinations they received. Since enrollment is concurrent with receiving the first vaccination, all enrolled participants will have received one vaccination and therefore will provide some safety data. The analysis is intent-to-treat; however, individuals who are randomized but not enrolled do not contribute data and hence are excluded. Because of blinding and the brief length of time between randomization and enrollment—typically no more than 4 working days, according to the HVTN MOP (Study Operations >Enrollment >Randomization)—very few such individuals are expected.

Analyses for primary endpoints will be performed in SAS. All other descriptive and inferential statistical analyses will be performed using SAS, S-Plus, and/or R statistical software.

No formal multiple comparison adjustments will be employed for safety or immunogenicity endpoints that address specified scientific questions (e.g., humoral- and cellular-based endpoints). However, multiple measurements of a specific type of immune response may be treated as a collection of hypotheses that requires a multiplicity adjustment. For example, determination of cellular immune responses to several different HIV-1 peptide pools as measured by the IFN-γ ELISpot assay may entail a multiplicity adjustment to account for the multiple peptide pools considered.

9.5.1 Analysis variables

The analysis variables consist of baseline variables, safety variables, immunogenicity variables, and social impact variables for primary and secondary objective analyses.

9.5.2 Baseline comparability

Groups will be compared for baseline characteristics including demographics and laboratory measurements, using descriptive statistics.

9.5.3 Safety analysis

Reactogenicity

The number and percentage of participants experiencing each type of reactogenicity sign or symptom will be tabulated by severity and vaccine regimen. For a given sign or symptom, each participant's reactogenicity will be counted once under the maximum severity for all injection visits.

Adverse experiences

Adverse experiences will be analyzed using MedDRA preferred terms. The number and percentage of participants experiencing each specific adverse experience will be tabulated by severity and by relationship to treatment. For the calculations in these tables, each participant's adverse experience will be counted once under the maximum severity or the strongest recorded causal relationship to treatment.

A complete listing of serious adverse experiences for each participant will provide details including severity, relationship to treatment, onset, duration and outcome.

Local laboratory values

Boxplots of local laboratory values by treatment will be generated for baseline values and for values measured during the course of the study. Each boxplot will show the 1st quartile, the median, and the 3rd quartile. Outliers, or values outside the boxplot, will also be plotted. If appropriate, horizontal lines representing boundaries for abnormal values will be plotted.

9.5.4 Immunogenicity analysis

The statistical analysis for immunogenicity will employ the intent-to-treat principle, i.e., all data from enrolled participants will be used according to the initial randomization assignment regardless of how many injections they received. The only exception will be to exclude data from HIV-infected participants at or post infection. Thus, for HIV-infected participants, only immunogenicity data from samples known to be drawn prior to HIV infection will be included in the analysis.

If assay data are qualitative (i.e., positive or negative) then analyses will be performed by tabulating the frequency of positive response for each assay by vaccine regimen at each time point that an assessment is performed. Binomial response rates will be presented with their corresponding exact 95% confidence interval estimates. For Parts A and B, crude rates for each vaccine group will be calculated. The combined placebo groups of Part B (N=18) will be used to estimate the false positive rate. Although, because of the lack of precision in this estimate, net response rates will not be calculated for the vaccine groups.

To compare the response rates of any two vaccine arms, a significant difference will be declared if the 2-sided 95% confidence interval for the difference in response rates between 2 groups excludes 0. In addition to reporting the point prevalence response rates, cumulative and cumulative repeat (over time/visit) probabilities of cellular immune responses will be estimated with corresponding confidence intervals using maximum likelihood based methods [102]. Missing responses will be assumed to be missing at random, i.e., conditional on the observed data the missingness is independent of the unobserved responses.

For continuous assay variables, overall differences between groups at a specific time point will be tested by a 2-sample t-test if the data appear to be normally distributed, or by utilizing the nonparametric Wilcoxon rank sum test if the data are not normally distributed. If a portion of the measurements are censored below the assay quantification limit, then the Gehan-Wilcoxon test will be employed. More sophisticated analyses employing repeated

measures methodology (for example, repeated measures ANOVA or generalized estimating equations) may be utilized to incorporate immune responses over several time points. However, inference from such analyses would be limited by the small sample size of this study. All statistical tests will be 2-sided and will be considered statistically significant if $p \leq 0.05$. Graphical descriptions of the longitudinal immune responses will also be given.

Some immunologic assays have underlying continuous or count-type readout that is often dichotomized into responder/nonresponder categories. For these assays, graphical and tabular summaries of the underlying distributions will be made. These summaries may be performed on transformed data (e.g., log transformation) to better satisfy assumptions of symmetry and homoscedasticity. If group comparisons in these underlying distributions reveal that differences are best summarized as a shift in the location of the distribution, then results will be presented in the form of group means (or medians) with associated confidence intervals and statistical tests for differences between groups as described above. If group comparisons in these underlying distributions reveal that differences are best summarized by a mixture model (i.e., responder and nonresponder subgroups are clearly identifiable) then results will be presented in the form of response rates with associated confidence intervals and statistical tests as described above.

9.5.4.1 Missing data

If the probability of missing immunogenicity measurements depends on either covariates or on the immunogenicity outcomes of participants, then the methods described above may give biased inferences and point estimates. If a substantial amount of immunogenicity data is missing (at least 1 value missing from more than 20% of participants), then secondary analyses of the immunogenicity endpoints will be conducted using methods that relax the missing completely at random assumption to a missing at random assumption. For a univariate binary and quantitative outcome, respectively, a generalized linear model with a binomial or normal error distribution will be used for estimation and testing. For assessing repeated immunogenicity measurements, linear mixed effects models will be used. The models will be fit using maximum likelihood methods, and will include as covariates all available baseline predictors of the missing outcomes. The longitudinal models will also include all observed immunogenicity data.

9.5.5 Social impact analysis

Social impacts will be tabulated by type of event and impact on quality of life. The number and percentage of participants experiencing each type of social impact will also be tabulated by impact on quality of life. For this calculation multiple events of the same type for a participant will be counted once under the maximum impact for all post-vaccination visits.

In addition, a listing will be generated of all participants who experienced a major disturbance of their quality of life due to study participation. The listing includes all social impacts experienced by these participants, descriptions of each impact, duration, impact on quality of life, actions taken by the participant and staff, and whether or not there was a resolution.

9.5.6 Analyses prior to end of study

Safety

Unblinded interim analyses of safety data are prepared at regular intervals for the HVTN Safety Monitoring Board (SMB). Ad hoc interim safety reports may also be prepared for SMB review at the request of the Protocol Safety Review Team.

Immunogenicity

Unblinded immunogenicity analyses will be done only when primary immunogenicity endpoint assay results are available for all samples at the primary immunogenicity time point. Unblinded reports will be provided to the protocol chairs, vaccine developer, other key HVTN members and investigators for the purpose of informing future trial-related decisions after review by the Laboratory Program. Any exceptions must be approved by the SDMC Director and Lab Program Director.

9.6 Randomization of treatment assignments

The randomization sequence will be obtained by computer-generated random numbers and provided to each HVTU by the SDMC using the procedures described in the HVTN MOP (Study Operations>Enrollment>Randomization). At each institution, the pharmacist with primary responsibility for drug dispensing is charged with maintaining security of the randomization list. The randomization will be done in blocks to ensure balance across groups.

This page is intentionally blank

STUDY OPERATIONS

10 Protocol conduct

The protocol will be conducted according to standard HVTN policies and procedures specified in the HVTN MOP (Study Operations), including procedures for the following:

- Protocol registration, activation and implementation
- Informed consent, screening, enrollment
- Clinical and safety assessments
- Safety monitoring and reporting
- Data collection and documentation
- Study follow-up and close-out
- Unblinding of staff and participants
- Quality control
- Protocol monitoring and compliance
- Advocacy and assistance through local and governmental activities to participants regarding social harms associated with the vaccine trial
- Risk reduction counseling
- Outside testing and belief questionnaire

Any policies or procedures that vary from HVTN standards or require additional instructions will be described in the *HVTN 060 Study Specific Procedures* (e.g., instructions for randomization specific to this study).

11 Informed consent

Informed consent is the essential process of ensuring that participants fully understand what will and may happen to them while participating in a research study. The HVTN informed consent form documents that a participant (1) understands the potential risks, benefits, and alternatives to participation, and (2) is willing to participate in an HVTN study. Informed consent is not confined to the signing of the consent form; it also includes all written or verbal study information HVTU staff discuss with the participant, before and during the trial. HVTU staff will obtain informed consent of participants according to the HVTN policies and procedures specified in the HVTN MOP (Study Operations>Informed Consent).

An HVTU may employ recruitment efforts prior to the participant consenting. Some HVTUs use a telephone script to pre-screen people before they come to the clinic for a full screening visit. Participants must sign a screening or protocol-specific consent before any procedures to determine eligibility are performed. HVTUs must submit recruitment and pre-screening materials to IRBs/IECs for human subjects review.

11.1 Screening consent form

Some HVTUs have approval from their local Institutional Review Board (IRB) and/or Independent Ethics Committee (IEC) to use a general screening consent form that allows screening for an unspecified HIV vaccine trial. In this way, HVTU staff can continually screen potential participants, and when needed, proceed quickly to obtain protocol-specific enrollment consent. Sites conducting IRB/IEC–approved general screening or pre-screening may use the results from this screening for determining eligibility in this protocol, provided the tests are conducted within the time period specified in the eligibility criteria.

11.2 Protocol-specific consent form

The protocol-specific consent form describes the study products to be used and all aspects of protocol participation, including screening and enrollment procedures. A sample protocol-specific consent form is located in Appendix A.

Each HVTU is responsible for developing a protocol-specific consent form for local use, based on the sample protocol-specific consent form in Appendix A. The consent form must be developed in accordance with local IRB/IEC requirements and the principles of informed consent as described in Title 45, Code of Federal Regulations (CFR) Part 46 and Title 21 CFR, Part 50, and in the International Conference on Harmonisation (ICH) Guideline 4.8.10. It must be approved by all responsible ethical review bodies before any participants are consented for the study.

The sample form in Appendix A includes interspersed instructions for developing specific content.

11.3 Assessment of understanding

Study staff will ensure that participants fully understand the study before enrolling them. This involves reviewing the informed consent form with the participant, allowing time to reflect on the procedures and issues presented, and answering all questions completely.

An Assessment of Understanding is used to document the participant's understanding of key concepts in an HIV vaccine trial.

When the Assessment of Understanding is used to document the participant's full understanding before the enrollment consent is signed, most IRBs/IECs will require that the participant have been told about the assessment and signed a screening consent. This is because a site may not initiate study procedures without the participant's consent, and administering the Assessment of Understanding can be viewed as a study procedure. The consent process (including the use of the Assessment of Understanding) should be explained thoroughly to the IRB/IEC, whose recommendations should be followed.

The participant must complete the Assessment of Understanding—with assistance of staff, if necessary, in reading and understanding the questions and responses—before first vaccination. Participants should verbalize understanding of all questions answered incorrectly. This process, and the participant's understanding of the key concepts, should be documented in source documentation at the site.

Informed consent does not end with the signing of the consent form. Periodically throughout the study, key study concepts should be reviewed with the participant. At each study visit, HVTU staff should consider reviewing the procedures and requirements for that visit and for the remaining visits.

12 Procedures

Participants are considered to be enrolled only upon receipt of the first study vaccination at Day 0.

HVTU and HVTN Laboratory Program staff will conduct pre-enrollment and post-enrollment study procedures according to HVTN procedures as specified in the HVTN MOP (Study Operations). Any procedures which vary from the HVTN standard will be defined in the *HVTN 060 Study Specific Procedures*.

Pre-enrollment and post-enrollment procedures are performed on all participants (unless otherwise noted) at the time points indicated in Appendix B, Appendix C, and Appendix D, using the blood draw volumes specified.

12.1 Pre-enrollment procedures

Screening assessments and other pre-enrollment procedures are listed in Table 12-1. Timepoints are specified in Appendix D.

Screening procedures are done to determine eligibility and to provide a baseline for comparison of safety data. Screening may occur over the course of several contacts/visits up to and including Day 0 before vaccination. All inclusion and exclusion criteria must be assessed within 56 days before enrollment, unless otherwise specified in Section 7.

The time interval between randomization and enrollment should not exceed 4 working days, as defined in the HVTN MOP (Study Operations). Subsequently, the HVTU registers the participant by scheduling the Day 0 visit (enrollment) via the web-based randomization system, and requests the randomization assignment.

Table 12-1 Pre-enrollment procedures

Screening assessments			
Clinical assessments	Local lab assessments		HIV infection assessments
Medical history	Pregnancy test (females)	Chemistry panel:	HIV screening test
Complete physical exam	Urine dipstick/urinalysis	ALT	
Abbreviated physical exam	CBC with differential	AST	
Concomitant medications	Platelet count	Alkaline phosphatase	
	Syphilis (RPR)	Creatinine	
	T-cell subsets	CPK	
	Hepatitis B		
	Hepatitis C		

Other pre-enrollment procedures	
Screening informed consent (if applicable)	Behavioral risk assessment
Protocol informed consent	Risk reduction counseling
Assessment of understanding	Pregnancy prevention counseling
Specimen collection	HIV pre- and post-test counseling
Obtain demographics	Participant randomization
Confirm eligibility	

12.2 Post-enrollment procedures

Safety assessments, immunogenicity determinations, and other post-enrollment procedures are listed in Table 12-2.

Table 12-2 Post-enrollment procedures

Safety assessments			
Clinical assessments	Local lab assessments		HIV infection assessments
Abbreviated physical exam	Urine dipstick/urinalysis	Chemistry panel:	HIV diagnostic ELISA
Complete physical exam	CBC with differential	ALT	HIV Western blot (if applicable)
Concomitant medications	Platelet count	AST	HIV RNA PCR (if applicable)
Intercurrent illness/AE	Pregnancy test (females)	Alkaline phosphatase	HIV DNA PCR (if applicable)
Reactogenicity	T-cell subsets	Creatinine	
		CPK	

Immunogenicity determinations		
Humoral assay	Cellular assays	Ancillary assays
HIV binding ELISA (Part B)	IFN-γ ELISpot	MHC tetramer binding
	Intracellular cytokine staining	Antibody to IL-12
		Antibody to GM-CSF
		T cell repertoire studies (Part B)

Other post-enrollment procedures		
Vaccination administration	Risk reduction counseling	Outside testing/belief assessment
Specimen collection and shipping	Pregnancy prevention counseling	Cryopreservation/storage of specimens
HIV pre- and post-test counseling	HLA typing	Participant unblinding
	Social impact assessment	

12.3 Total blood volumes

Required blood volumes are shown in Appendices B and C. Not shown is any additional blood volume that would be required if a safety lab needs to be repeated, or if a serum pregnancy test needs to be performed. The additional blood volume would likely be minimal. The total blood volume drawn for each participant will not exceed 500 mL in any 8-week period.

12.4 Laboratory procedures

A laboratory procedures manual will provide further guidelines for operational issues concerning the clinical laboratories and phlebotomy. The procedures include general specimen collection guidelines, special considerations for blood collection, HIV testing guidelines, suggested tube types with catalog numbers, guidelines for processing whole blood, and labeling guidelines.

In specific situations the blood collection tubes will be redirected to another laboratory for special screening criteria or safety issues. In these cases special shipping instructions will be provided in Special Instructions posted on the HVTN website.

13 Study product preparation and administration

HVTU pharmacists should consult the *Pharmacy Guidelines and Instructions for DAIDS Clinical Trials Networks* manual for standard pharmacy operations procedures. The protocol schema and vaccine regimen are shown in Section 13.1. See the Investigator's Brochure for further information about study products.

13.1 Schema and vaccine regimen

HIV-1 *gag* DNA Vaccine: HIV *gag* DNA Plasmid (GENEVAX® *gag*-2962)

IL-12 DNA adjuvant: *IL-12* DNA (GENEVAX® *IL-12*-6285 Part A and GENEVAX® *IL-12*-4532 Part B)

CTL MEP vaccine: HIV CTL Multi-Epitope Peptide (MEP vaccine) 4 peptides (A, B, C, and J) 250 mcg per peptide, i.e. 1000 mcg total/dose labeled as HIV CTL Peptides (625 mcg/mL per peptide) co-formulated (by the HVTU pharmacist) with:

- RC529-SE: Adjuvant at 50 mcg dose labeled as RC-529 SE 500 mcg/mL in 10% Squalene
- GM-CSF: Granulocyte-macrophage colony-stimulating factor cytokine adjuvant at 250 mcg dose

Placebo: Sodium Chloride Injection USP, 0.9% (NaCl 0.9%, Normal Saline)

Part A		Priming			Boosting	
		Vaccination schedule in months (days)				
Group	Treatment	0 (0)	1 (28)	3 (84)	6 (168)	9 (273)
1	T1	HIV *gag* DNA Plasmid 1500 mcg IM*	HIV *gag* DNA Plasmid 1500 mcg IM*	HIV *gag* DNA Plasmid 1500 mcg IM*		
	C1	Placebo for HIV *gag* DNA Plasmid 0.75 mL IM*	Placebo for HIV *gag* DNA Plasmid 0.75 mL IM*	Placebo for HIV *gag* DNA Plasmid 0.75 mL IM*		
2	T2	HIV *gag* DNA Plasmid 1500 mcg admixed with *IL-12* DNA 100 mcg IM*	HIV *gag* DNA Plasmid 1500 mcg admixed with *IL-12* DNA 100 mcg IM*	HIV *gag* DNA Plasmid 1500 mcg admixed with *IL-12* DNA 100 mcg IM*		
	C2	Placebo for HIV *gag* DNA Plasmid / *IL-12* DNA 0.8 mL IM*	Placebo for HIV *gag* DNA Plasmid / *IL-12* DNA 0.8 mL IM*	Placebo for HIV *gag* DNA Plasmid / *IL-12* DNA 0.8 mL IM*		
3	T3	HIV *gag* DNA Plasmid 1500 mcg admixed with *IL-12* DNA 500 mcg IM*	HIV *gag* DNA Plasmid 1500 mcg admixed with *IL-12* DNA 500 mcg IM*	HIV *gag* DNA Plasmid 1500 mcg admixed with *IL-12* DNA 500 mcg IM*		
	C3	Placebo for HIV *gag* DNA Plasmid / *IL-12* DNA 1 mL IM*	Placebo for HIV *gag* DNA Plasmid / *IL-12* DNA 1 mL IM*	Placebo for HIV *gag* DNA Plasmid / *IL-12* DNA 1 mL IM*		
4	T4	HIV *gag* DNA Plasmid 1500 mcg admixed with *IL-12* DNA 1500 mcg IM*	HIV *gag* DNA Plasmid 1500 mcg admixed with *IL-12* DNA 1500 mcg IM*	HIV *gag* DNA Plasmid 1500 mcg admixed with *IL-12* DNA 1500 mcg IM*		
	C4	Placebo for HIV *gag* DNA Plasmid / *IL-12* DNA 1.5 mL IM*	Placebo for HIV *gag* DNA Plasmid / *IL-12* DNA 1.5 mL IM*	Placebo for HIV *gag* DNA Plasmid / *IL-12* DNA 1.5 mL IM*		

Part B		Priming			Boosting	
		Vaccination schedule in months (days)				
Group	Treatment	0 (0)	1 (28)	3 (84)	6 (168)	9 (273)
5	T5	HIV *gag* DNA Plasmid 1500 mcg IM*	HIV *gag* DNA Plasmid 1500 mcg IM*	HIV *gag* DNA Plasmid 1500 mcg IM*	HIV *gag* DNA Plasmid 1500 mcg IM*	HIV *gag* DNA Plasmid 1500 mcg IM*
	C5	Placebo for HIV *gag* DNA Plasmid 0.75 mL IM*	Placebo for HIV *gag* DNA Plasmid 0.75 mL IM*	Placebo for HIV *gag* DNA Plasmid 0.75 mL IM*	Placebo for HIV *gag* DNA Plasmid 0.75 mL IM*	Placebo for HIV *gag* DNA Plasmid 0.75 mL IM*
6	T6	HIV *gag* DNA Plasmid 1500 mcg admixed with *IL-12* DNA 1500 mcg IM*	HIV *gag* DNA Plasmid 1500 mcg admixed with *IL-12* DNA 1500 mcg IM*	HIV *gag* DNA Plasmid 1500 mcg admixed with *IL-12* DNA 1500 mcg IM*	HIV *gag* DNA Plasmid 1500 mcg admixed with *IL-12* DNA 1500 mcg IM*	HIV *gag* DNA Plasmid 1500 mcg admixed with *IL-12* DNA 1500 mcg IM*
	C6	Placebo for HIV *gag* DNA Plasmid / *IL-12* DNA 1.5 mL IM*	Placebo for HIV *gag* DNA Plasmid / *IL-12* DNA 1.5 mL IM*	Placebo for HIV *gag* DNA Plasmid / *IL-12* DNA 1.5 mL IM*	Placebo for HIV *gag* DNA Plasmid / *IL-12* DNA 1.5 mL IM*	Placebo for HIV *gag* DNA Plasmid / *IL-12* DNA 1.5 mL IM*
7	T7	HIV *gag* DNA Plasmid 1500 mcg admixed with *IL-12* DNA 1500 mcg IM*	HIV *gag* DNA Plasmid 1500 mcg admixed with *IL-12* DNA 1500 mcg IM*	HIV *gag* DNA Plasmid 1500 mcg admixed with *IL-12* DNA 1500 mcg IM*	HIV CTL Peptides 1000 mcg admixed with RC529-SE 50 mcg and GM-CSF 250mcg IM*	HIV CTL Peptides 1000 mcg admixed with RC529-SE 50 mcg and GM-CSF 250mcg IM*
	C7	Placebo for HIV *gag* DNA Plasmid / *IL-12* DNA 1.5 mL IM*	Placebo for HIV *gag* DNA Plasmid / *IL-12* DNA 1.5 mL IM*	Placebo for HIV *gag* DNA Plasmid / *IL-12* DNA 1.5 mL IM*	Placebo for HIV CTL Peptides / RC529-SE/ GM-CSF 1 mL IM*	Placebo for HIV CTL Peptides / RC529-SE/ GM-CSF 1 mL IM*

*All vaccinations will be administered in the same deltoid at each vaccination visit (unless medically contraindicated for that deltoid). See Section 13.3.1.

Enrollment in Groups 1 and 2 will occur simultaneously. See Section 8.2 regarding initial safety evaluation.

Enrollment in Groups 3 and 4 will be sequential, according to dose escalation criteria in Section 8.3.

Enrollment in Groups 5, 6, and 7 will occur simultaneously. See Section 8.4 regarding safety evaluation for moving from Part A to Part B.

Group 1

T1: HIV *gag* DNA Plasmid 1500 mcg administered as 0.75 mL IM in same deltoid at months 0, 1, and 3 (unless medically contraindicated for that deltoid).

C1: Placebo for HIV *gag* DNA Plasmid 0.75 mL IM in same deltoid at months 0, 1, and 3 (unless medically contraindicated for that deltoid).

Group 2

T2: HIV *gag* DNA Plasmid 1500 mcg admixed with *IL-12* DNA 100 mcg administered as 0.8 mL IM in same deltoid at months 0, 1, and 3 (unless medically contraindicated for that deltoid).

C2: Placebo for HIV *gag* DNA Plasmid / *IL-12* DNA 0.8 mL IM in same deltoid at months 0, 1, and 3 (unless medically contraindicated for that deltoid).

Group 3

T3: HIV *gag* DNA Plasmid 1500 mcg admixed with *IL-12* DNA 500 mcg administered as 1 mL IM in same deltoid at months 0, 1, and 3 (unless medically contraindicated for that deltoid).

C3: Placebo for HIV *gag* DNA Plasmid / *IL-12* DNA 1 mL IM in same deltoid at months 0, 1, and 3 (unless medically contraindicated for that deltoid).

Group 4

T4: HIV *gag* DNA Plasmid 1500 mcg admixed with *IL-12* DNA 1500 mcg administered as 1.5 mL IM in same deltoid at months 0, 1, and 3 (unless medically contraindicated for that deltoid).

C4: Placebo for HIV *gag* DNA Plasmid / *IL-12* DNA 1.5 mL IM in same deltoid at months 0, 1, and 3 (unless medically contraindicated for that deltoid).

Group 5

T5: HIV *gag* DNA Plasmid 1500 mcg administered as 0.75 mL IM in same deltoid at months 0, 1, 3, 6 and 9 (unless medically contraindicated for that deltoid).

C5: Placebo for HIV *gag* DNA Plasmid 0.75 mL IM in same deltoid at months 0, 1, 3, 6 and 9 (unless medically contraindicated for that deltoid).

Group 6

T6: HIV *gag* DNA Plasmid 1500 mcg admixed with *IL-12* DNA 1500 mcg administered as 1.5 mL IM in same deltoid at months 0, 1, 3, 6 and 9 (unless medically contraindicated for that deltoid).

C6: Placebo for HIV *gag* DNA Plasmid / *IL-12* DNA 1.5 mL IM in same deltoid at months 0, 1, 3, 6 and 9 (unless medically contraindicated for that deltoid).

Group 7

T7: HIV *gag* DNA Plasmid 1500 mcg admixed with *IL-12* DNA 1500 mcg administered as 1.5 mL IM in same deltoid at months 0, 1, and 3 (unless medically contraindicated for that deltoid)

and

HIV CTL Peptides 1000 mcg admixed with RC529-SE 50 mcg and GM-CSF 250mcg administered as 1 mL IM in same deltoid at months 6 and 9 (unless medically contraindicated for that deltoid).

C7: Placebo for HIV *gag* DNA Plasmid admixed with Placebo for *IL-12* DNA 1.5 mL IM in same deltoid at months 0, 1, and 3 (unless medically contraindicated for that deltoid)

and

Placebo for HIV CTL Peptides / RC529-SE / GM-CSF 1 mL IM in same deltoid at months 6 and 9 (unless medically contraindicated for that deltoid).

13.2 Study product formulation and preparation

See the Investigator's Brochure for further information about study product(s).

13.2.1 HIV *gag* DNA Plasmid (HIV-1 *gag* DNA Vaccine, GENEVAX *gag-2962*)

HIV *gag* DNA Plasmid is supplied as a sterile, clear, colorless, preservative-free aqueous formulation. Each 2 mL vial contains 1.2 mL +/– 0.1 mL of GENEVAX *gag*-2962 2 mg/mL in 30 mM Citrate Buffer pH 6.5 containing 150 mM NaCl, 0.01% ethylenediamine tetraacetic acid (EDTA) and 0.25% bupivacaine-HCl. The HIV *gag* DNA Plasmid should be stored at 2° to 8°C until use. The product is contraindicated in participants with known hypersensitivity to bupivacaine.

13.2.2 *IL-12* DNA (*IL-12* adjuvant, GENEVAX *IL-12*-6285 Part A and GENEVAX *IL-12*-4532 Part B)

GENEVAX *IL-12*-6285 and GENEVAX *IL-12*-4532 are clear colorless liquids. They are supplied as a sterile, preservative-free aqueous formulations. Each formulation will be labeled as *IL-12* DNA with different lot numbers for each formulation. For Part A, each 2 mL vial contains 0.9 mL +/– 0.1 mL of GENEVAX *IL-12*-6285 at a concentration of 2 mg/mL in 30 mM Citrate Buffer pH 6.5 containing 150 mM NaCl, 0.01% ethylenediamine tetraacetic acid (EDTA) and 0.25% bupivacaine-HCl. These vials will be labeled with Lot # 76172001A. For Part B, each 2 mL vial contains 0.9 mL +/– 0.1 mL of GENEVAX *IL-12*-4532 at a concentration of 2 mg/mL in 30 mM Citrate Buffer pH 6.5 containing 150 mM NaCl, 0.01% ethylenediamine tetraacetic acid (EDTA) and 0.25% bupivacaine-HCl. These vials will be labeled with Lot # 76180001A. Both lots should be stored at 2° to 8°C until ready for use. The product is contraindicated in participants with known hypersensitivity to bupivacaine.

13.2.3 HIV CTL Peptides (HIV CTL Multi-Epitope Peptide, HIV Th/CTL Peptides, HIV CTL MEP, MEP)

The HIV CTL is a lyophilized product. The tetravalent peptides are formulated at 625 mcg/mL of each peptide in 3% mannitol and 12.5mM succinic acid, pH2. HIV CTL peptides product is lyophilized at 0.6 mL fill volume per vial. After reconstitution with 0.6 mL of diluent, the product appears spongy in texture and white in color. The product will be intramuscularly delivered in the presence of RC529-SE with GM-CSF as a total volume of 1 mL per dose. The HIV CTL Peptides product should be stored refrigerated at 2° to 8°C until ready for use.

13.2.4 RC529-SE (labeled as RC-529 SE 500 mcg/mL in 10% Squalene)

RC529-SE is a sterile stable (milky white) emulsion. It is an oil-in-water formulation of RC529, an aminoalkylglucosamine phosphate (AGP) synthetic analog of monophosphoryl Lipid A (MPL). RC529-SE is supplied as 0.5 mL volume in 2.0 mL vials at a concentration of 500 mcg/mL. *This product contains lecithin derived from eggs.* It should be stored at 2° to 8°C until use. *Do not freeze.* RC529-SE should be mixed well prior to use by inverting the vial. Once RC529-SE is mixed with MEP, it must be administered within 3 hours

13.2.5 Peptide Diluent (Diluent for HIV CTL Peptides, Sodium Succinate diluent)

Diluent for HIV CTL Peptides is used for reconstitution of lyophilized HIV CTL peptides product and raising pH closer to physiological range for delivery. It is a clear, colorless solution supplied as 1 mL volume in 2 mL vials. It should be kept at 2° to 8°C until use. Do not freeze.

13.2.6 rGM-CSF (GM-CSF, Leukine®)

rGM-CSF adjuvant is a licensed product. It is supplied as a clear and colorless solution. The package insert supplied with this product contains information regarding the product. The vials must be stored as directed by the manufacturer of the product.

13.2.7 Placebo (Sodium Chloride Injection USP, 0.9%, NaCl 0.9%)

Sodium chloride injection USP, 0.9% will be used as the placebo for all groups. The volume to be administered will vary from group to group to maintain the blind between active and placebo treatment (T and C) in each group. The vials must be stored as directed by the manufacturer of the product unless otherwise instructed.

13.2.8 Preparation of HIV *gag* DNA Plasmid 1500 mcg or Placebo (Groups 1 and 5)

HIV gag *DNA Plasmid 1500 mcg*

All vials should be visually inspected prior to use. If any vial contains material different from its description above, do not use the vial and contact the protocol pharmacist. Using aseptic technique, the pharmacist will use a 3 mL syringe to withdraw 0.75 mL of HIV *gag* DNA Plasmid from the vial. The pharmacist will label the syringe as HIV *gag* DNA Plasmid 1500mgc or Placebo. It should be stored at 2° to 8°C (36° to 46°F) if not for immediate injection. The syringe must be labeled with a 4 hour expiration date/time.

Any unused portion of entered vials and expired pre-filled syringes should be disposed of in accordance with institutional or pharmacy policy.

Placebo (for HIV gag *DNA Plasmid 1500 mcg)*

All vials should be visually inspected prior to use. If any vial contains material different from its description above, do not use the vial and contact the protocol pharmacist. Using aseptic technique, the pharmacist will use a 3 mL syringe to withdraw 0.75 mL of Sodium Chloride Injection USP, 0.9% from the vial. The pharmacist will label the syringe as HIV *gag* DNA Plasmid 1500mgc or Placebo. It should be stored at 2° to 8°C (36° to 46°F) if not for immediate injection. The syringe must be labeled with a 4 hour expiration date/time.

Any unused portion of entered vials and expired pre-filled syringes should be disposed of in accordance with institutional or pharmacy policy.

13.2.9 Preparation of HIV *gag* DNA Plasmid 1500 mcg / *IL-12* DNA 100 mcg or Placebo (Group 2)

HIV gag *DNA Plasmid 1500 mcg /* IL-12 *DNA 100 mcg*

All vials should be visually inspected prior to use. If any vial contains material different from its description above, do not use the vial and contact the protocol pharmacist. Using aseptic technique, the pharmacist will use a 0.3 mL insulin syringe/needle to withdraw 0.08 mL (8 unit marking) from the vial of *IL-12* DNA 2mg/mL vial (Lot # 76172001A). The contents of this syringe will then be slowly injected into the vial containing 1.2 mL of HIV *gag* DNA Plasmid (2 mg/mL). The pharmacist should avoid creating bubbles. To prevent aerosolization, the pharmacist should, before removing the needle from the vial, bring the needle above the level of the liquid and allow excess air to rise into the syringe. Remove the syringe from the vial and discard into a sharp's container. The pharmacist will mix the solution in the vial by gentle swirling (avoid creating bubbles). The final product should be a clear colorless liquid containing 1.28 mL of a 15:1 mixture of HIV *gag* DNA Plasmid*: IL-12 DNA*, consisting of 1875 mcg/mL of HIV *gag* DNA and 125 mcg/mL of *IL-12* DNA. Using a new 3 mL syringe, the pharmacist will withdraw 0.8 mL of this final product. The pharmacist will label the syringe as HIV gag DNA Plasmid 1500 mcg / *IL-12* DNA 100 mcg or placebo. The final product must be administered within 4 hours after preparation. It must be stored at 2 to 8°C if not administered immediately after preparation.

Any unused portion of entered vials and expired pre-filled syringes should be disposed of in accordance with institutional or pharmacy policy.

Placebo (for HIV gag *DNA Plasmid 1500 mcg /* IL-12 *DNA 100 mcg)*

Using aseptic technique, the pharmacist will withdraw 0.8 mL of sodium chloride injection USP, 0.9% into a 3 mL syringe. The pharmacist will label the syringe as HIV gag DNA Plasmid 1500 mcg / *IL-12* DNA 100 mcg or placebo. The final product must be administered

within 4 hours after preparation. It must be stored at 2 to 8°C if not administered immediately after preparation.

Any unused portion of entered vials and expired pre-filled syringes should be disposed of in accordance with institutional or pharmacy policy.

13.2.10 Preparation of HIV *gag* DNA Plasmid 1500 mcg / *IL-12* DNA 500 mcg or Placebo (Group 3)

HIV gag *DNA Plasmid 1500 mcg /* IL-12 *DNA 500 mcg*

All vials should be visually inspected prior to use. If any vial contains material different from its description above, do not use the vial and contact the protocol pharmacist. Using aseptic technique, the pharmacist will use a 1 mL syringe to withdraw 0.4 mL from the vial of IL-12 DNA 2mg/mL vial (Lot # 76172001A). The contents of this syringe will then be slowly injected into the vial containing 1.2 mL of HIV *gag* DNA Plasmid (2 mg/mL). The pharmacist should avoid creating bubbles. To prevent aerosolization, the pharmacist should, before removing the needle from the vial, bring the needle above the level of the liquid and allow excess air to rise into the syringe. Remove the syringe from the vial and discard into a sharp's container. The pharmacist will mix the solution in the vial by gentle swirling (avoid creating bubbles). The final product should be a clear colorless liquid containing 1.6 mL of a 3:1 mixture of HIV *gag* DNA Plasmid*: IL-12* DNA, consisting of 1500 mcg/mL of HIV *gag* DNA and 500 mcg/mL of *IL-12* DNA. Using a new 3 mL syringe, the pharmacist will withdraw 1 mL of this final product. The pharmacist will label the syringe as HIV *gag* DNA Plasmid 1500 mcg / *IL-12* DNA 500 mcg or placebo. The final product must be administered within 4 hours after preparation. It must be stored at 2 to 8°C if not administered immediately after preparation.

Any unused portion of entered vials and expired pre-filled syringes should be disposed of in accordance with institutional or pharmacy policy.

Placebo (for HIV gag *DNA Plasmid 1500 mcg /* IL-12 *DNA 500 mcg)*

Using aseptic technique, the pharmacist will withdraw 1 mL of sodium chloride injection USP, 0.9% into a 3 mL syringe. The pharmacist will label the syringe as HIV *gag* DNA Plasmid 1500 mcg / *IL-12* DNA 500 mcg or placebo. The final product must be administered within 4 hours after preparation. It must be stored at 2 to 8°C if not administered immediately after preparation.

Any unused portion of entered vials and expired pre-filled syringes should be disposed of in accordance with institutional or pharmacy policy.

13.2.11 Preparation of HIV *gag* DNA Plasmid 1500 mcg / IL-12 DNA 1500 mcg or Placebo (Group 4)

HIV gag *DNA Plasmid 1500 mcg /* IL-12 *DNA 1500 mcg*

All vials should be visually inspected prior to use. If any vial contains material different from its description above, do not use the vial and contact the protocol pharmacist. Using aseptic technique, the pharmacist will use a 3 mL syringe to withdraw 0.9 mL of HIV *gag* DNA Plasmid 2 mg/mL vial. The contents of this syringe will then be slowly injected into the vial containing 0.9 mL of *IL-12* DNA (2 mg/mL) (Lot # 76172001A). The pharmacist should avoid creating bubbles. To prevent aerosolization, the pharmacist should, before removing the needle from the vial, bring the needle above the level of the liquid and allow excess air to rise into the syringe. Remove the syringe from the vial and discard into a sharp's container. The pharmacist will mix the solution in the vial by gentle swirling (avoid creating bubbles).

The final product should be a clear colorless liquid containing 1.8 mL of a 1:1 mixture of HIV *gag* DNA Plasmid*: IL-12* DNA, consisting of 1000 mcg/mL of HIV *gag* DNA and 1000 mcg/mL of *IL-12* DNA. Using a new 3 mL syringe, the pharmacist will withdraw 1.5 mL of this final product. The pharmacist will label the syringe as HIV *gag* DNA Plasmid 1500 mcg / *IL-12* DNA 1500 mcg or placebo. The final product must be administered within 4 hours after preparation. It must be stored at 2 to 8°C if not administered immediately after preparation.

Any unused portion of entered vials and expired pre-filled syringes should be disposed of in accordance with institutional or pharmacy policy.

Placebo (for HIV gag *DNA Plasmid 1500 mcg /* IL-12 *DNA 1500 mcg)*

Using aseptic technique, the pharmacist will withdraw 1.5 mL of sodium chloride injection USP, 0.9% into a 3 mL syringe. The pharmacist will label the syringe as HIV *gag* DNA Plasmid 1500 mcg / *IL-12* DNA 1500 mcg or placebo. The final product must be administered within 4 hours after preparation. It must be stored at 2 to 8°C if not administered immediately after preparation.

Any unused portion of entered vials and expired pre-filled syringes should be disposed of in accordance with institutional or pharmacy policy.

13.2.12 Preparation of HIV *gag* DNA Plasmid 1500 mcg / IL-12 DNA 1500 mcg or Placebo (Groups 6 and 7)

HIV gag *DNA Plasmid 1500 mcg /* IL-12 *DNA 1500 mcg*

All vials should be visually inspected prior to use. If any vial contains material different from its description above, do not use the vial and contact the protocol pharmacist. Using aseptic technique, the pharmacist will use a 3 mL syringe to withdraw 0.9 mL of HIV *gag* DNA Plasmid 2 mg/mL vial. The contents of this syringe will then be slowly injected into the vial containing 0.9 mL of *IL-12* DNA (2 mg/mL) (Lot # 76180001A). The pharmacist should avoid creating bubbles. To prevent aerosolization, the pharmacist should, before removing the needle from the vial, bring the needle above the level of the liquid and allow excess air to rise into the syringe. Remove the syringe from the vial and discard into a sharp's container. The pharmacist will mix the solution in the vial by gentle swirling (avoid creating bubbles). The final product should be a clear colorless liquid containing 1.8 mL of a 1:1 mixture of HIV *gag* DNA Plasmid*: IL-12* DNA, consisting of 1000 mcg/mL of HIV *gag* DNA and 1000 mcg/mL of *IL-12* DNA. Using a new 3 mL syringe, the pharmacist will withdraw 1.5 mL of this final product. The pharmacist will label the syringe as HIV gag DNA Plasmid 1500 mcg / IL-12 DNA 1500 mcg or placebo. The final product must be administered within 4 hours after preparation. It must be stored at 2 to 8°C if not administered immediately after preparation.

Any unused portion of entered vials and expired pre-filled syringes should be disposed of in accordance with institutional or pharmacy policy.

Placebo (for HIV gag *DNA Plasmid 1500 mcg /* IL-12 *DNA 1500 mcg)*

Using aseptic technique, the pharmacist will withdraw 1.5 mL of sodium chloride injection USP, 0.9% into a 3 mL syringe. The pharmacist will label the syringe as HIV gag DNA Plasmid 1500 mcg / IL-12 DNA 1500 mcg or placebo. The final product must be administered within 4 hours after preparation. It must be stored at 2 to 8°C if not administered immediately after preparation.

Any unused portion of entered vials and expired pre-filled syringes should be disposed of in accordance with institutional or pharmacy policy.

13.2.13 Preparation of MEP 1000mcg / RC529-SE 50mcg / GM-CSF 250 mcg or Placebo (Group 7)

MEP 1000mcg / RC529-SE 50mcg / GM-CSF 250 mcg

All vials should be visually inspected prior to use. If any vial contains material different from its description above, do not use the vial and contact the protocol pharmacist. Using aseptic technique (in a laminar flow hood), the pharmacist will use a 1 mL syringe to withdraw 0.6 mL of sodium succinate diluent from the vial. The contents of this syringe will then be injected into the HIV CTL Peptides vial (625 mcg/mL per peptide). The vial should be gently tapped to re-suspend the HIV CTL Peptides until no solid cake material is visible. Still using aseptic technique (in a laminar flow hood), the pharmacist will use a new 1 mL syringe to withdraw 0.15 mL of RC529-SE. The contents of this syringe will then be added to the re-suspended HIV CTL Peptides vial. Again, using a new 1 mL syringe, the pharmacist will aseptically withdraw 0.75 mL of GM-CSF injection (500 mcg/mL) from the vial and inject the contents of this syringe into the vial containing re-suspended HIV CTL Peptides/ RC529-SE. The vial should then be swirled gently to mix the contents.

Prior to injection, using a 3 mL syringe, the pharmacist will withdraw 1 mL of the HIV CTL Peptides/ RC529-SE/GM-CSF admixture. The final product (HIV CTL Peptides 1000 mcg total dose admixed with 50 mcg of RC529-SE and 250 mcg of GM-CSF) should be labeled as PEP/RC529-SE/GM-CSF or placebo. The pharmacist must apply a blue overlay to the syringe and the syringe must be labeled with a 3 hour expiration (based on time of preparation). The syringe should be stored at 2° to 8°C (36° to 46°F) if not for immediate injection.

Any unused portion of entered vials and expired pre-filled syringes should be disposed of in accordance with institutional or pharmacy policy.

Placebo for MEP 1000mcg / RC529-SE 50mcg / GM-CSF 250 mcg

All vials should be visually inspected prior to use. If any vial contains material different from its description above, do not use the vial and contact the protocol pharmacist. Using aseptic technique, the pharmacist should withdraw 1 mL of sodium chloride injection USP, 0.9% into a 3 mL syringe. The syringe should be labeled as MEP/RC529-SE/GM-CSF or placebo. The pharmacist must apply a blue overlay to the syringe and the syringe must be labeled with a 3 hour expiration date (based on time of preparation). The syringe should be stored at 2° to 8°C (36° to 46°F) if not for immediate injection (to prevent unblinding).

Any unused portion of entered vials and expired pre-filled syringes should be disposed of in accordance with institutional or pharmacy policy.

13.2.14 Pharmacy procedures to preserve blinding

The pharmacist will prepare the doses for administration and dispense to the clinic. To preserve blinding, a blue overlay will be placed on syringes containing MEP / RC529-SE / GM-CSF or its placebo.

13.3 Study product administration

All vaccinations are to be given intramuscularly into the deltoid muscle. The needle length should be appropriate for the participant’s weight.

When preparing a dose in a syringe and administering the dose, consideration should be given to the volume of solution that may remain in the needle after the dose is administered. The pharmacy and clinic staffs are encouraged to work together to administer the dose specified in the protocol.

The person who administers the dose must not be the same individual who is responsible for clinical follow-up of that dose. At sites where registered pharmacists are legally authorized to administer drug, the HVTU may choose to have the HVTU pharmacist administer the vaccinations.

13.3.1 Administration of vaccinations

Syringes should be kept refrigerated at 2° to 8°C (36° to 46°F) until just prior to injection unless the administration is immediately after preparation. Administer the vaccine into the deltoid muscle after preparation of the site with alcohol. The same deltoid should be used at each vaccination visit unless medically contraindicated. If the injection is administered in the opposite deltoid due to a medical contraindication, the appropriate study staff should document this clearly and submit a Study Product Administration Error form. Under this circumstance, this is NOT a protocol violation.

As significant adverse experiences—including cardiac arrest and death—have occurred with intravascular delivery of bupivacaine, it is essential that aspiration for blood be performed prior to injection of all *gag* DNA, *IL-12* DNA, or their placebo injections to ensure that i.v. delivery does not occur.

13.4 Study product acquisition

The HIV *gag* DNA Plasmid, *IL-12* DNA (both lots), HIV CTL peptides product, RC529-SE, GM-CSF, and Diluent for HIV CTL peptides will be provided by Wyeth Vaccines Research. Sodium chloride injection USP, 0.9% will not be provided through the protocol but must be purchased at the site.

At US HVTUs the pharmacist can obtain study products from the NIAID Clinical Research Products Management Center (CRPMC) by following the ordering procedures given in the section on Study Product Control in *Pharmacy Guidelines and Instructions for DAIDS Clinical Trials Networks*.

At non-US HVTUs the pharmacist can obtain study products from the NIAID Clinical Research Products Management Center (CRPMC). Once a non-US HVTU is registered for the study and all required documents have been received by DAIDS (CRPMC), the Pharmacist can order product by following the procedures given in the *HVTN 060 Study Specific Procedures (SSP).*

13.5 Pharmacy records

The HVTU pharmacist is required to maintain complete records of all study products received from the CRPMC and subsequently dispensed. For US sites, all unused study products must be returned to the CRPMC after the study is completed or terminated. The procedures are included in the sections on Study Product Placebo and Drug Dispensing in *Pharmacy Guidelines and Instructions for DAIDS Clinical Trials Networks*. For non-US sites, specific instructions will be sent to the site after the study is completed or terminated.

The pharmacist of record is responsible for maintaining randomization codes and randomization confirmation notices for each participant in a secure manner.

14 Safety monitoring and review

14.1 Assessing reactogenicity

Reactogenicity assessments are performed for all participants following each vaccination. HVTU staff will assess reactogenicity according to standard HVTN procedures as specified in the HVTN MOP (Study Operations>Safety Assessment>Reactogenicity). Any procedures which vary from the HVTN standard will be defined in *HVTN 060 Study Specific Procedures*.

The reactogenicity assessment period is for 3 days following the vaccination. Clinic staff will follow new or unresolved reactogenicity symptoms present at Day 3 to resolution. The schedule is shown in Table 14-1.

Assessments to be performed:

- Systemic symptoms: body temperature, malaise and/or fatigue, myalgia, headache, chills, arthralgia, nausea, vomiting
- Local symptoms (proximal to injection site): pain, tenderness
- Vaccine-related lesions: erythema, induration
- Axillary lymph nodes (required only when reactogenicity assessments are performed by HVTU staff): lymph node tenderness, enlargement

Table 14-1 Schedule of reactogenicity assessments

Day	Time	Performed by
0[a]	Baseline: before vaccination	HVTU staff
	Early: 25 to 45 minutes after vaccination	HVTU staff
	Between early assessment and 11:59pm Day 0	HVTU staff or participant
1	Between 12:00am and 11:59pm Day 1	HVTU staff or participant
2	Between 12:00am and 11:59pm Day 2	HVTU staff or participant
3	Between 12:00am and 11:59pm Day 3	HVTU staff or participant

[a]Day of vaccination

14.2 Grading adverse events

Local and systemic signs and symptoms are assessed and graded based on *the Division of AIDS Table for Grading the Severity of Adult and Pediatric Adverse Events* (DAIDS AE Grading Table), Version 1.0, December, 2004.

14.3 Adverse events reporting and safety pause rules

All adverse events are reported to the SDMC on the appropriate case report form (CRF) according to procedures in the HVTN MOP (Study Operations>Safety Assessments>Adverse Experiences). The mechanism of reporting certain Grade II and higher grade vaccine-related symptoms and adverse events to the SDMC Clinical Affairs staff is depicted in Table 14-2 (for Part A) and Table 14-3 (for Part B). The mechanism of reporting SAEs and other events meeting expedited adverse event (EAE) reporting requirements to DAIDS is specified in Section 14.4, *Expedited adverse event reporting*.

Vaccinations may be suspended for safety concerns other than those described in Table 14-2 and Table 14-3, **or before pause rules are met,** if, in the judgment of the Protocol Safety Review Team, participant safety may be threatened.

If a safety pause is triggered, SDMC Clinical Affairs staff notifies the HVTN 060 Protocol Safety Review Team, PAB (Pharmaceutical Affairs Branch), RCC/RAB (Regulatory Compliance Center / Regulatory Affairs Branch), and participating HVTUs that vaccinations are held until further notice. In addition, in the case of a safety pause that can be triggered by only 1 event (see Table 14-2 and Table 14-3), SDMC Clinical Affairs staff notifies the HVTN SMB; DAIDS notifies the US FDA.

Each HVTU is responsible for submitting to its IRB/IEC (per local requirements) protocol-related safety information (IND safety reports, notifications of vaccination holds due to safety pause rules, etc.) as required by the local institution.

Table 14-2 Adverse experience notification and safety pause rules for Part A

Toxicity	Symptom/AE	HVTU action	SDMC action	Criterion for pause
Grade 4	Any lab abnormality, local or systemic symptom, or adverse event	Immediate	Immediate	≥1 vaccine-related symptom at specified grade
Grade 3	Any lab abnormality, local or systemic symptom, or adverse event	Immediate	Immediate	≥1 vaccine-related symptom at specified grade
Grade 2	Erythema, induration	Immediate	Immediate	≥1 vaccine-related symptom at specified grade
Grade 2	Lab abnormality, fever, vomiting, other clinical AE (except subjective local and systemic symptoms, erythema or induration)	Prompt	Prompt	≥2 ppts experience the same vaccine-related symptom at specified grade or higher

Immediate: HVTU notifies SDMC Clinical Affairs staff immediately by pager or live phone (not voice mail). SDMC Clinical Affairs staff notifies PSRT, PAB, RCC/RAB, HVTN SMB, and participating HVTUs as soon as possible that vaccinations are held until further notice.

Prompt: HVTU submits completed forms as soon as possible (and within 1 working day of receiving notice of event). SDMC Clinical Affairs staff notifies PSRT, PAB, RCC/RAB, HVTN SMB, and participating HVTUs as soon as possible during working hours (Pacific Standard Time)—or, if the information was received during off hours, by the morning of the next work day—that vaccinations are held until further notice.

Phone number and pager number are listed in *HVTN 060 Study Specific Procedures*

Table 14-3 Adverse experience notification and safety pause rules for Part B

Toxicity	Symptom/AE	HVTU action	SDMC action	Criterion for pause
Grade 4	Any lab abnormality, local or systemic symptom, adverse event	Immediate	Immediate	≥1 vaccine-related symptom at specified grade
Grade 3	Lab abnormality, clinical AE (except subjective local and systemic symptoms)	Immediate	Immediate	≥1 vaccine-related symptom at specified grade
	Fever, vomiting	Prompt	Prompt	≥1 ppts report the same vaccine-related symptom at specified grade
	Subjective local and systemic symptoms: pain, tenderness, malaise and/or fatigue, headache, chills, nausea, myalgia, arthralgia	Prompt	Prompt	≥2 and ≥10% of ppts report the same vaccine-related symptom at specified grade or higher
Grade 2 or 3	Erythema, induration	Prompt	Prompt	≥2 participants experience the same vaccine-related symptom at specified grade or higher
Grade 2	Lab abnormality, fever, vomiting, clinical AE (except subjective local and systemic symptoms)	Prompt	Prompt	≥2 participants experience the same vaccine-related symptom at specified grade or higher

Immediate: HVTU notifies SDMC Clinical Affairs staff immediately by pager or live phone (not voice mail). SDMC Clinical Affairs staff notifies PSRT, PAB, RCC/RAB, HVTN SMB, and participating HVTUs as soon as possible that vaccinations are held until further notice.

Prompt: HVTU submits completed forms as soon as possible (and within 1 working day of receiving notice of event). SDMC Clinical Affairs staff notifies PSRT, PAB, RCC/RAB, HVTN SMB, and participating HVTUs as soon as possible during working hours (Pacific Standard Time)—or, if the information was received during off hours, by the morning of the next work day—that vaccinations are held until further notice.

Phone number and pager number are listed in *HVTN 060 Study Specific Procedures*

14.3.1 Follow up and resolution after a safety pause

The HVTN 060 Protocol Safety Review Team reviews safety data and decides whether permanent discontinuation of vaccination is appropriate, consulting the HVTN SMB and the US FDA if necessary. DAIDS notifies the US FDA of the decision. SDMC Clinical Affairs staff notifies participating HVTUs and PAB of the decision. Resumption of vaccinations after a safety pause triggered by a Grade 4 event will require consultation with HVTN SMB and FDA.

14.4 Expedited adverse event reporting

The expedited adverse event (EAE) reporting requirements and definitions for this study and the methods for expedited reporting of adverse events (AEs) to the DAIDS Regulatory Compliance Center (RCC) Safety Office are defined in *The Manual for Expedited Reporting of Adverse Events to DAIDS* (DAIDS EAE Manual), dated May 6, 2004. The DAIDS EAE Manual is available on the RCC website: http://rcc.tech-res-intl.com.

AEs reported on an expedited basis must be documented on the DAIDS Expedited Adverse Event Reporting Form (EAE Reporting Form) available on the RCC website: http://rcc.tech-res-intl.com.

HVTUs must submit the form to DAIDS through the RCC. The HVTU must simultaneously send a copy of the EAE Reporting Form to SDMC Clinical Affairs staff.

14.4.1 EAE reporting level

This study uses the Standard Level of expedited AE reporting as defined in the DAIDS EAE Manual.

14.4.2 Study products for expedited reporting to DAIDS

The study products that must be considered in determining relationships of AEs requiring expedited reporting to DAIDS are:

- GENEVAX® *gag*-2962/placebo
- GENEVAX® *IL-12*-6285/placebo
- GENEVAX® *IL-12*-4532/placebo
- HIV CTL MEP/placebo

14.4.3 Grading severity of events

The Division of AIDS Table for Grading the Severity of Adult and Pediatric Adverse Events (DAIDS AE Grading Table), Version 1.0, December, 2004, must be used and is available on the RCC website at http://rcc.tech-res-intl.com.

14.4.4 EAE reporting periods

AEs must be reported on an expedited basis at the Standard Level during the Protocol-defined EAE Reporting Period, which is the entire study duration for an individual subject (from study enrollment until study completion or discontinuation of the subject from study participation for any reason).

After the end of the Protocol-defined EAE Reporting Period stated above, sites must report serious, unexpected, clinical suspected adverse drug reactions if the study site staff becomes aware of the event on a passive basis, i.e., from publicly available information.

14.4.5 Biosafety concerns: Recombinant DNA Advisory Committee and Institutional Biosafety Committee review and reporting

Human gene transfer trials conducted at or sponsored by institutions receiving NIH funds must be submitted to the NIH Office of Biotechnology Activities (OBA) for review by the Recombinant DNA Advisory Committee (RAC) and to Institutional Biosafety Committees (IBCs) in accordance with the NIH *Guidelines for Research Involving Recombinant DNA Molecules* (NIH Guidelines). A major role for the RAC and the IBC is to examine clinical trials that involve the transfer of recombinant DNA to humans.

The NIH Guidelines create exceptions to RAC review, but the HVTN 060 protocol team determined that the exceptions did not apply. Therefore, the protocol team, jointly with Wyeth, submitted the application for RAC review and responded to RAC comments. The application followed the guidance provided in the NIH Guidelines.

After an initial review of the protocol submission, including a brief summary of the submission's main features and other relevant information, members of the RAC determined that the submission did not require an in-depth review and public RAC discussion. RAC notified the HVTN of this decision on August 16, 2004.

Without documented exemptions, the NIH Guidelines state that "[n]o research participant shall be enrolled in a human gene transfer experiment until the RAC review process has been completed (see Appendix M-I-B, RAC Review Requirements); IBC approval (from the clinical trial site) has been obtained; Institutional Review Board (IRB) approval has been obtained; and all applicable regulatory authorization(s) have been obtained." *Section IV-B-7-b-(6)*

Once RAC review is completed, investigators at each site are responsible for obtaining Institutional Biosafety Committee (IBC) approval and periodic review of the research per NIH Guidelines *Section IV-B-7-b-(6)* and *Section IV-B-2-b*. IBC review and approval must be documented by the investigator and submitted as part of protocol registration for this trial.

The HVTN and DAIDS will ensure that reporting requirements to RAC, as outlined in *Appendix M-I-C-1.Initiation of the Clinical Investigation*, *Appendix M-I-C-3.Annual Reports*, and *Appendix M-I-C-4.Safety Reporting* are satisfied per the NIH Guidelines.

14.5 Participant departure from schedule of clinic visits or vaccinations

14.5.1 Delaying vaccinations for a participant

Under certain circumstances a participant's scheduled vaccination may need to be held. These include but are not limited to the following:

- Receipt of live attenuated vaccines within 30 days prior to vaccination
- Receipt of medically indicated subunit or killed vaccines (e.g., influenza, pneumococcal) within 14 days prior to vaccination
- Use of other investigational research agents within 30 days prior to vaccination
- Receipt of blood products or immunoglobulin within 45 days prior to vaccination
- Prevaccination abnormal vital signs or clinical symptoms that may mask assessment of vaccine reaction

Vaccinations cannot be administered outside the window period specified in the *HVTN 060 Study Specific Procedures*.

14.5.2 Stopping vaccinations for a participant

Under certain circumstances, an individual participant's vaccinations will be stopped. Such participants should be encouraged to participate in follow-up visits and all protocol-related procedures (unless medically contraindicated) for the remainder of the trial. Specific events that will result in stopping a participant's vaccination schedule include the following:

- Clinically significant condition (i.e., a condition that affects the immune system or for which continued vaccinations and/or blood draws may pose additional risk), including but not limited to the following:
  - HIV infection (requires termination from the study)
  - Pregnancy (regardless of outcome)
  - Any Grade 4 local or systemic symptom, lab abnormality, or adverse experience, that is subsequently confirmed to be *possibly*, *probably*, or *definitely* related to vaccination

  - Any Grade 3 lab abnormality or other clinical adverse experience (exception: fever or vomiting and subjective local and systemic symptoms) that is subsequently confirmed to be *possibly*, *probably*, or *definitely* related to vaccination
  - Type 1 hypersensitivity associated with vaccination
- Inability to receive vaccination within the specified period for the designated study visit (see *HVTN 060 Study Specific Procedures*)
- Investigator determination in consultation with the study chair and statistician, e.g., for repeated nonadherence to protocol requirements

14.5.3 Participant termination from the study

Under certain circumstances, an individual participant may be terminated from participation in this study. Specific events that will result in early termination include:

- Participant refused to participate further
- Participant relocated to an area without a nearby HVTU and remote follow-up is not possible
- HVTU determined that the participant is lost to follow-up
- Participant becomes HIV-infected
- Investigator determination in consultation with the study chair and statistician, e.g., for repeated nonadherence to protocol requirements

14.6 Study termination (for all participants)

This study may be terminated by the determination of the HVTN 060 Protocol Safety Review Team, HVTN Safety Monitoring Board, US FDA, US NIH, vaccine developer or regulatory authority (e.g., IRB or IEC), as well as local regulatory authority for non-US sites. See Section 14.7 for discussion of the safety review process.

14.7 HVTN review of cumulative safety data

Routine safety review begins at enrollment, and occurs daily, weekly, monthly and every 4 months during the study.

Reviews proceed from a standardized set of protocol-specific safety data reports. These reports are produced by SDMC and annotated with queries to the HVTU and additional notes. Events are tracked by the internal reports until resolution. Other reports, containing the queries and notes, are distributed to the HVTN 060 Protocol Safety Review Team. The following reports are produced:

- Clinical quality control
- Safety review
- Pre-existing conditions
- Adverse events (AEs) requiring review
- Adverse event/concomitant medication
- WBC/differential
- Safety summary

More detailed information regarding the contents and distribution of these reports can be found in the HVTN MOP.

14.7.1 Daily review

Blinded daily safety reviews are routinely conducted by the SDMC Clinical Affairs staff for SAEs and events that meet safety pause criteria (Table 14-2 and Table 14-3).

14.7.2 Weekly review

Blinded weekly safety reviews are routinely performed by the SDMC Clinical Affairs staff and by the HVTN 060 Protocol Safety Review Team. After the vaccinations and the final 2-week safety visits are completed, less frequent safety reviews may be scheduled at the discretion of the Protocol Safety Review Team. The SDMC Clinical Affairs staff reviews reports of all clinical values that fall outside of the standard HVTN safety parameters (see HVTN MOP [Study Operations>Standard Reports>Clinical Safety Review>Weekly Safety Review Reports]). Values identified during the review that are considered questionable, inconsistent, or unexplained are referred to the HVTU clinic coordinator for verification.

The Protocol Safety Review Team is composed of the following required members:

- Protocol chair and co-chair
- HVTN clinical trials physician
- SDMC Clinical Affairs staff member
- DAIDS medical officer

The protocol team clinic coordinator, protocol specialist, and vaccine developer representative may also be included at the request of the Protocol Safety Review Team.

14.7.3 Quarterly review

In addition to the detailed clinical monitoring reports discussed above, protocol-specific summary reports of reactogenicity and AE data are provided to the HVTN 060 protocol team and the HVTN Phase I/II Committee in a blinded fashion approximately once per quarter.

14.7.4 Safety Monitoring Board review

The HVTN Safety Monitoring Board is composed of the following individuals:

- SMB Chair
- DAIDS Medical Officer representative
- Non-US representative
- US representative
- Statistician
- Clinician
- HVTN Director

Members of the HVTN Safety Monitoring Board are not directly affiliated with the protocols under review. The safety monitoring board will review unblinded safety data approximately every 4 months. This review is designed to provide confirmation with respect to ad hoc review requests as well as increase overall sensitivity for detecting potential safety problems by looking across multiple protocols that use the same or similar vaccine candidates. The

review consists of evaluation of unblinded safety data, including comparisons of adverse experiences in vaccine and placebo recipients in aggregate, as well as review of individual SAE reports. The Safety Monitoring Board will conduct additional special reviews at the request of the HVTN 060 Protocol Safety Review Team.

14.7.5 Review for dose escalation

The Protocol Safety Review Team will examine the safety and reactogenicity events for all participants in Groups 1 and 2 combined and Group 3 to date and will discuss every event that triggers the pause rules and determine the advisability of continuing the dose escalation. At a minimum, if 2 or more participants enrolled in a given group report the same Grade 4, vaccine-related reactogenicity or adverse experience, then further vaccinations will be permanently suspended (in that group and any other group at a higher dose level). As noted in the pausing rules (see Section 14.3.1), any such Grade 4 event will require protocol safety review team consultation with the US FDA and HVTN SMB. Additionally, vaccinations may be suspended for any safety concern if, in the judgment of the protocol safety review team, participant safety may be threatened.

14.7.6 Review for advancing from Part A to Part B

See Section 8.4.

References

1. UNAIDS, WHO. AIDS epidemic update. **2004**;UNAIDS/04.45E.

2. Centers for Disease Control and Prevention. HIV/AIDS surveillance report. **2003**.

3. UNAIDS. Report on the global HIV/AIDS epidemic. **2004**;UNAIDS/04.16E.

4. World Health Organization. AIDS surveillance in the Americas. **1998**.

5. Bloch C. Epidemiological HIV/AIDS situation in Argentina. Actualizaciones en SIDA **2001**;**9 Suppl**:15-24.

6. Sanchez JL, Whittington WLH, Zuckerman RA, Ashley RL, Lama JR, Galvan R. Bacterial STDs and HSV-2 acquisition among MSM HIV seroconverters in Peru. Conf HIV Pathog Treatm, Buenos Aires, Argentina, **7-8-2001**.

7. Lama JR, Goicochea LP, Chion M, Alva J, Ueda L, Cairo J. [Comportamiento sexual y factores de riesgo para ETS e infeccion VIH entre hombres que tienen sexo con otros hombres de Lima, Peru]. Pan-American Conference on AIDS, Lima, Peru, **12-3-1997**.

8. Tabet S, Sanchez J, Lama J, et al. HIV, syphilis and heterosexual bridging among Peruvian men who have sex with men. AIDS **2002**;16:1271-7.

9. Schechter M, Strathdee SA, Martindale SL, et al. Evidence of elevated HIV incidence and relapse to unsafe sex among young men having sex with men (MSM) in Vancover Canada. Int Conf AIDS, **6-28-1998**.

10. Fauci AS. The AIDS epidemic--considerations for the 21st century. N Engl J Med **1999**;341:1046-50.

11. Varmus H, Nathanson N. Science and the control of AIDS. Science **1998**;280:1815.

12. Yang OO, Kalams SA, Rosenzweig M, et al. Efficient lysis of human immunodeficiency virus type 1-infected cells by cytotoxic T lymphocytes. J Virol **1996**;70:5799-806.

13. Yang OO, Kalams SA, Trocha A, et al. Suppression of human immunodeficiency virus type 1 replication by CD8+ cells: evidence for HLA class I-restricted triggering of cytolytic and noncytolytic mechanisms. J Virol **1997**;71:3120-8.

14. Wagner L, Yang OO, Garcia-Zepeda EA, et al. Beta-chemokines are released from HIV-1-specific cytolytic T-cell granules complexed to proteoglycans. Nature **1998**;391:908-11.

15. Moodycliffe AM, Shreedhar V, Ullrich SE, et al. CD40-CD40 ligand interactions in vivo regulate migration of antigen-bearing dendritic cells from the skin to draining lymph nodes. J Exp Med **2000**;191:2011-20.

16. Ahlers JD, Belyakov IM, Thomas EK, Berzofsky JA. High-affinity T helper epitope induces complementary helper and APC polarization, increased CTL, and protection against viral infection. J Clin Invest **2001**;108:1677-85.

17. Geginat J, Sallusto F, Lanzavecchia A. Cytokine-driven proliferation and differentiation of human naive, central memory, and effector memory CD4(+) T cells. J Exp Med **2001**;194:1711-9.

18. Wolff JA, Malone RW, Williams P, et al. Direct gene transfer into mouse muscle in vivo. Science **1990**;247:1465-8.

19. Tang DC, DeVit M, Johnston SA. Genetic immunization is a simple method for eliciting an immune response. Nature **1992**;356:152-4.

20. Ulmer JB, Donnelly JJ, Parker SE, et al. Heterologous protection against influenza by injection of DNA encoding a viral protein. Science **1993**;259:1745-9.

21. McDonnell WM, Askari FK. DNA vaccines. N Engl J Med **1996**;334:42-5.

22. Wang R, Epstein J, Baraceros FM, et al. Induction of CD4(+) T cell-dependent CD8(+) type 1 responses in humans by a malaria DNA vaccine. Proc Natl Acad Sci U S A **2001**;98:10817-22.

23. Le TP, Coonan KM, Hedstrom RC, et al. Safety, tolerability and humoral immune responses after intramuscular administration of a malaria DNA vaccine to healthy adult volunteers. Vaccine **2000**;18:1893-901.

24. Tacket CO, Roy MJ, Widera G, Swain WF, Broome S, Edelman R. Phase 1 safety and immune response studies of a DNA vaccine encoding hepatitis B surface antigen delivered by a gene delivery device. Vaccine **1999**;17:2826-9.

25. Lu S, Arthos J, Montefiori DC, et al. Simian immunodeficiency virus DNA vaccine trial in macaques. J Virol **1996**;70:3978-91.

26. Egan MA, Charini WA, Kuroda MJ, et al. Simian immunodeficiency virus (SIV) gag DNA-vaccinated rhesus monkeys develop secondary cytotoxic T-lymphocyte responses and control viral replication after pathogenic SIV infection. J Virol **2000**;74:7485-95.

27. Barouch DH, Santra S, Schmitz JE, et al. Control of viremia and prevention of clinical AIDS in rhesus monkeys by cytokine-augmented DNA vaccination. Science **2000**;290:486-92.

28. Amara RR, Villinger F, Altman JD, et al. Control of a mucosal challenge and prevention of AIDS by a multiprotein DNA/MVA vaccine. Science **2001**;292:69-74.

29. MacGregor RR, Boyer JD, Ugen KE, et al. First human trial of a DNA-based vaccine for treatment of human immunodeficiency virus type 1 infection: safety and host response. J Infect Dis **1998**;178:92-100.

30. Donnelly JJ, Ulmer JB, Liu MA. DNA vaccines. Life Sci **1997**;60:163-72.

31. Ihata A, Watabe S, Sasaki S, et al. Immunomodulatory effect of a plasmid expressing CD40 ligand on DNA vaccination against human immunodeficiency virus type-1. Immunology **1999**;98:436-42.

32. Kim JJ, Ayyavoo V, Bagarazzi ML, et al. Development of a multicomponent candidate vaccine for HIV-1. Vaccine **1997**;15:879-83.

33. Kim JJ, Ayyavoo V, Bagarazzi ML, et al. In vivo engineering of a cellular immune response by coadministration of IL-12 expression vector with a DNA immunogen. J Immunol **1997**;158:816-26.

34. Kim JJ, Bagarazzi ML, Trivedi N, et al. Engineering of in vivo immune responses to DNA immunization via codelivery of costimulatory molecule genes. Nat Biotechnol **1997**;15:641-6.

35. Kusakabe K, Xin KQ, Katoh H, et al. The timing of GM-CSF expression plasmid administration influences the Th1/Th2 response induced by an HIV-1-specific DNA vaccine. J Immunol **2000**;164:3102-11.

36. Ahlers JD, Dunlop N, Alling DW, Nara PL, Berzofsky JA. Cytokine-in-adjuvant steering of the immune response phenotype to HIV-1 vaccine constructs: granulocyte-macrophage colony-stimulating factor and TNF-alpha synergize with IL-12 to enhance induction of cytotoxic T lymphocytes. J Immunol **1997**;158:3947-58.

37. Iwasaki A, Stiernholm BJ, Chan AK, Berinstein NL, Barber BH. Enhanced CTL responses mediated by plasmid DNA immunogens encoding costimulatory molecules and cytokines. J Immunol **1997**;158:4591-601.

38. Okada E, Sasaki S, Ishii N, et al. Intranasal immunization of a DNA vaccine with IL-12- and granulocyte-macrophage colony-stimulating factor (GM-CSF)-expressing plasmids in liposomes induces strong mucosal and cell-mediated immune responses against HIV-1 antigens. J Immunol **1997**;159:3638-47.

39. Kim JJ, Maguire HC, Jr., Nottingham LK, et al. Coadministration of IL-12 or IL-10 expression cassettes drives immune responses toward a Th1 phenotype. J Interferon Cytokine Res **1998**;18:537-47.

40. Belyakov IM, Ahlers JD, Clements JD, Strober W, Berzofsky JA. Interplay of cytokines and adjuvants in the regulation of mucosal and systemic HIV-specific CTL. J Immunol **2000**;165:6454-62.

41. van der Meide PH, Villinger F, Ansari AA, et al. Stimulation of both humoral and cellular immune responses to HIV-1 gp120 by interleukin-12 in Rhesus macaques. Vaccine **2002**;20:2296-302.

42. Kobayashi M, Fitz L, Ryan M, et al. Identification and purification of natural killer cell stimulatory factor (NKSF), a cytokine with multiple biologic effects on human lymphocytes. J Exp Med **1989**;170:827-45.

43. Chehimi J, Starr SE, Frank I, et al. Natural killer (NK) cell stimulatory factor increases the cytotoxic activity of NK cells from both healthy donors and human immunodeficiency virus-infected patients. J Exp Med **1992**;175:789-96.

44. Kohl S, Sigaroudinia M, Charlebois ED, Jacobson MA. Interleukin-12 administered in vivo decreases human NK cell cytotoxicity and antibody-dependent cellular cytotoxicity to human immunodeficiency virus-infected cells. J Infect Dis **1996**;174:1105-8.

45. Romani L, Puccetti P, Bistoni F. Interleukin-12 in infectious diseases. Clin Microbiol Rev **1997**;10:611-36.

46. Ma X, D'Andrea A, Kubin M, et al. Production of interleukin-12. Res Immunol **1995**;146:432-8.

47. Takenaka H, Maruo S, Yamamoto N, et al. Regulation of T cell-dependent and -independent IL-12 production by the three Th2-type cytokines IL-10, IL-6, and IL-4. J Leukoc Biol **1997**;61:80-7.

48. Fantuzzi L, Gessani S, Borghi P, et al. Induction of interleukin-12 (IL-12) by recombinant glycoprotein gp120 of human immunodeficiency virus type 1 in human monocytes/macrophages: requirement of gamma interferon for IL-12 secretion. J Virol **1996**;70:4121-4.

49. Gately MK, Desai BB, Wolitzky AG, et al. Regulation of human lymphocyte proliferation by a heterodimeric cytokine, IL-12 (cytotoxic lymphocyte maturation factor). J Immunol **1991**;147:874-82.

50. Bertagnolli MM, Lin BY, Young D, Herrmann SH. IL-12 augments antigen-dependent proliferation of activated T lymphocytes. J Immunol **1992**;149:3778-83.

51. Gately MK, Wolitzky AG, Quinn PM, Chizzonite R. Regulation of human cytolytic lymphocyte responses by interleukin-12. Cell Immunol **1992**;143:127-42.

52. Scott P. IL-12: initiation cytokine for cell-mediated immunity. Science **1993**;260:496-7.

53. Germann T, Gately MK, Schoenhaut DS, et al. Interleukin-12/T cell stimulating factor, a cytokine with multiple effects on T helper type 1 (Th1) but not on Th2 cells. Eur J Immunol **1993**;23:1762-70.

54. Afonso LC, Scharton TM, Vieira LQ, Wysocka M, Trinchieri G, Scott P. The adjuvant effect of interleukin-12 in a vaccine against Leishmania major. Science **1994**;263:235-7.

55. Mahon BP, Ryan MS, Griffin F, Mills KH. Interleukin-12 is produced by macrophages in response to live or killed Bordetella pertussis and enhances the efficacy of an acellular pertussis vaccine by promoting induction of Th1 cells. Infect Immun **1996**;64:5295-301.

56. Lindblad EB, Elhay MJ, Silva R, Appelberg R, Andersen P. Adjuvant modulation of immune responses to tuberculosis subunit vaccines. Infect Immun **1997**;65:623-9.

57. Miller MA, Skeen MJ, Ziegler HK. Nonviable bacterial antigens administered with IL-12 generate antigen-specific T cell responses and protective immunity against Listeria monocytogenes. J Immunol **1995**;155:4817-28.

58. Germann T, Bongartz M, Dlugonska H, et al. Interleukin-12 profoundly up-regulates the synthesis of antigen-specific complement-fixing IgG2a, IgG2b and IgG3 antibody subclasses in vivo. Eur J Immunol **1995**;25:823-9.

59. Buchanan JM, Vogel LA, Van Cleave VH, Metzger DW. Interleukin 12 alters the isotype-restricted antibody response of mice to hen eggwhite lysozyme. Int Immunol **1995**;7:1519-28.

60. Rakhmilevich AL, Timmins JG, Janssen K, Pohlmann EL, Sheehy MJ, Yang NS. Gene gun-mediated IL-12 gene therapy induces antitumor effects in the absence of toxicity: a direct comparison with systemic IL-12 protein therapy. J Immunother **1999**;22:135-44.

61. Rakhmilevich AL, Janssen K, Turner J, Culp J, Yang NS. Cytokine gene therapy of cancer using gene gun technology: superior antitumor activity of interleukin-12. Hum Gene Ther **1997**;8:1303-11.

62. Rakhmilevich AL, Janssen K, Hao Z, Sondel PM, Yang NS. Interleukin-12 gene therapy of a weakly immunogenic mouse mammary carcinoma results in reduction of spontaneous lung metastases via a T-cell-independent mechanism. Cancer Gene Ther **2000**;7:826-38.

63. Weber SM, Shi F, Heise C, Warner T, Mahvi DM. Interleukin-12 gene transfer results in CD8-dependent regression of murine CT26 liver tumors. Ann Surg Oncol **1999**;6:186-94.

64. Kim JJ, Trivedi NN, Nottingham LK, et al. Modulation of amplitude and direction of in vivo immune responses by co-administration of cytokine gene expression cassettes with DNA immunogens. Eur J Immunol **1998**;28:1089-103.

65. Boyer JD, Cohen AD, Ugen KE, et al. Therapeutic immunization of HIV-infected chimpanzees using HIV-1 plasmid antigens and interleukin-12 expressing plasmids. AIDS **2000**;14:1515-22.

66. Yoshida S, Kashiwamura SI, Hosoya Y, et al. Direct immunization of malaria DNA vaccine into the liver by gene gun protects against lethal challenge of Plasmodium berghei sporozoite. Biochem Biophys Res Commun **2000**;271:107-15.

67. Shan M, Liu K, Fang H. [DNA vaccination of the induction of immune responses by codelivery of IL-12 expression vector with hepatitis C structural antigens]. Zhonghua Gan Zang Bing Za Zhi **1999**;7:236-9.

68. Operschall E, Schuh T, Heinzerling L, Pavlovic J, Moelling K. Enhanced protection against viral infection by co-administration of plasmid DNA coding for viral antigen and cytokines in mice. J Clin Virol **1999**;13:17-27.

69. Hanlon L, Argyle D, Bain D, et al. Feline leukemia virus DNA vaccine efficacy is enhanced by coadministration with interleukin-12 (IL-12) and IL-18 expression vectors. J Virol **2001**;75:8424-33.

70. Noormohammadi AH, Hochrein H, Curtis JM, Baldwin TM, Handman E. Paradoxical effects of IL-12 in leishmaniasis in the presence and absence of vaccinating antigen. Vaccine **2001**;19:4043-52.

71. Iwasaki A, Dela Cruz CS, Young AR, Barber BH. Epitope-specific cytotoxic T lymphocyte induction by minigene DNA immunization. Vaccine **1999**;17:2081-8.

72. Pachuk CJ, Ciccarelli RB, Samuel M, et al. Characterization of a new class of DNA delivery complexes formed by the local anesthetic bupivacaine. Biochim Biophys Acta **2000**;1468:20-30.

73. Berna Biotech announces the registration of the hepatitis B vaccine SUPERVAX in Argentina [press release]. Berna Biotech; **9-8-2003**. http://www.bernabiotech.com/news/latestnews/20030908_01.html, accessed September 15, 2003.

74. Grabstein KH, Urdal DL, Tushinski RJ, et al. Induction of macrophage tumoricidal activity by granulocyte-macrophage colony-stimulating factor. Science **1986**;232:506-8.

75. Perkins RC, Vadhan-Raj S, Scheule RK, Hamilton R, Holian A. Effects of continuous high dose rhGM-CSF infusion on human monocyte activity. Am J Hematol **1993**;43:279-85.

76. Rossman MD, Ruiz P, Comber P, Gomez F, Rottem M, Schreiber AD. Modulation of macrophage Fc gamma receptors by rGM-CSF. Exp Hematol **1993**;21:177-83.

77. Inaba K, Inaba M, Romani N, et al. Generation of large numbers of dendritic cells from mouse bone marrow cultures supplemented with granulocyte/macrophage colony-stimulating factor. J Exp Med **1992**;176:1693-702.

78. Hasan MS, Agosti JM, Reynolds KK, Tanzman E, Treanor JJ, Evans TG. Granulocyte macrophage colony-stimulating factor as an adjuvant for hepatitis B vaccination of healthy adults. J Infect Dis **1999**;180:2023-6.

79. Ramanathan RK, Potter DM, Belani CP, et al. Randomized trial of influenza vaccine with granulocyte-macrophage colony-stimulating factor or placebo in cancer patients. J Clin Oncol **2002**;20:4313-8.

80. Somani J, Lonial S, Rosenthal H, Resnick S, Kakhniashvili I, Waller EK. A randomized, placebo-controlled trial of subcutaneous administration of GM-CSF as a vaccine adjuvant: effect on cellular and humoral immune responses. Vaccine **2002**;21:221-30.

81. Anandh U, Bastani B, Ballal S. Granulocyte-macrophage colony-stimulating factor as an adjuvant to hepatitis B vaccination in maintenance hemodialysis patients. Am J Nephrol **2000**;20:53-6.

82. Kapoor D, Aggarwal SR, Singh NP, Thakur V, Sarin SK. Granulocyte-macrophage colony-stimulating factor enhances the efficacy of hepatitis B virus vaccine in previously unvaccinated haemodialysis patients. J Viral Hepat **1999**;6:405-9.

83. Hess G, Kreiter F, Kosters W, Deusch K. The effect of granulocyte-macrophage colony-stimulating factor (GM-CSF) on hepatitis B vaccination in haemodialysis patients. J Viral Hepat **1996**;3:149-53.

84. Evans TG, Schiff M, Graves B, et al. The safety and efficacy of GM-CSF as an adjuvant in hepatitis B vaccination of chronic hemodialysis patients who have failed primary vaccination. Clin Nephrol **2000**;54:138-42.

85. Kountouras J, Boura P, Tsapas G. In vivo effect of granulocyte-macrophage colony-stimulating factor and interferon combination on monocyte-macrophage and T-lymphocyte functions in chronic hepatitis B leukocytopenic patients. Hepatogastroenterology **1998**;45:2295-302.

86. Weber J, Sondak VK, Scotland R, et al. Granulocyte-macrophage-colony-stimulating factor added to a multipeptide vaccine for resected Stage II melanoma. Cancer **2003**;97:186-200.

87. Spitler LE, Grossbard ML, Ernstoff MS, et al. Adjuvant therapy of stage III and IV malignant melanoma using granulocyte-macrophage colony-stimulating factor. J Clin Oncol **2000**;18:1614-21.

88. von Mehren M, Arlen P, Gulley J, et al. The influence of granulocyte macrophage colony-stimulating factor and prior chemotherapy on the immunological response to a vaccine (ALVAC-CEA B7.1) in patients with metastatic carcinoma. Clin Cancer Res **2001**;7:1181-91.

89. Gribben JG, Devereux S, Thomas NS, et al. Development of antibodies to unprotected glycosylation sites on recombinant human GM-CSF. Lancet **1990**;335:434-7.

90. Wadhwa M, Bird C, Fagerberg J, et al. Production of neutralizing granulocyte-macrophage colony-stimulating factor (GM-CSF) antibodies in carcinoma patients following GM-CSF combination therapy. Clin Exp Immunol **1996**;104:351-8.

91. Wadhwa M, Skog AL, Bird C, et al. Immunogenicity of granulocyte-macrophage colony-stimulating factor (GM- CSF) products in patients undergoing combination therapy with GM-CSF. Clin Cancer Res **1999**;5:1353-61.

92. Ragnhammar P, Friesen HJ, Frodin JE, et al. Induction of anti-recombinant human granulocyte-macrophage colony- stimulating factor (Escherichia coli-derived) antibodies and clinical effects in nonimmunocompromised patients. Blood **1994**;84:4078-87.

93. Ullenhag G, Bird C, Ragnhammar P, et al. Incidence of GM-CSF antibodies in cancer patients receiving GM-CSF for immunostimulation. Clin Immunol **2001**;99:65-74.

94. Svenson M, Hansen MB, Ross C, et al. Antibody to granulocyte-macrophage colony-stimulating factor is a dominant anti-cytokine activity in human IgG preparations. Blood **1998**;91:2054-61.

95. Wadhwa M, Meager A, Dilger P, et al. Neutralizing antibodies to granulocyte-macrophage colony-stimulating factor, interleukin-1alpha and interferon-alpha but not other cytokines in human immunoglobulin preparations. Immunology **2000**;99:113-23.

96. Kitamura T, Tanaka N, Watanabe J, et al. Idiopathic pulmonary alveolar proteinosis as an autoimmune disease with neutralizing antibody against granulocyte/macrophage colony-stimulating factor. J Exp Med **1999**;190:875-80.

97. Seymour JF, Presneill JJ, Schoch OD, et al. Therapeutic efficacy of granulocyte-macrophage colony-stimulating factor in patients with idiopathic acquired alveolar proteinosis. Am J Respir Crit Care Med **2001**;163:524-31.

98. Gilbert PB, Chiu YL, Allen M, et al. Long-term safety analysis of preventive HIV-1 vaccines evaluated in AIDS vaccine evaluation group NIAID-sponsored Phase I and II clinical trials. Vaccine **2003**;21:2933-47.

99. Toledo H, Baly A, Castro O, et al. A phase I clinical trial of a multi-epitope polypeptide TAB9 combined with Montanide ISA 720 adjuvant in non-HIV-1 infected human volunteers. Vaccine **2001**;19:4328-36.

100. Cohen GB, Islam SA, Noble MS, et al. Clonotype tracking of TCR repertoires during chronic virus infections. Virology **2002**;304:474-84.

101. Bechhofer RE, Santner TJ, Goldsman DM. Design and analysis of experiments for statistical selection, screening, and multiple comparisons. New York: John Wiley & Sons, **1995**.

102. Hudgens MG. Estimating cumulative probabilities from incomplete longitudinal binary responses with application to HIV vaccine trials. Statistics in Medicine **2003**;22:463-79.

Protocol history

The Protocol Team may modify the original version of the protocol. Modifications are made to HVTN protocols via clarification memos, letters of amendment, or full protocol amendments. HVTN protocols are modified and distributed according to the standard HVTN procedures as described in the HVTN MOP (Organization and Policy>Vaccine Selection and Protocol Development).

The table below describes the version history of, and modifications to, Protocol HVTN 060.

Protocol history and modifications

Date	Protocol version	Protocol modification	Comment
22-Mar-05	Version 1	Original protocol	

Protocol team

Information on protocol team member designation and responsibilities and on the protocol development process can be found in the HVTN MOP.

Contact information for protocol team members, HVTUs, and labs can be found in the *HVTN 060 Study Specific Procedures*.

Chair
Spyros Kalams
Vanderbilt Univ. School of Medicine
615-343-1743
spyros.a.kalams@vanderbilt.edu

Co-chair
Scott Parker
U of Alabama at Birmingham
205-976-2373
sdparker@uab.edu

Clinical trials physician
Marnie Elizaga
HVTN Core
206-667-2342
melizaga@hvtn.org

Vaccine developer representatives
Michael Lubeck
Wyeth Vaccines Research
845-602-1275
lubeckm@wyeth.com

Dagna Laufer
Wyeth Vaccines Research
484-865-2011
laugerd@wyeth.com

John Eldridge
Wyeth Vaccines Research
eldridj@wyeth.com

Laboratory program representative
Cristine Cooper
HVTN Laboratory Operations
206-667-5812
cjcooper@hvtn.org

Medical officer
Maria Allende
NIAID Vaccine Clinical Research Branch
301-435-3730
mallende@niaid.nih.gov

Pharmacist
Scharla Estep
PAB, DAIDS, NIAID
301-435-3746
sr72v@nih.gov

Statisticians
Barbara Metch
SCHARP
206-667-4656
barb.metch@scharp.org

Ya-Lin Chiu
SCHARP
206-667-6176
ychiu@scharp.org

Clinic coordinator
Katie Crumbo
Vanderbilt Univ. School of Medicine
615-343-3478
katie.crumbo@van615derbilt.edu

Community Advisory Board members
Edd Lee
Project Achieve/NY Blood Ctr
212-367-1534
edd@avac.org

Regulatory affairs
Renée Holt
SCHARP
206-667-7505
renee@scharp.org

Clinical affairs
Michael Cooney
SCHARP
206-667-7006
mcooney@scharp.org

Protocol operations manager
Drienna Holman
SCHARP
206-667-6695
dholman@scharp.org

Protocol specialist
Drienna Holman
SCHARP
206-667-6695
dholman@scharp.org

Community education
Gail Broder
HVTN Core
206-667-7348
gbroder@hvtn.org

Protocol development coordinator
Huguette Redinger
HVTN Core
206-667-7749
hredinger@hvtn.org

Technical writer
Erik Schwab
HVTN Core
206-667-7623
eschwab@hvtn.org

APPENDICES

Appendix A: Sample informed consent form

Title: A Phase I clinical trial to evaluate the safety and immunogenicity of an HIV-1 *gag* DNA vaccine with or without *IL-12* DNA adjuvant, boosted with homologous plasmids or with HIV CTL multiepitope peptide vaccine / RC529-SE plus GM-CSF, in healthy, HIV-1 uninfected adult participants

Short title: A safety study of HIV vaccines and adjuvants

Thank you for your interest in this study.

The HIV Vaccine Trials Network (HVTN) and [site] are conducting a research study (an experiment).[1]

Site: Footnotes connecting template language to CFR and ICH guidelines are intended for writers, reviewers, and IRBs/IECs. Delete before giving consent form to participant unless your site favors inclusion

This study is testing 2 experimental vaccines against HIV, the virus that causes AIDS. Vaccines are given to prevent infection or fight disease. This study is also testing 3 experimental adjuvants. An adjuvant is a substance that helps the body respond to a vaccine.

We are testing the vaccines and adjuvants to see if they are safe to give to people and well tolerated. We are also testing to see how your immune system responds to them. The immune system protects your body against infections.[2]

We are also looking at any problem you may experience because of being in this study, such as social discrimination, personal problems, or unfairness.

This study is paid for by the US National Institutes of Health (NIH). The researcher in charge of this study at this clinic is
[Insert name of the site PI].

Participation in this study is voluntary. You do not have to join.[3] If you join this study and stay in it, you will be in it for about 9 months if you are in Part A or 15 months if you are in Part B. You will be told which part of the study you will be in.[4] About 156 people will take part in this study ([#] at this clinic).[5]

This is an *informed consent* form. It answers these questions:

1. What is being tested?
2. How do I join this study?
3. What will happen during clinic visits?

[1] **21 CFR 50.25.a.1** A statement that this study involves research… **ICH 4.8.10.a** That the trial involves research.

[2] **21 CFR 50.25.a.1** …an explanation of the purposes of the research… **ICH 4.8.10.b** The purpose of the trial.

[3] **21 CFR 50.25.a.8** A statement that participation is voluntary…. **ICH 4.8.10.m** That the subject's participation in the trial is voluntary….

[4] **21 CFR 50.25.a.1** …and the expected duration of the subject's participation… **ICH 4.8.10.s** The expected duration of the subject's participation in the trial.

[5] **21 CFR 50.25.b.6** The approximate number of subjects involved in this study. **ICH 4.8.10.t** The approximate number of subjects involved in the trial.

4. What will happen to my blood samples?
5. What are the risks and inconveniences?
6. What are the benefits?
7. What are the alternatives to participating?
8. What are my responsibilities?
9. Can the researchers stop injections or take me out of this study?
10. What if I get HIV during this study?
11. What if I choose to leave this study?
12. Who makes sure this study is done correctly?
13. How will my private information be protected?
14. What if the experimental vaccine injures me or makes me sick?
15. What if the researchers learn new information during this study?
16. Will I have to pay?
17. Will I be paid?
18. Who should I call if I have questions or problems?

Read this consent form carefully. Please ask questions about anything you do not understand. The clinic staff will talk with you about the information in this form, and test your understanding. We encourage you to ask questions about this study at any time.

Site: Add the following paragraph (or one like it) if appropriate:

You may want to talk to others (such as family, friends, or your doctor) before you decide whether to join this study.

We will ask you to sign this form. Signing means:

- you have read the form (or had it explained to you),
- you understand it, and
- you agree to join this study.

We will give you a copy of this form.

1. What is being tested?[6]

The 2 vaccines are called *gag* DNA vaccine and *multi-epitope peptide* vaccine (MEP). They were developed by Wyeth Vaccines Research.

The 3 adjuvants are called *IL-12* DNA, RC529-SE and GM-CSF.

The vaccines and adjuvants in this study are made in a laboratory. The vaccines are not made from live HIV or from HIV-infected cells. They do not contain live or killed HIV. ***It is impossible to get HIV infection or AIDS from these experimental vaccines.***

The vaccines and adjuvants are experimental. They have not been approved for treating or preventing HIV infection. Except for the adjuvant GM-CSF, the US Food and Drug Administration (FDA) allows their use in research only. GM-CSF is approved by the US FDA to help restore the white blood cells of people who have been treated for cancer.

[6] **ICH 4.8.10.c** The trial treatment(s) and the probability for random assignment to each treatment.

gag DNA vaccine

DNA is a natural substance in the body that instructs the body to make proteins. Proteins are natural substances that the body uses to build and maintain itself as well as protect itself against disease.

This experimental vaccine contains a piece of DNA made in the laboratory. The DNA will instruct the body to make part of a protein called Gag that is found in HIV. Your body's immune system may respond to this protein by making cells that recognize and fight against this type of HIV protein.

IL-12 *DNA adjuvant*

The *IL-12* DNA adjuvant is DNA that will tell your body to make IL-12, a normal protein in the body that helps immune cells work together. The *IL-12* DNA adjuvant is given with the *gag* DNA vaccine to help the body's immune system respond to the vaccine.

MEP vaccine and RC529-SE adjuvant

The MEP vaccine contains a mixture of 4 peptides made in the laboratory. Peptides are small protein molecules. Your body's immune system may respond to these peptides by making cells that recognize and fight against parts of HIV that are like these peptides. The MEP vaccine is always mixed with RC529-SE, an adjuvant with man-made fat molecules mixed in oil and water. RC529-SE may help your immune system respond to the vaccine.

GM-CSF adjuvant

GM-CSF is a protein made by the body's immune system and is often given to cancer patients on chemotherapy to help restore their blood cells.

Placebo

Not everyone in this study will get an experimental vaccine. Some people will get a placebo, an inactive substance that does not contain vaccine. In this study, the placebo is sterile salt water. We give placebo to some people, and compare the results from the people who got the experimental vaccines with the results from the placebo group. This helps us measure the effects of the experimental vaccines.

Being assigned to a group

You will be assigned to get experimental vaccine or placebo at random, like the toss of a coin. You have an 83% (5 in 6) chance of getting an experimental vaccine. You have a 17% (1 in 6) chance of getting placebo. This is a double-blind study. That means that neither you nor the researchers at your clinic know which product (experimental vaccine or placebo) you are getting until after the study is over.

There are two parts in this study, Part A and Part B. Clinic staff at this site can tell you which of the groups is currently enrolling.

Part A

Part A will test the *gag* DNA vaccine alone and with the *IL-12* DNA adjuvant. Part A will have 4 groups; clinic staff will tell you which group is currently enrolling. In each group, 10 people will receive the experimental vaccine and 2 people will receive the placebo. All people in Part A will be scheduled to receive an injection (shot) at 3 separate visits.

Group 1 and 2 will begin at the same time.

Group 1 will receive 3 injections of the *gag* DNA vaccine alone, or placebo.

Group 2 will receive 3 injections of the *gag* DNA vaccine with a low dose of the *IL-12* DNA adjuvant, or placebo.

Group 3 will receive 3 injections of the *gag* DNA vaccine with a medium dose of the *IL-12* DNA adjuvant (5 times higher than the dose given to Group 2), or placebo. Group 3 will begin only if no serious safety concerns are seen in people from Groups 1 and 2 two weeks after the second vaccination.

Group 4 will receive 3 injections of the *gag* DNA vaccine with a high dose of the *IL-12* DNA adjuvant (15 times higher than the dose given to Group 2), or placebo. Group 4 will begin only if no serious safety concerns are seen in people from Group 3 two weeks after the second vaccination.

Part B

People in Part B will receive their injections only if no serious safety concerns are seen in people from Part A two weeks after their second vaccination**.** Part B will test the *gag* DNA vaccine alone and with the *IL-12* DNA adjuvant.

Part B will also test the MEP vaccine and adjuvants. Researchers will first review the results from study HVTN 056 that is also testing these products in people, to make sure that no serious safety concerns are caused by the MEP vaccine.

Part B will have 3 groups; clinic staff will tell you which group is currently enrolling. In each group 30 people will receive the experimental vaccine and 6 people will receive the placebo. All people in Part B will be scheduled to receive an injection (shot) at 5 separate visits.

Group 5 will receive 5 shots of the *gag* DNA vaccine alone, or placebo.

Group 6 will receive 5 shots of the *gag* DNA vaccine with a high dose of the *IL-12* DNA adjuvant (the same dose given to group 4), or placebo.

Group 7 will receive 3 shots of the *gag* DNA vaccine with a high dose of the *IL-12* DNA adjuvant (the same dose given to group 4), or placebo.

They will then get 2 shots of the MEP vaccine with RC529-SE adjuvant and GM-CSF, or placebo.

The following table shows the groups and the study products they get.

	Number of people	First injection	Number of months after first injection			
			1	3	6	9
**PART A**						
Group 1	10	*gag* DNA	*gag* DNA	*gag* DNA	—	—
	2	placebo	placebo	placebo	—	—
Group 2	10	*gag* DNA + low dose *IL-12* DNA	*gag* DNA + low dose *IL-12* DNA	*gag* DNA + low dose *IL-12* DNA	—	—
	2	placebo	placebo	placebo	—	—
Group 3	10	*gag* DNA + medium dose *IL-12* DNA	*gag* DNA + medium dose *IL-12* DNA	*gag* DNA + medium dose *IL-12* DNA	—	—
	2	placebo	placebo	placebo	—	—
Group 4	10	*gag* DNA + high dose *IL-12* DNA	*gag* DNA + high dose *IL-12* DNA	*gag* DNA + high dose *IL-12* DNA	—	—
	2	placebo	placebo	placebo	—	—
**PART B**						
Group 5	30	*gag* DNA	*gag* DNA	*gag* DNA	*gag* DNA	*gag* DNA
	6	placebo	placebo	placebo	placebo	placebo
Group 6	30	*gag* DNA + high dose *IL-12* DNA	*gag* DNA + high dose *IL-12* DNA	*gag* DNA + high dose *IL-12* DNA	*gag* DNA + high dose *IL-12* DNA	*gag* DNA + high dose *IL-12* DNA
	6	placebo	placebo	placebo	placebo	placebo
Group 7	30	*gag* DNA + high dose *IL-12* DNA	*gag* DNA + high dose *IL-12* DNA	*gag* DNA + high dose *IL-12* DNA	MEP*/GM-CSF	MEP*/GM-CSF
	6	placebo	placebo	placebo	placebo	placebo

*MEP is always given with the RC529-SE adjuvant.

2. How do I join this study?[7]

To see if you can take part in this study, you will have some screening procedures. Screening will include:

- questions about your medical history
- personal questions about your sexual behavior and any drug use
- physical exam

Site: In the following item, revise units of measure as appropriate

- blood tests to check for diseases such as HIV, syphilis, and hepatitis, and to check your general health; about 50 mL, or 3 tablespoons, of your blood will be drawn
- urine sample

[7] **21 CFR 50.25.a.1** …a description of the procedures to be followed… **ICH 4.8.10.d** The trial procedures to be followed, including all invasive procedures.

- pregnancy test (for women)

You may have already signed a consent form for screening. Information from the screening can only be used for 56 days (8 weeks). If you are not enrolled into the study by then, you may need to be screened again.

The results of the screening tests may show that you cannot join this study. We will explain the results to you, and tell you about places where you can get support and medical care if you need it.

If you are pregnant or breastfeeding, you cannot join this study.

If you are HIV positive, you cannot join this study. The clinic staff will counsel you about your HIV infection and about telling your partner(s). The clinic staff will tell you about places where you can get support and medical care, and about other studies you may want to join.

3. What will happen during clinic visits?[7]

Site: Give number of visits and range of visit lengths

You will visit the clinic about [#] times. The length of visits will vary from [#] to [#] hours.

If necessary, we may ask you to return to the clinic for more visits and/or lab tests.

You will be tested for HIV regularly. You will be counseled about the test and your results. You will also get regular counseling on how to reduce your risk of getting HIV.

At some visits, we will ask you questions to see if you have experienced personal problems or discrimination because of being in an HIV vaccine study. You can tell us about these problems at any time. We will also ask you personal questions about your sexual behavior and any drug use.

The following table shows what will happen at each study visit.

Part A

Procedure	Screening visit	1st injection visit	Time after 1st injection visit (in months)						
			½	1	1½	3	3½	6	9
Injection		√		√		√			
Medical history	√								
Complete physical	√								√
Brief physical		√	√	√	√	√	√	√	
Urine test	√		√		√		√		
Blood drawn	√	√	√		√		√	√	√
Pregnancy test (women)	√	√		√		√		√	
HIV testing/counseling	√						√	√	√
Interview/questionnaire	√	√	√	√	√	√	√	√	√

Not shown in this table is a time after all study participants have completed their last scheduled visit when you can find out what products you received.

Part B

Procedure	Screening visit	1st injection visit	Time after 1st injection visit (in months)										
			½	1	1½	3	3½	6	6½	9	9½	12	15
Injection		√		√		√		√		√			
Medical history	√												
Complete physical	√												√
Brief physical		√	√	√	√	√	√	√	√	√	√	√	
Urine test	√		√		√		√		√		√		
Blood drawn	√	√	√		√		√	√	√	√	√	√	√
Pregnancy test (women)	√	√		√		√		√		√		√	
HIV testing/counseling	√						√		√		√	√	√
Interview/questionnaire	√	√	√	√	√	√	√	√	√	√	√	√	√

Not shown in this table is a time after all study participants have completed their last scheduled visit when you can find out what products you received.

At each visit, we will check for any changes in your health. We will ask you how you are feeling, and if you are taking any medication. At some visits we will examine you, and ask you to give urine or blood samples. We will *not* test your blood or urine for illegal drugs.

After all participants have had their final clinic visit (or sooner if necessary), we will tell you whether you got the experimental vaccine or placebo. To do so, we may ask you to come to the clinic one more time. Not all participants join this study at the same time, so you may have to wait as much as a year after your final clinic visit to learn what you got.

Injections

If you are in Part A, you will get an injection (shot) at 3 of your clinic visits. If you are in Part B, you will get an injection at 5 of your clinic

visits. If you are a woman, you will have a pregnancy test before each injection. The injections will be given in the muscle of your upper arm by syringe and needle. Usually the injections are given in the same arm.

After each injection, you will stay in the clinic for at least 25 to 45 minutes. Clinic staff will watch you for possible reactions to the injection.

You will be asked to record your temperature and other side effects on a symptom log the evening of the injection and for the following 3 days, or longer if necessary. You will be asked to contact the clinic to report these symptoms daily. The clinic staff may ask you to return to the clinic if necessary. It is very important to stay in touch with the clinic staff.

If you have serious reactions, we may decide that you should not get any more injections. If that happens, we will ask you to return for other visits and tests, to check your health and to look for an immune response to any injections you got earlier.

Blood samples

Site: In this subsection, revise units of measure as appropriate

At some visits, we will take samples of your blood. The amount will depend on the lab tests we need to do. It will be some amount between 20mL and 450mL (4 teaspoons to about 2 cups). The total amount of blood taken from you during this study will be no more than 1365 mL (about 5¾ cups) in Part A and no more than 2490 mL (about 10½ cups) in Part B. To compare, people who donate blood can give about 500mL (about 2 cups) every 8 weeks.

4. What will happen to my blood samples?

Use in this study

We will use some of your blood for routine safety testing, to check your health and see if you have side effects. We will tell you the results of routine lab tests at your next visit, or sooner if necessary.

We will use some of your blood to test your immune response to the experimental vaccines or placebo.

In addition, a genetic test called *HLA typing* may be performed. HLA stands for human leukocyte antigen—a tiny marker on your cells that helps protect the body from infections. You inherited your HLA type from your father and mother. We think that people with different HLA types may respond differently to the experimental vaccine. If HLA typing is done on your blood, this will not affect your participation in this study.

Tests of immune response are for HIV-related or vaccine-related research only (not to check your health), so we will not tell you or the clinic the results.

Site: Per HVTN policy, the following passage must be retained verbatim:

Storage and future testing

We will store other samples of your blood for future research that is not part of this study. This may include genetic testing. Your samples would be used for HIV-related or vaccine-related research only. We may also test your immune response to other vaccines you may have received, or other illnesses that you may have been exposed to in the past. An Institutional Review Board or Independent Ethics Committee, which watches over the safety and rights of research participants, must approve any research studies using your samples. Your samples will not be sold.

Your samples may contribute to a new invention or discovery. There is no plan for you to share in any money or other benefits resulting from this invention or discovery.

Your samples will be stored indefinitely. Please do not participate in this study if you do not wish to have your samples stored for future research.

The researchers do not plan to contact you or your health care provider with results from future studies using your blood. This is because the procedures in research are often experimental. If the researchers decide that a specific test result would provide important information for your health, we will try to contact you. If you want this information, tell the clinic staff. Always let the study clinic know if you change your address and/or phone number.

5. What are the risks and inconveniences?[8]

Being in this study may harm you. It also restricts your behavior; you may find the restrictions inconvenient.

This section describes the risks and restrictions we know about. There may be unknown risks, even serious ones. These unknown risks could affect you, or your fetus if you become pregnant. If we learn about new risks during this study, we will tell you.[9]

Risks of injections

Injections can cause pain, soreness, redness, and swelling on the part of your body where you got the injection. On rare occasions, they may cause bacterial infection at the part of your body where you got the injection.

[8] **21 CFR 50.25.a.2** A description of any reasonably foreseeable risks or discomforts to the subject. **21 CFR 50.25.b.1** A statement that the particular treatment or procedure may involve risks to the subject (or to the embryo or fetus, if the subject is or may become pregnant) which are currently unforeseeable. **ICH 4.8.10.g** The reasonably foreseeable risks or inconveniences to the subject and, when applicable, to an embryo, fetus, or nursing infant.

[9] **ICH 4.8.10.p** That the subject or the subject's legally acceptable representative will be informed in a timely manner if information becomes available that may be relevant to the subject's willingness to continue participation in the trial. **21 CFR 50.25.b.5** A statement that significant new findings developed during the course of the research which may relate to the subject's willingness to continue participation will be provided to the subject.

Risks of the vaccine or placebo

Vaccines can cause fever, chills, rash, aches and pains, muscle aches, muscle damage, nausea, headache, dizziness, and fatigue.

We do not know if these experimental vaccines will change your response to an approved HIV vaccine if you receive one in the future. Currently, there is no approved and licensed HIV vaccine. If such a vaccine becomes available in the future, we do not know whether getting the experimental vaccines in this study will cause your body to respond differently to a licensed vaccine, altering your body's ability to prevent HIV infection and disease. Your body's ability to prevent HIV infection and AIDS may become better or worse, or stay the same

Allergic reaction

You could have an allergic reaction to any of the study products, like a rash, hives, or even difficulty breathing. *Allergic reactions can be life threatening.* The clinic staff will watch you for at least 25 to 45 minutes after each injection (the time when most allergic reactions occur) and give you treatment if you need it. People with a known allergy to bupivacaine (Marcaine), or related local anesthetics (like Lidocaine (Xylocaine), Mepivacaine (Polocaine/Carbocaine), Etidocaine (Duranest), or Prilocaine) cannot participate in this study. People with a known allergy to eggs or yeast cannot participate in Part B of this study.

General risks of DNA vaccines

Possible risks related to DNA vaccines include: muscle damage at the site of the injection, the production of antibodies which might react with normal body tissues and cause an autoimmune disease, and insertion of the vaccine DNA into the body's DNA. This could lead to cancer or unknown side effects. Although these risks are possible, only muscle damage at the site of injection has been seen so far in animals or humans. Other HIV DNA vaccines have also been given to humans since 1995. These vaccines have not caused serious side effects, although we do not yet have long-term safety information about the people in these studies.

Risks of gag DNA vaccine

The *gag* DNA vaccine in this study has not been given to humans before, so we do not know all of the risks or side effects. However, DNA *gag* vaccines similar to this one have been tested in several hundred humans with no known serious side effects. The *gag* DNA vaccine in this study was tested in mice and rabbits with no serious side effects. However, animal tests may not show what will happen in humans.

Risks of IL-12 DNA adjuvant

The *IL-12* DNA adjuvant in this study has not been given to humans before, so we do not know all the risks or side effects. A similar *IL-12*

DNA vaccine adjuvant has been tested in animals with no serious side effects. However, animal tests may not show what will happen in humans.

IL-12, in the form of a protein known as recombinant human IL-12, has been tested in many studies with both healthy people and patients with infectious diseases. In healthy people, the most frequently reported side effects included fever, headache, muscle aches, nausea, chills, weakness, fatigue, and reactions at the injection site. Some patients had changes in test results for blood sugar, liver activity, and white blood cell count (cells that fight infection). These changes were temporary and test results returned to normal without treatment. On rare occasions, after receiving high doses of recombinant human IL-12, people have reported anxiety, confusion, depression, gastrointestinal bleeding, kidney problems, an abnormal feeling of burning or tingling, and increased blood pressure. The recombinant human IL-12 protein is not the same as the *IL-12* DNA adjuvant used in this study. Also, the *IL-12* DNA adjuvant is being given at a much lower dose.

Risks of gag DNA and IL-12 DNA adjuvant given together

Since this combination of *gag* DNA vaccine and *IL-12* DNA has not been given to humans before this study, all possible risks or side effects are currently not known. Animals have had the vaccine and adjuvant together in doses similar to or larger than those planned to be given in this study without problems. However, animal tests may not show what will happen in humans.

Risks of GM-CSF adjuvant

GM-CSF has been used as an adjuvant in trials with hundreds of healthy people and no serious side effects have been seen. It has also been tested in HVTN 056.

GM-CSF can cause a temporary increase in white blood cells. If this happens to you, it may become difficult to tell if you have a true infection, or if this is a response to the GM-CSF.

GM-CSF has been given to thousands of cancer patients to help restore their blood cells. In cancer patients, GM-CSF can cause water retention, shortness of breath, a fast heart rate, fever, headache, bone pain, body aches, rash, chills, fatigue, increased white blood cells (blood cells that fight infection) and platelets (cells in the blood that help stop bleeding) and overall fatigue and weakness. When given in high doses over a long time, GM-CSF has become less effective in some cancer patients. We do not know if use of GM-CSF in this study will have the same effect, if you ever need this treatment in the future.

Risks of MEP vaccine, RC529-SE and GM-CSF given together

HVTN 056, which uses these products together, is in progress. In that study 40 people are getting 3 injections of the vaccine without the GM-

CSF adjuvant and 40 more are getting 3 injections of the same vaccine with the GM-CSF adjuvant. Sixteen people are receiving placebo. So far, 52 people have received their first injection, and 24 have received all 3 injections of vaccine or placebo. In the first few days after injection, most participants have experienced mild to moderate pain or tenderness (or both) at the injection site. About half of the participants have experienced mild fatigue or have felt unwell. In a few cases, the side effects, such as fatigue, injection site pain or tenderness, were severe enough to interfere with normal daily activities. Some participants have missed work for a day. In most people these side effects improved to mild or went away within a few days. In a few people the symptoms lasted a few days longer. We do not know if this study will see similar results.

The MEP vaccine with the RC529-SE adjuvant and GM-CSF has also been tested in rabbits with no serious side effects. Animal testing does not always show what will happen in people.

Since the RC529-SE is made with lecithin, a product which comes from eggs, one known risk is an allergic reaction for people who are allergic to eggs.

Risks of bupivacaine administration

The DNA vaccine contains bupivacaine. Bupivacaine helps the DNA vaccine get into the muscle. It is an anesthetic similar to the numbing medicine used by dentists. When used in doses greater than 50 times higher than in this study, bupivacaine can cause problems with the nervous system and heart. The nervous system side effects can be confusion, anxiety, dizziness, blurred vision, shaking, or seizures. The heart side effects can be decreased heart pumping, fast heart rate, low blood pressure, abnormal heart beats, or even death. Other possible side effects include nausea, vomiting, or chills. All of these side effects are very rare, but may happen as a result of accidental injection of the vaccine into the bloodstream, or a slow breakdown of the drug.

Risks of all the study products given together

These 5 experimental products have never been tested together in humans or animals before. This study will test if they are safe when given together.

Blood drawing

Drawing blood may cause pain and bruising. On rare occasions, it may cause bacterial infection at the part of your body where the blood is taken. Sometimes, drawing blood causes people to feel lightheaded or to faint.

Personal problems

Some participants in other HIV vaccine studies have reported experiencing personal problems because of their participation. Spouses, other family members, or sexual partners have sometimes reacted by:

- becoming angry when a participant joined a study without consulting them
- worrying that the test vaccine would be harmful
- assuming that the participant was infected with HIV and shunning them
- assuming that the participant is engaging in certain sexual activities or drug use, and treating them unfairly

On rare occasions, a participant has reported losing a job because of being in an HIV vaccine study. This was either because the study took too much time away from work, or because the employer thought the participant was HIV infected or at high risk for HIV.

If genetic testing (such as HLA typing) is performed on your blood, there is a very small chance that the results will cause personal problems. Information from this test may suggest you are at risk for certain diseases. This does not mean you will get a disease, but if your test results were known, you could have trouble getting insurance or a job. This risk is extremely small, because the test results do not identify you by name. They do not become part of your medical records.

Clinic staff will help you with personal problems you may experience because of being in this study.

HIV exposure

If you are exposed to HIV at some time after getting an experimental vaccine, we do not know what will happen. The experimental vaccine could have a positive or negative effect, or no effect, on:

- your risk of becoming infected with HIV if exposed
- the time it takes to develop AIDS after being infected
- the course of HIV infection

We do not know if getting the experimental vaccine will protect you from HIV. This study will not answer that question. In the past, some people have become infected with HIV even though they got an experimental vaccine. The experimental vaccine did not cause the HIV infection, but did not prevent infection in these cases. Because we do not know the effect of the experimental vaccine, *we ask you not to do anything that may expose you to HIV, like having unprotected sex or sharing needles or injection equipment.*

False positive HIV test

Standard HIV tests look for antibodies (made by cells in your immune system) that recognize HIV. An experimental vaccine may cause your body to produce these antibodies. In this case, the standard HIV test could show a positive result. This does not mean you are infected with HIV—the

test result could be a *false positive*. The risk of a false positive test is very low with these study products.

If this happens, we will do further tests to confirm that you are not infected with HIV. If the experimental vaccine caused the false positive result, we do not know how long the HIV test will stay positive. We will offer retesting free of charge as long as the positive HIV test is due to the experimental vaccine.

If you are tested for HIV outside this study, a false positive result may cause you trouble. You may have trouble with:

- insurance
- medical/dental care
- travel to other countries
- employment
- military service

Blood banks and medical institutions know that an experimental vaccine may give a false positive result. Still, if you continue to have a false positive result after the study you will have trouble donating blood, body fluids, or body tissues. You may even be permanently banned from donating.

To help with these situations, or to prevent discrimination, we can talk to insurance companies, employers, and others to explain that you are in a study. We would do this only at your request and with your written permission.

Restrictions on your behavior

While you are in this study, there are restrictions on your behavior.

- Because of the risk of a false positive HIV test, you should get your HIV testing done only at this clinic. If you have to be tested for HIV outside this study, please talk to the clinic staff.
- You must not donate blood, body tissues, or body fluids.
- You may not be able to join other medical research studies. If you are thinking about joining another study, please talk to the clinic staff.
- After you are finished with this study, you may not be able to join other HIV vaccine studies.

We ask you not to do anything that may expose you to HIV, like having unprotected sex or sharing needles or injection equipment.

Pregnancy risks

Women should not become pregnant during this study (about 9 months if you are in Part A and 15 months if you are in Part B), because we do not know how the experimental vaccines or adjuvants could affect the fetus. If you are a woman having sex that could lead to pregnancy, you must agree to use effective birth control, starting at least 21 days prior to enrollment and continuing until after your last clinic visit. This means using any of the following methods:

- Birth control drugs that prevent pregnancy—given by pills, shots, patches, vaginal rings, or inserts under the skin
- Male or female condoms, with or without a cream or gel that kills sperm
- Diaphragm or cervical cap with a cream or gel that kills sperm
- Intrauterine device (IUD)
- Any other contraceptive method approved by the researchers

6. What are the benefits?

This study may not benefit you personally. Being in this study may help in the search for an HIV vaccine.[10]

Site: Add information about other benefits (health care, tests, etc.) as appropriate

7. What are the alternatives to participating?

You may choose not to join this study. Other services you receive at this institution will not be affected.[11]

If you choose not to join this study, you may join a different experimental HIV vaccine study, if one is available and you are eligible.[12]

8. What are my responsibilities?[13]

If you join this study, you will be asked to:

- come to all clinic visits
- record your temperature and other side effects on the symptom log the evening of the injection and for the following 3 days, or longer if necessary
- tell clinic staff about any symptoms or side effects you have

[10] **21 CFR 50.25.a.3** A description of any benefits to the subject or to others which may reasonably be expected from the research. **ICH 4.8.10.h** The reasonably expected benefits. When there is no intended clinical benefit to the subject, the subject should be made aware of this.

[11] **21 CFR 50.25.a.8** …that refusal to participate will involve no penalty or loss of benefits to which the subject is otherwise entitled…. **ICH 4.8.10.m** …that the subject may refuse to participate…without penalty or loss of benefits to which the subject is otherwise entitled.

[12] **21 CFR 50.25.a.4** A disclosure of appropriate alternative procedures or courses of treatment, if any, that might be advantageous to the subject. **ICH 4.8.10.i** The alternative procedure(s) or course(s) of treatment that may be available to the subject, and their important potential benefits and risks.

[13] **ICH 4.8.10.e** The subject's responsibilities.

- tell clinic staff about any medications you are taking
- tell clinic staff before getting any other vaccines, such as a flu shot
- follow instructions from the clinic staff
- stay in touch with the clinic staff; tell them if you have moved or if you want to leave the study.
- get your HIV testing done only at the clinic
- women: avoid pregnancy until after your last clinic visit

The clinic staff will share the HVTN Participant's Bill of Rights and Responsibilities with you. That document tells more about your rights and responsibilities.

9. Can the researchers stop injections or take me out of this study?

Your injections may be stopped if:

- getting injections would be harmful to you
- you become pregnant
- you need a treatment, and the treatment and the experimental vaccine might interfere with each other
- the experimental vaccine or placebo is no longer available

If you must stop getting injections before this study is over, we will ask you to come back to the clinic to check your health and your immune response.

If you become pregnant during the trial, we will stop your study injections. We will ask you to stay in the trial so that we can monitor your safety. If it does not cause you problems, we will want to continue to take blood from you to do the laboratory tests we describe in this consent form. If you leave the trial or the trial ends before you deliver the baby, we will ask to contact you to know the outcome of the pregnancy.

You may be taken out of this study entirely if:

- you cannot or do not attend the study visits
- you do not follow instructions
- you get infected with HIV
- the study is canceled[14]

[14] **21 CFR 50.25.b.2** Anticipated circumstances under which the subject's participation may be terminated by the investigator without regard to the subject's consent. **ICH 4.8.10.r** The foreseeable circumstances and/or reasons under which the subject's participation in the trial may be terminated.

10. What if I get HIV during this study?

If you get infected with HIV during this study, the clinic staff will do additional HIV testing to confirm the infection and learn more about it. You will not be able to stay in this study.

Site: Include required reporting information as needed

The clinic staff will counsel you about your HIV infection and about telling your partner(s). Medical care and treatment for HIV infection are not a part of this study. The clinic staff will tell you about places where you can get support and medical care, and about other studies you may want to join.

11. What if I choose to leave this study?[15]

If you join this study, you can leave it at any time. If you leave this study, you will not lose any benefits or rights you would normally have or be disadvantaged in any way.[16]

If you decide to leave this study, please tell the clinic staff. We will ask you to come back to the clinic at least once, to check your health and look for an immune response to the study products.

Like everyone else in this study, you will have to wait until all participants complete their final study visit to find out whether you got the experimental vaccine or the placebo.

12. Who makes sure this study is done correctly?

Several groups watch over this study to see that your rights are protected and that the researchers are following this study plan.[17] These groups include:

Site: Modify list for non-US monitors and IBC as appropriate

- the US National Institutes of Health (NIH)
- the US Food and Drug Administration (FDA)
- a local Institutional Review Board or Independent Ethics Committee
- Wyeth Vaccines Research and people who work for them
- the HIV Vaccine Trials Network and people who work for them
- [Insert name of local regulatory authority as appropriate]

[15] **21 CFR 50.25.b.4** The consequences of a subject's decision to withdraw from the research and procedures for orderly termination of participation by the subject.

[16] **21 CFR 50.25.a.8** …that the subject may discontinue participation at any time without penalty or loss of benefits to which the subject is otherwise entitled. **ICH 4.8.10.m** …the subject may…withdraw from the trial, at any time, without penalty or loss of benefits to which the subject is otherwise entitled.

[17] **ICH 4.8.10.n** That the monitor(s), the auditor(s), the IRB/IEC, and the regulatory authority(ies) will be granted direct access to the subject's original medical records for verification of clinical trial procedures and/or data, without violating the confidentiality of the subject, to the extent permitted by the applicable laws and regulations and that, by signing a written informed consent form, the subject or the subject's legally acceptable representative is authorizing such access.

A local Community Advisory Board is also involved in this study. Community Advisory Boards assist scientists in developing research studies and review these studies for issues important to the community. The Community Advisory Board will not have access to medical information that can identify you.

13. How will my private information be protected?[18]

US sites: Check HIPAA authorization for conflicts with this section

We will do our best to protect your private information. Your records are kept in locked files at the clinic. On most records, we use a participant ID number, not your name.

The results of this study may be published. Any publication will not use your name or identify you personally.

Most of the groups who watch over this study may review your medical records. Your records may also be reviewed by clinic staff, and by the vaccine developer or people who work for them. Reviewers will keep your records private.

Samples of your blood or other fluids are stored in a secure central storage site (not the clinic). Your name is not on the samples. The label on each sample tube contains only 4 pieces of information: a participant ID number, the substance in the tube, a visit number, and a visit date.

The results of tests for immune response, including genetic tests, are confidential. They do not identify you by name. They are not part of your medical records.

We cannot guarantee absolute privacy. Information about you may be released if required by law.

[18] **21 CFR 50.25.a.5** A statement describing the extent, if any, to which confidentiality of records identifying the subject will be maintained and that notes the possibility that the Food and Drug Administration may inspect the records. **ICH 4.8.10.o** That records identifying the subject will be kept confidential and, to the extent permitted by the applicable laws and/or regulations, will not be made publicly available. If the results of the trial are published, the subject's identity will remain confidential.

Site: insert local legal requirements here.

Site: If this study is being done at a US site where a Certificate of Confidentiality does apply, include the following 3 paragraphs verbatim. Bulleted examples should include all appropriate cases (reportable communicable disease, risk of harm to self or others, etc.)

To help us protect your privacy, the US government has given us a Certificate of Confidentiality.[19] The certificate means that the researchers cannot be forced to tell people who are not connected with this study that you are in it. If you would like to read the certificate, ask the clinic staff. We will use the certificate to refuse to give information that may identify you, even in court proceedings.

Sometimes the certificate cannot be used. For example, if someone from the US government wants to review projects that the government pays for, we cannot withhold information. We also must cooperate to meet the requirements of the US Food and Drug Administration (FDA).

Sometimes we may have to release information about you without your permission. For example, we may do this if:

- you have a disease that we must report to the health department, such as certain sexually transmitted infections
- we suspect that you may be harming yourself or others or planning to do so

14. What if my participation in the study makes me sick or injures me?[20]

If you get sick or injured, tell the clinic staff immediately. The clinic staff will treat you for study-related problems or tell you where you can get more treatment if you need it.

The cost of treatment of problems related to receiving vaccine (or placebo) will be covered by Wyeth Vaccines Research (the vaccine developer) or by the clinical trial site from the funds that support this clinical trial.

No funds are available from the clinical trial sites, the US NIH, or the HVTN to provide compensation for nonphysical injury such as lost work or pain and suffering. You and/or your health insurance carrier will continue to be responsible for costs for your medical care outside this study or for medical expenses determined not directly related to study

[19] **http://grants.nih.gov/grants/policy/coc/background.htm** Under section 301(d) of the Public Health Service Act (42 U.S.C. 241(d)) the Secretary of Health and Human Services may authorize persons engaged in biomedical, behavioral, clinical, or other research to protect the privacy of individuals who are the subjects of that research. This authority has been delegated to the National Institutes of Health (NIH). Persons authorized by the NIH to protect the privacy of research subjects may not be compelled in any Federal, State, or local civil, criminal, administrative, legislative, or other proceedings to identify them by name or other identifying characteristic.

[20] **21 CFR 50.25.a.6** For research involving more than minimal risk, an explanation as to whether any compensation and an explanation as to whether any medical treatments are available if injury occurs and, if so, what they consist of, or where further information may be obtained. **ICH 4.8.10.j** The compensation and/or treatment available to the subject in the event of trial-related injury.

procedures or products. You will not be giving up any of your legal rights by signing this consent form.

15. What if the researchers learn new information during this study?

Results of this study or other scientific research may affect your willingness to continue to participate in this study.[21] If we learn new information of this kind, we will share it with you.

16. Will I have to pay?

You do not have to pay for the study products, research clinic visits, examinations, or laboratory tests that are part of this study.[22]

17. Will I be paid?

Site: Explain what is paid for. Example:

You will receive $[#] for each visit you complete, to cover the cost of [Insert text].[23]

18. Who should I call if I have questions or problems?[24]

If you have questions about this study, contact
[name and telephone number of the investigator or other study staff].

If you have any symptoms that you think may be related to this study, contact
[name and telephone number of the investigator or other study staff].

If you have questions about your rights as a research participant, or problems or concerns about how you are being treated in this study, contact
[name/title/phone of person on IRB or other appropriate organization].

If you want to leave this study, contact
[name and telephone number of the investigator or other study staff].

[21] **21 CFR 50.25.b.5** A statement that significant new findings developed during the course of the research which may relate to the subject's willingness to continue participation will be provided to the subject. **ICH 4.8.10.p** That the subject or the subject's legally acceptable representative will be informed in a timely manner if information becomes available that may be relevant to the subject's willingness to continue participation in the trial.

[22] **21 CFR 50.25.b.3** Any additional costs to the subject that may result from participation in the research. **ICH 4.8.10.l** The anticipated expenses, if any, to the subject for participating in the trial.

[23] **ICH 4.8.10.k** The anticipated prorated payment, if any, to the subject for participating in the trial.

[24] **21 CFR 50.25.a.7** An explanation of whom to contact for answers to pertinent questions about the research and research subjects' rights, and whom to contact in the event of a research-related injury to the subject. **ICH 4.8.10.q** The person(s) to contact for further information regarding the trial and the rights of trial subjects, and whom to contact in the event of trial-related injury.

If you have read this consent form (or had it explained to you), understand it, and agree to take part in this study, please sign your name below.

Participant's name (print)	Participant's signature and date	Time (if signed on date of enrollment)

Study staff conducting consent discussion (print)	Study staff signature and date	Time (if signed on date of enrollment)

Site: For participants unable to read or write, substitute the signature block below:

Participant's name (print)	Participant's mark	Date	Time (if signed on date of enrollment)

Study staff conducting consent discussion (print)	Study staff signature and date	Time (if signed on date of enrollment)

Witness's name (print)	Witness's signature and date	Time (if signed on date of enrollment)

Appendix B: Laboratory procedures for Part A

				Tube volume (mL)										
			Visit:	**1**	**2**	**3**	**4**	**5**	**6**	**7**	**8**	**9**	**10**	
			Day:	Screening visit	D0	D14	D28	D42	D84	D98	D168	D182	D273	
			Month:		M0	M0.5	M1	M1.5	M3	M3.5	M6	M6.5	M9	
Procedure	Ship to	Assay location	Tube		**VAC1**		**VAC2**		**VAC3**					**Total**
Blood Collection														
Screening or diagnostic assays														
Screening HIV test	Local	Local	SST	5	—	—	—	—	—	—	—	—	—	**5**
HIV diagnostic ELISA	Richmond/Local	Richmond/Local	SST	—	—	—	—	—	—	5	5	—	5	**15**
HIV RNA PCR	Richmond/Local	Richmond/Local	PPT	—	5	—	—	—	—	5	5	—	5	**20**
HIV DNA PCR	Richmond/Local	Richmond/Local	ACD	—	10	—	—	—	—	10	10	—	10	**40**
HBsAg/anti-HCV/Syphilis	Local	Local	SST	5	—	—	—	—	—	—	—	—	—	**5**
Safety labs														
CBC/ Diff/ platelets	Local	Local	EDTA	5	—	5	—	5	—	5	—	—	5	**25**
Chemistry panel	Local	Local	SST	5	—	5	—	5	—	5	—	—	5	**25**
T cell subsets	Local	Local	EDTA	5	—	5	—	—	—	5	—	—	5	**20**
Immunogenicity assays														
HLA typing	CSR	Duke	ACD	—	20	—	—	—	—	—	—	—	—	**20**
Humoral assay														
Ab to IL-12	CSR	Wyeth	SST	—	5	5	—	5	—	*5*	—	—	5	**25**
Cellular assays														
ELISpot	CSR	Duke	Na Hep	—	60		—	—	—	*60*	—	—	60	**180**
ICS	CSR	FHCRC	Na Hep	—	40	—	—	—	—	*40*	—	—	40	**120**
Tetramer	CSR	Wyeth	Na Hep	—	60	—	—	—	—	*60*	—	—	60	**180**
Specimen storage														
PBMC	CSR		Na Hep	—	80	—	—	80	—	80	160	—	160	**560**
Serum	CSR		SST	—	10	—	—	10	—	10	15	—	15	**60**
**Total**				**25**	**290**	**20**	**0**	**105**	**0**	**290**	**195**	**0**	**375**	**1300**
**56-Day total**				**25**	**315**	**335**	**335**	**440**	**105**	**395**	**195**	**195**	**375**	
Urine Collection														
Urinalysis				X	—	X	—	X	—	X	—	—	—	
Pregnancy test				X	X	—	X	—	X	—	X	—	—	

Richmond = Viral and Rickettsial Disease Laboratory, California Department of Health Services (Richmond, California, USA); Duke = Duke University Medical Center (Durham, North Carolina, USA); FHCRC/UW = Fred Hutchinson Cancer Research Center/University of Washington (Seattle, Washington, USA); CSR = Central Specimen Repository

Screening may occur over the course of several contacts/visits up to and including Day 0 prior to vaccination. Additional tests performed at screening include syphilis, HepB and HepC serologies using serum samples.

Local labs may perform HIV diagnostic algorithm (following HVTN SOP) upon approval from the HVTN Laboratory Operations Division.

For viral assays, samples are sent to Richmond; test to be performed if clinically indicated. Non-US sites may use local labs with pre-approval from the HVTN Laboratory Operations Division.

Local labs may assign other tube types for HIV diagnostics ELISA, viral assays and safety labs.

Chemistry panels are defined in Table 12-1 (pre-enrollment) and Table 12-2 (post-enrollment).

Part A immunogenicity assays will be performed retrospectively at the discretion of the Protocol Chair and the Laboratory Program Director

Based on the number of responders observed at the primary immunogenicity timepoint (in *italic* in Visit 7), humoral and cellular assays may be performed on all participants including Visit 9.

Molecular HLA typing may be performed on enrolled participants using cryopreserved PBMC collected at baseline, initially in participants who demonstrate vaccine-induced T-cell responses at post-vaccination timepoints.

Appendix C: Laboratory procedures for Part B

				Tube volume (mL)													
				Visit													
			Visit:	**1**	**2**	**3**	**4**	**5**	**6**	**7**	**8**	**9**	**10**	**11**	**12**	**13**	
			Day:	Screening visit	D0	D14	D28	D42	D84	D98	D168	D182	D273	D288	D364	D455	
			Month:		M0	M0.5	M1	M1.5	M3	M3.5	M6	M6.5	M9	M9.5	M12	M15	
Procedure	Ship to	Assay location	Tube		**VAC1**		**VAC2**		**VAC3**		**VAC4**		**VAC5**				**Total**
Blood Collection																	
Screening or diagnostic assays																	
Screening HIV test	Local	Local	SST	5	—	—	—	—	—	—	—	—	—	—	—	—	**5**
HIV diagnostic ELISA	Richmond/Local	Richmond/Local	SST	—	—	—	—	—	—	5	—	5	—	5	5	5	**25**
HIV RNA PCR	Richmond/Local	Richmond/Local	PPT	—	5	—	—	—	—	5	—	5	—	5	5	5	**30**
HIV DNA PCR	Richmond/Local	Richmond/Local	ACD	—	10	—	—	—	—	10	—	10	—	10	10	10	**60**
HBsAg/anti-HCV	Local	Local	SST	5	—	—	—	—	—	—	—	—	—	—	—	—	**5**
Safety labs																	
CBC/ Diff/ platelets	Local	Local	EDTA	5	—	5	—	5	—	5	—	5	—	5	5	5	**40**
Chemistry panel	Local	Local	SST	5	—	5	—	5	—	5	—	5	—	5	5	5	**40**
T cell subsets	Local	Local	EDTA	5	—	5	—	—	—	5	—	—	—	5	—	5	**25**
Immunogenicity assays																	
HLA typing	CSR	Duke	ACD	—	20	—	—	—	—	—	—	—	—	—	—	—	**20**
Humoral assay																	
HIV Binding ELISA	CSR	Duke	SST	—	5	—	—	—	—	—	—	5	—	*5*	—	5	**20**
Ab to GM-CSF	CSR	Wyeth	SST	—	5	—	—	—	—	—	—	5	—	*5*	—	5	**20**
Ab to IL-12	CSR	Wyeth	SST	—	5	5	—	5	—	5	—	—	—	—	—	5	**25**
Cellular assays																	
ELISpot	CSR	Duke	Na Hep	—	60		—	—	—	60	—	—	—	*60*	—	60	**240**
ICS	CSR	FHCRC	Na Hep	—	40	—	—	—	—	40	—	—	—	*40*	—	40	**160**
Tetramer	CSR	Wyeth	Na Hep	—	60	—	—	—	—	60	—	60	—	*60*	—	60	**300**
Tcell repertoire studies	CSR	Vanderbilt	Na Hep	—	—	—	—	—	—	50	—	50	—	*50*	50	50	**250**
Specimen storage																	
PBMC	CSR		Na Hep	—	80	—	—	80	—	90	90	160	100	100	160	160	**1020**
Serum	CSR		SST	—	10	—	—	10	—	10	15	15	15	15	15	15	**120**
**Total**				**25**	**300**	**20**	**0**	**105**	**0**	**350**	**105**	**325**	**115**	**370**	**255**	**435**	**2405**
**56-Day total**				**25**	**325**	**345**	**345**	**450**	**105**	**455**	**105**	**430**	**115**	**485**	**255**	**435**	
Urine Collection																	
Urinalysis				X	—	X	—	X	—	X	—	X	—	X	—	—	
Pregnancy test				X	X	—	X	—	X	—	X	—	X	—	X	—	

Richmond = Viral and Rickettsial Disease Laboratory, California Department of Health Services (Richmond, California, USA); Duke = Duke University Medical Center (Durham, North Carolina, USA); FHCRC/UW = Fred Hutchinson Cancer Research Center/University of Washington (Seattle, Washington, USA); CSR = Central Specimen Repository

Screening may occur over the course of several contacts/visits up to and including Day 0 prior to vaccination. Additional tests performed at screening include syphilis, HepB and HepC serologies using serum samples.

Local labs may perform HIV diagnostic algorithm (following HVTN SOP) upon approval from the HVTN Laboratory Operations Division.

For viral assays, samples are sent to Richmond; test to be performed if clinically indicated. Non-US sites may use local labs with pre-approval from the HVTN Laboratory Operations Division.

Local labs may assign appropriate alternative tube types for locally performed tests.

Chemistry panels are defined in Table 12-1 (pre-enrollment) and Table 12-2 (post-enrollment).

Molecular HLA typing may be performed on enrolled participants using cryopreserved PBMC collected at baseline, initially in participants who demonstrate vaccine-induced T-cell responses at post-vaccination timepoints.

Based on the number of responders observed at the primary immunogenicity timepoint (in *italic* in Visit 11), humoral and cellular assays may be performed on all participants, including Visits 7 and 13.

Appendix D: Procedures at HVTU

		Visit													
	Visit:	**01[a]**	**02**	**03**	**04**	**05**	**06**	**07**	**08**	**09**	**10**	**11**	**12**	**13**	**Post**
	Day:	Screening	D0	D14	D28	D42	D84	D70	D168	D182	D273	D287	D364	D455	
	Month:		M0	M.5	M1	M1.5	M3	M3.5	M6	M6.5	M9	M9.5	M12	M15	
	Part A:		**VAC1**		**VAC2**		**VAC3**								
Procedure	Part B:		**VAC1**		**VAC2**		**VAC3**		**VAC4**		**VAC5**				
**Study procedures**															
Signed screening consent (if used)		X	–	–	–	–	–	–	–	–	–	–	–	–	–
Assessment of understanding		X	–	–	–	–	–	–	–	–	–	–	–	–	–
Signed protocol consent		X	–	–	–	–	–	–	–	–	–	–	–	–	–
Medical history		X	–	–	–	–	–	–	–	–	–	–	–	–	–
Complete physical exam		X	–	–	–	–	–	–	–	–	A	–	–	B	–
Obtain demographics		X	–	–	–	–	–	–	–	–	–	–	–	–	–
Confirm eligibility		X	–	–	–	–	–	–	–	–	–	–	–	–	–
Randomize participant		X	–	–	–	–	–	–	–	–	–	–	–	–	–
Abbreviated physical exam		–	X	X	X	X	X	X	X	B	B	B	B	–	–
Risk reduction/pregnancy prevention counseling		X	X	X	X	X	X	X	X	B	X	B	B	B	–
Behavioral risk assessment		X	–	–	–	–	–	–	–	–	–	–	–	–	–
Social impact assessment		–	–	–	–	–	X	–	X	–	A	–	–	B	–
Outside testing and belief questionnaire		–	–	–	–	–	X	–	–	–	A	–	–	B	–
Concomitant medications		X	X	X	X	X	X	X	X	B	X	B	B	B	–
Intercurrent illness / adverse experience			X	X	X	X	X	X	X	B	X	B	B	B	–
HIV infection assessment/results[b]		X	–	–	–	–	–	X	A	B	A	B	B	B	–
**Local lab assessment**															
Pregnancy (urine or serum HCG)[c]		X	X	–	X	–	X	–	X	–	B	–	B	–	–
Urine dipstick		X	–	X	–	X	–	X	–	B	–	B	–	–	–
CBC, differential, platelet[d]		X	–	X	–	X	–	X	–	B	A	B	B	B	–
Chemistry panel (see Table 12-1 and Table 12-2)[d]		X	–	X	–	X	–	X	–	B	A	B	B	B	–
RPR, Hepatitis B, Hepatitis C		X	–	–	–	–	–	–	–	–	–	–	–	–	–
T cell subsets		X	–	X	–	–	–	X	–	–	A	B	–	B	–
**Vaccination procedures**															
Vaccination		–	X	–	X	–	X	–	B	–	B	–	–	–	–
Reactogenicity assessments[e]		–	X	–	X	–	X	–	B	–	B	–	–	–	–
**Post-study**															
Unblind participant		–	–	–	–	–	–	–	–	–	–	–	–	–	X

"X" denotes procedure is to be performed in both parts of study. "A" denotes procedure is to be performed in Part A only. "B" denotes procedure is to be performed in Part B only.

[a] Screening may occur over the course of several contacts/visits up to and including Day 0 prior to vaccination.

[b] Includes pre- and post-test counseling and follow-up contact to report results to participant.

[c] For female participants, pregnancy test must be performed on the day of vaccination prior to vaccination. Pregnancy test to determine eligibility may be performed at screening or on Day 0 prior to first vaccination. Serum pregnancy tests may be used to confirm the results of, or substitute for, a urine pregnancy test.

[d] Blood draws required at post-enrollment vaccination visits must be performed prior to administration of study agent; however, it is not necessary to have results prior to administration. Lab tests may be drawn within the 3 days prior to vaccination.

[e] Reactogenicity assessments performed daily for up to 3 days post-vaccination (see Section 14.1).
