# Supplementary material for: Safety and Immunogenicity of an HIV-1 Gag DNA Vaccine with or without IL-12 and/or IL-15 Plasmid Cytokine Adjuvant in Healthy, HIV-1 Uninfected Adults
Source: PLoS One. 2012 Jan 5;7(1):e29231. doi: 10.1371/journal.pone.0029231 (PMC3252307; doi:10.1371/journal.pone.0029231)
Supplement: Protocol S2 — Trial Protocol HVTN 063. (PDF) [file pone.0029231.s003.pdf]

HIV VACCINE
TRIALS NETWORK

PROTOCOL
HVTN 063

A Phase I clinical trial to evaluate the safety and immunogenicity of HIV-1 *gag* DNA vaccine alone or with *IL-15* DNA, boosted with HIV-1 *gag* DNA + *IL-15* DNA, or HIV-1 *gag* DNA + *IL-12* DNA, in healthy, HIV-1 uninfected adult participants

BB IND 12439 HELD BY DAIDS

CLINICAL TRIAL SPONSORED BY

Division of AIDS (DAIDS)
National Institute of Allergy and Infectious Diseases (NIAID)
National Institutes of Health (NIH)
Department of Health and Human Services (DHHS)
Bethesda, Maryland, USA

VACCINE PROVIDED BY

Wyeth Vaccines Research
Pearl River, New York, USA

April 11, 2006

HVTN 063, Version 2, FINAL

Protocol HVTN 063 is designed as a two-part trial. Part A is a dose escalation safety study and includes 4 groups receiving HIV-1 *gag* DNA vaccine with escalating doses of the *IL-15* DNA adjuvant, or placebo. Part B is a regimen selection design. Each group in Part B examines HIV-1 *gag* DNA vaccine + *IL-15* DNA at the highest dose used in Part A, as a priming regimen, followed by one of 3 different boosting strategies. One group (Group 5) receives 2 additional boost injections of HIV-1 *gag* DNA vaccine + *IL-15* DNA. Another group (Group 6) receives 2 boost injections of multi-epitope peptide (MEP) vaccine. A third group (Group 7) receives 2 boost injections of HIV-1 *gag* DNA vaccine + *IL-12* DNA. The trial is being re-designed to eliminate Group 6.

The rationale for the change is that Wyeth Vaccines Research has recently indicated that the Group 6 regimen is not a priority for testing, as a similar regimen is being tested in HVTN 060, Group 7, in which 30 participants will receive HIV-1 *gag* DNA vaccine + *IL-12* DNA boosted with MEP vaccine, and 6 participants will receive placebo. Wyeth has indicated that the HVTN 060 trial provides an opportunity to test the concept of a cytokine plasmid adjuvanted DNA vaccine prime followed by MEP boost, so that the concept need not be repeated in HVTN 063.

Part B has been modified such that Group 6 will be eliminated. Groups 5 and 7 are being retained in order to 1) evaluate the safety, tolerability, and immunogenicity of HIV-1 *gag* DNA vaccine + *IL-15* DNA with a total of 5 injections, and to 2) evaluate the safety, tolerability and immunogenicity of a prime-boost regimen of HIV-1 *gag* DNA vaccine + *IL-15* DNA followed by HIV-1 *gag* DNA vaccine + *IL-12* DNA.

Per this amendment, all references to Group 6 have been deleted from the protocol. However, Groups 5 and 7 will retain their respective group numbers in order to avoid a major impact on protocol logistics such as data management plans and programming.

Brief contents

Detailed contents ... 4
Tables and figures ... 8
Schema ... 9
Overview ... 10
Introduction ... 13
Ethical considerations ... 14

Study products

1 Product background ... 16
2 Study product descriptions ... 24
3 Preclinical studies ... 26
4 Clinical studies ... 39
5 Summary ... 40

Study design

6 Study objectives ... 44
7 Study type, study population, and eligibility criteria ... 45
8 Safety and immunogenicity evaluations ... 48
9 Statistical considerations ... 53

Study operations

10 Protocol conduct ... 62
11 Informed consent ... 63
12 Procedures ... 65
13 Study product preparation and administration ... 68
14 Safety monitoring and review ... 76
References ... 85
Protocol history ... 92
Protocol team ... 93

Appendices

Appendix A: Sample informed consent form (Part A) ... 96
Appendix B: Sample informed consent form (Part B) ... 115
Appendix C: Laboratory procedures for Part A ... 135
Appendix D: Laboratory procedures for Part B ... 136
Appendix E: Procedures at HVTU for Part A ... 137
Appendix F: Procedures at HVTU for Part B ... 138
Appendix G: Sample Addendum to Informed Consent Form for PART A ... 139

Detailed contents

Brief contents ... 3
Tables and figures ... 8
Schema ... 9
Overview ... 10
Introduction ... 13
Ethical considerations ... 14

Study products

1 Product background ... 16
1.1 Plasmid DNA vaccination ... 18
1.2 DNA-based HIV vaccines ... 18
1.3 Cytokine-augmented DNA vaccination ... 19
1.3.1 IL-15 background ... 19
1.3.2 rIL-15 as vaccine adjuvant ... 20
1.3.3 *IL-15* DNA as adjuvant ... 20
1.4 IL-12 background ... 21
1.5 Recombinant human IL-12 (rhIL-12) protein ... 22
1.5.1 Clinical studies of rhIL-12 protein in therapeutic settings ... 22
1.5.2 Clinical studies of rhIL-12 protein as vaccine adjuvant ... 22
1.5.3 *IL-12* DNA compared to rhIL-12 protein ... 22
1.6 *IL-12* DNA as a vaccine adjuvant ... 23
2 Study product descriptions ... 24
2.1 HIV-1 *gag* DNA vaccine ... 24
2.2 *IL-15* DNA adjuvant ... 24
2.3 IL-12 DNA adjuvant ... 25
2.4 Placebo ... 25
3 Preclinical studies ... 26
3.1 Preclinical safety studies ... 26
3.1.1 Preclinical safety studies of *gag* DNA ... 26
3.1.2 Preclinical safety studies of *gag* DNA and *IL-15* DNA ... 26
3.1.3 Safety of SIV *gag* DNA and *IL-15* DNA in Rhesus macaques ... 27
3.1.4 Biodistribution study in rabbits ... 27
3.1.5 Preclinical safety studies of *gag* DNA and *IL-12* DNA ... 28
3.1.5.1 Toxicology study of murine *IL-12* DNA alone or in combination with HIV-1 *gag* DNA in mice ... 30
3.1.5.2 Toxicology study of *IL-12* DNA alone or in combination with HIV-1 *gag* DNA in New Zealand white rabbits ... 30
3.1.5.3 Biodistribution analysis of *IL-12* DNA and *gag* DNA in rabbits ... 31
3.1.5.4 Integration analysis of *IL-12* DNA and *gag* DNA in rabbits ... 31
3.1.5.5 Safety of rhesus *IL-12* DNA plasmid in rhesus macaques ... 32
3.2 Preclinical immunogenicity studies ... 32
3.2.1 In vivo expression and immunogenicity studies in mice ... 32
3.2.1.1 Study in Balb/c mice: IL-15-*1696* with or without HIV gag-*3339* ... 32
3.2.1.2 Kinetics of IL-15 Expression in Mice ... 34
3.2.2 Rhesus *IL-15* DNA: augmentation of SIV *gag* p37-specific immune responses in rhesus macaques ... 35

3.2.3 Preclinical immunogenicity studies of SIV *gag* DNA alone and with rhesus *IL-12* DNA in rhesus macaques 37
4 Clinical studies 39
4.1 HIV-1 *gag* DNA vaccine 39
4.2 *IL-15* DNA adjuvant 39
4.3 *IL-12* DNA adjuvant 39
5 Summary 40
5.1 Rationale for trial design 40
5.2 HVTN 060 and timing of initiation of HVTN 063 40
5.3 Plans for future product development and testing 40

Study design

6 Study objectives 44
6.1 Primary objectives 44
6.2 Secondary objectives 44
7 Study type, study population, and eligibility criteria 45
8 Safety and immunogenicity evaluations 48
8.1 Considerations for trial start 48
8.2 Initial safety evaluation 48
8.3 Safety considerations for dose escalation 48
8.4 Safety evaluation for moving from Part A to Part B 48
8.5 Other safety considerations 49
8.5.1 Exclusion of participants with allergy to amide type local anesthetics 49
8.5.2 Monitoring of autoimmune illness 49
8.6 Distinguishing intercurrent HIV infection from vaccine-induced positive serology 49
8.7 Immunogenicity evaluations 50
8.7.1 Humoral immunogenicity studies (HVTN) 50
8.7.1.1 Binding antibodies by ELISA 50
8.7.2 Cellular immunogenicity studies for <u>Part A</u> (HVTN) 50
8.7.2.1 IFN-γ ELISpot 50
8.7.3 Intracellular cytokine staining (ICS) 50
8.7.4 Cellular immunogenicity studies for <u>Part B</u> (HVTN) 51
8.7.4.1 IFN-γ ELISpot 51
8.7.4.2 Intracellular cytokine staining (ICS) 51
8.8 Ancillary studies 51
8.8.1 Assay development (HVTN) 51
8.8.2 T-cell receptor repertoire studies for Part B (Vanderbilt University) 51
8.8.3 Ancillary studies (Wyeth) 51
8.8.3.1 Humoral immunogenicity studies for Part A 51
8.8.3.2 Humoral immunogenicity studies for Part B 52
8.8.3.3 Cellular immunogenicity studies 52
8.9 HLA typing 52
9 Statistical considerations 53
9.1 Overview 53
9.2 Objectives 53
9.3 Endpoints 53
9.3.1 Safety 53
9.3.2 Immunogenicity 53
9.3.3 Social impacts 53
9.4 Accrual and sample size 53

9.4.1 Sample size calculations for safety 54
9.4.2 Sample size calculations for immunogenicity 55
9.5 Statistical analysis 56
9.5.1 Analysis variables 56
9.5.2 Baseline comparability 56
9.5.3 Safety analysis 56
9.5.4 Immunogenicity analysis 57
9.5.4.1 Missing data 58
9.5.5 Social impact analysis 58
9.5.6 Analyses prior to end of study 58
9.6 Randomization of treatment assignments 59

Study operations

10 Protocol conduct 62

11 Informed consent 63
11.1 Screening consent form 63
11.2 Protocol-specific consent form 63
11.3 Assessment of understanding 64

12 Procedures 65
12.1 Pre-enrollment procedures 65
12.2 Post-enrollment procedures 66
12.3 Total blood volumes 66
12.4 Autoimmune symptoms assessment 66
12.5 Laboratory procedures 66

13 Study product preparation and administration 68
13.1 Schemas and vaccines regimens 68
13.2 Study products formulation and preparation 70
13.2.1 HIV *gag* DNA (HIV-1 *gag* DNA Vaccine, GENEVAX® *gag-2962*) 70
13.2.2 *IL-15* DNA (*IL-15* adjuvant, GENEVAX® *IL-15*-1696) 70
13.2.3 *IL-12* DNA (*IL-12* adjuvant, GENEVAX® *IL-12*-4532) 71
13.2.4 Placebo (Sodium Chloride Injection USP, 0.9%, NaCl 0.9%) 71
13.2.5 Preparation of HIV *gag* DNA 1500 mcg or Placebo (Group 1) 71
13.2.6 Preparation of HIV *gag* DNA 1500 mcg / *IL-15* DNA 100 mcg or Placebo (Group 2) 71
13.2.7 Preparation of HIV *gag* DNA 1500 mcg / *IL-15* DNA 500 mcg or Placebo (Group 3) 72
13.2.8 Preparation of HIV *gag* DNA 1500 mcg / *IL-15* DNA 1500 mcg or Placebo (Groups 4- 7) 73
13.2.9 Preparation of HIV *gag* DNA 1500 mcg / *IL-12* DNA 1500 mcg or Placebo (Group 7) 73
13.2.10 Pharmacy procedures to preserve blinding 74
13.3 Study products administration 74
13.3.1 Administration of vaccinations 74
13.4 Study products acquisition 74
13.5 Pharmacy records 75

14 Safety monitoring and review 76
14.1 Assessing reactogenicity 76
14.2 Grading adverse experiences 76
14.3 Adverse event reporting and safety pause /AE review rules 76
14.3.1 Adverse events to which safety pause /AE review rules apply 77

14.3.2 Adverse event reporting 77
14.3.3 PSRT AE review and safety pause 77
14.3.4 Review and notification following safety pause 79
14.4 Expedited Adverse Event Reporting to DAIDS 80
14.4.1 EAE Reporting Level 80
14.4.2 Study Agents for Expedited Reporting to DAIDS 80
14.4.3 EAE Reporting Periods 80
14.5 Compliance with the NIH Guidelines for Research Involving Recombinant DNA Molecules 80
14.6 Participant departure from schedule of vaccinations 81
14.6.1 Delaying vaccinations for a participant 81
14.6.2 Stopping vaccinations for a participant 81
14.6.3 Participant termination from the study 82
14.7 Study termination (for all participants) 82
14.8 HVTN review of cumulative safety data 82
14.8.1 Daily review 83
14.8.2 Weekly review 83
14.8.3 Quarterly review 83
14.8.4 Safety Monitoring Board review 83
14.8.5 Review for dose escalation 84
14.8.6 Review for advancing from Part A to Part B 84
References 85
Protocol history 92
Protocol team 93

Appendices

Appendix A: Sample informed consent form (Part A) 96
Appendix B: Sample informed consent form (Part B) 115
Appendix C: Laboratory procedures for Part A 135
Appendix D: Laboratory procedures for Part B 136
Appendix E: Procedures at HVTU for Part A 137
Appendix F: Procedures at HVTU for Part B 138
Appendix G: Sample Addendum to Informed Consent Form for PART A 139

Tables and figures

Figure 1-1 Coordination of product trials 17

Table 2-1 Structural differences between *gag* plasmids APL-400-047 and GENEVAX® *gag*-2962 plasmid 24

Table 3-1 Summary of the toxicology studies with *IL-15* DNA-1696 26

Table 3-2 Levels (copy numbers) of GENEVAX *IL-15*-1696 in injection site 28

Table 3-3 Summary of toxicology studies with human *IL-12* DNA - 6285 29

Table 3-4 Design of study to evaluate *IL-15* DNA as adjuvant in mice* 32

Figure 3-1 Splenocytes isolated at day 56 (2 weeks post 3rd immunization) were tested by Gag-specific IFN-γ ELISpot analysis. Units on y-axis are SFC/million splenocytes. Data are representative of two separate experiments, as represented by Table 3-4. 33

Figure 3-2 Serum samples on day 56 (2 weeks post 3- immunization) were tested for Gag-specific IgG response. Units on y-axis are Log Mean Titers. Data are representative of 2 separate experiments. 33

Table 3-5 Study design of expression kinetic study in Balb/c mice: GENEVAX *IL-15*-1696 plus GENEVAX *gag*-2962, an HIV DNA complexed with bupivacaine 34

Figure 3-3 Expression of IL-15 in mouse muscle for groups injected with *IL-15*-1696 DNA, *IL-15*-1696 + *gag*-2962 DNA or backbone control. 35

Table 3-6 Experimental design of study evaluating rhesus *IL-15* DNA administered as an adjuvant with SIV *gag* DNA 36

Figure 3-4 SIV Gag-specific IFN-γ ELISpot assay 36

Figure 3-5 SIV p27Gag-specific ELISA assay 37

Table 3-7 IFN-γ ELISpot responses (per 10^6 cells) in monkeys immunized with SIV *gag* DNA and rhesus *IL-12* DNA 38

Table 7-1 Study inclusion criteria 46

Table 7-2 Study exclusion criteria 47

Table 9-1 Probability of response for different safety scenarios 55

Table 9-2 Exact 95% 2-sided confidence intervals for response rates based on observing a particular rate of responses in the vaccinees (n=27). 55

Table 9-3 Minimum detectable differences in response rates between 2 groups (of size 27) 56

Table 12-1 Pre-enrollment procedures 65

Table 12-2 Post-enrollment procedures 66

Table 14-1 Schedule of reactogenicity assessments 76

Protocol history and modifications 92

Schema

Study products

gag DNA: HIV-1 *gag* DNA vaccine at 1500 mcg per dose (formulated with bupivacaine)

IL-15 DNA: *IL-15* DNA adjuvant at 100, 500 and 1500 mcg per dose (formulated with bupivacaine)

IL-12 DNA: *IL-12* DNA adjuvant at 1500 mcg per dose (formulated with bupivacaine)

Placebo: Sodium chloride injection USP, 0.9%

Administration: Single intramuscular injection in the deltoid by needle and syringe.

Volumes:

0, 1, 3 months: Group 1, 0.75 mL; Group 2, 0.8 mL; Group 3, 1 mL; Groups 4–7, 1.5 mL

6, 9 months: Group 5, 1.5 mL; Group 7, 1.5 mL

					Vaccination schedule in months (days)			Booster schedule in months (days)	
Study arm	N	*gag* DNA dose (mcg)	*IL-15* DNA dose (mcg)	*IL-12* DNA dose (mcg)	0 (0)	1 (28)	3 (84)	6 (168)	9 (273)
**Part A**									
1	10	1500	—	—	*gag* DNA	*gag* DNA	*gag* DNA	—	—
	2	—	—	—	placebo	placebo	placebo	—	—
2	10	1500	100	—	*gag* DNA + *IL-15* DNA	*gag* DNA + *IL-15* DNA	*gag* DNA + *IL-15* DNA	—	—
	2	—	—	—	placebo	placebo	placebo	—	—
3	10	1500	500	—	*gag* DNA + *IL-15* DNA	*gag* DNA + *IL-15* DNA	*gag* DNA + *IL-15* DNA	—	—
	2	—	—	—	placebo	placebo	placebo	—	—
4	10	1500	1500	—	*gag* DNA + *IL-15* DNA	*gag* DNA + *IL-15* DNA	*gag* DNA + *IL-15* DNA	—	—
	2	—	—	—	placebo	placebo	placebo	—	—
Pause for safety evaluation									
**Part B**									
5	30	1500	1500*	—	*gag* DNA + *IL-15* DNA	*gag* DNA + *IL-15* DNA	*gag* DNA + *IL-15* DNA	*gag* DNA + *IL-15* DNA	*gag* DNA + *IL-15* DNA
	6	—	—	—	placebo	placebo	placebo	placebo	placebo
6	0	—	—	—	—	—	—	—	—
	0	—	—	—	—	—	—	—	—
7	30	1500	1500*	1500**	*gag* DNA + *IL-15* DNA	*gag* DNA + *IL-15* DNA	*gag* DNA + *IL-15* DNA	*gag* DNA + *IL-12* DNA	*gag* DNA + *IL-12* DNA
	6	—	—	—	placebo	placebo	placebo	placebo	placebo
Total		A (48) + B (72) = 120							

* Maximum tolerated dose from Part A.

** Maximum tolerated dose from HVTN 060.

Enrollment in Groups 1 and 2 will occur simultaneously. See Section 8.2 regarding initial safety evaluation.

Enrollment in Groups 3 and 4 will be sequential. See Section 8.3 for dose escalation criteria.

Enrollment in Groups 5 and 7 (Part B) will occur simultaneously. See Section 8.4 regarding safety evaluation for moving from Part A to Part B

Overview

Title

A Phase I clinical trial to evaluate the safety and immunogenicity of HIV-1 *gag* DNA vaccine alone or with *IL-15* DNA, boosted with HIV-1 *gag* DNA + *IL-15* DNA, or HIV-1 *gag* DNA + *IL-12* DNA, in healthy, HIV-1 uninfected adult participants

Participants

Healthy HIV-1-uninfected adult participants (18 to 50 years old) from US and non-US HVTUs (to be selected contingent on site preparedness)

Number of participants

Part A: 48 (40 vaccinees, 8 placebo recipients)

Part B: 72 (60 vaccinees, 12 placebo recipients)

Total: 120 (100 vaccinees, 20 placebo recipients)

Primary objectives

Part A

To evaluate the safety and tolerability of intramuscular administration of HIV-1 *gag* DNA vaccine alone, and of HIV-1 *gag* DNA vaccine plus *IL-15* DNA (at escalating doses of 100 mcg, 500 mcg, and 1500 mcg).

Part B

To evaluate the safety and tolerability of intramuscular administration of HIV-1 *gag* DNA vaccine plus *IL-15* DNA as a priming series, followed by boost vaccinations with HIV-1 *gag* DNA vaccine plus *IL-15* DNA or HIV-1 *gag* DNA vaccine plus *IL-12* DNA.

Study products

HIV-1 gag *DNA vaccine*

The HIV-1 *gag* DNA vaccine contains an RNA-optimized truncated *gag* gene (p37) derived from strain HXB2. The plasmid backbone includes a eukaryotic gene expression unit that contains elements from the human cytomegalovirus (hCMV) immediate early promoter/enhancer and the bovine growth hormone (BGH) polyadenylation signal, a chimeric kanamycin resistance gene, and a pUC bacterial origin of replication. The vaccine is formulated in 30 mM citrate buffer pH 6.5 containing 150 mM NaCl, 0.01% ethylenediamine tetraacetic acid (EDTA), and 0.25% bupivacaine-HCl.

IL-15 *DNA adjuvant*

GENEVAX® *IL-15*-1696 consists of the human *IL-15* gene inserted into the DNA plasmid expression vector WLV001M, the same plasmid backbone used for the HIV-1 *gag* DNA vaccine. The human *IL-15* gene has been optimized for high-level expression. The *IL-15* DNA is formulated in 30 mM citrate buffer pH 6.5 containing 150 mM NaCl, 0.01% EDTA, and 0.25% bupivacaine-HCl.

IL-12 *DNA adjuvant*

The *IL-12* DNA-4532 adjuvant is a dual promoter expression plasmid which expresses the genes encoding human IL-12 proteins p35 and p40 under separate regulatory control. The p35 subunit is under the control of the hCMV promoter/enhancer and the SV40 (simian virus 40)

polyadenylation signal. The p40 subunit is under the control of the SCMV (simian cytomegalovirus) promoter and the BGH (bovine growth hormone) polyadenylation signal. The plasmid contains a chimeric kanamycin resistance gene and a pUC bacterial origin of replication. The *IL-12* DNA is formulated in 30 mM citrate buffer pH 6.5 containing 150 mM NaCl, 0.01% EDTA, and 0.25% bupivacaine-HCl.

Placebo

Sodium chloride injection USP, 0.9%

Study design

Multicenter, randomized, placebo-controlled, double-blind trial

Study duration

Part A: 12 months per participant

Part B: 18 months per participant

Safety monitoring

HVTN 063 Protocol Safety Review Team

HVTN Safety Monitoring Board

Vaccine provider

Wyeth Vaccines Research (Pearl River, New York, USA)

Sponsor

Division of AIDS (DAIDS), National Institute of Allergy and Infectious Diseases (NIAID), National Institutes of Health (NIH), Department of Health and Human Services (DHHS) (Bethesda, Maryland, USA)

IND holder

DAIDS, NIH

Study sites

US and non-US HIV Vaccine Trials Units (HVTUs) to be selected contingent on site preparedness

HVTN Core Operations

HVTN Vaccine Leadership Group/Core Operations Center (HVTN Core), Fred Hutchinson Cancer Research Center (FHCRC) (Seattle, Washington, USA)

Statistical and data management center

Statistical Center for HIV/AIDS Research and Prevention (SCHARP), FHCRC

Central laboratories

Duke University Medical Center (Durham, North Carolina, USA)

FHCRC/University of Washington (Seattle, Washington, USA)

University of Washington Virology Specialty Laboratory (Seattle, Washington, USA)

South Africa Immunology Laboratory and National Institute for Communicable Disease (Johannesburg, South Africa)

Protocol leadership

Chair: Lindsey Baden MD, Harvard University, Boston, Massachusetts, USA

Co-Chair: Xia Jin MD PhD, University of Rochester, Rochester, New York, USA

Clinical Trials Physician: Marnie Elizaga MD, HVTN Core, Seattle, Washington, USA

Statistician: Barbara Metch MS, SCHARP, Seattle, Washington, USA

DAIDS Medical Officer: Massimo Cardinali MD, DAIDS, Bethesda, Maryland, USA

Introduction

The ongoing worldwide epidemic of the human immunodeficiency virus type 1 (HIV-1) remains one of the major global health challenges. HIV-1 causes the acquired immunodeficiency syndrome (AIDS), which is responsible for tremendous human suffering and economic loss throughout the world. Currently, over 39 million people are living with HIV-1 infection [1]. Without treatment, it is likely that nearly all of these will die of AIDS in the next 2 decades.

Since 1996, potent new antiretroviral therapies, including combination regimens with protease inhibitors, have created the possibility that HIV-1 infection might become a chronic, manageable disease among individuals with access to these medications. In the US, AIDS deaths are down to 18,000 per year as a result of the new antiretrovirals [2]. However, for the developing world, where over 98% of the nearly 5 million annual incident HIV-1 infections occur [1], it is unlikely that these drugs will be widely accessible, due to many logistical challenges associated with their use.

Globally, 13,000 new infections occur each day. More than 3 million AIDS deaths occur per year [1], and nearly 20 million have died since the HIV epidemic began [3]. AIDS has become the leading infectious disease killer, the fourth leading cause of death overall. In severely affected countries, life expectancy has fallen by more than 10 years [1]. AIDS is the leading killer in Africa, with over 25 million Africans living with HIV/AIDS. Sub-Saharan Africa has been affected most; in 7 Sub-Saharan African countries, over 22 million adults (aged 15-49) are living with HIV/AIDS [3]. For example, in Botswana, 37.3% of adults aged 15 to 49 are infected with HIV, while in South Africa more than 25% of women in antenatal clinics are infected [3].

After sub-Saharan Africa and Asia, Latin America is the region most severely affected by HIV infection. The HIV epidemic in Latin America reflects diverse transmission patterns: in Andean countries HIV is most often transmitted sexually, primarily among men who have sex with men (MSM), while in Brazil, Uruguay and Argentina a significant proportion (39% of AIDS cases in Argentina) of HIV transmission occurs through injection drug use (IDU). In all countries of the Andean Region, MSM account for a substantial proportion of HIV infections and comprise a “bridge” group for spread into the heterosexual population due to the high frequency of bisexuality. HIV incidence is higher among high-risk MSM in Brazil and Peru compared to most U.S. populations [4-9].

The need for better education, better treatment access, better prevention programs, and better prevention technologies is therefore clear. Specifically, the need for a safe, effective, and affordable HIV-1 vaccine is paramount [10,11]. The ideal HIV-1 vaccine for global use should meet several of the following criteria:

- proven safety in healthy HIV-uninfected persons
- induction of long-lasting HIV-specific cell-mediated and humoral immunity capable of conferring protection against HIV
- tolerability
- potential for production in sufficient quantity to meet global needs
- affordability
- stability during distribution and storage

Ethical considerations

Multiple candidate HIV vaccines will need to be studied simultaneously in different populations around the world before a successful HIV preventive vaccine is found. It is critical that universally accepted ethical guidelines are followed at all sites involved in the conduct of these clinical trials. The HVTN has addressed ethical concerns in the following ways:

- HVTN trials are designed and conducted to enhance the knowledge base necessary to find a preventive vaccine, with methodology that is scientifically rigorous and valid, and in accordance with Good Clinical Practice (GCP) guidelines.
- HVTN scientists and protocol team members incorporate the philosophies underlying major codes, declarations, and other guidance documents relevant to human subject research into the design and conduct of HIV vaccine clinical trials.
- HVTN scientists and protocol team members are committed to substantive community input into the planning, conduct, and follow up of the research which will help ensure that locally appropriate cultural and linguistic needs of study populations are met.
- The HVTN advocates that all HVTN sites should develop a plan for the care and treatment of participants who develop HIV infection during a trial. This plan should be formulated by representatives of host countries, communities from which potential trial participants will be drawn, sponsors, and the HVTN.
- Prior to implementation, HVTN trials are rigorously reviewed by both local and national regulatory bodies, in addition to scientists who have no involvement with the trial under consideration.
- The HVTN provides training so that all participating sites similarly ensure fair subject selection, protect the privacy of research subjects, and obtain meaningful informed consent.
- The HVTN recognizes the importance of institutional review and values the role of in-country Institutional Review Boards (IRBs) and Institutional Ethics Committees (IECs) as custodians responsible for ensuring the ethical conduct of research in the local setting.

STUDY PRODUCTS

1 Product background

Despite the major strides that have been made in HIV therapy with the advent of potent antiretroviral drugs, these medications are quite expensive and are still not readily available for the vast majority of infected individuals worldwide. Even when the medications are available, the associated long-term toxicities and the frequent emergence of drug-resistance mutations can complicate therapy, making the formulation of effective vaccines imperative. Although it is unclear at present which vaccine-induced immune responses will protect against infection, the evidence to date suggests that vaccine-induced cellular immune responses are able to control viremia and prevent disease progression in animal infection models. The ability to measure immune responses has also advanced markedly over the past few years and will allow investigators to more accurately measure the immunogenicity of vaccine constructs, and correlate the magnitude and breadth of these responses with protection.

Cytotoxic T lymphocytes (CTL) are generated early during the course of acute HIV-1 infection. After infection of CD4+ T cells, viral proteins that are generated in the cytosol are degraded and presented as epitopic peptides (usually 9 to 11 amino acids in length) on the cell surface complexed to HLA Class I molecules. CTL recognize infected cells through the interaction of the T cell receptor (TCR) with the HLA-epitope complex. This occurs prior to the assembly of progeny virions, a process that takes approximately 2.6 days. During this time, an infected cell is vulnerable to attack by CTL, and if eliminated at this time, progeny virus will not be released [12,13]. CTL are also able to mediate an antiviral effect through the elaboration of soluble factors that inhibit viral replication. These include the chemokines RANTES, MIP-1alpha and MIP-1beta, as well as other factors not yet fully defined. The release of these factors occurs when the TCR recognizes an infected cell. In fact, these factors are released concurrently with the mobilization of the cell's cytolytic machinery when an infected cell is recognized [14], and this likely has an important effect on the microenvironment of the infected cell. RANTES, MIP-1alpha and MIP-1beta have been shown to inhibit HIV infection of cells by competing with the virus for chemokine coreceptors present on the cell surface that are necessary for viral entry. Although the exact contribution of each of these two mechanisms toward suppression of HIV infection *in vivo* is not clear, it is likely that they act synergistically to contain cell-to-cell spread of HIV.

The generation of cytotoxic T lymphocyte responses after exposure to viral pathogens involves a complex relationship between antigen presenting cells and T cells. Chemokines such as MIP-1alpha and MIP-1beta (among others) recruit T and B cells to sites of inflammation, where dendritic cells interact with naïve T cells [15]. These interactions lead to CD40L upregulation on CD4+ T cells, which leads to upregulation of CD40 [16], costimulatory molecules , and the release of cytokines such as IL-12 and IL-15 from mature dendritic cells [17]. This series of events has been shown to be fundamental to the generation of high quality and high magnitude CD4+ Th and CD8+ CTL responses.

Wyeth Research is pursuing development of a combination HIV vaccine consisting of CTL multiepitope peptide (HIV CTL MEP) and facilitated DNA technology (HIV-1 *gag* DNA + *IL-12* or *IL-15* DNA) platforms. The HIV CTL MEP and HIV-1 *gag* DNA + *IL-12* DNA vaccine approaches are currently being examined in integrated Phase I prime/boost studies:

- In HVTN 060, priming with HIV-1 *gag* DNA ± *IL-12* DNA will be followed by boost vaccination(s) with HIV-1 *gag* DNA alone, HIV CTL MEP vaccine, or HIV-1 *gag* DNA + *IL-12* DNA.

- In the present study, the prime/boost strategy consists of priming with HIV-1 *gag* DNA + *IL-15* DNA followed by boosting with HIV-1 *gag* DNA + *IL-15* DNA or HIV-1 *gag* DNA + *IL-12* DNA.

The above referenced studies are presented diagrammatically in Figure 1-1. Detailed descriptions of the individual components of the vaccine are presented in Section 2.

Prior to beginning the booster vaccination series in Part B of HVTN 063, the safety data for HIV-1 *gag* DNA + *IL-12* DNA from HVTN 060 Part A will be evaluated.

Figure 1-1 Coordination of product trials

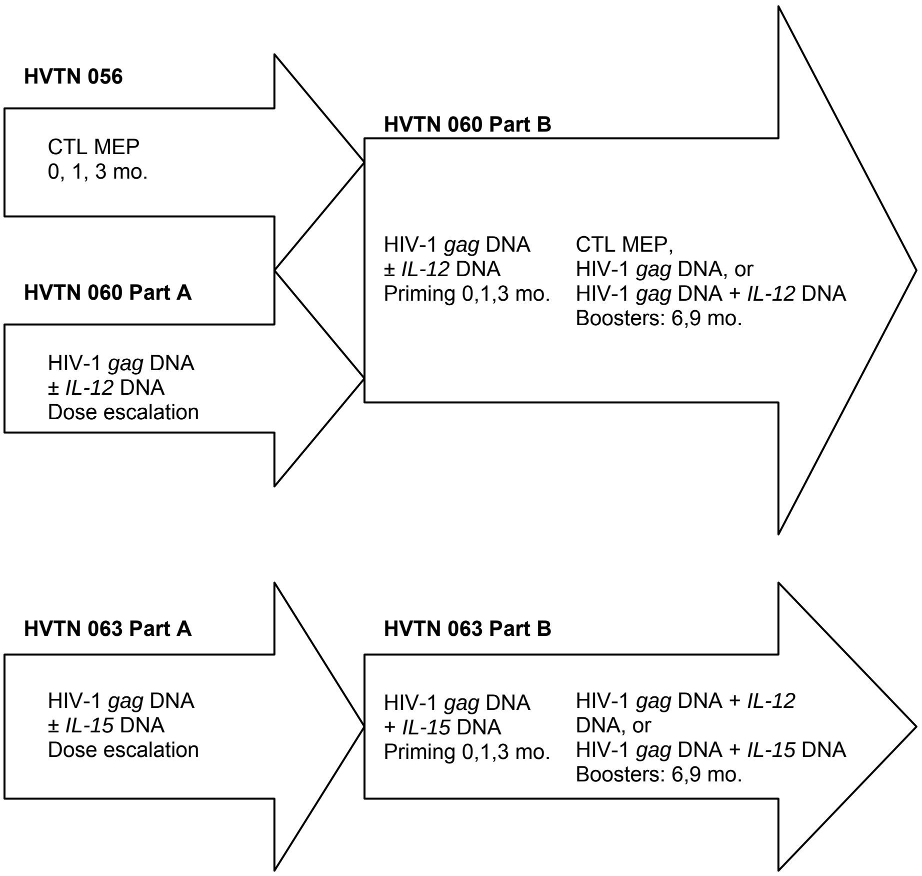

1.1 Plasmid DNA vaccination

Plasmid DNA vaccination is a novel vaccination modality that has been shown to elicit both humoral and cellular immune responses in animal models. DNA vaccines are also simple and inexpensive to construct, readily produced in large quantities, and stable for long periods of time. If DNA vaccination proves to be efficacious, production and delivery to individuals in developing nations may be more economically and logistically feasible than with other types of vaccines.

Plasmid DNA vaccination involves the administration of purified plasmid DNA encoding an antigen. Plasmid DNA is typically injected into skeletal muscle or, alternatively, inoculated as plasmid-coated beads by gene gun into the epidermis. The protein is expressed in transfected mammalian cells, including macrophages and dendritic cells; enters into both the MHC Class I and Class II processing pathways; and elicits strong and persistent humoral and cellular immune responses [18-21].

Results of DNA vaccine clinical trials for a number of infectious diseases have been reported. A Phase I study of a malaria gene-based DNA vaccine demonstrated that CTLs and IFN-alpha–producing T lymphocytes could be induced in human participants [22]. In a Phase I trial in 20 healthy adult participants to test the safety, tolerability, and immune responses to a DNA vaccine for malaria (containing a backbone very similar to that being proposed in the current study) there were no severe or serious adverse experiences and no significant clinical or laboratory findings [23]. Anti-dsDNA antibodies were not detected. Furthermore, CTLs reactive with the expressed *Plasmodium falciparum* circumsporozoite (*Pf*CSP) protein were observed, although no antigen-specific antibodies were detected. In a Phase I study of a DNA vaccine encoding Hepatitis B surface antigen, the vaccine was delivered by the gold bead particle acceleration PowderJect XR1 gene gun into 6 Hepatitis B seronegative subjects and 1 seropositive subject. Although none of the seronegative subjects developed primary immune responses in the form of antibodies, the vaccine was well tolerated with minimal, mostly local reactions [24]. Overall, DNA vaccination in humans is a promising vaccination modality that has achieved a good safety record but has not yet achieved its full potential in terms of immunogenicity.

1.2 DNA-based HIV vaccines

DNA vaccines have shown partial efficacy in viral challenge studies in nonhuman primates. SIV *env* DNA vaccination of rhesus monkeys reduced viral loads following challenge with the virulent SIVmac251 isolate in rhesus monkeys [25]. Furthermore, SIV *gag* DNA vaccination of rhesus monkeys led to the development of potent CTL responses and reduced viral loads following intravenous challenge with the pathogenic SIVmn E660 virus [26]. These and other nonhuman primate studies [27,28] have prompted clinical trials of DNA-based HIV vaccines.

The first clinical trial of a DNA vaccine in HIV seropositive individuals was conducted by Wyeth (Apollon) and evaluated the intramuscular injection of a DNA vaccine expressing the Env and Rev proteins. This study provided extensive safety data and revealed no significant adverse clinical or laboratory findings in any of the subjects. In particular, there was no evidence of antinuclear antibody formation, muscle enzyme elevation, or changes in blood pressure and pulse [29].

After encouraging results from this preliminary study, a blinded Phase I study of the Env- and Rev-encoding DNA vaccine (GENEVAX® HIV, Apollon) was undertaken in healthy, uninfected subjects [30]. The vaccine was given at a dose of 100 mcg, 300 mcg or 1000 mcg

by intramuscular injection. Participants demonstrated modest but sporadic T lymphocyte immune responses, but the conclusions were limited by the small number of subjects (6 per group). In both studies, the plasmid was administered with bupivacaine to enhance DNA uptake, gene expression, and immune responses. Results of the study suggested that further investigations should be undertaken with higher doses and enhancements with different formulation or combination strategies.

Others have also evaluated DNA-based HIV vaccines in clinical studies and have shown that DNA-based HIV vaccines show promise when used in connection with a mixed modality booster immunogen [31].

1.3 Cytokine-augmented DNA vaccination

The safety and partial efficacy of DNA vaccination in nonhuman primate studies has led to a number of approaches to augment the efficacy of this vaccine modality. One such strategy involves the boosting of a DNA-primed immune response with a recombinant live vector. A second promising strategy involves the coadministration of plasmid-encoded immunomodulator molecules. Augmentation of DNA vaccine–elicited HIV-specific cellular immune responses in mice has been reported by a number of laboratories using plasmids expressing GM-CSF, IL-2, CD40L, IL-12, IL-15, B7-2, ICAM-1, LFA-3, RANTES, MIP-1 alpha, or MCP-1 [32-36].

Cytokines are molecules secreted mainly by bone marrow–derived cells that act in an autocrine or paracrine manner to induce a specific response in cells expressing the appropriate cytokine receptor. Recent vaccine strategies have attempted to incorporate cytokine-expressing plasmids with the viral genes of interest in efforts to elicit cell-mediated immune responses. Several animal studies have shown that *IL-15* DNA injection enhances Th1 dependent responses to plasmid-based vaccines [37-41]. These studies, in combination with the preliminary results summarized in this protocol and the *IL-15* DNA Investigator's Brochure, provide the rationale to pursue HIV *gag* and *IL-15* plasmid–based vaccines in this first in human study to determine whether DNA encoding IL-15 is safe and whether it provides a significant adjuvant effect.

1.3.1 IL-15 background

IL-15 was initially characterized as a T cell growth factor with similar *in vitro* properties as IL-2 [42,43]. However, a large body of evidence has now demonstrated unique *in vivo* functions for IL-15 that point towards promising applications for use as a vaccine adjuvant and for the immunotherapy of cancer [44]. IL-2 and IL-15 share several functions. These include the initial stimulation of the proliferation of activated T and B cells on antigenic stimulation, as well as the maintenance and activation of natural killer (NK) cells [45]. However, the two cytokines also provide contrasting contributions to adaptive immune responses mediated by lymphocytes [46]. IL-2 is pivotally involved in activation-induced cell death (AICD), a process that leads to the elimination of self-reactive T cells and thereby facilitates the induction of tolerance to self [47]. By contrast, IL-15 inhibits this IL-2-induced AICD process [48]. Furthermore, IL-15 stimulates the maintenance of CD8+ memory phenotype T cells, whereas IL-2 inhibits their persistence *in vivo* [46,48-53]. An analysis of mice with disrupted genes for IL-2 and IL-15 and cytokine receptors support the competitive roles for IL-2 and IL-15 in AICD and the expression of memory cells. In particular, IL-$2^{-/-}$ and IL-$2Ra^{-/-}$ mice develop the massive enlargement of peripheral lymphoid organs that is associated with polyclonal T- and B-cell expansion and also develop autoimmune diseases such as hemolytic anemia and inflammatory bowel disease [54,55]. In contrast to this

phenotype, mice genetically deficient in IL-15 or the private IL-15 receptor, IL-15Ra, do not manifest lymphoid enlargement, high immunoglobulin levels or autoimmune disease. Rather, they display a marked reduction in the numbers of NK cells and NK-T cells, as well as a marked reduction in the numbers of memory CD8+ T cells [56,57].

Taken together with the *in vitro* analyses, the *in vivo* studies support the view that, in their special adaptive immune functions, IL-2 and IL-15 favor opposing actions that tend to emphasize one or the other of the two competing major goals of the immune response. Through its contributions to AICD, its interference with the persistence of memory CD8+ T cells and its role in the persistence of CD4+CD25+ (IL-2Ra) regulatory T cells or negative-regulatory T cells, IL-2 favors the elimination of those lymphocytes that are directed towards self-antigens and thereby play a crucial role in the persistence of peripheral self–tolerance [46-48,50]. By contrast, through its inhibition of IL-2 mediated AICD and its role in the persistence of CD8+ memory T cells, IL-15 favors the persistence of CD4+ and CD8+ T cells that are of value in the maintenance of a long-lasting specific immune response to foreign pathogens [46,48,56-62].

1.3.2 rIL-15 as vaccine adjuvant

Recombinant IL-15 (rIL-15) has recently been evaluated as an adjuvant for tetanus toxoid and influenza vaccine immunization in rhesus macaques [63]. rIL-15 administered at 10 mcg/kg twice weekly for four weeks induced modest expansion of Flu specific CD8 but resulted in significantly elevated levels of CD8 memory at 6 months. rIL-15 also significantly enhanced early and late tetanus toxoid specific CD4 responses. Following booster immunization, IL-15 enhanced CD8 T cell responses, whereas rIL-2 or rIL-2/rIL-15 administration were less effective.

rIL-15 has not yet been evaluated as a vaccine adjuvant or therapeutic in humans.

1.3.3 *IL-15* DNA as adjuvant

IL-15 DNA was first shown to induce significant enhancement of cytotoxic responses by Kim *et al.* with HIV-1 DNA immunogens [64]. The observed increases in CTL response were MHC class I restricted and CD8+ dependent. The original observation was confirmed the following year by Xin *et al.* [37], who observed enhanced Th1 dependent HIV-1-specific cell mediated immunity in mice when HIV-1 DNA vaccine and *IL-15* DNA was administered by the intranasal route. More recently, Moore *et al.* [39] demonstrated *IL-15* DNA induced a potent humoral response and antigen-specific IFN-γ production to Nef when co-administered with *nef* DNA at low levels in mice. In a Herpes Simplex Virus Type 2 mouse model, the Weiner lab demonstrated that coadministration of *IL-15* DNA with HSV gD2 DNA vaccine enhanced immunity to HSV, as demonstrated by enhanced survival and a reduction in frequency and severity of herpetic lesions following intravaginal HSV challenge [38].

A key study by Oh *et al.* [59] evaluated the relative adjuvant activities of IL-15 and IL-2, which mediate their immune enhancing activities through the use related cellular receptors. Vaccinia vectors expressing HIV gp160 were coadministered to mice with additional vaccinia vectors expressing IL-15 or IL-2. Whereas both IL-2 and IL-15 in the vaccinia vector induced strong and long-lasting antibody-mediated immunity as well as short term cytotoxic T cell responses against HIV gp120, only IL-15 vaccinia supported robust CD8+ T cell–mediated long-term immunity. This observation was recently confirmed in a primate study by Villinger *et al.* [63] who compared the relative adjuvant activities of rIL-2 and rIL-15 (10 mcg/kg/dose) with respect to induction of long-term memory following immunization with influenza vaccine or tetanus toxoid vaccine. The study indicated that the initial expansion of Flu specific CD8 cells using rIL-15 was modest, but resulted in significantly elevated levels of

memory cells at 6 months. rIL-15 also significantly enhanced early and late tetanus toxoid specific CD4 responses. rIL-2 administration resulted in the initial enhancement of CD8 effector responses, but specific memory responses were no different from responses seen in cytokine non-treated monkeys.

The safety of rIL-15 administration was recently reported at doses of 10 mcg and 100 mcg/kg in SIV-infected monkeys. rIL-15 was administered twice weekly for 4 weeks and macaques were evaluated for 3 months [61]. These results indicated that administration of rIL-15 increased CD8+ T cells but not CD4+ T cells or B cells numbers in peripheral blood, nor did viral load change. Importantly, no adverse effects were associated with rIL-15 administration at the either dose.

In preclinical macaque studies, we have shown *IL-15* DNA, when co-administered with SIV *gag* DNA (1.5 mg/dose, 4 doses), significantly enhances responses to SIV *gag* without induction of adverse events (see Section 3.2.2).

1.4 IL-12 background

IL-12 was first described in 1989 as a cytokine capable of stimulating NK cell and cytotoxic lymphocyte maturation [62,65,66]. IL-12 is a heterodimeric cytokine composed of a heavy chain of 40 kDa and a light chain of 35 kDa that are covalently linked by a disulfide bond [67].

IL-12 is produced primarily by phagocytic cells, including monocytes, macrophages, and neutrophils [68]. To lesser degrees, dendritic cells, keratinocytes, and nonmucosal mast cells also possess the ability to produce IL-12. Both a T cell independent pathway (exposure to microbes or their components) and a T cell dependent pathway (interaction of CD40 ligand on activated T cells with CD40 on IL-12 producing cells) can stimulate IL-12 production [69]. Both intracellular and extracellular pathogens are capable of inducing IL-12 production from phagocytic cells. In addition, the gp120 glycoprotein of HIV has been shown to stimulate IL-12 production [70].

The major cells influenced by IL-12 are T cells and NK cells, resulting in cytokine production, proliferation of activated T lymphocytes, and enhanced NK cell proliferation [62,71-74]. The cytolytic activity of cytotoxic T and NK cells is enhanced by IL-12. IL-12 is required for the optimal proliferation of mitogen- or antigen-stimulated T cells. IL-12 increases IFN-γ production from NK and T cells, enhances NK/lymphokine-activated killer cell cytotoxicity, and promotes Th1 immune responses [74,75].

IL-12 is important in influencing the differentiation of Th1 cells from uncommitted precursor cells in response to signals derived from the innate immune system and inhibiting Th2 cell differentiation. Once Th1 cells develop, IL-12 further enhances the Th1 response by promoting activity of this cell subset.

IL-12 stimulates cellular immune responses and thus may serve to stimulate HIV-specific immunity or enhance general cell-mediated immune responses. Both these actions may improve immune responses to HIV vaccines. The capacity of IL-12 to promote Th1 responses has led to interest in its role as a vaccine adjuvant for diseases requiring cellular immune responses. In a mouse model of Leishmaniasis, a protective Th1 cell response was induced by vaccination with parasite extracts in combination with IL-12. NK cell activation and IFN-γ production were seen in vaccinated mice that protected normally susceptible mice from parasite challenge [76]. Other studies in mice have also demonstrated the development of protective immune responses to a *Mycobacterium tuberculosis* subunit vaccine, to doses of heat-killed *Listeria monocytogenes* or listerial antigen preparations, and to an acellular

pertussis vaccine when given with IL-12 [77-79]. In each of these studies Th1 responses to the vaccine were amplified by the addition of IL-12.

IL-12 may also exert effects on antibody responses. In mice, IL-12 administration has led to a shift in the isotype of antigen specific antibodies. IFN-alpha promoted the differentiation of B cells to produce cytophilic antibodies in mice (IgG2a, IgG2b, IgG3) and decreased expression of IgG1, IgE, and IgA. IL-12 as a vaccine adjuvant may modulate the type of antibody response toward those isotypes that are protective [80,81].

1.5 Recombinant human IL-12 (rhIL-12) protein

1.5.1 Clinical studies of rhIL-12 protein in therapeutic settings

Although human clinical trial data are not available for the *IL-12* DNA adjuvant, recombinant human IL-12 (rhIL-12) protein has been used extensively in various therapeutic and prophylactic settings. Over 1100 subjects have received 1 or more doses of rhIL-12 given in therapeutic trials for oncology, HIV disease, hepatitis C virus infection, and hepatitis B virus infection, as well as for malaria and asthma.

For reference purposes, the Investigator's Brochure for Recombinant Human IL-12 is provided as an attachment in the *IL-12* plasmid Investigator's Brochure.

1.5.2 Clinical studies of rhIL-12 protein as vaccine adjuvant

Several studies have demonstrated the potential of rhIL-12 as an adjuvant to specifically modulate the characteristics of a developing antibody response by enhancing expression of isotypes associated with efficacy in infectious diseases and suppressing others that may be associated with pathology. In addition, they suggest the potential for the use of IL-12 to modify the antibody response at specific sites to modulate the characteristics of an ongoing immune response. IL-12 has also been shown to enhance the absolute levels of antigen-specific antibody in serum. The mechanism for enhanced antibody production remains to be determined but may reflect expansion of T helper cells and enhanced cytokine expression. Wyeth has conducted a number of clinical studies with rhIL-12 as a vaccine adjuvant as described in the Investigator's Brochure. Safety and tolerability data from these studies support the safety of up to 2 mcg rhIL-12/dose. Those studies also revealed that rhIL-12 had a modest stimulatory effect when given with T cell dependent vaccines. At doses higher than 4 mcg of rhIL-12, an unacceptable level of adverse experiences was noted. These included headache, injection site pain, myalgia, fever, asthenia, lymphadenopathy, and other symptoms; please refer to the Investigator's Brochure for more details of these reactions. rhIL-12 is currently being pursued at Wyeth as an adjuvant for glycoprotein-based vaccines at rhIL-12 levels up to 2 mcg/dose. In the proposed study, rhIL-12 is not being used. The study will assess the safety and adjuvant activity of *IL-12* DNA.

1.5.3 *IL-12* DNA compared to rhIL-12 protein

In comparative studies in mice using *IL-12* DNA therapy and IL-12 protein therapy against various tumors, *IL-12* DNA therapy proved to be as efficient as the IL-12 protein therapy, and induced far less toxic side effects [82]. Preclinical studies conducted using intratumoral *IL-12* DNA therapy showed that this treatment can induce a striking anti-tumor response in various murine tumor models, including melanoma, sarcoma and adenocarcinoma [82-85]. The lack of severe side effects of *IL-12* DNA therapy in this animal model suggested that it may be a safe alternative to IL-12 protein therapy for certain human cancers.

1.6 *IL-12* DNA as a vaccine adjuvant

Modulation of the immune response by coadministration of cytokine plasmids is one of the most promising approaches under investigation aimed at enhancing the immunogenicity of DNA vaccines. Coinjection of cytokine plasmids has been shown by several laboratories to enhance immune responses. Seminal studies of *IL-12* DNA combined with HIV DNA vaccines were performed in rodents by the Weiner Laboratory [34]. These studies demonstrated a dramatic increase in specific CTL activity when a *gag/pol* plasmid or an *env* plasmid was coadministered with an *IL-12* plasmid, as compared with results in animals receiving *env* or *gag/pol* plasmids alone. The molecular adjuvant activity of several Th1 cytokines (GM-CSF, IL-2, IL-12, IL-15, and IL-18) was then evaluated in mice in a subsequent study by the Weiner group [64]. This study revealed that the *IL-12* plasmid was the best driver of MHC-restricted CD8+ CTL activity. Codelivery of *IL-12* DNA and HIV DNA vaccines was also evaluated by the Weiner Laboratory in chimpanzees [86]. DNA immunogens were administered at 500 mcg/plasmids at weeks 0, 5 ,9 and 15. A chimpanzee receiving *gag/pol* and *IL-12* plasmid immunizations exhibited enhanced antigen-specific responses to multiple antigens at multiple timepoints, as compared with a chimpanzee that did not receive *IL-12* plasmid injections.

The use of plasmid *IL-12* as a molecular adjuvant has also been evaluated by other investigators. *IL-12* plasmid was shown to function effectively as an adjuvant in a murine plasmodium model [87], a Hepatitis C murine study [88], influenza virus model [89] and Feline leukemia virus model (FLV) [90]. Other investigators have reported an absence of adjuvant effect for *IL-12* plasmid when coadministered with DNA vaccines for leishmaniasis [91], influenza virus NP [92], and LaCrosse Virus [89]. Wyeth Research has evaluated *IL-12* DNA and *gag* DNA administration in 20 macaques and has not observed any adverse events related to *IL-12* DNA administration. Coadministration of *IL-12* DNA resulted in substantially enhanced cellular immune responses (see Section 3.2.3). In summary, the use of *IL-12* plasmid DNA as a molecular adjuvant has resulted in enhancement of cellular immune responses in a number of studies and has been safe. Taken together, the preclinical data support the evaluation of this adjuvant in human vaccine trials.

2 Study product descriptions

2.1 HIV-1 *gag* DNA vaccine

The GENEVAX® *gag*-2962 plasmid is highly purified, supercoiled, plasmid DNA containing an RNA-optimized truncated *gag* gene (p37) inserted into the DNA plasmid expression vector WLV-001M. The HIV-1 *gag* gene is derived from strain HXB2 but has been RNA-optimized by inactivating inhibitory sequences that allows high level Rev independent expression of the *gag* gene. The WLV-001M plasmid backbone consists of 3 genetic units. The first is a eukaryotic gene expression unit that contains genetic elements from the human cytomegalovirus (hCMV) immediate early promoter/enhancer and the bovine growth hormone (BGH) polyadenylation signal. The second component is a chimeric kanamycin resistance gene *(km^r)* that confers resistance to a limited number of aminoglycosides while enabling selection of bacteria containing the km^r plasmid. The third component is a pUC bacterial origin of replication (ori) that is required for the propagation of the plasmid during fermentation of bacteria.

The GENEVAX® *gag*-2962 plasmid is a second-generation Gag-expressing plasmid that was derived from *gag* plasmid APL-400-047 which was previously evaluated in the AVEG 031 clinical trial (BB-IND 6972) [93-95]. GENEVAX® *gag*-2962 plasmid differs in structure from predecessor plasmid APL-400-047 as described in Table 2-1.

The vaccine is formulated in 30 mM citrate buffer pH 6.5 containing 150 mM NaCl, 0.01% EDTA, and 0.25% bupivacaine-HCl. Studies have shown that formulation of bupivacaine at a 0.25% concentration with plasmid DNA at levels of 800 mcg or higher results in quantitative association (100%) of bupivacaine with DNA [96]. Thus, as the gag, IL-12 and IL-15 DNA vaccine/molecular adjuvants are formulated at 2 mg/mL, bupivacaine in these formulated preparations is complexed with plasmid DNA and is not found in an uncomplexed form as a free molecule.

Table 2-1 Structural differences between *gag* plasmids APL-400-047 and GENEVAX® *gag*-2962 plasmid

APL-400-047	GENEVAX® *gag*-2962 plasmid
Codes for both gag and pol genes from HIV-1 (HXB2 strain)	Codes for RNA optimized truncated gag (p37) from HIV-1 (HXB2 strain)
Presence of RSV enhancer	RSV enhancer has been removed for increased expression
SV40 poly A	BGH poly A
Absence of 5′ untranslated region of IE gene of hCMV	Incorporation of 5′ untranslated sequences from the IE gene of hCMV to enhance the promoter activity
Origin of replication sequence for low-copy plasmid	Single base change in the origin of replication sequence for high copy plasmid

In vitro expression analysis of GENEVAX® *gag*-2962 plasmid in mammalian cells demonstrated a significant enhancement in *gag* expression by GENEVAX® *gag*-2962 plasmid (up to 200-fold) over that observed with APL-400-047. GENEVAX® *gag*-2962 is formulated with 0.25% bupivacaine as a facilitating agent for DNA uptake.

2.2 *IL-15* DNA adjuvant

GENEVAX® *IL-15*-1696 comprises of human *IL-15* gene inserted into the DNA plasmid expression vector WLV001M (WLV backbone construct described above).To minimize the

chances of integration into host cellular DNA, the plasmid does not contain retroviral LTRs or a eukaryotic origin of replication.

The *IL-15* gene has been optimized for high-level expression. Optimization includes removal of *IL-15* signal peptide sequence that contributes to poor translation of *IL-15* transcripts. The *IL-15* signal peptide sequence has been exchanged with that of rhesus *IL-15* and Kozak sequences were included for efficient translation. The expression of *IL-15* gene was determined by measuring secreted protein in cell supernatants of transfected tissue culture cell lines using ELISA and the CTLL2 bioassay. These *IL-15* gene modifications, in addition to removal of 5′ UTR, increased IL-15 expression by 8-10 fold compared to the wild-type construct.

The *IL-15* DNA is formulated in 30 mM citrate buffer pH 6.5 containing 150 mM NaCl, 0.01% EDTA and 0.25% bupivacaine-HCl.

2.3 IL-12 DNA adjuvant

The GENEVAX® *IL-12*-4532 plasmid is a dual promoter expression plasmid consisting of 6259 nucleotides. It contains 2 cistrons that express the genes encoding human IL-12 proteins p35 and p40 under separate regulatory control. The p35 subunit is under the control of the hCMV promoter/enhancer and the SV40 (simian virus 40) polyadenylation signal whereas the p40 subunit is under the control of the SCMV (simian cytomegalovirus) promoter and the BGH (bovine growth hormone) polyadenylation signal. The plasmid backbone of *IL-12*-4532 is identical to that of the GENEVAX® *gag*-2962 plasmid, as described above.

The *IL-12* DNA is formulated in 30 mM citrate buffer pH 6.5 containing 150 mM NaCl, 0.01% EDTA, and 0.25% bupivacaine-HCl.

2.4 Placebo

The placebo for all products in this trial will be sodium chloride injection USP, 0.9%.

3 Preclinical studies

3.1 Preclinical safety studies

3.1.1 Preclinical safety studies of *gag* DNA

Several toxicology studies conducted in macaques, rabbits and mice suggest that *gag* DNA is well tolerated (Section 3.1.5).

During multiple dose administration of SIV *gag* DNA, with or without rhesus *IL-12* DNA, monitoring of hematologic parameters in macaques demonstrated values that were similar to those of controls and that remained within the normal range. In addition, studies in mice and rabbits given multiple doses of *gag* DNA, with or without *IL-12* DNA, showed no product-related clinical signs and no effect on body weight, food consumption, body temperature or clinical pathology parameters. The biodistribution of *gag* DNA was similar to that of the precursor plasmid APL 400-047, which has exhibited a favorable safety profile in a human trial [95]. According to these animal studies, *gag* DNA appears to be well tolerated and elicits immunogenic responses that may be enhanced by coadministration with an adjuvant.

3.1.2 Preclinical safety studies of *gag* DNA and *IL-15* DNA

A summary of the preclinical toxicology studies with *IL-15* DNA is presented in Table 3-1.

Table 3-1 Summary of the toxicology studies with *IL-15* DNA-1696

Study	Regimen	Result
Biodistribution analysis in rabbits of GENEVAX *IL-15*-1696 combined with *gag*-2962<br><br>(This study included another arm in which rabbits received GENEVAX *IL-12*-6285 co-administered with gag-2962. For these results see the GENEVAX *IL-12*-6285 IB, section 4.3.1).	30 rabbits (15/sex) were administered a mixture of GENEVAX *IL-15*-1696 with *gag*-2962 (1500 µg each) in a single bolus IM dose<br><br>Ten rabbits (N=5/sex) were sacrificed at each of three time points: Days 3, 60, and 94.	Tissues collected on Days 3, 60, and 94 post-dosing. TaqMan® real-time PCR assays were used to isolate genomic DNA from bone marrow, brain, heart, injection site skin, quadriceps muscle, kidney, liver, lung, mesenteric lymph node, gonads, spleen and thymus.<br><br><u>Day 3 plasmid levels</u>: highest levels of both plasmids were found in injection site skin (mean >1 x 10^6 copies /µg of genomic DNA). Muscle also contained detectable plasmid levels. Plasmid levels in other tissues near or < assay lower limit of quantitation (LLOQ: 100 copies/µg of genomic DNA) throughout study.<br><br><u>Day 60 plasmid levels</u>: in muscle, blood, kidney lower than the LLOQ, whereas skin had detectable levels but significantly less than at Day 3 (mean levels in males: 6106/7,691 and females: 37,421/47,764 copies *IL-15/gag* plasmids/µg genomic DNA).<br><br><u>Day 94 plasmid levels</u>: decrease of more than 99.9% of mean plasmid (*IL-15*-1696 and *gag*-2962) value in skin and muscle at Day 3 (mean levels in males 1,936/1,576 and females 10,456/9,570 copies *IL-15/gag* plasmids/µg genomic DNA).

Study	Regimen	Result
(N+1 study in rabbits)<br><br>Study to determine the toxicity of multiple IM injections of combined plasmids, *gag*-2962 plus *IL-15*-1696,	1: Control vehicle (bupivacaine).<br>2: *gag*-2962 + *IL-15*-1696 DNA (1500 µg IM per dose for each);<br>3: *IL-15*-1696 DNA alone (1500 µg IM);<br><br>(N=60; 10 per sex per group, 3 groups)<br><br>IM bolus injections on days 1, 22, 43, 64<br><br>Animals euthanized on study days 66 and 94 (2 days and 30 days post final dose).	• All animals survived to scheduled necropsy.<br>• No effects on body weight, food consumption, clinical pathology parameters, organ weights, or macroscopic or microscopic observations.<br>• Purple discoloration around injection site present for 3 days in one male receiving combined plasmids and for 2 days each in two females receiving *IL-15*-1696 alone.<br>• For both erythema and edema, a treatment-related increased incidence and frequency of injection site irritation scores of 1 (very slight formation) and 2 (slight formation) in Group 2 males. These changes were not considered adverse.<br>• No adverse effects of *gag*-2962 or *IL-15*-1696 on any parameter examined. (See section 4.3.2)<br>• Samples tested for antibody against HIV p24 Gag protein showed seroconversion and antibody production against p24 Gag. Group 2 animals showed increase in Gag-specific antibody (294/250 GMT* Day 66/Day 94) over controls (39 GMT).<br><br>*GMT=Geometric mean titer.
Hematologic safety study with SIV *gag* DNA and rhesus *IL-15* DNA in rhesus macaques	1: SIV *gag DNA* + *IL-15* DNA, 1500 µg each<br>2: *SIV gag* DNA alone, 1500 µg<br>3. SIV *gag* DNA+*IL-15 DNA*+*IL-12* DNA, 1500 µg each<br>4: unvaccinated controls<br><br>N=20 (5 per group)<br><br>Dose administered IM at weeks 0, 4, 8, 29.	Analysis of white blood cells, hemoglobin, platelets, red blood cells, % lymphocytes demonstrated mean values similar to controls with no significant changes from baseline over the study course in all treatment groups.<br><br>No significant weight loss and no adverse events related to general appearance, appetite, injection site or behavior were observed.<br><br>1) Detection of anti-IL-15 antibody in rhesus macaques<br>• At week 31, 2 weeks after the fourth immunization, no anti-IL-15 antibody production was observed in groups receiving *IL-15* DNA.<br>2) Serum IL-15 levels post-immunization.<br>• Sera collected 2 wks post each vaccination showed no statistical difference in systemic IL-15 levels between *IL-15* DNA groups and unvaccinated controls.

3.1.3 Safety of SIV *gag* DNA and *IL-15* DNA in Rhesus macaques

This study was designed to assess the adjuvant activity of rhesus *IL-15* DNA when administered with SIV *gag* DNA as well as the hematologic safety of co-administration of these 2 plasmids. For the design and immunogenicity results of this study, which was conducted in rhesus macaques, see Section 3.2.2.

At baseline and throughout the study, the macaques were monitored for weight, hematologic effects and adverse events. No significant weight loss was observed during the course of the study. The CBC panel showed no significant changes in hematocrit, white blood cell count, platelet count, lymphocyte levels, or red blood cell counts. In addition, no adverse events relating to general appearance, appetite, appearance of dosage site, and behavior were reported.

3.1.4 Biodistribution study in rabbits

In a 94-day biodistribution study, 30 rabbits (N=15 per/sex) received a single IM injection of 1500 mcg each of *IL-15*-1696 and GENEVAX *gag*-2962. Tissues were collected at euthanasia on Days 3, 60 and 94 (N=10; 5 per sex at each time point) to determine plasmid distribution. Tissues collected included brain, heart, lung, gonads, muscle and skin from the

injection site, thymus, spleen, bone marrow, lymph nodes, kidney, liver and blood. TaqMan® assays were used to detect and quantify amounts of the three plasmids in the rabbit tissues. Both male and female animals had the highest plasmid levels at Day 3 in the skin from the site of injection (mean > 1 x 10^6 copies/mcg of genomic DNA) (Table 3-2), with muscle also having detectable levels of plasmid. All other tissues had plasmid levels near or less than the assay lower limit of quantification (LLOQ) (10 copies/mcg of genomic DNA) throughout the study. By Day 60, plasmid levels in the muscle for both groups had decreased to lower than the LLOQ in most animals, whereas plasmid levels in the skin were detectable at all timepoints but were significantly less at Day 60 than at Day 3. By Day 94, plasmid levels in the skin and muscle had decreased to more than 99% of the mean value detected in the skin and muscle at Day 3.

Table 3-2 Levels (copy numbers) of GENEVAX *IL-15*-1696 in injection site

Day 3		Day 60		Day 94		% Decrease in copies from Day3 to Day 94	
M	F	M	F	M	F	M	F
12,023,944 (SD:13,568,432)	8,832,279 (SD:13,035,914)	6106 (SD:9065)	37,421 (SD:69,232)	1936 (SD:3146)	10,456 (SD:14,247)	99.98	99.88

Based on these results, there was no apparent plasmid persistence in any tissues examined, except injection site skin, that would indicate a biodistribution safety concern for either GENEVAX *IL-15*-1696 or GENEVAX *gag*-2962. The reason(s) for the observed initial high level of plasmid detected at injection site skin and of its persistence over three months is not completely understood. However, it is thought that this persistence may be related to both technical factors associated with vaccine delivery and to the animal model. The large volume of vaccine used in this study (1.5 mL) combined with a relatively large gauge needle (22 gauge versus 26 gauge used in mouse studies), provided conditions for significant flow- back of inoculum along the needle track and pooling of inoculum under the skin. Deposition of higher than normal levels of plasmid immediately below the skin combined with cytokine activity in the rabbit skin may then have resulted in inoculum being incorporated into rabbit skin cells. It should be noted that rabbit skin differs significantly in structure from human skin, and thus may show different kinetics of degradation. For example, if DNA vaccine were entrapped in the thicker keratinized layers in rabbit skin, plasmid degradation might be expected to proceed more slowly.

3.1.5 Preclinical safety studies of *gag* DNA and *IL-12* DNA

Brief descriptions of the preclinical toxicology studies are provided in Table 3-3. Additional detail is provided in the Investigator's Brochure. Note that *gag* DNA toxicology studies were conducted with plasmid *gag*-3339 which differs from plasmid *gag*-2962 by a single nucleotide difference in the origin of replication (noncoding). Similarly, *IL-12*-6285 was used for the toxicology studies of *IL-12* DNA, and also differs from the study product, *IL-12*-4532, by a single nucleotide change in the noncoding origin of replication region of the plasmid (identical plasmid backbone as that used for *gag*-2962). The single nucleotide difference between the plasmid backbones is associated with increased levels of *gag*-2962 and *IL-12*-4532 plasmid production in bacteria but does not affect levels of *gag* or IL-12 gene expression in eukaryotic cells. Overall, *gag* DNA and *IL-12* DNA have been shown to be safe and well tolerated in mice, rabbits, and nonhuman primates.

Table 3-3 Summary of toxicology studies with human *IL-12* DNA - 6285

Study	Regimen	Methods/ Results
Biodistribution analysis in rabbits of *gag*-2962 combined with GENEVAX *IL-12*-6285<br><br>(NOTE: This study included another arm in which rabbits received GENEVAX *IL-15*-1696 co-administered with gag-2962. For these results see the GENEVAX *IL-15*-1696 IB, section 4.3.1).	30 rabbits (N=15/sex) were administered a mixture of GENEVAX *IL-12*-6285 with *gag*-2962 (1500 µg each) in a single bolus IM dose<br><br>Ten rabbits (N=5/sex) were sacrificed at each of three time-points: Days 3, 60, and 94.	Tissues collected on Days 3, 60, and 94 post-dosing. TaqMan® real-time PCR assays were used to isolate genomic DNA from bone marrow, brain, heart, injection site skin, quadriceps muscle, kidney, liver, lung, mesenteric lymph node, gonads, spleen, and thymus.<br><br>Day 3 plasmid levels: high levels of *gag* and *IL-12* plasmids in injection site skin (*gag* plasmid mean: 6.0 x 10^6 copies /µg of genomic DNA, 10/10 animals; *IL-12* plasmid mean: 2.9 x 10^6 copies /µg of genomic DNA, 10/10 animals). Muscle also contained significant levels of plasmid (*gag* plasmid mean: 1.3 x 10^6 copies /µg of genomic DNA, 10/10 animals; *IL-12* plasmid mean: 1.5 x 10^5 copies /µg of genomic DNA, 10/10 animals). Plasmid levels in other tissues near or < assay LLOQ (100 copies/µg of genomic DNA) throughout study.<br><br>Day 60 plasmid levels: high levels of *gag* and *IL-12* plasmids at injection site skin of female rabbits (*gag* plasmid mean: 3.8 x 10^6 copies /µg of genomic DNA, 5/5 animals; *IL-12* plasmid mean: 1.4 x 10^6 copies /µg of genomic DNA, 5/5 animals). Decreased levels of *gag* and *IL-12* plasmids in injection site of male skin (*gag* plasmid mean: 2.2 x 10^4 copies /µg of genomic DNA, 5/5 animals; IL-12 plasmid mean: 7.8 x 10^3 copies /µg of genomic DNA, 5/5 animals). Muscle contained little or no plasmid (*gag* plasmid mean: 464 copies /µg of genomic DNA, 2/10 animals; *IL-12* plasmid 18 copies /µg of genomic DNA, 1/10 animals).<br><br>Day 94 plasmid levels: Reduced but significant levels of *gag* and *IL-12* plasmids at injection site skin of female rabbits (*gag* plasmid mean: 2.6 x 10^5 copies /µg of genomic DNA, 5/5 animals; *IL-12* plasmid mean: 1.1 x 10^5 copies /µg of genomic DNA, 5/5 animals). Muscle contained no or low levels of plasmid (*gag* plasmid mean: 3.4 x10^3 copies /µg of genomic DNA, 3/10 animals; *IL-12* plasmid 1.1 x 10^3 copies /µg of genomic DNA, 4/10 animals).
		Integration analysis of Day 94 injection site skin (analysis performed by BioReliance). Host cell DNAs from injection site skin from 6 rabbits showing high IL-12 and gag plasmid copy numbers on biodistribution study at Day 94 (IL-12/ gag copies/µg of genomic DNA: 239,217/ 643,736; 174,984/ 401,172; 537, 816/ 1,244,799; 14,481/ 33,083; 12,067/ 33,225; 1900/ 3,973) and from 2 control animal were extracted and purified by alternating TAE and TBE agarose gels (4 gel purifications). Q-PCR analyses on purified DNA were performed to detect possible presence of integrated gag or IL-12 plasmid sequence. Q-PCR results indicated that the six high copy samples for gag and IL-12 plasmids were less than the assay LLOQ (<100 copies/ plasmid), indicating no evidence of plasmid integration in these samples for either *gag* or *IL-12* sequence.
10-wk toxicity study with 4-wk recovery in Crl:CD-1® (ICR) BR mice<br><br>(N=60 per group, 30 M, 30 F)	1: gag-3339 + murine IL-12 DNA (100µg IM per dose for each);<br>2: murine IL-12 DNA alone (100µg IM);<br>3: Control vehicle (bupivacaine).<br>4 IM bolus injections, one dose on days 1, 22, 43, 64)	10 animals per sex per group: euthanized 14 days after 1st dose and 2 days after 4th dose. Remaining 10 in each group: 4-week compound/vehicle-free period.<br><br>No test article-related clinical signs; no test article-related effects on body weight, food consumption, clinical pathology parameters, organ weight, or macroscopic or microscopic changes. Injection site changes in controls and treated animals related to injection procedure or vehicle components.

Study	Regimen	Methods/ Results
10-wk toxicity study with 4-wk recovery in male (M) and female (F) New Zealand white rabbits<br>(N = 60; 20 per group, 10 M, 10 F)	1: GENEVAX IL-12-6285+ gag-3339 (1500µg per dose, each plasmid),<br>2: GENEVAX IL-12-6285 alone (1500µg per dose),<br>3: control vehicle (bupivacaine)<br>4 IM bolus injections, one dose on days 1, 22, 43, 64)	(5 per sex per group: euthanized:<br>2 days post final dose; 30 days post final dose to assess compound/ vehicle-free recovery)<br>No test article-related clinical signs; no effects on body weight, food consumption, body temperature; no test article-related clinical pathology abnormalities, no organ weight, macroscopic or microscopic changes; injection site irritation (lymphohistiocytic inflammation, slight to marked hemorrhage, muscle necrosis) in all groups related to injection procedure or vehicle components; inflammation and muscle necrosis resolved at recovery necropsy 4 weeks post final dose.
Hematologic safety study with different dose levels of SIV *gag* DNA and rhesus *IL-12* DNA in male rhesus macaques<br>N=25 (5 per group)	1: *gag*+*IL-12* DNAs, 1.5 mg each<br>2: gag alone, 1.5 mg<br>3: gag+IL-12 DNAs, 5.0 mg each<br>4: gag DNA alone, 5.0 mg<br>5: unvaccinated controls<br>Dose administered IM at weeks 0, 4 and 8.	Analysis of white blood cells, hemoglobin, platelets, red blood cells, % lymphocytes demonstrated mean values similar to controls and within normal range.
Hematologic safety study with SIV *gag* DNA and rhesus *IL-12* DNA and/or rhesus *IL-15* DNA in rhesus macaques	1. *SIV gag* DNA alone, 1500 µg<br>2. SIV *gag* DNA + *IL-12* DNA, 1500 µg each<br>3. SIV *gag* DNA + *IL-15* DNA, 1500 µg each<br>4. SIV *gag* DNA + *IL-12* DNA + *IL-15* DNA, 1500 µg each<br>5. controls<br>N=25 (5 per group)<br>Dose administered IM at weeks 0, 4, 8, 29.	Analysis of white blood cells, hemoglobin, platelets, red blood cells, % lymphocytes demonstrated mean values similar to controls with no significant changes from baseline over the study course in all treatment groups.<br>No significant weight loss and no adverse events related to general appearance, appetite, injection site, or behavior were observed.<br>No detection of anti-IL-15 antibody in any group of macaques receiving DNA vaccine immunization

3.1.5.1 Toxicology study of murine *IL-12* DNA alone or in combination with HIV-1 *gag* DNA in mice

This study was designed to assess the toxicity of 4 intramuscular bolus injections of murine *IL-12* DNA alone (100 mcg), or combined with *gag* DNA (100 mcg), in mice. Administration was well tolerated. There were no product-related clinical signs and no product-related effects on body weight, food consumption, clinical pathology parameters, organ weight or macroscopic or microscopic changes. Injection site reactions were not different between the *IL-12* DNA, *gag* DNA plus *IL-12* DNA, and control (vehicle) injections.

3.1.5.2 Toxicology study of *IL-12* DNA alone or in combination with HIV-1 *gag* DNA in New Zealand white rabbits

The purpose of this study was to evaluate the toxicity of 4 intramuscular injections of *IL-12* DNA, 1500 mcg, administered alone or in combination with *gag* DNA, 1500 mcg, in New Zealand white rabbits. Administration of *IL-12* DNA alone or *gag* DNA/*IL-12* DNA was well tolerated. There were no product-related effects on body weight, food consumption or body temperature and no indication of injection site irritation at the recovery necropsy evaluation (day 30 post final dose). There were no product-related effects on clinical pathology

parameters, and no organ weight or macroscopic or microscopic changes were noted at 30 days post final dose.

3.1.5.3 Biodistribution analysis of *IL-12* DNA and *gag* DNA in rabbits

Rabbits received a single intramuscular injection of 1500 mcg each of *IL-12* DNA and *gag* DNA. Tissues were collected on Days 2, 30, 60 and 94 to determine plasmid distribution. Tissues were sent to Exploratory Drug Safety in Andover, Massachusetts, USA, where a previously developed TaqMan® assay was used to detect and quantify amounts of *IL-12* plasmid and *gag* plasmid in the rabbit tissues.

On Day 3 post-dosing, high levels of *gag* and *IL-12* plasmids were observed in injection site skin (*gag* plasmid mean: 6.0×10^6 copies/mcg of genomic DNA, 10/10 animals; *IL-12* plasmid mean: 2.9×10^6 copies/mcg of genomic DNA, 10/10 animals). Muscle also contained significant levels of plasmid (*gag* plasmid mean: 1.3×10^6 copies/mcg of genomic DNA, 10/10 animals; *IL-12* plasmid mean: 1.5×10^5 copies/mcg of genomic DNA, 10/10 animals). Plasmid levels in other tissues were near or less than the assay LLOQ (100 copies/mcg of genomic DNA) throughout study.

On Day 60 high levels of *gag* and *IL-12* plasmids were observed at the injection site skin of female rabbits only (*gag* plasmid mean: 3.8×10^6 copies/mcg of genomic DNA, 5/5 animals; *IL-12* plasmid mean: 1.4×10^6 copies/mcg of genomic DNA, 5/5 animals). In the injection site skin of male rabbits, decreased levels of *gag* and *IL-12* plasmids were observed (*gag* plasmid mean: 2.2×10^4 copies/mcg of genomic DNA, 5/5 animals; *IL-12* plasmid mean: 7.8 x 10^3 copies/mcg of genomic DNA, 5/5 animals). Muscle from males and females contained little or no plasmid (*gag* plasmid mean: 464 copies/mcg of genomic DNA, 2/10 animals; *IL-12* plasmid 18 copies/mcg of genomic DNA, 1/10 animals).

By Day 94 post dosing, reduced levels of *gag* and *IL-12* plasmids were observed at the injection site skin of female rabbits (*gag* plasmid mean: 2.6×10^5 copies/mcg of genomic DNA, 5/5 animals; *IL-12* plasmid mean: 1.1×10^5 copies/mcg of genomic DNA, 5/5 animals). In a few animals of both sexes, muscle contained no or low levels of plasmids (*gag* plasmid mean: 3.4×10^3 copies/mcg of genomic DNA, 3/10 animals; *IL-12* plasmid 1.1×10^3 copies/mcg of genomic DNA, 4/10 animals). Muscle of 7 animals contained no *gag* plasmid, while 6 animals had no *IL-12* plasmid in muscle. Results indicated that *gag* and *IL-12* plasmids did not persist at significant levels in the tissues examined, except for injection site skin.

3.1.5.4 Integration analysis of *IL-12* DNA and *gag* DNA in rabbits

Discussions were subsequently held with the FDA regarding possible concerns over plasmid integration in injection site skin samples possessing high copy number. The FDA recommended that Wyeth conduct an integration analysis of the high copy number injection site skin samples at Day 94 and further recommended specific tissue samples for analysis. Wyeth subsequently contracted with BioReliance to perform the integration analysis. The analysis evaluated host cell DNA extracted from Day 94 injection site skin obtained from six rabbit showing high *IL-12/gag* plasmid copy numbers (239,217/ 643,736; 174,984/ 401,172; 537, 816/ 1,244,799; 14,481/ 33,083; 12,067/ 33,225; 1900/ 3,973 *IL-12/gag* plasmid copies/µg of genomic DNA) and from skin obtained from two negative control rabbits. Host cell DNAs were extracted and then purified by alternating TAE and TBE agarose gels by 4 gel purifications. Two independent Q-PCR analyses on purified DNA were then performed to detect possible presence of integrated *gag* or *IL-12* plasmid sequence. Result s of the analysis failed to produce evidence for integration of either *gag* or *IL-12* plasmid sequence as *gag* and

IL-12 Q-PCR values from the six high copy test samples were less than the assay LLOQ (<100 copies).

3.1.5.5 Safety of rhesus *IL-12* DNA plasmid in rhesus macaques

To date 20 rhesus macaques have been coimmunized with either 15 mg (three IM, 5 mg doses at 0, 4 and 8 weeks) or 4.5 mg (three 1.5 mg doses at 0, 4 and 8 weeks) of rhesus *IL-12* DNA and no obvious adverse effects have been observed. IL-12 serum levels did not increase following plasmid administration and anti-IL-12 antibodies were not detected following vaccination. In addition, white blood cell count, hemoglobin, platelet count, red blood cell count and percent of lymphocytes were monitored biweekly for 12 weeks, and at Weeks 18 and 20 post inoculation. All evaluations revealed values within the normal range over the period studied.

3.2 Preclinical immunogenicity studies

3.2.1 In vivo expression and immunogenicity studies in mice

3.2.1.1 Study in Balb/c mice: IL-15-*1696* with or without HIV gag-*3339*

Results of a study conducted in Balb/C mice (Table 3-4) indicate that *IL-15* DNA was able to increase cellular and humoral immune response in mice immunized with HIV *gag-3339* DNA, as measured by IFN-γ ELISpot and ELISA assay. The augmented immune responses (Figure 3-1 and Figure 3-2) were comparable to immune responses observed with *IL-12* plus *gag* DNA.

Table 3-4 Design of study to evaluate *IL-15* DNA as adjuvant in mice*

Group #	Vaccine DNA	Dose	Days
1	HIV *gag +IL-15*	100 mcg, each	0, 21, 42
2	HIV *gag+IL-12*	100 mcg, each	0, 21, 42
3	HIV *gag*	100 mcg	0, 21, 42
4	Backbone (WLV-001)	None	0, 21, 42

*All vaccines are given at final volume of 100 mcl as 2 intramuscular injections into the calf muscles.

Figure 3-1 Splenocytes isolated at day 56 (2 weeks post 3rd immunization) were tested by Gag-specific IFN-γ ELISpot analysis. Units on y-axis are SFC/million splenocytes. Data are representative of two separate experiments, as represented by Table 3-4.

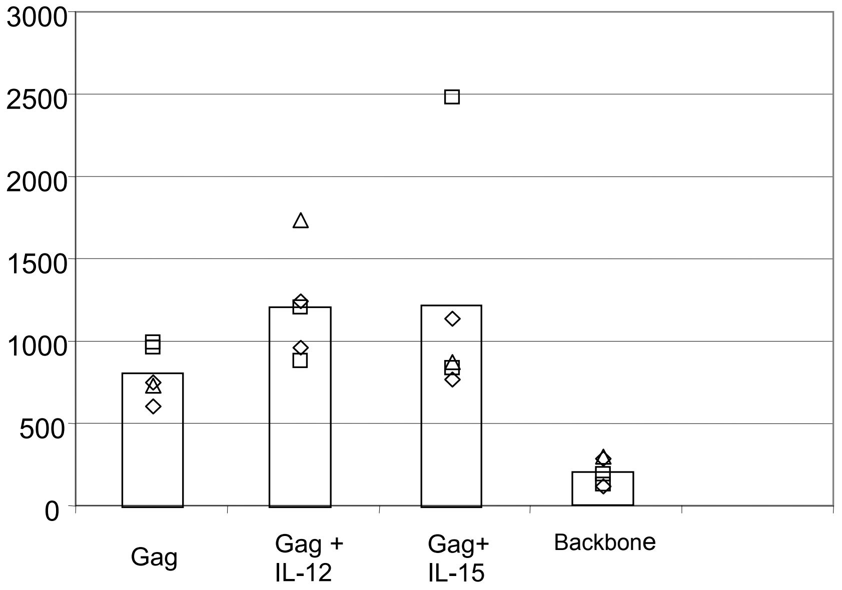

Figure 3-2 Serum samples on day 56 (2 weeks post 3- immunization) were tested for Gag-specific IgG response. Units on y-axis are Log Mean Titers. Data are representative of 2 separate experiments.

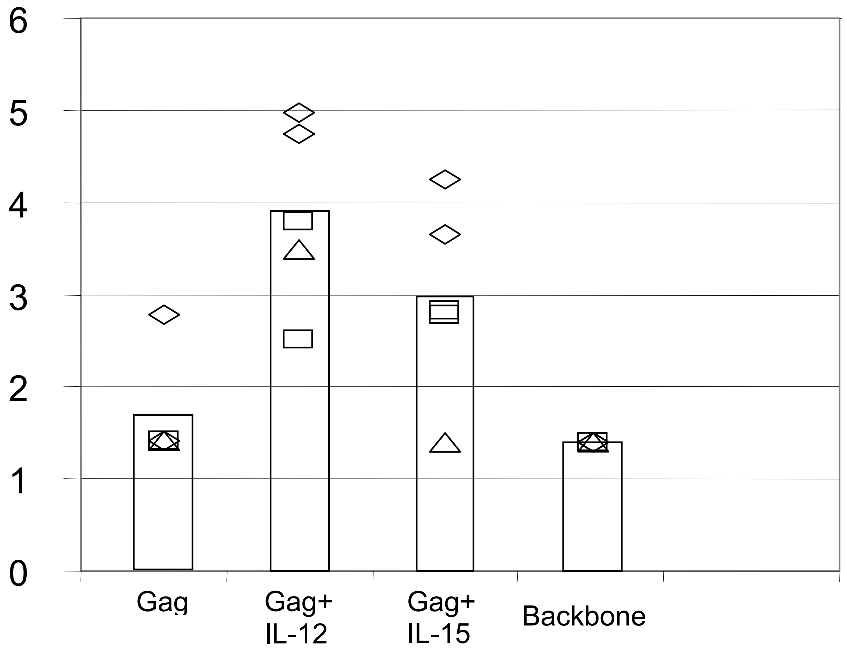

3.2.1.2 Kinetics of IL-15 Expression in Mice

A study was conducted in Balb/c mice to evaluate the kinetics of IL-15 and Gag expression after single IM administration of GENEVAX *IL-15*-1696 administered alone or co-administered with *gag*-2962.

The study design is presented in Table 3-5.

Table 3-5 Study design of expression kinetic study in Balb/c mice: GENEVAX *IL-15*-1696 plus GENEVAX *gag*-2962, an HIV DNA complexed with bupivacaine

Groups[a]	Site of Injection	Concentration of Plasmid	Days for bleed and tissue collection	Plasmid Injection Volume *IL-15*-1696 @ 2mg/ml *gag*-2962 @ 2mg/ml
IL-15-1696 DNA	IM-same leg	200 mcg	Days 2, 4, 6, 10,15, 30, & 60	100 µl
gag-2962 DNA	IM-same leg	200 mcg	As above	100 µl
IL-15-1696 DNA + *gag* -2962 DNA	IM-same leg	200 mcg 200 mcg	As above	100 µl 100 µl
backbone control	IM-same leg	200 mcg	As above	100 µl
Naïve	------	-------	Day 30	0 µl

[a] Seventy (70) Balb/c mice in each treatment group (groups 1-4); ten untreated controls (group 5).
In groups 1-4, 5 male and 5 female mice sacrificed on days of tissue collection. Group 5 animals sacrificed on day 30.

Serum and muscle samples were collected on the days of sacrifice, following IM injection in quadriceps of 200 mcg of *IL-15*-1696 administered alone or with *gag*-2962 or of backbone construct (WLV-001). Serum and muscle samples were collected on day 30 only in untreated controls (Group 5). A standard ELISA was used to quantify IL-15 protein.

The results are shown in Figure 3-3. These results demonstrate that administration of *IL-15*-1696 DNA alone or with *gag*-2962 DNA results in transient expression of IL-15 protein, with peak expression at 4 to 6 days post vaccination. IL-15 was not detected in muscle by Day 15 or at later time-points. No difference in level of IL-15 expression in muscle was observed between males and females.

IL-15 was not detected in serum samples taken over the 60-day time period (data not shown). That increased levels of IL-15 are not detected in blood at any time point suggests that administration of *IL-15*-1696 is likely to result in IL-15-induced effects locally but not at the systemic level.

Figure 3-3 Expression of IL-15 in mouse muscle for groups injected with *IL-15*-1696 DNA, *IL-15*-1696 + *gag*-2962 DNA or backbone control.

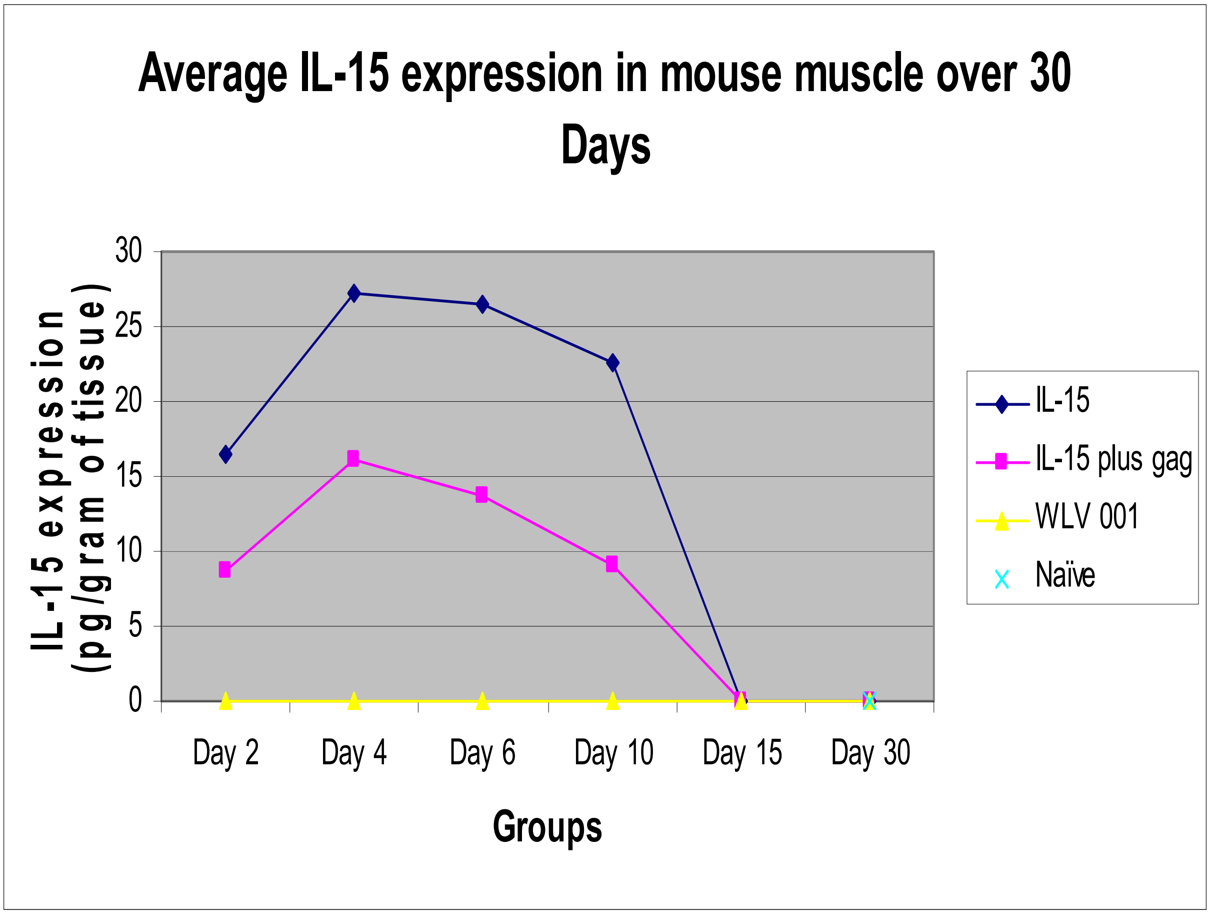

3.2.2 Rhesus *IL-15* DNA: augmentation of SIV *gag* p37-specific immune responses in rhesus macaques

A study conducted in rhesus macaques (Table 3-6) demonstrated that rhesus *IL-15* plasmid was able to increase cellular and humoral immune response in macaques immunized with SIV *gag* p37, as measured by IFN-γ ELISpot and ELISA assays (Figure 3-4 and Figure 3-5). Significantly enhanced cellular responses were observed after the fourth immunization whereas a trend for enhanced antibody responses was observed after the third immunization. Groups of 5 monkeys immunized on the same schedule with *IL-12* DNA + *gag* DNA vaccine or a combined *IL-15* DNA, *IL-12* DNA and *gag* DNA vaccine at 1.5 mg for each vaccine component. The *IL-15/IL-12/gag* DNA group showed enhanced humoral immune responses relative to responses observed in groups immunized with *gag* DNA combined with either individual cytokine, but no increase in cellular immune response was observed in the *IL-15/IL-12/gag* DNA group above that observed with individual cytokine adjuvants (data not shown). No adverse events were associated with administration of any of the *gag* DNA/molecular adjuvant combinations.

Table 3-6 Experimental design of study evaluating rhesus *IL-15* DNA administered as an adjuvant with SIV *gag* DNA

Group#	No. Animals	Vaccine products*	Dose	Route	No. Vaccines
1	5	SIV *gag* DNA + Rhesus *IL-15* DNA + backbone plasmid	1.5mg 1.5mg 1.5mg	IM	4
2	5	SIV *gag* DNA + backbone plasmid	1.5mg 1.5mg	IM	4
3	5	None	None	IM	4

*The vaccines for each group were pre-mixed and administered IM into deltoid and quadriceps muscles in equal volumes of two 0.9-ml injections for a total of 1.8 mL of vaccine per animal. Animals were vaccinated at weeks 0, 4, 8 and 29.

Figure 3-4 SIV Gag-specific IFN-γ ELISpot assay

PBMC were collected at various time-points and stimulated overnight with 15 mer peptides overlapping by 11 amino acids encompassing the entire length of the SIV Gag protein. 2×10^5 cells were deposited into each well. The data are reported as spot-forming cells (SFC)/10^6 cells. Error bars are standard error. Vaccine was administered at weeks 0, 4, 8, and 29.

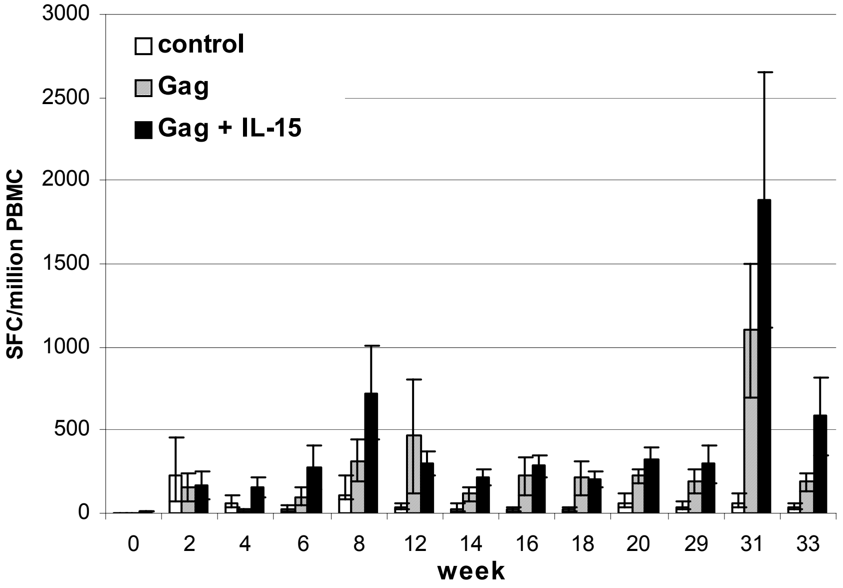

Figure 3-5 SIV p27Gag-specific ELISA assay

Sera were collected at various time-points and tested on ELISA plates coated with 20 ng/well SIV p27 Gag protein. The endpoint titer per week was reported as the last titer when the O.D.450 was higher than the O.D.450 of the pre-immune sera (±3 standard deviations). Error bars are standard error. Vaccine was administered at weeks 0, 4, 8, and 29.

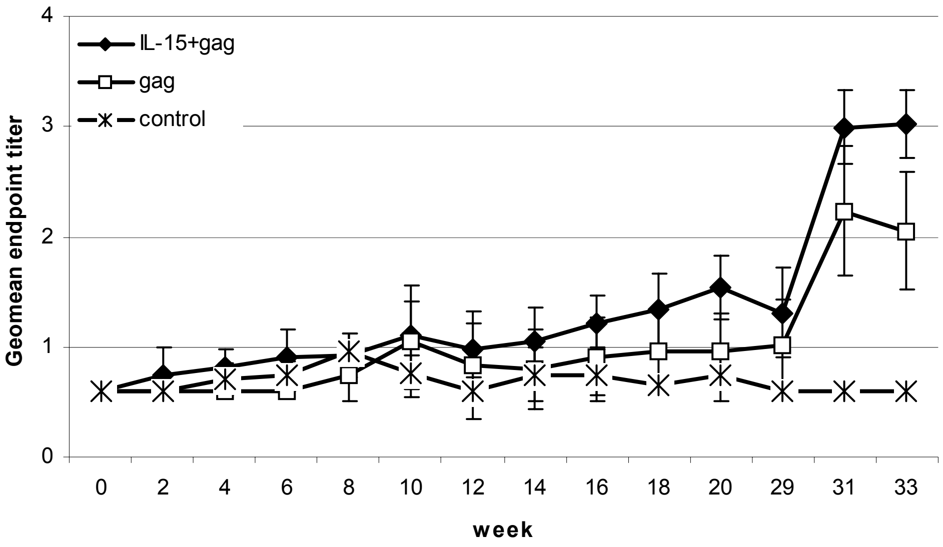

The above animals subsequently received intravenous SHIV 89.6P challenge (30 MID50). Results following challenge have indicated that animals receiving *IL-15* and *gag* plasmids did not show enhanced rates of virus replication or accelerated rates of CD4 decline (data not shown).

3.2.3 Preclinical immunogenicity studies of SIV *gag* DNA alone and with rhesus *IL-12* DNA in rhesus macaques

Four groups of 5 rhesus macaques were vaccinated intramuscularly (0, 4, 8 weeks) with 1.5 or 5 mg of SIV *gag* DNA, either with or without rhesus *IL-12* DNA at 1.5 and 5 mg, respectively. Robust (>1:1000) serum antibody responses to SIV Gag were observed in 8/10 animals receiving *IL-12* DNA (high 5 mg or low 1.5 mg dose) compared to 3/10 animals in the groups that did not receive *IL-12* DNA. IFN-γ ELISpot assay to overlapping SIV *gag* peptide pools was also performed to measure cellular immune responses. Moderate numbers of IFN-γ expressing cells (mean 300 SFU/million cells) were detected in macaques vaccinated with 1.5 or 5 mg SIV *gag* DNA alone by Week 10, compared to high numbers of IFN-γ expressing cells (mean 1400 SFU/million cells) in groups receiving *IL-12* DNA (see Table 3-7). IFN-γ ELISpot responses were similar with either dose level of *IL-12* DNA.

Table 3-7 IFN-γ ELISpot responses (per 10^6 cells) in monkeys immunized with SIV *gag* DNA and rhesus *IL-12* DNA.

	Week					
Vaccine	2	4	6	8	10	20
1.5 mg SIV *gag* DNA + 1.5 mg IL-12 DNA	41	65	768	718	2098	928
	158	40	405	1200	425	89
	135	9	690	193	2948	948
	62	23	1436	450	1115	249
	69	9	500	0	135	456
Mean	93	29	760	292	1344	534
1.5 mg SIV *gag* DNA	11	23	228	160	278	50
	13	0	119	128	240	251
	25	3	10	0	28	19
	9	0	110	63	321	101
	79	36	25	15	411	178
Mean	27	12	98	73	256	120
5 mg SIV *gag* DNA + 5 mg IL-12 DNA	129	404	1190	473	1796	978
	1	118	534	150	538	159
	65	168	966	333	1373	499
	0	348	640	83	725	768
	196	289	1175	218	2733	431
Mean	78	265	901	251	1433	567
5 mg SIV *gag* DNA	10	24	43	0	392	71
	101	54	36	43	255	32
	115	495	421	110	726	281
	15	5	336	305	271	207
	25	236	233	0	48	142
Mean	53	163	214	92	338	147
Controls	36	0	0	15	68	0
	45	33	0	0	85	29
	113	96	0	88	13	5
	88	9	0	8	0	19
	46	0	0	0	0	4
Mean	66	28	0	22	33	11

The humoral and cellular immune responses elicited by a RNA optimized SIV *gag* DNA construct were substantially enhanced by coimmunization with rhesus *IL-12* expressing plasmid DNA.

The above animals subsequently received intravenous SHIV 89.6P challenge (30 MID50). Preliminary results following challenge have indicated that animals receiving *IL-12* and *gag* plasmids demonstrated decreased levels of peak viremia, lower viral setpoints, and slower rate of CD4+ T cell decline (data not shown).

4 Clinical studies

4.1 HIV-1 *gag* DNA vaccine

The HIV-1 *gag* DNA vaccine (GENEVAX® *gag*-2962) will be evaluated in humans for the first time in humans in HVTN 060.

4.2 *IL-15* DNA adjuvant

No previous clinical studies of *IL-15*-1696 DNA have been performed.

4.3 *IL-12* DNA adjuvant

The *IL-12* DNA vaccine adjuvant (GENEVAX® *IL-12*-4532) has not yet been evaluated in humans. This will be evaluated for the first time in humans in HVTN 060 with HIV-1 *gag* DNA vaccine. All available safety data from HVTN 060, Part A will be evaluated prior to initiating the booster vaccination series in Part B of HVTN 063 using HIV-1 *gag* DNA vaccine with *IL-12* DNA. The dose for use in Part B of this study will be the maximum tolerated dose from HVTN 060, Part A.

5 Summary

5.1 Rationale for trial design

IL-15 cytokine has been shown to be critical for induction of CD8 memory responses. However, prominent CD8 immune responses may not be evident immediately following HIV DNA vaccine priming with *IL-15* DNA adjuvant, but may rather be more evident several months later, especially following booster vaccinations. This trial will evaluate prime/boost regimens using *gag* + *IL-15* DNA as priming agents in combination with two different boosting agents. The two cohorts in Part B will assess the relative capacity of *gag* DNA + *IL-12* DNA and *gag* DNA + *IL-15* DNA to boost individuals primed with *gag* DNA + *IL-15* DNA. Although other HIV DNA vaccines have not shown significant promise when examined as boosting agents in other studies, addition of cytokine adjuvants to our DNA vaccine may well provide the desired adjuvant activity, as observed in the SIV *gag* DNA + *IL-15* primate study described in Section 3.1.3. Use of DNA vaccines as priming and booster agents would have great advantages over mixed modality prime/boost regimens, where immunity to recombinant vectors may prevent their repeated use.

As this is the first trial to investigate plasmid *IL-15*, the trial begins with a cautious dose escalation of *IL-15* DNA in Part A. There is a *gag* alone arm to judge whether there are safety issues with this particular *gag* plasmid. The 10:2 randomization ratio is standard for Part A of HVTN Phase I studies, an approach developed by SCHARP for the HVTN. This design is powered to observe safety and toxicity outcomes if they are likely (prevalence ≥20%). Two placebo recipients are included in each group to maintain blinding and the combined placebo groups will provide limited safety information.

Part B represents a regimen optimization/selection design to evaluate the effects of a *gag* DNA+*IL-12* DNA boost compared to boosts with the same plasmids. Each group consists of 30 vaccinees and 6 placebo recipients, who are included in each group to maintain blinding. The pooled placebo groups (N = 12) will provide safety information and will be used to calculate false positive rates for immunogenicity assays. Statistical properties for safety and immunogenicity analyses are provided in Section 9.

5.2 HVTN 060 and timing of initiation of HVTN 063

The HVTN 060 study, entitled "A Phase I clinical trial to evaluate the safety and immunogenicity of an HIV-1 *gag* DNA vaccine with or without *IL-12* DNA adjuvant, boosted with homologous plasmids or with HIV CTL multiepitope peptide vaccine / RC529-SE plus GM-CSF, in healthy, HIV-1 uninfected adult participants," began enrollment in parallel with HVTN 063. Safety data from Part A of HVTN 060 will be evaluated prior to administration of *gag* + *IL-12* DNA boosting in Part B of HVTN 063. The temporal relationship of the present protocol to HVTN 060 is outlined in Figure 1-1.

5.3 Plans for future product development and testing

The current HIV-1 *gag* DNA vaccine represents a prototype plasmid vaccine and as such, contains only the p37 portion of the Gag molecule. The next generation plasmid vaccine will contain the full *gag* gene sequence as well as several other HIV genes (e.g., *pol, nef, env, vif, tat*).

Based on results of HVTN 060 and this study, a decision will be made regarding the optimal cytokine adjuvant(s) to pursue in Phase I/II studies with the expanded HIV DNA vaccines.

This page is intentionally blank

STUDY DESIGN

6 Study objectives

6.1 Primary objectives

Part A

- To evaluate the safety and tolerability of intramuscular administration of HIV-1 *gag* DNA vaccine, alone; and of HIV-1 *gag* DNA vaccine plus *IL-15* DNA (at escalating doses of 100 mcg, 500 mcg, and 1500 mcg)

Part B

- To evaluate the safety and tolerability of intramuscular administration of HIV-1 *gag* DNA vaccine plus *IL-15* DNA as a priming series, followed by boost vaccinations with HIV-1 *gag* DNA vaccine plus *IL-15* DNA or HIV-1 *gag* DNA vaccine plus *IL-12* DNA.

6.2 Secondary objectives

Part A

- To evaluate the immunogenicity of intramuscular administration of HIV-1 *gag* DNA vaccine.
- To evaluate the contribution of *IL-15* DNA (at escalating doses of 100 mcg, 500 mcg, and 1500 mcg) to the immunogenicity of HIV-1 *gag* DNA vaccine.

Part B

- To evaluate the immunogenicity of intramuscular administration of HIV-1 *gag* DNA vaccine plus *IL-15* DNA as a priming series, followed by boost vaccinations with HIV-1 *gag* DNA vaccine plus *IL-15* DNA, or HIV-1 *gag* DNA vaccine plus *IL-12* DNA.

Parts A & B

- To evaluate the social impact of participation in this trial.

7 Study type, study population, and eligibility criteria

The study is a Phase I multicenter, randomized, placebo controlled, double-blind trial to evaluate the safety and immunogenicity of HIV-1 *gag* DNA alone or with *IL-15* DNA, boosted with HIV-1 *gag* DNA +*IL-15* DNA vaccine, or HIV-1 *gag* DNA + *IL-12* DNA vaccine. Participants will be healthy HIV-1-uninfected (seronegative) adults who comprehend the purpose of the study and have provided written informed consent. Participants will be recruited and screened; those determined to be eligible, based on the inclusion and exclusion criteria (see Table 7-1 and Table 7-2), will be enrolled in the study and followed for a period of 12 months (Part A) or 18 months (Part B).

In Part A, participants and investigators are not blinded by groups, but within each group, they are blinded to whether they receive placebo (normal saline) or vaccine. In Part B, participants and investigators are blinded as to group as well as placebo/vaccine assignment. In Parts A and B, participants will receive vaccinations at Days 0, 28, and 84 (Months 0, 1 and 3). Booster vaccinations for participants in Part B will be administered at Days 168 and 273 (Months 6 and 9). All vaccinations will be administered by a single intramuscular injection in the deltoid using needle/syringe in the outpatient setting.

See Table 7-1 for inclusion criteria and Table 7-2 for exclusion criteria. Final eligibility determination will depend on results of laboratory tests, medical history, physical examinations, and answers to the self-administered and/or interview questions.

See Section 12.1 for screening procedures.

Table 7-1 Study inclusion criteria

Investigators should always use good clinical judgment in considering a volunteer's overall fitness for trial participation. Some volunteers might not be appropriate for enrollment even if they meet all inclusion/exclusion criteria because medical, psychiatric, or social conditions might make evaluation of safety and/or immunogenicity difficult.

General

Age: 18 to 50 years

Access to a participating HVTU and willingness to be followed for the planned duration of the study

Assessment of understanding: Complete a questionnaire prior to first vaccination; verbalize understanding of all questions answered incorrectly

Willingness to receive HIV test results

Informed consent: Be able and willing to provide informed consent

Health: Be in good general health as shown by medical history, physical exam, and screening laboratory tests performed within 56 days of enrollment

Laboratory

Hemoglobin: ≥ sex-specific institutional lower limit of normal and at least 11.0 g/dL for women, 13.0 g/dL for men

WBC count = 3,300 to 12,000 cells/mm^3

Total lymphocyte count ≥ 800 cells/mm^3

Remaining differential either within institutional normal range or accompanied by site physician approval

Platelets = 125,000 to 550,000/mm^3

Chemistry panel

Part A: ALT, AST, alkaline phosphatase, and creatinine values do not exceed institutional upper limit of normal, and CPK value does not exceed 2 times the institutional upper limit of normal;

Part B: ALT and AST do not exceed 1.25 times the institutional upper limit of normal, creatinine does not exceed institutional upper limit of normal, and CPK value does not exceed 2 times the institutional upper limit of normal.

Negative HIV blood test. US participants are required to have a negative FDA-approved ELISA test. Non-US sites will use locally available and locally approved assays.

Negative Hepatitis B surface antigen (HBsAg)

Negative anti–Hepatitis C virus antibodies (anti-HCV), or negative HCV PCR if the anti-HCV is positive

Normal urine:

- Negative urine glucose, and
- Negative or trace urine protein, and
- Negative or trace urine hemoglobin (if trace hemoglobin is present on dipstick, a microscopic urinalysis is required to exclude participants with counts greater than the institutional normal range)

Additional inclusion criteria for female participants

Negative serum or urine β-HCG pregnancy test performed on the day of initial vaccination prior to vaccination

Reproductive status: a female participant must:

- agree to consistently use contraception for at least 21 days prior to enrollment until the last protocol visit, for sexual activity that could lead to pregnancy. Contraception is defined as using any of the following methods:
  - condoms (male or female) with or without a spermicide
  - diaphragm or cervical cap with spermicide
  - intrauterine device (IUD)
  - hormonal contraception
  - successful vasectomy in the male partner (considered successful if a woman reports that a male partner has [1] microscopic documentation of azoospermia, or [2] a vasectomy more than 2 years ago with no resultant pregnancy despite sexual activity post-vasectomy)
- or not be of reproductive potential, such as having reached menopause (no menses for one year) or having undergone hysterectomy, bilateral oophorectomy, or tubal ligation
- agree not to seek pregnancy through alternative methods such as artificial insemination or in vitro fertilization until last protocol visit

Table 7-2 Study exclusion criteria

Participant has received any of the following substances:

HIV vaccine(s) in a prior HIV vaccine trial. *For potential participants who have received control/placebo in an HIV vaccine trial, documentation of the identity of the study control/placebo must be provided to the Protocol Safety Team, who will determine eligibility on a case-by-case basis.*

Immunosuppressive medications within 168 days before first vaccination, e.g., oral/parenteral corticosteroids, and/or cytotoxic medications. *Not excluded: A participant using any of the following is not excluded: (1) corticosteroid nasal spray for allergic rhinitis; (2) topical corticosteroids for mild, uncomplicated dermatitis*

Blood products within 120 days before first vaccination

Immunoglobulin within 60 days before first vaccination

Live attenuated vaccines within 30 days before first vaccination

Investigational research agents within 30 days before first vaccination

Medically indicated subunit or killed vaccines, e.g., influenza within 14 days, pneumococcal within 14 days, or allergy treatment with antigen injections within 30 days prior to initial study vaccine administration

Current anti-TB prophylaxis or therapy

Participant has a clinically significant medical condition, physical examination findings, clinically significant abnormal laboratory results, or past medical history with clinically significant implications for current health. A clinically significant condition or process includes but is not limited to:

- *a process that would affect the immune response*
- *a process that would require medication that affects the immune response*
- *any contraindication to repeated injections or blood draws*
- *a condition that requires active medical intervention or monitoring to avert grave danger to the participant's health or well-being during the study period*
- *a condition or process in which signs or symptoms could be confused with reactions to vaccine*
- *any condition specifically listed among the exclusion criteria below*

Any medical, psychiatric, or social condition, or occupational or other responsibility that, in the judgment of the investigator, would interfere with, or serve as a contraindication to, protocol adherence, assessment of safety or reactogenicity, or a participant's ability to give informed consent

Diagnosed allergy to amide-type local anesthetics [(bupivacaine (Marcaine), lidocaine (Xylocaine), mepivacaine (Polocaine/Carbocaine), etidocaine (Duranest), prilocaine (Citanest, EMLA cream)].

Serious adverse reactions to vaccines including anaphylaxis and related symptoms such as hives, respiratory difficulty, angioedema, and/or abdominal pain. *Not excluded: A participant who had a non-anaphylactic adverse reaction to pertussis vaccine as a child.*

Autoimmune disease

Immunodeficiency

Active syphilis infection. *Not excluded: Syphilis fully treated over six months ago.*

Asthma that is not mild and well controlled. Exclude a participant who:

- generally uses a bronchodilator (short acting beta2 agonist) 7 days a week, or
- In the past year, has (any of the following):
  - had >1 exacerbation of symptoms treated with oral steroids (Note: oral steroid use is exclusionary within 168 days before first vaccination)
  - used moderate to high dose inhaled corticosteroids (e.g. more than the equivalent of 250 mcg fluticasone; 400 mcg budesonide; 500 mcg beclomethasone; or 1000 mcg triamcinolone/flunisolide, as a daily dose) or theophylline
  - needed emergent care, urgent care, hospitalization or intubation for asthma

Diabetes mellitus type I or type II, including cases controlled with diet alone. *Not excluded: history of isolated gestational diabetes.*

Thyroid disease or thyroidectomy requiring medication during the last 12 months

Angioedema within the last 3 years if episodes are considered serious or have required medication within the last 2 years

Hypertension:

- If a person has been diagnosed with hypertension during screening or previously, exclude for hypertension that is not well controlled.
  Well controlled hypertension is defined as blood pressure consistently ≤ 140 mm Hg systolic and ≤ 90 mm Hg diastolic, with or without medication, with only isolated, brief instances of higher readings, that may not exceed 150 mm Hg systolic or 100 mm Hg diastolic. For these participants, blood pressure must be ≤140 mm Hg systolic and ≤ 90 mm Hg diastolic at enrollment.
- If a person has NOT been diagnosed with hypertension during screening or previously, exclude for:
  - Systolic blood pressure ≥ 150 mm Hg at enrollment
  - Diastolic blood pressure ≥ 100 mm Hg at enrollment

BMI≥40; or BMI ≥ 35 with more than one of the following: age >45, BPs>140/90, current smoker, known hyperlipidemia. A person with BMI over 35 should be asked about other contributory health risks

Bleeding disorder diagnosed by a doctor, e.g., factor deficiency, coagulopathy, or platelet disorder requiring special precautions

Malignancy *Not excluded: A participant with a surgical excision and subsequent observation period that in the investigator's estimation has a reasonable assurance of sustained cure and/or is unlikely to recur during the period of the study.*

Seizure disorder *Not excluded: A participant with a history of seizure who has not required medications or had seizure for 3 years.*

Asplenia: any condition resulting in the removal of the spleen or absence of a functional spleen

Psychiatric condition that precludes compliance with the protocol. Specifically excluded are persons with any of the following:

- psychoses within the past 3 years
- ongoing risk for suicide
- history of suicide attempt or gesture within the past 3 years

Additional exclusion criteria for female participants: Participant is pregnant and/or breast feeding, or planning to become pregnant during the period of study participation

8 Safety and immunogenicity evaluations

8.1 Considerations for trial start

No considerations for trial start have been identified.

8.2 Initial safety evaluation

For Part A, enrollment for Groups 1 and 2 (i.e., enrollment across all participating HVTUs) will be restricted to a maximum of 1 participant per day per group and restricted to U.S. sites until 5 participants per group have been enrolled. The HVTN 063 Protocol Safety Review Team will review the safety and reactogenicity data reported for the first 72 hours post-vaccination on each of these 10 participants and will determine whether it is safe to proceed with full enrollment in these groups. If enrollment proceeds in the US following the safety review, then enrollment in these groups may also be initiated at sites outside the US.

Enrollment for each subsequent dose group in Part A (Group 3, then Group 4) across all participating HVTUs will be restricted to a maximum of 1 participant per day and restricted to U.S. sites until 5 participants have been enrolled. The PSRT will review the safety and reactogenicity data reported for the first 72 hours after the first vaccination on each of these 5 participants and will determine whether it is safe to proceed with full enrollment in that group. If enrollment proceeds in the US following the safety review, then enrollment in these groups may also be initiated at sites outside the US.

8.3 Safety considerations for dose escalation

For Part A, in addition to monitoring participant safety throughout the study period, the PSRT will review cumulative safety data available on all participants in each group (vaccinees and placebo combined) up to and including the 2 week visit after the second vaccination to determine whether dose escalation may occur. The PSRT may consult with the HVTN Safety Monitoring Board (SMB) on an ad hoc basis for these evaluations. See Section 14.8.5 for additional information.

8.4 Safety evaluation for moving from Part A to Part B

In addition to monitoring participant safety throughout the study period, the PSRT will review all cumulative safety data available from Part A up to and including the 2 week visit after the third vaccination in Group 4. Based on the assessment of these safety data, the PSRT will determine the appropriateness of moving to Part B as well as the recommended (maximum tolerated) dose to be used in Part B, in consultation with the HVTN SMB and the FDA. The HVTN SMB may perform an additional ad hoc unblinded review of this safety data to facilitate this process. The FDA will be provided with the data for their review.

Initiation of the booster vaccination series (Visit 8) in Part B of this study will begin only after the HIV-1 *gag* DNA vaccine and *IL-12* DNA adjuvant have been shown to be well tolerated in Part A of the initial Phase I trial (HVTN 060). At a minimum, this review will include safety data up to and including the 2 week post-vaccination visit after the second dose of vaccine with the maximum tolerated dose of *IL-12* DNA. The dose of *IL-12* DNA that will be used in this trial will be the maximum tolerated dose from HVTN 060.

8.5 Other safety considerations

8.5.1 Exclusion of participants with allergy to amide type local anesthetics

The HIV-1 *gag* DNA vaccine and *IL-12* DNA adjuvant are formulated in bupivacaine. Individuals with allergy to amide type local anesthetics (based on a self-reported medical history) will be excluded from enrollment in this study.

8.5.2 Monitoring of autoimmune illness

As IL-15 protein has been associated with effects on the mechanisms of self-tolerance, careful assessment for autoimmune illness will be monitored during the study. An autoimmune symptoms assessment will be performed at each visit.

8.6 Distinguishing intercurrent HIV infection from vaccine-induced positive serology

The study product may elicit an antibody response to HIV proteins. Therefore, vaccine-induced positive serology may occur in this study. Several precautionary measures will be taken to distinguish intercurrent HIV infection from vaccine-induced positive serology:

- Participants will be counseled frequently during the trial on avoidance of HIV infection.
- Participants will be counseled on the risks of seeking HIV testing outside of the network during study participation, and discouraged from doing so.
- Participants will have clinical evaluations at visits specified in Appendices E or F. Signs or symptoms of an acute HIV infection syndrome, an intercurrent illness consistent with HIV-1 infection, or probable HIV exposure would prompt a diagnostic work-up per the standard HVTN algorithm to determine HIV infection.
- Diagnostic HIV-1 ELISAs will be performed from blood draws at multiple time points throughout the trial (see Appendices C and D).
- The HVTN Laboratory Program is responsible for in-study diagnostic testing. For non-US sites, local labs may perform HIV diagnostic algorithms (following HVTN SOP) with pre-approval from the HVTN Laboratory Operations Division.
- If intercurrent HIV-1 infection is suspected or positive test results are observed post-vaccination, the Laboratory Program or approved diagnostic laboratory will proceed with the HVTN algorithm to distinguish vaccine-induced antibody responses from actual HIV infection.
- Continued follow-up will identify subsequent HIV infections or address concerns in participants whose HIV-1 ELISA is positive or indeterminate at the end of the study. All participants who have positive or indeterminate HIV-1 serology at the last study visit (as measured by the Abbott HIV 1, 2 kit or other standard anti-HIV antibody screening test used by blood banks) will be offered follow-up HIV-1 diagnostic testing (HIV-1 ELISA, Western blot, PCR) periodically and free of charge as medically/socially indicated (approximately every 6 months). This follow-up will be available until the ELISA/Western blot pattern no longer yields positive or indeterminate results or until HIV infection is confirmed.
- Potential participants identified as being HIV infected during screening and participants who become HIV infected during the study will be referred for medical

treatment and management of the HIV infection. These individuals will also be referred to appropriate ongoing clinical trials or observational studies.

8.7 Immunogenicity evaluations

The ability of the vaccine to induce humoral responses and/or epitope-specific CD8+ and CD4+ T cell responses will be evaluated by the methods described below. For all assays, cryopreserved specimens from additional time points of immunological interest, as indicated in Appendices C and D, may be tested if positive responses are detected at the primary immunogenicity time points.

One of the goals in assaying additional time points (if positive responses are detected at primary immunogenicity time points) would be to compare the level of memory response at later time points since IL-15 has been shown to contribute to the durability of T cell responses. Another goal in assaying additional time points (if positive responses are detected at primary immunogenicity time points) would be to examine the whether effector T cells generated in the presence of IL-12 DNA adjuvant improves memory T cell responses.

8.7.1 Humoral immunogenicity studies (HVTN)

8.7.1.1 Binding antibodies by ELISA

Binding antibodies to commercially available Gag will be assessed at the HVTN Laboratory Operations Division by ELISA using single serum dilutions (1/50 or 1/100) on samples from all study participants taken at baseline and at visits designated in Appendices C and D. Any of the time points that yield positive results, defined as an optical density (OD) of ≥ 0.2, in the initial ELISA may be subject to endpoint titration ELISA employing 6 (2-7-fold) serial dilutions of serum beginning at a 1/50 or 1/100. Additional ELISA will be performed in all participants using cryopreserved samples if a significant number of positive responses are observed at the primary time points (see Appendix C and Appendix D).

8.7.2 Cellular immunogenicity studies for Part A (HVTN)

8.7.2.1 IFN-γ ELISpot

Bulk T cell responses will be assessed by IFN-γ ELISpot using cryopreserved peripheral blood mononuclear cells (PBMC) stimulated overnight with synthetic peptide pools that span the proteins encoded by the vaccine constructs. ELISpot assays will be performed at baseline and at the two weeks post final vaccination visit (see Appendix C). Additional time points may be assayed if positive responses are observed at the primary immunogenicity time point (see Appendix C). Responses will be reported as number of spot forming cells per 10^6 cells/well recognizing any specific peptide pool.

8.7.3 Intracellular cytokine staining (ICS)

Flow cytometry will be used to examine HIV-specific CD4+ and CD8+ T cell responses using ICS following stimulation with synthetic HIV peptides that span the proteins encoded by the vaccine construct. ICS assays will be performed at baseline and at the two weeks post final vaccination visit (see Appendix C). Additional time points may be assayed if positive responses are observed at the primary immunogenicity time point (see Appendix C). Responses will be reported as percentages of CD4+ or CD8+ T cells recognizing any specific peptide pool.

8.7.4 Cellular immunogenicity studies for Part B (HVTN)

8.7.4.1 IFN-γ ELISpot

Bulk T cell responses will be assessed by IFN-γ ELISpot using cryopreserved PBMC stimulated overnight with synthetic peptide pools that span the proteins encoded by the vaccine constructs. ELISpot assays will be performed at baseline and at two weeks post final (fifth) vaccination (see Appendix D). Additional time points may be assayed if positive responses are observed at the primary immunogenicity time point (see Appendix D). Responses will be reported as number of spot forming cells per 10^6 cells/well recognizing any specific peptide pool.

8.7.4.2 Intracellular cytokine staining (ICS)

Flow cytometry will be used to examine HIV-specific CD4+ and CD8+ T cell responses using ICS following stimulation with synthetic HIV peptides that span the proteins encoded by the vaccine constructs. ICS assays will be performed at baseline and two weeks post final (fifth) vaccinations (see Appendix D). Additional time points may be assayed if positive responses are observed at the primary immunogenicity time point (see Appendix D). Responses will be reported as percentages of CD4+ or CD8+ T cells recognizing any specific peptide pool.

8.8 Ancillary studies

8.8.1 Assay development (HVTN)

Cryopreserved samples may be used to perform additional ELISpot and ICS assays to support standardization and validation of these assays, and to evaluate additional immunological assays of interest. These assays may include, but are not limited to, fine epitope mapping by flow cytometry or ELISpot, or flow cytometric tetramer analysis.

8.8.2 T-cell receptor repertoire studies for Part B (Vanderbilt University)

HIV epitope-specific T-cell receptor (TCR) repertoires will be evaluated in vaccinees with detectable CD8+ T-cell responses. Tetramer-positive T cells from cryopreserved PBMC will be sorted and subjected to TCR analysis by PCR [97]. The breadth of TCR repertoires in vaccinees will be compared to matched, HLA-typed controls of HIV+ subjects at different stages of HIV disease. These subjects include a cohort of subjects with control of viremia (10 subjects with viral loads <50 copies off ART, and an additional 20 subjects with viral loads <1,000 copies off ART), actively followed by Dr. Kalams at Vanderbilt University Medical Center. These subjects have signed an informed consent, approved by the Vanderbilt University IRB, for an observational study (NIH-funded) to identify potential correlates of control of HIV viremia.

8.8.3 Ancillary studies (Wyeth)

The assays listed below will be performed on samples obtained at the visit following the last vaccination. Responses at earlier time points will be assessed if responses are observed at the time point following the last vaccination.

8.8.3.1 Humoral immunogenicity studies for Part A

Evaluation of binding antibody production against IL-15 will be performed on cryopreserved samples stored at the Central Specimen Repository (CSR) as shown in Appendix C.

8.8.3.2 Humoral immunogenicity studies for Part B

Evaluation of binding antibody production against IL-15 will be performed on cryopreserved samples stored at the CSR as shown in Appendix D.

Evaluation of binding antibody production against IL-12 will be performed on cryopreserved samples stored at the CSR as shown in Appendix D.

8.8.3.3 Cellular immunogenicity studies

Tetramer-binding assays will be performed at Wyeth on cryopreserved PBMC stored at the CSR as shown in Appendix C for Part A and Appendix D for Part B. These analyses will use a panel of tetramers that recognize CTL epitopes encoded by the vaccine constructs in the context of the appropriate HLA molecule.

8.9 HLA typing

Molecular HLA typing may be performed on enrolled participants using cryopreserved PBMC collected at baseline, initially in participants who demonstrate vaccine-induced T-cell responses at post-vaccination time points. Other participants (including placebo recipients) may be HLA-typed to support future studies of immunological interest at the discretion of the protocol chair and the HVTN Laboratory Program. These assays may include, but are not limited to, fine epitope mapping by flow cytometry or ELISpot, or flow cytometric tetramer analysis.

9 Statistical considerations

9.1 Overview

This study is a multicenter, randomized, placebo-controlled, double-blind trial. The data analysis will evaluate safety and immunogenicity data of the study groups. The study design blinds participants and site staff performing the clinical assessment to vaccine or placebo arms of each group. In Part B, participants and clinic staff are blinded to group as well.

9.2 Objectives

The primary objective of this trial concerns safety; the secondary objectives concern immunogenicity and social impacts.

9.3 Endpoints

9.3.1 Safety

Assessment of product safety will include clinical observation and monitoring of hematological and chemical parameters. Safety will be evaluated by monitoring participants for local and systemic adverse reactions after each injection and for 12 months in Part A and 18 months in Part B after the first injection. Section 14 describes the safety monitoring plan and reports.

The following parameters will be assessed:

- Local reactogenicity signs and symptoms
- Systemic reactogenicity signs and symptoms
- Laboratory measures of safety
- Adverse and serious adverse events

9.3.2 Immunogenicity

Secondary immunogenicity endpoints are:

- HIV-specific cellular responses assessed by IFN-γ ELISpot
- HIV-specific cellular responses as assessed by intracellular cytokine staining (ICS)
- HIV binding antibody by ELISA

9.3.3 Social impacts

Social impact variables include any negative experiences or problems the participant experienced due to his/her participation in this study. The following social impacts will be followed during the course of the study: social, travel, work, school, health care, life insurance, health insurance, housing, military and any additional impacts identified by a participant.

9.4 Accrual and sample size

Recruitment will target 120 healthy, HIV-uninfected adult participants.

Part A will consist of 4 groups. Groups 1 and 2 will be enrolled simultaneously with participants randomized to receive either *gag* DNA or placebo (Group 1), or *gag* DNA plus *IL-15* DNA (100 mcg) or placebo (Group 2) in a 10:2 ratio. Groups 3 and 4 will proceed sequentially according to dose escalation rules outlined in Sections 8.3 and 14.8.5. Groups 3 and 4 will each enroll participants randomized to receive *gag* DNA plus *IL-15* DNA (Group 3: 500 mcg, then Group 4: 1500 mcg) or placebo in a 10:2 ratio per group.

Part B will consist of 2 groups (Groups 5 and 7) and will follow Part A according to rules outlined in Section 8.4. Groups 5 and 7 will be enrolled simultaneously and randomized to receive either *gag* DNA plus *IL-15* DNA (at the maximum tolerated dose from Part A) or placebo for priming vaccinations followed by boost vaccinations of *gag* DNA + *IL-15* DNA (Group 5), *gag* DNA + *IL-12* DNA (maximum tolerated dose in HVTN 060) (Group 7) or placebo, in a (30:6) ratio for vaccine to placebo.

Since enrollment is concurrent with receiving the first study vaccination, all participants will provide some safety data hence sample size calculations for safety in Section 9.4.1 are based on the target sample sizes. However this is not true of the immunogenicity data, therefore the sample size calculations in Section 9.4.2 account for 10% of enrolled participants having missing data for the primary immunogenicity endpoints, based on previous HVTN and AVEG studies.

9.4.1 Sample size calculations for safety

The goal of the safety evaluation for this study is to identify safety concerns associated with administration. Sample size calculations for safety are expressed in terms of the ability to detect rare events, e.g., serious adverse events (SAEs).

The ability of the study to identify serious adverse experiences can be expressed by the maximum true rate of events that would be unlikely to be observed and the minimum true rate of events that would very likely be observed.

Specifically, for each vaccine arm of Part A (n=10), there is a 90% chance of observing at least 1 serious adverse experience if the true rate of such an event is at least 21%; there is a 90% chance that we would not observe any serious adverse experiences if the true rate was no more than 1%.

For safety analyses, for each vaccine arm of Part B, there is a 90% chance of observing at least 1 serious adverse experience if the true rate of such an event is at least 8%; there is a 90% chance that we would not observe any serious adverse experiences if the true rate was no more than 0.35%.

Probabilities of observing 0 or 2 or more serious adverse experiences among groups of size 10 and 30 are presented in Table 9-1 for a range of possible true event rates. These calculations provide a more complete picture of the sensitivity of this study design to identify potential safety problems with the vaccine.

Table 9-1 Probability of response for different safety scenarios

True event rate (%)	Pr(0/10)	Pr(2+/10)	Pr(0/30)	Pr(2+/30)
1.0	90.4	0.4	74.0	3.6
3.5	70.0	4.6	34.3	28.3
5.0	59.9	8.6	21.5	44.6
10.0	34.9	26.4	4.2	81.6
20.0	10.7	62.4	0.1	98.9
30.0	2.8	85.1	<0.1	>99.9
40.0	0.6	95.4	<0.1	>99.9
50.0	<0.1	98.9	<0.1	>99.9
60.0	<0.1	99.8	<0.1	>99.9

If none of the 90 participants receiving the gag DNA plus IL-15 DNA vaccine priming sequence experiences a safety event (combining Part A and B groups), the exact 95% 2-sided upper confidence bound for the rate of such events overall is 4.0%. Restricted to any of the vaccine arms (n=10) in Part A, the exact 95% 2-sided upper confidence bound for this rate is 31%. Restricted to any of the vaccine arms (n=30) in Part B, the exact 95% 2-sided upper confidence bound for this rate is 12%.

9.4.2 Sample size calculations for immunogenicity

In Part A, the precision with which response rates can be estimated, based on a sample of 10 vaccines, is limited. The standard error of the estimated response rates depends on the true underlying response rate but can be bounded by 0.16 ($=\sqrt{0.5 \times 0.5 / n}$). Thus, the width of a 95% confidence interval, using a normal approximation method to calculate width, for the response rate in any one arm will be no greater than 0.63 (i.e., ±1.96 x 0.16).

For comparisons of Part B groups, Table 9-2 below displays exact 95% confidence intervals for several possible observed rates of response for groups of size 27. The n of 27 assumes a 10% loss of data.

Table 9-2 Exact 95% 2-sided confidence intervals for response rates based on observing a particular rate of responses in the vaccinees (n=27).

Observed response rate(%)	Confidence interval (n=27)
51.9	[31.9, 71.3]
59.3	[38.8, 77.6]
70.4	[49.8, 86.2]
81.5	[61.9, 93.7]
88.9	[70.8, 97.6]

There is limited power for a formal comparison of immunogenicity response rates between vaccine groups of size n=27 and, hence, formal comparisons are not listed in the objectives. For illustration, we include power calculations for comparison of response rates between vaccine groups (n=27) that can be distinguished with statistical power of 80% and 90% for an exact 2-sided test with a Type I error rate of 0.05 in Table 9-3. Note the sizes of the differences the trial is powered to detect are very large.

Table 9-3 Minimum detectable differences in response rates between 2 groups (of size 27)

True response rate 1st group (%)	Minimum detectable response rate 2nd group (%)	
	80% power	90% power
10	47	52
20	60	66
30	72	77
40	80	85
50	88	92
60	94	97
70	>99	>99

9.5 Statistical analysis

All data from enrolled participants will be analyzed according to the initial randomization assignment regardless of how many vaccinations they received. Since enrollment is concurrent with receiving the first vaccination, all participants will have received one vaccination and therefore will provide some safety data. The analysis is intent-to-treat; however, individuals who are randomized but not enrolled do not contribute data and hence are excluded. Because of blinding and the brief length of time between randomization and enrollment— typically no more than 4 working days, according to the *HVTN Manual of Operations* (HVTN MOP) (Study Operations >Enrollment >Randomization)—very few such individuals are expected.

Analyses for primary endpoints will be performed in SAS. All other descriptive and inferential statistical analyses will be performed using SAS, S-Plus, and/or R statistical software.

No formal multiple comparison adjustments will be employed for safety or immunogenicity endpoints that address specified scientific questions (e.g., humoral- and cellular-based endpoints). However, multiple measurements of a specific type of immune response may be treated as a collection of hypotheses that requires a multiplicity adjustment. For example, determination of cellular immune responses to several different HIV-1 peptide pools as measured by the IFN-γ ELISpot assay may entail a multiplicity adjustment to account for the multiple peptide pools considered.

9.5.1 Analysis variables

The analysis variables consist of baseline variables, safety variables, immunogenicity variables, and social impact variables for primary and secondary objective analyses.

9.5.2 Baseline comparability

Groups will be compared for baseline characteristics including demographics and laboratory measurements, using descriptive statistics.

9.5.3 Safety analysis

Reactogenicity

The number and percentage of participants experiencing each type of reactogenicity sign or symptom will be tabulated by severity and vaccine regimen. For a given sign or symptom, each participant's reactogenicity will be counted once under the maximum severity for all injection visits.

Adverse experiences

Adverse experiences will be analyzed using MedDRA preferred terms. The number and percentage of participants experiencing each specific adverse experience will be tabulated by severity and by relationship to treatment. For the calculations in these tables, each participant's adverse experience will be counted once under the maximum severity or the strongest recorded causal relationship to treatment.

A complete listing of expedited adverse events reported to the DAIDS Safety Office including severity, relationship to treatment, onset, duration and outcome.

Local laboratory values

Boxplots of local laboratory values by treatment will be generated for baseline values and for values measured during the course of the study. Each boxplot will show the 1st quartile, the median, and the 3rd quartile. Outliers, or values outside the boxplot, will also be plotted. If appropriate, horizontal lines representing boundaries for abnormal values will be plotted.

9.5.4 Immunogenicity analysis

The statistical analysis for immunogenicity will employ the intent-to-treat principle, i.e., all data from enrolled participants will be used according to the initial randomization assignment regardless of how many injections they received. The only exception will be to exclude data from HIV-infected participants at or post infection. Thus, for HIV-infected participants, only immunogenicity data from samples known to be drawn prior to HIV infection will be included in the analysis.

If assay data are qualitative (i.e., positive or negative) then analyses will be performed by tabulating the frequency of positive response for each assay by vaccine regimen at each time point that an assessment is performed. Binomial response rates will be presented with their corresponding exact 95% confidence interval estimates. For Parts A and B, crude rates for each vaccine group will be calculated. The combined placebo groups of Part B (N=12) will be used to estimate the false positive rate. Although, because of the lack of precision in this estimate, net response rates will not be calculated for the vaccine groups.

To compare the response rates of any two vaccine arms, a significant difference will be declared if the 2-sided 95% confidence interval for the difference in response rates between 2 groups excludes 0. If assays are run at multiple time points, the probability of observing at least one positive response by a given time point and the probability of observing more than one response by a given time point will be estimated, with corresponding confidence intervals, for each vaccine arm using maximum likelihood based methods [60]. Missing responses will be assumed to be missing at random, i.e., conditional on the observed data the missingness is independent of the unobserved responses.

For continuous assay variables, overall differences between groups at a specific time point will be tested by a 2-sample t-test if the data appear to be normally distributed, or by utilizing the nonparametric Wilcoxon rank sum test if the data are not normally distributed. If a portion of the measurements are censored below the assay quantification limit, then the Gehan-Wilcoxon test will be employed. More sophisticated analyses employing repeated measures methodology (for example, repeated measures ANOVA or generalized estimating equations) may be utilized to incorporate immune responses over several time points. However, inference from such analyses would be limited by the small sample size of this study. All statistical tests will be 2-sided and will be considered statistically significant if $p \leq 0.05$. Graphical descriptions of the longitudinal immune responses will also be given.

Some immunologic assays have underlying continuous or count-type readout that is often dichotomized into responder/nonresponder categories. For these assays, graphical and tabular summaries of the underlying distributions will be made. These summaries may be performed on transformed data (e.g., log transformation) to better satisfy assumptions of symmetry and homoscedasticity. If group comparisons in these underlying distributions reveal that differences are best summarized as a shift in the location of the distribution, then results will be presented in the form of group means (or medians) with associated confidence intervals and statistical tests for differences between groups as described above. If group comparisons in these underlying distributions reveal that differences are best summarized by a mixture model (i.e., responder and nonresponder subgroups are clearly identifiable) then results will be presented in the form of response rates with associated confidence intervals and statistical tests as described above.

9.5.4.1 Missing data

If the probability of missing immunogenicity measurements depends on either covariates or on the immunogenicity outcomes of participants, then the methods described above may give biased inferences and point estimates. If a substantial amount of immunogenicity data are missing (at least 1 value missing from more than 20% of participants), then secondary analyses of the immunogenicity endpoints will be conducted using methods that relax the missing completely at random assumption to a missing at random assumption. For a univariate binary and quantitative outcome, respectively, a generalized linear model with a binomial or normal error distribution will be used for estimation and testing. For assessing repeated immunogenicity measurements, linear mixed effects models will be used. The models will be fit using maximum likelihood methods, and will include as covariates all available baseline predictors of the missing outcomes. The longitudinal models will also include all observed immunogenicity data.

9.5.5 Social impact analysis

Social impacts will be tabulated by type of event and impact on quality of life. The number and percentage of participants experiencing each type of social impact will also be tabulated by impact on quality of life. For this calculation multiple events of the same type for a participant will be counted once under the maximum impact for all post-vaccination visits.

In addition, a listing will be generated of all participants who experienced a major disturbance of their quality of life due to study participation. The listing includes all social impacts experienced by these participants, descriptions of each impact, duration, impact on quality of life, actions taken by the participant and staff, and whether or not there was a resolution.

9.5.6 Analyses prior to end of study

Safety

Unblinded interim analyses of safety data are prepared at regular intervals for the HVTN Safety Monitoring Board (SMB). Ad hoc interim safety reports may also be prepared for SMB review at the request of the Protocol Safety Review Team.

Immunogenicity

An unblinded statistical analysis of an immunogenicity endpoint may be performed when the Laboratory Program has completed testing at least 80% of samples from the primary immunogenicity visit and all participants have completed the visit.

9.6 Randomization of treatment assignments

The randomization sequence will be obtained by computer-generated random numbers and provided to each HVTU by the SDMC using the procedures described in the HVTN MOP (Study Operations>Enrollment>Randomization). At each institution, the pharmacist with primary responsibility for drug dispensing is charged with maintaining security of the randomization list. The randomization will be done in blocks to ensure balance across groups.

This page is intentionally blank

STUDY OPERATIONS

10 Protocol conduct

The protocol will be conducted according to standard HVTN policies and procedures specified in the HVTN MOP (Study Operations), including procedures for the following:

- Protocol registration, activation and implementation
- Informed consent, screening, enrollment
- Clinical and safety assessments
- Safety monitoring and reporting
- Data collection and documentation
- Study follow-up and close-out
- Unblinding of staff and participants
- Quality control
- Protocol monitoring and compliance
- Advocacy and assistance through local and governmental activities to participants regarding social harms associated with the vaccine trial
- Risk reduction counseling
- Outside testing and belief questionnaire

Any policies or procedures that vary from HVTN standards or require additional instructions will be described in the *HVTN 063 Study Specific Procedures* (e.g., instructions for randomization specific to this study).

11 Informed consent

Informed consent is the essential process of ensuring that participants fully understand what will and may happen to them while participating in a research study. The HVTN informed consent form documents that a participant (1) understands the potential risks, benefits, and alternatives to participation, and (2) is willing to participate in an HVTN study. Informed consent is not confined to the signing of the consent form; it also includes all written or verbal study information HVTU staff discuss with the participant, before and during the trial. HVTU staff will obtain informed consent of participants according to the HVTN policies and procedures specified in the HVTN MOP (Study Operations>Informed Consent).

An HVTU may employ recruitment efforts prior to the participant consenting. Some HVTUs use a telephone script to pre-screen people before they come to the clinic for a full screening visit. Participants must sign a screening or protocol-specific consent before any procedures to determine eligibility are performed. HVTUs must submit recruitment and pre-screening materials to IRBs/IECs for human subjects review.

11.1 Screening consent form

Some HVTUs have approval from their local Institutional Review Board (IRB) and/or Independent Ethics Committee (IEC) to use a general screening consent form that allows screening for an unspecified HIV vaccine trial. In this way, HVTU staff can continually screen potential participants, and when needed, proceed quickly to obtain protocol-specific enrollment consent. Sites conducting IRB/IEC–approved general screening or pre-screening may use the results from this screening for determining eligibility in this protocol, provided the tests are conducted within the time period specified in the eligibility criteria.

11.2 Protocol-specific consent form

The protocol-specific consent form describes the study products to be used and all aspects of protocol participation, including screening and enrollment procedures. A sample protocol-specific consent form is located in Appendices A and B for Part A and B respectively.

Each HVTU is responsible for developing a protocol-specific consent form for local use, based on the sample protocol-specific consent form in Appendix A. The consent form must be developed in accordance with local IRB/IEC requirements and the principles of informed consent as described in Title 45, Code of Federal Regulations (CFR) Part 46 and Title 21 CFR, Part 50, and in the International Conference on Harmonisation (ICH) Guideline 4.8.10. It must be approved by all responsible ethical review bodies before any participants are consented for the study.

The sample forms in Appendices A and B include interspersed instructions for developing specific content.

The DAIDS Regulatory Compliance Center (RCC) Protocol Registration Office will review all site-specific informed consent forms and approve them for use according to DAIDS policies. The study cannot be initiated at a site until the site is fully registered with the DAIDS RCC Protocol Registration Office and has received written notification of protocol activation.

11.3 Assessment of understanding

Study staff should ensure that participants fully understand the study before enrolling them. This involves reviewing the informed consent form with the participant, allowing time to reflect on the procedures and issues presented, and answering all questions completely.

An Assessment of Understanding is used to document the participant's understanding of key concepts in an HIV vaccine trial.

When the Assessment of Understanding is used to document the participant's full understanding before the enrollment consent is signed, most IRBs/IECs will require that the participant have been told about the assessment and signed a screening consent. This is because a site may not initiate study procedures without the participant's consent, and administering the Assessment of Understanding can be viewed as a study procedure. The consent process (including the use of the Assessment of Understanding) should be explained thoroughly to the IRB/IEC, whose recommendations should be followed.

The participant must complete the Assessment of Understanding—with assistance of staff, if necessary, in reading and understanding the questions and responses—before first vaccination. Participants should verbalize understanding of all questions answered incorrectly. This process, and the participant's understanding of the key concepts, should be documented in source documentation at the site.

Informed consent does not end with the signing of the consent form. Periodically throughout the study, key study concepts should be reviewed with the participant. At each study visit, HVTU staff should consider reviewing the procedures and requirements for that visit and for the remaining visits.

12 Procedures

Participants are considered to be enrolled only upon receipt of the first study vaccination at Day 0.

HVTU and HVTN Laboratory Program staff will conduct pre-enrollment and post-enrollment study procedures according to HVTN procedures as specified in the HVTN MOP (Study Operations). Any procedures which vary from the HVTN standard will be defined in the *HVTN 063 Study Specific Procedures*.

Pre-enrollment and post-enrollment procedures are performed on all participants (unless otherwise noted) at the time points indicated in Appendices E and F, using the blood draw volumes specified in Appendices C and D.

12.1 Pre-enrollment procedures

Screening assessments and other pre-enrollment procedures are listed in Table 12-1. Timepoints are specified in Appendices E and F.

Screening procedures are done to determine eligibility and to provide a baseline for comparison of safety data. Screening may occur over the course of several contacts/visits up to and including Day 0 before vaccination. All inclusion and exclusion criteria must be assessed within 56 days before enrollment, unless otherwise specified in Section 7.

The time interval between randomization and enrollment should not exceed 4 working days, as defined in the HVTN MOP (Study Operations). Subsequently, the HVTU registers the participant by scheduling the Day 0 visit (enrollment) via the web-based randomization system, and requests the randomization assignment.

Table 12-1 Pre-enrollment procedures

Screening assessments			
Clinical assessments	Local lab assessments		HIV infection assessments
Medical history	Pregnancy test (females)	Chemistry panel:	HIV screening test
Complete physical exam	Urine dipstick/urinalysis	ALT	
Abbreviated physical exam	CBC with differential	AST	
Concomitant medications	Platelet count	Alkaline phosphatase	
Autoimmune symptoms assessment	Syphilis	Creatinine	
	Hepatitis B	CPK	
	Hepatitis C		
	T-cell subsets		

Other pre-enrollment procedures	
Screening informed consent (if applicable)	Behavioral risk assessment
Protocol informed consent	Risk reduction counseling
Assessment of understanding	Pregnancy prevention counseling
Specimen collection	HIV pre- and post-test counseling
Obtain demographics	Participant randomization
Confirm eligibility	

12.2 Post-enrollment procedures

Safety assessments, immunogenicity determinations, and other post-enrollment procedures are listed in Table 12-2.

Table 12-2 Post-enrollment procedures

Safety assessments			
Clinical assessments	Local lab assessments		HIV infection assessments
Abbreviated physical exam	Pregnancy test (females)	Chemistry panel:	HIV ELISA
Complete physical exam	Urine dipstick/urinalysis	ALT	HIV Western blot (if applicable)
Concomitant medications	CBC with differential	AST	HIV RNA PCR
Intercurrent illness/AE	Platelet count	Alkaline phosphatase	
Reactogenicity	T-cell subsets	Creatinine	
Autoimmune symptoms assessment		CPK	

Immunogenicity determinations		
Endpoint assays (Humoral)	Endpoint assays (Cellular)	Ancillary assays
HIV binding antibody ELISA	IFN-γ ELISpot	MHC tetramer binding
	Intracellular cytokine staining (ICS)	Antibodies to IL-15
		Antibodies to IL-12 (Part B)
		T-cell receptor repertoire

Other post-enrollment procedures		
Vaccination administration	Risk reduction counseling	Outside testing/belief assessment
Specimen collection and shipping	Pregnancy prevention counseling	Cryopreservation/storage of specimens
HIV pre- and post-test counseling	HLA typing	Participant unblinding
Social impact assessment		

12.3 Total blood volumes

Required blood volumes are shown in Appendix C and D. Not shown is any additional blood volume that would be required if a safety lab needs to be repeated, or if a serum pregnancy test needs to be performed. The additional blood volume would likely be minimal. The total blood volume drawn for each participant will not exceed 500 mL in any 8-week period.

12.4 Autoimmune symptoms assessment

An autoimmune symptoms assessment will be performed at each visit.

12.5 Laboratory procedures

A *Laboratory Procedures* manual will be available that provides further guidelines for operational issues concerning the clinical laboratories and phlebotomy. The procedures include general specimen collection guidelines, special considerations for blood collection, HIV testing guidelines, guidelines for processing whole blood, and labeling guidelines.

In specific situations the blood collection tubes will be redirected to another laboratory for special screening criteria or safety issues. In these cases special shipping instructions will be provided in Special Instructions posted on the HVTN website.

13 Study product preparation and administration

HVTU pharmacists should consult the *Pharmacy Guidelines and Instructions for DAIDS Clinical Trials Networks* manual for standard pharmacy operations procedures. The protocol schema and vaccine regimen are shown in Section 13.1. See the Investigator's Brochure for further information about study products.

13.1 Schemas and vaccines regimens

Part A Schema

Study arm	Treatment	Vaccination schedule in months (days)		
		**0(0)**	**1(28)**	**3(84)**
Group 1	T1	HIV *gag* DNA 1500 mcg IM*	HIV *gag* DNA 1500 mcg IM*	HIV *gag* DNA 1500 mcg IM*
	C1	Placebo for HIV *gag* DNA 0.75 mL IM*	Placebo for HIV *gag* DNA 0.75 mL IM*	Placebo for HIV *gag* DNA 0.75 mL IM*
Group 2	T2	HIV *gag* DNA 1500 mcg admixed with *IL-15* DNA 100 mcg IM*	HIV *gag* DNA 1500 mcg admixed with *IL-15* DNA 100 mcg IM*	HIV *gag* DNA 1500 mcg admixed with *IL-15* DNA 100 mcg IM*
	C2	Placebo for HIV *gag* DNA / *IL-15* DNA 0.8 mL IM*	Placebo for HIV *gag* DNA / *IL-15* DNA 0.8 mL IM*	Placebo for HIV *gag* DNA / *IL-15* DNA 0.8 mL IM*
Group 3	T3	HIV *gag* DNA 1500 mcg admixed with *IL-15* DNA 500 mcg IM*	HIV *gag* DNA 1500 mcg admixed with *IL-15* DNA 500 mcg IM*	HIV *gag* DNA 1500 mcg admixed with *IL-15* DNA 500 mcg IM*
	C3	Placebo for HIV *gag* DNA / *IL-15* DNA 1 mL IM*	Placebo for HIV *gag* DNA / *IL-15* DNA 1 mL IM*	Placebo for HIV *gag* DNA / *IL-15* DNA 1 mL IM*
Group 4	T4	HIV *gag* DNA 1500 mcg admixed with *IL-15* DNA 1500 mcg IM*	HIV *gag* DNA 1500 mcg admixed with *IL-15* DNA 1500 mcg IM*	HIV *gag* DNA 1500 mcg admixed with *IL-15* DNA 1500 mcg IM*
	C4	Placebo for HIV *gag* DNA / *IL-15* DNA 1.5 mL IM*	Placebo for HIV *gag* DNA / *IL-15* DNA 1.5 mL IM*	Placebo for HIV *gag* DNA / *IL-15* DNA 1.5 mL IM*

*All vaccinations will be administered in the same deltoid at each vaccination visit (unless medically contraindicated for that deltoid). See Section 13.3.1.
See Section **8.2** regarding initial safety evaluation.
Enrollment in Groups 1 and 2 will occur simultaneously. See Section **8.2** regarding initial safety evaluation.
Enrollment in Groups 3 and 4 will be sequential. See Section **8.3** for dose escalation criteria.

Part B Schema

Study arm	Treatment	Vaccination schedule in months (days)			Booster schedule in months (days)	
		0(0)	1(28)	3(84)	6(168)	9(273)
Group 5	T5	HIV *gag* DNA 1500 mcg admixed with *IL-15* DNA 1500 mcg IM*	HIV *gag* DNA 1500 mcg admixed with *IL-15* DNA 1500 mcg IM*	HIV *gag* DNA 1500 mcg admixed with *IL-15* DNA 1500 mcg IM*	HIV *gag* DNA 1500 mcg admixed with *IL-15* DNA 1500 mcg IM*	HIV *gag* DNA 1500 mcg admixed with *IL-15* DNA 1500 mcg IM*
	C5	Placebo for HIV *gag* DNA / *IL-15* DNA 1.5 mL IM*	Placebo for HIV *gag* DNA / *IL-15* DNA 1.5 mL IM*	Placebo for HIV *gag* DNA / *IL-15* DNA 1.5 mL IM*	Placebo for HIV *gag* DNA / *IL-15* DNA 1.5 mL IM*	Placebo for HIV *gag* DNA / *IL-15* DNA 1.5 mL IM*
Group 7	T7	HIV *gag* DNA 1500 mcg admixed with *IL-15* DNA 1500 mcg IM*	HIV *gag* DNA 1500 mcg admixed with *IL-15* DNA 1500 mcg IM*	HIV *gag* DNA 1500 mcg admixed with *IL-15* DNA 1500 mcg IM*	HIV *gag* DNA 1500 mcg admixed with *IL-12* DNA 1500 mcg IM*	HIV gag DNA 1500 mcg admixed with IL-12 DNA 1500 mcg IM*
	C7	Placebo for HIV *gag* DNA / *IL-15* DNA 1.5 mL IM*	Placebo for HIV *gag* DNA / *IL-15* DNA 1.5 mL IM*	Placebo for HIV *gag* DNA / *IL-15* DNA 1.5 mL IM*	Placebo for HIV *gag* DNA / *IL-12* DNA 1.5 mL IM*	Placebo for HIV *gag* DNA / *IL-12* DNA 1.5 mL IM*

*All vaccinations will be administered in the same deltoid at each vaccination visit (unless medically contraindicated for that deltoid). See Section 13.3.1.

Enrollment in Groups 5 and 7 will occur simultaneously. See Section 8.2 regarding initial safety evaluation.

See Section 8.4 regarding safety evaluation for moving from Part A to Part B

Group 1

T1: HIV *gag* DNA 1500 mcg administered as 0.75 mL IM in same deltoid at months 0, 1, and 3 (unless medically contraindicated for that deltoid).

C1: Placebo for HIV *gag* DNA administered as 0.75 mL IM in same deltoid at months 0, 1, and 3 (unless medically contraindicated for that deltoid).

Group 2

T2: HIV *gag* DNA 1500 mcg admixed with *IL-15* DNA 100 mcg administered as 0.8 mL IM in same deltoid at months 0, 1, and 3 (unless medically contraindicated for that deltoid).

C2: Placebo for HIV *gag* DNA / *IL-15* DNA administered as 0.8 mL IM in same deltoid at months 0, 1, and 3 (unless medically contraindicated for that deltoid).

Group 3

T3: HIV *gag* DNA 1500 mcg admixed with *IL-15* DNA 500 mcg administered as 1 mL IM in same deltoid at months 0, 1, and 3 (unless medically contraindicated for that deltoid).

C3: Placebo for HIV *gag* DNA / *IL-15* DNA administered as 1 mL IM in same deltoid at months 0, 1, and 3 (unless medically contraindicated for that deltoid).

Group 4

T4: HIV *gag* DNA 1500 mcg admixed with *IL-15* DNA 1500 mcg administered as 1.5 mL IM in same deltoid at months 0, 1 and 3 (unless medically contraindicated for that deltoid)

C4: Placebo for HIV *gag* DNA / *IL-15* DNA administered as 1.5 mL IM in same deltoid at months 0, 1 and 3, (unless medically contraindicated for that deltoid).

Group 5

T5: HIV *gag* DNA 1500 mcg admixed with *IL-15* DNA 1500 mcg administered as 1.5 mL IM in same deltoid at months 0, 1, 3, 6 and 9 (unless medically contraindicated for that deltoid).

C5: Placebo for HIV *gag* DNA / *IL-15* DNA administered as 1.5 mL IM in same deltoid at months 0, 1, 3, 6 and 9 (unless medically contraindicated for that deltoid).

Group 7

T7: HIV *gag* DNA 1500 mcg admixed with *IL-15* DNA 1500 mcg administered as 1.5 mL IM in same deltoid at months 0, 1, and 3 (unless medically contraindicated for that deltoid)

and

HIV *gag* DNA 1500 mcg admixed with *IL-12* DNA 1500 mcg administered as 1.5 mL IM in same deltoid at months 6 and 9 (unless medically contraindicated for that deltoid)

C7: Placebo for HIV *gag* DNA / *IL-15* DNA administered as 1.5 mL IM in same deltoid at months 0, 1, and 3 (unless medically contraindicated for that deltoid)

and

Placebo for HIV *gag* DNA / *IL-12* DNA administered as 1.5 mL IM in same deltoid at months 6 and 9 (unless medically contraindicated for that deltoid).

13.2 Study products formulation and preparation

See the Investigator's Brochure for further information about study products.

13.2.1 HIV *gag* DNA (HIV-1 *gag* DNA Vaccine, GENEVAX® *gag-2962*)

HIV *gag* DNA is supplied as a sterile, clear, colorless, preservative-free aqueous formulation. Each 2 mL vial contains 1.2 mL +/– 0.1 mL of GENEVAX® *gag*-2962 at a concentration of 2 mg/mL in 30 mM citrate buffer pH 6.5 containing 150 mM NaCl, 0.01% ethylenediamine tetraacetic acid (EDTA) and 0.25% bupivacaine-HCl. The HIV *gag* DNA should be stored at 2° to 8°C until use. The product is contraindicated in participants with known hypersensitivity to bupivacaine.

13.2.2 *IL-15* DNA (*IL-15* adjuvant, GENEVAX® *IL-15*-1696)

GENEVAX® *IL-15*-1696 is a clear colorless liquid. It is supplied as a sterile, preservative-free aqueous formulations. Each 2 mL vial contains 0.9 mL +/– 0.1 mL of GENEVAX® *IL-15*-1696 at a concentration of 2 mg/mL in 30 mM citrate buffer pH 6.5 containing 150 mM NaCl, 0.01% EDTA and 0.25% bupivacaine-HCl. The product should be stored at 2° to 8°C until ready for use. The product is contraindicated in participants with known hypersensitivity to bupivacaine.

13.2.3 *IL-12* DNA (*IL-12* adjuvant, GENEVAX® *IL-12*-4532)

GENEVAX® *IL-12*-4532 is a clear colorless liquid. The product is supplied as a sterile, preservative-free aqueous formulation. Each 2 mL vial contains 0.9 mL +/– 0.1 mL of GENEVAX® *IL-12*-4532 at a concentration of 2 mg/mL in 30 mM citrate buffer pH 6.5 containing 150 mM NaCl, 0.01% EDTA and 0.25% bupivacaine-HCl. The product should be stored at 2° to 8°C until ready for use. The product is contraindicated in participants with known hypersensitivity to bupivacaine.

13.2.4 Placebo (Sodium Chloride Injection USP, 0.9%, NaCl 0.9%)

Sodium chloride injection USP, 0.9% will be used as the placebo for all groups. The volume to be administered will vary from group to group to maintain the blind between active and placebo treatment (T and C) in each group. The vials must be stored as directed by the manufacturer of the product unless otherwise instructed.

13.2.5 Preparation of HIV *gag* DNA 1500 mcg or Placebo (Group 1)

HIV gag *DNA 1500 mcg*

One vial containing *HIV* gag *DNA 1500 mcg* will be needed to prepare this dose. The vial should be visually inspected prior to use. If the vial contains material different from its description above, do not use the vial and contact the protocol pharmacist. Using aseptic technique (in a laminar flow hood), the pharmacist will withdraw 0.75 mL of HIV *gag* DNA from the vial into a 3 mL syringe. The pharmacist will label the syringe as HIV gag DNA Plasmid 1500 mcg or placebo. The final product must be administered within 4 hours after preparation. It must be stored at 2° to 8°C if not administered immediately after preparation.

Any unused portion of entered vials and expired pre-filled syringes should be disposed of in accordance with institutional or pharmacy policy.

Placebo (for HIV gag *DNA 1500 mcg)*

One vial containing Sodium Chloride Injection USP, 0.9% will be needed to prepare this dose. The vial should be visually inspected prior to use. Using aseptic technique, the pharmacist will withdraw 0.75 mL of Sodium Chloride Injection USP, 0.9% from the vial into a 3 mL syringe. The pharmacist will label the syringe as HIV gag DNA Plasmid 1500 mcg or placebo. The final product must be administered within 4 hours after preparation. It must be stored at 2° to 8°C if not administered immediately after preparation.

Any unused portion of entered vials and expired pre-filled syringes should be disposed of in accordance with institutional or pharmacy policy.

13.2.6 Preparation of HIV *gag* DNA 1500 mcg / *IL-15* DNA 100 mcg or Placebo (Group 2)

HIV gag *DNA 1500 mcg /* IL-15 *DNA 100 mcg*

All vials should be visually inspected prior to use. If any vial contains material different from its description above, do not use the vial and contact the protocol pharmacist. Using aseptic technique, the pharmacist will use a 0.3 mL insulin syringe/needle to withdraw 0.08 mL (8 unit marking) from the vial containing *IL-15* DNA 2mg/mL. The contents of this syringe will then be slowly injected into the vial containing 1.2 mL of HIV *gag* DNA (2 mg/mL). The pharmacist should avoid creating bubbles. To prevent aerosolization, the pharmacist should, before removing the needle from the vial, bring the needle above the level of the liquid and allow excess air to rise into the syringe. Remove the syringe from the vial and discard into a sharp's container. The pharmacist will mix the solution in the vial by gentle swirling (avoid

creating bubbles). The final product should be a clear colorless liquid containing 1.28 mL of a 15:1 mixture of HIV *gag* DNA*: IL-15* DNA, consisting of 1875 mcg/mL of HIV *gag* DNA and 125 mcg/mL of *IL-15* DNA. Using a new 3 mL syringe, the pharmacist will withdraw 0.8 mL of this final product. The pharmacist will label the syringe as HIV *gag* DNA 1500 mcg / *IL-15* DNA 100 mcg or placebo. The final product must be administered within 4 hours after preparation. It must be stored at 2° to 8°C if not administered immediately after preparation.

Any unused portion of entered vials and expired pre-filled syringes should be disposed of in accordance with institutional or pharmacy policy.

Placebo (for HIV gag *DNA 1500 mcg /* IL-15 *DNA 100 mcg)*

Using aseptic technique, the pharmacist will withdraw 0.8 mL of sodium chloride injection USP, 0.9% into a 3 mL syringe. The pharmacist will label the syringe as HIV *gag* DNA 1500 mcg / *IL-15* DNA 100 mcg or placebo. The final product must be administered within 4 hours after preparation. It must be stored at 2 to 8°C if not administered immediately after preparation.

Any unused portion of entered vials and expired pre-filled syringes should be disposed of in accordance with institutional or pharmacy policy.

13.2.7 Preparation of HIV *gag* DNA 1500 mcg / *IL-15* DNA 500 mcg or Placebo (Group 3)

HIV gag *DNA 1500 mcg /* IL-15 *DNA 500 mcg*

All vials should be visually inspected prior to use. If any vial contains material different from its description above, do not use the vial and contact the protocol pharmacist. Using aseptic technique (in a laminar flow hood), the pharmacist will use a 1 mL syringe to withdraw 0.4 mL from the vial containing *IL-15* DNA 2mg/mL. The contents of this syringe will then be slowly injected into the vial containing 1.2 mL of HIV *gag* DNA (2 mg/mL). The pharmacist should avoid creating bubbles. To prevent aerosolization, the pharmacist should, before removing the needle from the vial, bring the needle above the level of the liquid and allow excess air to rise into the syringe. Remove the syringe from the vial and discard into a sharp's container. The pharmacist will mix the solution in the vial by gentle swirling (avoid creating bubbles). The final product should be a clear colorless liquid containing 1.6 mL of a 3:1 mixture of HIV *gag* DNA*: IL-15* DNA, consisting of 1500 mcg/mL of HIV *gag* DNA and 500 mcg/mL of *IL-15* DNA. Using a new 3 mL syringe, the pharmacist will withdraw 1 mL of this final product. The pharmacist will label the syringe as HIV *gag* DNA 1500 mcg / *IL-15* DNA 500 mcg or placebo. The final product must be administered within 4 hours after preparation. It must be stored at 2 to 8°C if not administered immediately after preparation.

Any unused portion of entered vials and expired pre-filled syringes should be disposed of in accordance with institutional or pharmacy policy.

Placebo (for HIV gag *DNA 1500 mcg /* IL-15 *DNA 500 mcg)*

Using aseptic technique, the pharmacist will withdraw 1 mL of sodium chloride injection USP, 0.9% into a 3 mL syringe. The pharmacist will label the syringe as HIV *gag* DNA 1500 mcg / *IL-15* DNA 500 mcg or placebo. The final product must be administered within 4 hours after preparation. It must be stored at 2 to 8°C if not administered immediately after preparation.

Any unused portion of entered vials and expired pre-filled syringes should be disposed of in accordance with institutional or pharmacy policy.

13.2.8 Preparation of HIV *gag* DNA 1500 mcg / *IL-15* DNA 1500 mcg or Placebo (Groups 4- 7)

HIV gag *DNA 1500 mcg /* IL-15 *DNA 1500 mcg*

All vials should be visually inspected prior to use. If any vial contains material different from its description above, do not use the vial and contact the protocol pharmacist. Using aseptic technique, the pharmacist will use a 3 mL syringe to withdraw 0.9 mL from the vial containing HIV *gag* DNA 2 mg/mL. The contents of this syringe will then be slowly injected into the vial containing 0.9 mL of *IL-15* DNA (2 mg/mL). The pharmacist should avoid creating bubbles. To prevent aerosolization, the pharmacist should, before removing the needle from the vial, bring the needle above the level of the liquid and allow excess air to rise into the syringe. Remove the syringe from the vial and discard into a sharp's container. The pharmacist will mix the solution in the vial by gentle swirling (avoid creating bubbles). The final product should be a clear colorless liquid containing 1.8 mL of a 1:1 mixture of HIV *gag* DNA*: IL-15* DNA, consisting of 1000 mcg/mL of HIV *gag* DNA and 1000 mcg/mL of *IL-15* DNA. Using a new 3 mL syringe, the pharmacist will withdraw 1.5 mL of this final product. The pharmacist will label the syringe as HIV *gag* DNA 1500 mcg / *IL-15* DNA 1500 mcg or placebo. The final product must be administered within 4 hours after preparation. It must be stored at 2 to 8°C if not administered immediately after preparation.

Any unused portion of entered vials and expired pre-filled syringes should be disposed of in accordance with institutional or pharmacy policy.

Placebo (for HIV gag *DNA 1500 mcg /* IL-15 *DNA 1500 mcg)*

Using aseptic technique, the pharmacist will withdraw 1.5 mL of sodium chloride injection USP, 0.9% into a 3 mL syringe. The pharmacist will label the syringe as HIV *gag* DNA 1500 mcg / *IL-15* DNA 1500 mcg or placebo. The final product must be administered within 4 hours after preparation. It must be stored at 2 to 8°C if not administered immediately after preparation.

Any unused portion of entered vials and expired pre-filled syringes should be disposed of in accordance with institutional or pharmacy policy.

13.2.9 Preparation of HIV *gag* DNA 1500 mcg / *IL-12* DNA 1500 mcg or Placebo (Group 7)

HIV gag *DNA 1500 mcg /* IL-12 *DNA 1500 mcg*

All vials should be visually inspected prior to use. If any vial contains material different from its description above, do not use the vial and contact the protocol pharmacist. Using aseptic technique, the pharmacist will use a 3 mL syringe to withdraw 0.9 mL from the vial containing HIV *gag* DNA 2 mg/mL. The contents of this syringe will then be slowly injected into the vial containing 0.9 mL of *IL-12* DNA (2 mg/mL) (Lot # 76180001A). The pharmacist should avoid creating bubbles. To prevent aerosolization, the pharmacist should, before removing the needle from the vial, bring the needle above the level of the liquid and allow excess air to rise into the syringe. Remove the syringe from the vial and discard into a sharp's container. The pharmacist will mix the solution in the vial by gentle swirling (avoid creating bubbles). The final product should be a clear colorless liquid containing 1.8 mL of a 1:1 mixture of HIV *gag* DNA*: IL-12* DNA, consisting of 1000 mcg/mL of HIV *gag* DNA and 1000 mcg/mL of *IL-12* DNA. Using a new 3 mL syringe, the pharmacist will withdraw 1.5 mL of this final product. The pharmacist will label the syringe as HIV *gag* DNA 1500 mcg / *IL-12* DNA 1500 mcg or placebo. The final product must be administered within 4 hours after preparation. It must be stored at 2 to 8°C if not administered immediately after preparation.

Any unused portion of entered vials and expired pre-filled syringes should be disposed of in accordance with institutional or pharmacy policy.

Placebo (for HIV gag *DNA 1500 mcg / IL-12 DNA 1500 mcg)*

Using aseptic technique, the pharmacist will withdraw 1.5 mL of sodium chloride injection USP, 0.9% into a 3 mL syringe. The pharmacist will label the syringe as HIV *gag* DNA 1500 mcg / *IL-12* DNA 1500 mcg or placebo. The final product must be administered within 4 hours after preparation. It must be stored at 2 to 8°C if not administered immediately after preparation.

Any unused portion of entered vials and expired pre-filled syringes should be disposed of in accordance with institutional or pharmacy policy.

13.2.10 Pharmacy procedures to preserve blinding

The pharmacist will prepare the doses for administration and dispense to the clinic.

13.3 Study products administration

All vaccinations are to be given intramuscularly into the deltoid muscle. The needle length should be appropriate for the participant's weight.

When preparing a dose in a syringe and administering the dose, consideration should be given to the volume of solution that may remain in the needle after the dose is administered. The pharmacy and clinic staffs are encouraged to work together to administer the dose specified in the protocol.

The person who administers the dose must not be the same individual who is responsible for clinical follow-up of that dose. At sites where registered pharmacists are legally authorized to administer drug, the HVTU may choose to have the HVTU pharmacist administer the vaccinations.

13.3.1 Administration of vaccinations

Syringes should be kept refrigerated at 2° to 8°C (36° to 46°F) until just prior to injection unless the administration is immediately after preparation. Administer the vaccine into the deltoid muscle after preparation of the site with alcohol. The same deltoid should be used at each vaccination visit unless medically contraindicated. If the injection is administered in the opposite deltoid due to a medical contraindication, the appropriate study staff should document this clearly and submit a Study Product Administration Error form. Under this circumstance, this is NOT a protocol violation.

As significant adverse experiences—including cardiac arrest and death—have occurred with intravascular delivery of bupivacaine, it is essential that aspiration for blood be performed prior to injection of all gag DNA, IL-15 DNA, IL-12 DNA, or their placebo injections to ensure that intravenous (i.v.) delivery does not occur.

13.4 Study products acquisition

All active study products will be provided by Wyeth Vaccines Research. The placebo (sodium chloride injection USP, 0.9%) will not be provided through the protocol but must be purchased by the site.

At US-HVTUs the pharmacist can obtain study products from the NIAID Clinical Research Products Management Center (CRPMC) by following the ordering procedures given in the

section on Study Product Control in *Pharmacy Guidelines and Instructions for DAIDS Clinical Trials Networks*.

At non-US HVTUs the pharmacist can obtain study products from the NIAID Clinical Research Products Management Center (CRPMC). Once a non-US HVTU is registered for the study and all required documents have been received by the CRPMC, the Pharmacist can order product by following the procedures given in the *HVTN063 Study Specific Procedures (SSP)*.

13.5 Pharmacy records

The HVTU pharmacist is required to maintain complete records of all study products received from the CRPMC and subsequently dispensed. All unused study products must be returned to the CRPMC after the study is completed or terminated unless otherwise instructed. The procedures are included in the sections on Study Product Placebo and Drug Dispensing in *Pharmacy Guidelines and Instructions for DAIDS Clinical Trials Networks*.

The pharmacist of record is responsible for maintaining randomization codes and randomization confirmation notices for each participant in a secure manner.

14 Safety monitoring and review

14.1 Assessing reactogenicity

Reactogenicity assessments are performed for all participants following each vaccination. HVTU staff will assess reactogenicity according to standard HVTN procedures as specified in the HVTN MOP (Study Operations>Safety Assessment>Reactogenicity). Any procedures which vary from the HVTN standard will be defined in *HVTN 063 Study Specific Procedures*.

The reactogenicity assessment period is for 3 days following the vaccination. Participants are instructed to record symptoms using a post-vaccination symptom log and contact the site daily during this reactogenicity assessment period. Clinic staff will follow new or unresolved reactogenicity symptoms present at Day 3 to resolution. The schedule is shown in Table 14-1.

Assessments to be performed:

- Systemic symptoms: body temperature, malaise and/or fatigue, myalgia, headache, chills, arthralgia, nausea, vomiting
- Local symptoms (proximal to injection site): pain, tenderness
- Vaccine-related lesions: erythema, induration
- Axillary lymph nodes (required only when reactogenicity assessments are performed by HVTU staff): lymph node tenderness, enlargement

Table 14-1 Schedule of reactogenicity assessments

Day	Time	Performed by
0[a]	Baseline: before vaccination	HVTU staff
	Early: 25 to 45 minutes after vaccination	HVTU staff
	Between early assessment and 11:59pm Day 0	HVTU staff or participant
1	Between 12:00am and 11:59pm Day 1	HVTU staff or participant
2	Between 12:00am and 11:59pm Day 2	HVTU staff or participant
3	Between 12:00am and 11:59pm Day 3	HVTU staff or participant

[a]Day of vaccination

14.2 Grading adverse experiences

Local and systemic signs and symptoms are assessed and graded based on *the Division of AIDS Table for Grading the Severity of Adult and Pediatric Adverse Events* (DAIDS AE Grading Table), Version 1.0, December, 2004 which is available on the RCC website at http://rcc.tech-res-intl.com/.

14.3 Adverse event reporting and safety pause /AE review rules

All adverse events are reported to the SDMC on the appropriate case report form (CRF) according to procedures in the HVTN MOP (Study Operations>Safety Assessments>Adverse Experiences). The mechanism of reporting certain Grade 2 and higher grade vaccine-related symptoms and adverse events to the SDMC Clinical Affairs staff is depicted in Table 14-2 (for Part A) and Table 14-3 (for Part B). The mechanism of reporting SAEs and other events meeting expedited adverse event (EAE) reporting requirements to DAIDS is specified in Section 14.4.

14.3.1 Adverse events to which safety pause /AE review rules apply

The adverse events applying toward a safety pause or Protocol Safety Review Team (PSRT) AE review are shown in Table 14-2 (for Part A) and Table 14-3 (for Part B). In order to be counted toward a safety pause or PSRT AE review, adverse events must be vaccine related. ‘Vaccine related’ means the event is judged to be possibly related, probably related, or definitely related to the study vaccination. Symptoms reported on a reactogenicity CRF are assumed by the SDMC to be vaccine related.

14.3.2 Adverse event reporting

Notify the SDMC Clinical Affairs staff of adverse events as indicated in Table 14-2 and Table 14-3. Telephone numbers and email addresses are listed in the *HVTN 063 Study Specific Procedures,* Key Resource Guide, and can be used to notify SDMC Clinical Affairs staff of any serious safety concern requiring their attention. Concerns requiring immediate attention should be communicated by telephone.

In the case of email notification, SDMC Clinical Affairs staff will reply during working hours (US Pacific Time) to confirm that the email has been received and reviewed. If email service is not available, the HVTU will notify SDMC Clinical Affairs of the event by telephone, then submit case report forms.

Case report forms should be faxed within the timeframes indicated in Table 14-2 and Table 14-3. If a DAIDS EAE Reporting Form is indicated for the event, a copy of the form is faxed to SDMC (see Section 14.4).

14.3.3 PSRT AE review and safety pause

If a PSRT AE review is triggered, the SDMC Clinical Affairs staff notifies the HVTN 063 PSRT as soon as possible during working hours (US Pacific Time)—or, if the information was received during off hours, by the morning of the next work day—that a PSRT AE review is needed. If a PSRT AE review cannot be completed within 24 hours of SDMC notification (excluding weekends and US federal holidays), an automatic safety pause occurs.

For all safety pauses, the SDMC Clinical Affairs staff notifies the PSRT, DAIDS Pharmaceutical Affairs Branch (PAB), Regulatory Compliance Center (RCC)/Regulatory Affairs Branch (RAB), and participating HVTUs that all study vaccinations are held until further notice. When an immediate safety pause is triggered, the SDMC Clinical Affairs staff also notifies the HVTN SMB; DAIDS notifies the US FDA.

Vaccinations may be suspended for safety concerns other than those described in Table 14-2 and Table 14-3, or before pause rules are met, if in the judgment of the PSRT, participant safety may be threatened.

Adverse events that do not prompt a safety pause or a PSRT AE review are routinely reviewed by the PSRT (Section 14.8.2).

Table 14-2 Adverse event notification and safety pause/AE review rules for Part A

Rule	Toxicity	Vaccine-related symptom/AE	HVTU action	SDMC action	Criterion for SDMC action	Criterion for each subsequent SDMC action
1	Grade 4	Any lab abnormality, adverse event, local or systemic reactogenicity symptom	Phone/page immediately, email and fax forms immediately	Immediate pause	≥1 ppt with AE/symptom at specified grade	≥1 additional ppt with the same AE/symptom at specified grade
2	Grade 3	Any lab abnormality, adverse event, or localized injection site reaction	Email immediately and fax forms immediately	Immediate pause	≥1 ppt with AE/symptom at specified grade	≥1 additional ppt with the same AE/symptom at specified grade
3	Grade 3	Fever or vomiting	Fax forms within 24 hours	Prompt PSRT AE review	≥2 ppts with the same AE/symptom at specified grade	≥2 additional ppts with the same AE/symptom at specified grade
4	Grade 3	Subjective reactogenicity symptom (injection site pain, tenderness, fatigue/malaise, myalgia, arthralgia, chills, headache, nausea)	Fax forms within 24 hours	Prompt PSRT AE review	≥3 ppts with the same AE/symptom at specified grade	≥2 additional ppts with the same AE/symptom at specified grade
5	Grade 2	Any lab abnormality, erythema, induration/swelling/edema, or adverse event (other than fever, vomiting, or a subjective reactogenicity symptom)	Fax forms within 24 hours	Prompt PSRT AE review	≥2 ppts with the same AE/symptom at specified grade	≥2 additional ppts with the same AE/symptom at specified grade
6	Grade 2	Fever or vomiting	Fax forms within 24 hours	Prompt PSRT AE review	≥5 ppts with the same AE/symptom at specified grade	≥2 additional ppts with the same AE/symptom at specified grade

For AE descriptions and grading, see *The Division of AIDS Table for Grading the Severity of Adult and Pediatric Adverse Events* (DAIDS AE Grading Table), Version 1.0, December, 2004.

Phone numbers and email addresses are listed in *HVTN 063 Study Specific Procedures*, Key Resource Guide.

Table 14-3 Adverse event notification and safety pause/AE review rules for Part B

Rule	Toxicity	Vaccine-related symptom/AE	HVTU action	SDMC action	Criterion for SDMC action	Criterion for each subsequent SDMC action
1	Grade 4	Any lab abnormality, adverse event, local or systemic reactogenicity symptom	Phone/page immediately, email and fax forms immediately	Immediate pause	≥1 ppt with AE/symptom at specified grade	≥1 additional ppt with the same AE/symptom at specified grade
2	Grade 3	Any lab abnormality, or adverse event	Email immediately and fax forms immediately	Immediate pause	≥1 ppt with AE/symptom at specified grade	≥1 additional ppt with the same AE/symptom at specified grade
3	Grade 3	Localized injection site reaction	Email immediately and fax forms immediately	Immediate pause	≥2 ppts with the same AE/symptom at specified grade	≥2 additional ppts with the same AE/symptom at specified grade
4	Grade 3	Fever or vomiting	Fax forms within 24 hours	Prompt PSRT AE review	≥2 ppts *and* ≥10% ppts with the same AE/symptom at specified grade	≥2 additional ppts, *and* ≥10% ppts overall with the same AE/symptom at specified grade
5	Grade 3	Subjective reactogenicity symptom (injection site pain, tenderness, fatigue/malaise, myalgia, arthralgia, chills, headache, nausea)	Fax forms within 24 hours	Prompt PSRT AE review	≥3 ppts *and* ≥5% ppts with the same AE/symptom at specified grade	≥2 additional ppts, *and* ≥10% ppts overall with the same AE/symptom at specified grade
6	Grade 2	Any lab abnormality, erythema, induration/swelling/ edema, or adverse event (other than fever, vomiting, or a subjective reactogenicity symptom)	Fax forms within 24 hours	Prompt PSRT AE review	≥2 ppts *and* ≥10% ppts with the same AE/symptom at specified grade	≥2 additional ppts, *and* ≥10% ppts overall with the same AE/symptom at specified grade
7	Grade 2	Fever or vomiting	Fax forms within 24 hours	Prompt PSRT AE review	≥5 ppts *and* ≥10% ppts with the same AE/symptom at specified grade	≥2 additional ppts, *and* ≥10% ppts overall with the same AE/symptom at specified grade

For AE descriptions and grading, see The Division of AIDS Table for Grading the Severity of Adult and Pediatric Adverse Events (DAIDS AE Grading Table), Version 1.0, December, 2004.

Phone numbers and email addresses are listed in *HVTN 063 Study Specific Procedures*, Key Resource Guide.

14.3.4 Review and notification following safety pause

The PSRT reviews safety data and decides whether the pause can be lifted or permanent discontinuation of vaccination is appropriate, consulting the SMB and the US FDA if necessary. DAIDS will consult with the US FDA for all immediate safety pauses. SDMC Clinical Affairs staff notifies participating HVTUs, PAB, and RCC/RAB of the decision regarding resumption or discontinuation of study vaccinations. SDMC Clinical Affairs staff also notifies the HVTN SMB, and DAIDS notifies the FDA, if these groups have not been informed earlier.

Each HVTU is responsible for submitting to its IRB/IEC and any local regulatory authority protocol-related safety information (such as IND safety reports, notification of vaccine holds due to the pause rules, etc.) as required.

14.4 Expedited Adverse Event Reporting to DAIDS

The expedited adverse event (EAE) reporting requirements and definitions for this study and the methods for expedited reporting of adverse events (AEs) to the DAIDS Regulatory Compliance Center (RCC) Safety Office are defined in "The Manual for Expedited Reporting of Adverse Events to DAIDS" (DAIDS EAE Manual), dated May 6, 2004. The DAIDS EAE Manual is available on the RCC website: http://rcc.tech-res-intl.com/.

AEs reported on an expedited basis must be documented on the DAIDS Expedited Adverse Event Reporting Form (EAE Reporting Form) available on the RCC website: http://rcc.tech-res-intl.com.

14.4.1 EAE Reporting Level

This study uses the Standard Level of expedited AE reporting as defined in the DAIDS EAE Manual.

14.4.2 Study Agents for Expedited Reporting to DAIDS

The study agents that must be considered in determining relationships of AEs requiring expedited reporting to DAIDS are:

- GENEVAX® gag-2962/placebo
- GENEVAX® IL-12-4532/placebo
- GENEVAX® IL-15-1696/placebo

14.4.3 EAE Reporting Periods

AEs must be reported on an expedited basis at the Standard Level during the Protocol-defined EAE Reporting Period, which is the entire study duration for an individual subject (from study enrollment until study completion or discontinuation of the subject from study participation for any reason).

After the end of the Protocol-defined EAE Reporting Period stated above, sites must report serious, unexpected, clinical suspected adverse drug reactions if the study site staff becomes aware of the event on a passive basis, i.e., from publicly available information.

14.5 Compliance with the NIH Guidelines for Research Involving Recombinant DNA Molecules

Human gene transfer trials conducted at or sponsored by institutions receiving NIH funds must be submitted to the NIH Office of Biotechnology Activities (OBA) for review by the Recombinant DNA Advisory Committee (RAC) and to Institutional Biosafety Committees (IBCs) in accordance with the NIH *Guidelines for Research Involving Recombinant DNA Molecules* (NIH Guidelines). A major role for the RAC and the IBC is to examine clinical trials that involve the transfer of recombinant DNA to humans.

The NIH Guidelines create exceptions to RAC review, but the HVTN 063 protocol team determined that the exceptions did not apply. Therefore, the protocol team, jointly with Wyeth, submitted the application with the study concept proposal for RAC review and

responded to RAC comments. The application followed the guidance provided in the NIH Guidelines. After initial review, RAC determined that the submission did not require an in-depth review and public RAC discussion. RAC notified the HVTN of this decision on August 16, 2004.

Without documented exemptions, the NIH Guidelines state that "[n]o research participant shall be enrolled in a human gene transfer experiment until the RAC review process has been completed (see Appendix M-I-B, RAC Review Requirements); IBC approval (from the clinical trial site) has been obtained; Institutional Review Board (IRB) approval has been obtained; and all applicable regulatory authorization(s) have been obtained." *Section IV-B-7-b-(6)*

Investigators at each site are responsible for obtaining Institutional Biosafety Committee (IBC) approval and periodic review of the research per NIH Guidelines *Section IV-B-7-b-(6)* and *Section IV-B-2-b*. IBC review and approval must be documented by the investigator and submitted as part of protocol registration for this trial.

The HVTN and DAIDS will ensure that reporting requirements to RAC, as outlined in *Appendix M-I-C-1.Initiation of the Clinical Investigation*, *Appendix M-I-C-3.Annual Reports*, and *Appendix M-I-C-4.Safety Reporting* are satisfied per the NIH Guidelines.

14.6 Participant departure from schedule of vaccinations

14.6.1 Delaying vaccinations for a participant

Under certain circumstances a participant's scheduled vaccination may need to be held. These include but are not limited to the following:

- Receipt of live attenuated vaccines within 30 days prior to vaccination
- Receipt of medically indicated subunit or killed vaccines (e.g., influenza, pneumococcal) within 14 days prior to vaccination
- Use of other investigational research agents within 30 days prior to vaccination
- Receipt of blood products or immunoglobulin within 45 days prior to vaccination
- Prevaccination abnormal vital signs or clinical symptoms that may mask assessment of vaccine reaction

Vaccinations cannot be administered outside the window period specified in the *HVTN 063 Study Specific Procedures*.

14.6.2 Stopping vaccinations for a participant

Under certain circumstances, an individual participant's vaccinations will be stopped. Such participants should be encouraged to participate in follow-up visits and all protocol-related procedures (unless medically contraindicated) per the protocol for the remainder of the trial. Specific events that will result in stopping a participant's vaccination schedule include the following:

- Clinically significant condition (i.e., a condition that affects the immune system or for which continued vaccinations and/or blood draws may pose additional risk), including but not limited to the following:
  - HIV infection (requires termination from the study)
  - Pregnancy (regardless of outcome)

    - Any Grade 4 local or systemic symptom, lab abnormality, or adverse experience, that is subsequently confirmed to be *possibly*, *probably*, or *definitely* related to vaccination
    - Any Grade 3 lab abnormality or other clinical adverse experience (exception: fever or vomiting and subjective local and systemic symptoms) that is subsequently confirmed to be *possibly*, *probably*, or *definitely* related to vaccination
    - Type 1 hypersensitivity associated with vaccination
- Inability to receive vaccination within the specified period for the designated study visit (see *HVTN 063 Study Specific Procedures*)
- Investigator determination in consultation with the study chair and statistician, e.g., for repeated nonadherence to protocol requirements

14.6.3 Participant termination from the study

Under certain circumstances, an individual participant may be terminated from participation in this study. Specific events that will result in early termination include:

- Participant refused to participate further
- Participant relocated to an area without a nearby HVTU and remote follow-up is not possible
- HVTU determined that the participant is lost to follow-up
- Participant becomes HIV-infected
- Investigator determination in consultation with the study chair and statistician, e.g., for repeated nonadherence to protocol requirements

14.7 Study termination (for all participants)

This study may be terminated by the determination of the HVTN 063 Protocol Safety Review Team, HVTN Safety Monitoring Board, US FDA, US NIH, vaccine developer or regulatory authority (e.g., IRB or IEC), as well as local regulatory authority for non-US sites. See Section 14.8 for discussion of the safety review process.

14.8 HVTN review of cumulative safety data

Routine safety review occurs at the start of enrollment, and then daily, weekly, quarterly and every 4 months during the study.

Reviews proceed from a standardized set of protocol-specific safety data reports produced by SDMC:

- Clinical quality control
- Safety review
- Pre-existing conditions
- Adverse events (AEs) requiring review
- Adverse event/concomitant medication
- WBC/differential

- Safety summary

More detailed information regarding the contents and distribution of these reports can be found in the HVTN MOP.

14.8.1 Daily review

Blinded daily safety reviews are routinely conducted by the SDMC Clinical Affairs staff for SAEs and events that meet safety pause criteria (Table 14-2, Table 14-3).

14.8.2 Weekly review

Blinded weekly safety reviews are routinely performed by the SDMC Clinical Affairs staff and by the HVTN 063 Protocol Safety Review Team. After the vaccinations and the final 2-week safety visits are completed, less frequent safety reviews may be scheduled at the discretion of the Protocol Safety Review Team. The SDMC Clinical Affairs staff reviews reports of all clinical values that fall outside of the standard HVTN safety parameters (see HVTN MOP [Study Operations>Standard Reports>Clinical Safety Review>Weekly Safety Review Reports]). Values identified during the review that are considered questionable, inconsistent, or unexplained are referred to the HVTU clinic coordinator for verification.

The Protocol Safety Review Team is composed of the following required members:

- Protocol chair and co-chair
- HVTN clinical trials physician
- SDMC Clinical Affairs staff member
- DAIDS medical officer

The protocol team clinic coordinator, protocol specialist, and vaccine developer representative may also be included at the request of the Protocol Safety Review Team.

14.8.3 Quarterly review

In addition to the detailed clinical monitoring reports discussed above, protocol-specific summary reports of reactogenicity and AE data are provided to the HVTN 063 protocol team and the HVTN Phase I/II Committee in a blinded fashion approximately once per quarter.

14.8.4 Safety Monitoring Board review

The HVTN safety monitoring board is composed of the following individuals:

- SMB Chair
- DAIDS Medical Officer representative
- Non-US representative
- US representative
- Statistician
- Clinician
- HVTN director

Members of the HVTN Safety Monitoring Board are not directly affiliated with the protocols under review. The safety monitoring board will review unblinded safety data approximately every 4 months. This review is designed to provide confirmation with respect to ad hoc

review requests as well as increase overall sensitivity for detecting potential safety problems by looking across multiple protocols that use the same or similar vaccine candidates. The review consists of evaluation of unblinded safety data, including comparisons of adverse experiences in vaccine and placebo recipients in aggregate, as well as review of individual SAE reports. The Safety Monitoring Board will conduct additional special reviews at the request of the HVTN 063 Protocol Safety Review Team.

14.8.5 Review for dose escalation

The Protocol Safety Review Team will examine the safety and reactogenicity events for all participants in each group to date and will discuss every event that triggers the pause rules and determine the advisability of continuing the dose escalation. At a minimum, if 2 or more participants enrolled in a given group report the same Grade 4, vaccine-related reactogenicity or adverse experience, then further vaccinations will be permanently suspended (in that group and any other group at a higher dose level). As noted in Section 14.3.1, any such Grade 4 event will require protocol safety review team consultation with the US FDA. Additionally, vaccinations may be suspended for any safety concern if, in the judgment of the protocol safety review team, participant safety is threatened.

14.8.6 Review for advancing from Part A to Part B

See Section 8.4.

References

1. UNAIDS, WHO. AIDS epidemic update. **2004**;UNAIDS/04.45E.

2. Centers for Disease Control and Prevention. HIV/AIDS surveillance report. **2003**.

3. UNAIDS. Report on the global HIV/AIDS epidemic. **2004**;UNAIDS/04.16E.

4. World Health Organization. AIDS surveillance in the Americas. **1998**.

5. Bloch C. Epidemiological HIV/AIDS situation in Argentina. Actualizaciones en SIDA **2001**;**9 Suppl**:15-24.

6. Sanchez JL, Whittington WLH, Zuckerman RA, Ashley RL, Lama JR, Galvan R. Bacterial STDs and HSV-2 acquisition among MSM HIV seroconverters in Peru. Conf HIV Pathog Treatm, Buenos Aires, Argentina, **7-8-2001**.

7. Lama JR, Goicochea LP, Chion M, et al. [Comportamiento sexual y factores de riesgo para ETS e infeccion VIH entre hombres que tienen sexo con otros hombres de Lima, Peru]. Pan-American Conference on AIDS, Lima, Peru, **12-3-1997**.

8. Tabet S, Sanchez J, Lama J, et al. HIV, syphilis and heterosexual bridging among Peruvian men who have sex with men. AIDS **2002**;16:1271-7.

9. Schechter M, Strathdee SA, Martindale SL, et al. Evidence of elevated HIV incidence and relapse to unsafe sex among young men having sex with men (MSM) in Vancover Canada. Int Conf AIDS, **6-28-1998**.

10. Fauci AS. The AIDS epidemic--considerations for the 21st century. N Engl J Med **1999**;341:1046-50.

11. Varmus H, Nathanson N. Science and the control of AIDS. Science **1998**;280:1815.

12. Yang OO, Kalams SA, Rosenzweig M, et al. Efficient lysis of human immunodeficiency virus type 1-infected cells by cytotoxic T lymphocytes. J Virol **1996**;70:5799-806.

13. Yang OO, Kalams SA, Trocha A, et al. Suppression of human immunodeficiency virus type 1 replication by CD8+ cells: evidence for HLA class I-restricted triggering of cytolytic and noncytolytic mechanisms. J Virol **1997**;71:3120-8.

14. Wagner L, Yang OO, Garcia-Zepeda EA, et al. Beta-chemokines are released from HIV-1-specific cytolytic T-cell granules complexed to proteoglycans. Nature **1998**;391:908-11.

15. Moodycliffe AM, Shreedhar V, Ullrich SE, et al. CD40-CD40 ligand interactions in vivo regulate migration of antigen-bearing dendritic cells from the skin to draining lymph nodes. J Exp Med **2000**;191:2011-20.

16. Ahlers JD, Belyakov IM, Thomas EK, Berzofsky JA. High-affinity T helper epitope induces complementary helper and APC polarization, increased CTL, and protection against viral infection. J Clin Invest **2001**;108:1677-85.

17. Geginat J, Sallusto F, Lanzavecchia A. Cytokine-driven proliferation and differentiation of human naive, central memory, and effector memory CD4(+) T cells. J Exp Med **2001**;194:1711-9.

18. Wolff JA, Malone RW, Williams P, et al. Direct gene transfer into mouse muscle in vivo. Science **1990**;247:1465-8.

19. Tang DC, DeVit M, Johnston SA. Genetic immunization is a simple method for eliciting an immune response. Nature **1992**;356:152-4.

20. Ulmer JB, Donnelly JJ, Parker SE, et al. Heterologous protection against influenza by injection of DNA encoding a viral protein. Science **1993**;259:1745-9.

21. McDonnell WM, Askari FK. DNA vaccines. N Engl J Med **1996**;334:42-5.

22. Wang R, Epstein J, Baraceros FM, et al. Induction of CD4(+) T cell-dependent CD8(+) type 1 responses in humans by a malaria DNA vaccine. Proc Natl Acad Sci U S A **2001**;98:10817-22.

23. Le TP, Coonan KM, Hedstrom RC, et al. Safety, tolerability and humoral immune responses after intramuscular administration of a malaria DNA vaccine to healthy adult volunteers. Vaccine **2000**;18:1893-901.

24. Tacket CO, Roy MJ, Widera G, Swain WF, Broome S, Edelman R. Phase 1 safety and immune response studies of a DNA vaccine encoding hepatitis B surface antigen delivered by a gene delivery device. Vaccine **1999**;17:2826-9.

25. Lu S, Arthos J, Montefiori DC, et al. Simian immunodeficiency virus DNA vaccine trial in macaques. J Virol **1996**;70:3978-91.

26. Egan MA, Charini WA, Kuroda MJ, et al. Simian immunodeficiency virus (SIV) gag DNA-vaccinated rhesus monkeys develop secondary cytotoxic T-lymphocyte responses and control viral replication after pathogenic SIV infection. J Virol **2000**;74:7485-95.

27. Barouch DH, Santra S, Schmitz JE, et al. Control of viremia and prevention of clinical AIDS in rhesus monkeys by cytokine-augmented DNA vaccination. Science **2000**;290:486-92.

28. Amara RR, Villinger F, Altman JD, et al. Control of a mucosal challenge and prevention of AIDS by a multiprotein DNA/MVA vaccine. Science **2001**;292:69-74.

29. MacGregor RR, Boyer JD, Ugen KE, et al. First human trial of a DNA-based vaccine for treatment of human immunodeficiency virus type 1 infection: safety and host response. J Infect Dis **1998**;178:92-100.

30. MacGregor RR, Ginsberg R, Ugen KE, et al. T-cell responses induced in normal volunteers immunized with a DNA-based vaccine containing HIV-1 env and rev. AIDS **2002**;16:2137-43.

31. Mwau M, Cebere I, Sutton J, Chikoti P, Winstone N, Wee E, Beattie T, Chen YH, Dorrell L, McShane H, Schmidt C, Brooks M, Patel S, Roberts J, Conlon C, Rowland-Jones S, Bwayo J, McMichael AJ, Hanke T. Stimulation of HIV-specific T cell responses by DNA and recombinant modified vaccinia virus Ankara vaccines in humans. J.Gen Virol. In press **2004**.

32. Ihata A, Watabe S, Sasaki S, et al. Immunomodulatory effect of a plasmid expressing CD40 ligand on DNA vaccination against human immunodeficiency virus type-1. Immunology **1999**;98:436-42.

33. Kim JJ, Ayyavoo V, Bagarazzi ML, et al. Development of a multicomponent candidate vaccine for HIV-1. Vaccine **1997**;15:879-83.

34. Kim JJ, Ayyavoo V, Bagarazzi ML, et al. In vivo engineering of a cellular immune response by coadministration of IL-12 expression vector with a DNA immunogen. J Immunol **1997**;158:816-26.

35. Kim JJ, Bagarazzi ML, Trivedi N, et al. Engineering of in vivo immune responses to DNA immunization via codelivery of costimulatory molecule genes. Nat Biotechnol **1997**;15:641-6.

36. Kusakabe K, Xin KQ, Katoh H, et al. The timing of GM-CSF expression plasmid administration influences the Th1/Th2 response induced by an HIV-1-specific DNA vaccine. J Immunol **2000**;164:3102-11.

37. Xin KQ, Hamajima K, Sasaki S, et al. IL-15 expression plasmid enhances cell-mediated immunity induced by an HIV-1 DNA vaccine. Vaccine **1999**;17:858-66.

38. Sin JI, Kim JJ, Boyer JD, Ciccarelli RB, Higgins TJ, Weiner DB. In vivo modulation of vaccine-induced immune responses toward a Th1 phenotype increases potency and vaccine effectiveness in a herpes simplex virus type 2 mouse model. J Virol **1999**;73:501-9.

39. Moore AC, Kong WP, Chakrabarti BK, Nabel GJ. Effects of antigen and genetic adjuvants on immune responses to human immunodeficiency virus DNA vaccines in mice. J Virol **2002**;76:243-50.

40. Kim JJ, Maguire HC, Jr., Nottingham LK, et al. Coadministration of IL-12 or IL-10 expression cassettes drives immune responses toward a Th1 phenotype. J Interferon Cytokine Res **1998**;18:537-47.

41. Min W, Lillehoj HS, Burnside J, Weining KC, Staeheli P, Zhu JJ. Adjuvant effects of IL-1beta, IL-2, IL-8, IL-15, IFN-alpha, IFN-gamma TGF-beta4 and lymphotactin on DNA vaccination against Eimeria acervulina. Vaccine **2001**;20:267-74.

42. Bamford RN, Grant AJ, Burton JD, et al. The interleukin (IL) 2 receptor beta chain is shared by IL-2 and a cytokine, provisionally designated IL-T, that stimulates T-cell proliferation and the induction of lymphokine-activated killer cells. Proc Natl Acad Sci U S A **1994**;91:4940-4.

43. Grabstein KH, Eisenman J, Shanebeck K, et al. Cloning of a T cell growth factor that interacts with the beta chain of the interleukin-2 receptor. Science **1994**;264:965-8.

44. Fehniger TA, Caligiuri MA. Interleukin 15: biology and relevance to human disease. Blood **2001**;97:14-32.

45. Waldmann TA, Tagaya Y. The multifaceted regulation of interleukin-15 expression and the role of this cytokine in NK cell differentiation and host response to intracellular pathogens. Annu Rev Immunol **1999**;17:19-49.

46. Waldmann TA, Dubois S, Tagaya Y. Contrasting roles of IL-2 and IL-15 in the life and death of lymphocytes: implications for immunotherapy. Immunity **2001**;14:105-10.

47. Lenardo MJ. Fas and the art of lymphocyte maintenance. J Exp Med **1996**;183:721-4.

48. Marks-Konczalik J, Dubois S, Losi JM, et al. IL-2-induced activation-induced cell death is inhibited in IL-15 transgenic mice. Proc Natl Acad Sci U S A **2000**;97:11445-50.

49. Zhang X, Sun S, Hwang I, Tough DF, Sprent J. Potent and selective stimulation of memory-phenotype CD8+ T cells in vivo by IL-15. Immunity **1998**;8:591-9.

50. Ku CC, Murakami M, Sakamoto A, Kappler J, Marrack P. Control of homeostasis of CD8+ memory T cells by opposing cytokines. Science **2000**;288:675-8.

51. Schluns KS, Williams K, Ma A, Zheng XX, Lefrancois L. Cutting edge: requirement for IL-15 in the generation of primary and memory antigen-specific CD8 T cells. J Immunol **2002**;168:4827-31.

52. Goldrath AW, Sivakumar PV, Glaccum M, et al. Cytokine requirements for acute and Basal homeostatic proliferation of naive and memory CD8+ T cells. J Exp Med **2002**;195:1515-22.

53. Becker TC, Wherry EJ, Boone D, et al. Interleukin 15 is required for proliferative renewal of virus-specific memory CD8 T cells. J Exp Med **2002**;195:1541-8.

54. Sadlack B, Kuhn R, Schorle H, Rajewsky K, Muller W, Horak I. Development and proliferation of lymphocytes in mice deficient for both interleukins-2 and -4. Eur J Immunol **1994**;24:281-4.

55. Willerford DM, Chen J, Ferry JA, Davidson L, Ma A, Alt FW. Interleukin-2 receptor alpha chain regulates the size and content of the peripheral lymphoid compartment. Immunity **1995**;3:521-30.

56. Kennedy MK, Glaccum M, Brown SN, et al. Reversible defects in natural killer and memory CD8 T cell lineages in interleukin 15-deficient mice. J Exp Med **2000**;191:771-80.

57. Lodolce JP, Boone DL, Chai S, et al. IL-15 receptor maintains lymphoid homeostasis by supporting lymphocyte homing and proliferation. Immunity **1998**;9:669-76.

58. Kim JJ, Trivedi NN, Nottingham LK, et al. Modulation of amplitude and direction of in vivo immune responses by co-administration of cytokine gene expression cassettes with DNA immunogens. Eur J Immunol **1998**;28:1089-103.

59. Oh S, Berzofsky JA, Burke DS, Waldmann TA, Perera LP. Coadministration of HIV vaccine vectors with vaccinia viruses expressing IL-15 but not IL-2 induces long-lasting cellular immunity. Proc Natl Acad Sci U S A **2003**;100:3392-7.

60. Hudgens MG. Estimating cumulative probabilities from incomplete longitudinal binary responses with application to HIV vaccine trials. Statistics in Medicine **2003**;22:463-79.

61. Petrovas C. IL-15 treatment of SIV-infected non-human primate. 11th conference on retroviruses and opportunistic infection, **2004**.

62. Kobayashi M, Fitz L, Ryan M, et al. Identification and purification of natural killer cell stimulatory factor (NKSF), a cytokine with multiple biologic effects on human lymphocytes. J Exp Med **1989**;170:827-45.

63. Villinger F, Miller R, Mori K, et al. IL-15 is superior to IL-2 in the generation of long-lived antigen specific memory CD4 and CD8 T cells in rhesus macaques. Vaccine **2004**;22:3510-21.

64. Kim JJ, Trivedi NN, Nottingham LK, et al. Modulation of amplitude and direction of in vivo immune responses by co-administration of cytokine gene expression cassettes with DNA immunogens. Eur J Immunol **1998**;28:1089-103.

65. Chehimi J, Starr SE, Frank I, et al. Natural killer (NK) cell stimulatory factor increases the cytotoxic activity of NK cells from both healthy donors and human immunodeficiency virus-infected patients. J Exp Med **1992**;175:789-96.

66. Kohl S, Sigaroudinia M, Charlebois ED, Jacobson MA. Interleukin-12 administered in vivo decreases human NK cell cytotoxicity and antibody-dependent cellular cytotoxicity to human immunodeficiency virus-infected cells. J Infect Dis **1996**;174:1105-8.

67. Romani L, Puccetti P, Bistoni F. Interleukin-12 in infectious diseases. Clin Microbiol Rev **1997**;10:611-36.

68. Ma X, D'Andrea A, Kubin M, et al. Production of interleukin-12. Res Immunol **1995**;146:432-8.

69. Takenaka H, Maruo S, Yamamoto N, et al. Regulation of T cell-dependent and -independent IL-12 production by the three Th2-type cytokines IL-10, IL-6, and IL-4. J Leukoc Biol **1997**;61:80-7.

70. Fantuzzi L, Gessani S, Borghi P, et al. Induction of interleukin-12 (IL-12) by recombinant glycoprotein gp120 of human immunodeficiency virus type 1 in human monocytes/macrophages: requirement of gamma interferon for IL-12 secretion. J Virol **1996**;70:4121-4.

71. Gately MK, Desai BB, Wolitzky AG, et al. Regulation of human lymphocyte proliferation by a heterodimeric cytokine, IL-12 (cytotoxic lymphocyte maturation factor). J Immunol **1991**;147:874-82.

72. Bertagnolli MM, Lin BY, Young D, Herrmann SH. IL-12 augments antigen-dependent proliferation of activated T lymphocytes. J Immunol **1992**;149:3778-83.

73. Gately MK, Wolitzky AG, Quinn PM, Chizzonite R. Regulation of human cytolytic lymphocyte responses by interleukin-12. Cell Immunol **1992**;143:127-42.

74. Scott P. IL-12: initiation cytokine for cell-mediated immunity. Science **1993**;260:496-7.

75. Germann T, Gately MK, Schoenhaut DS, et al. Interleukin-12/T cell stimulating factor, a cytokine with multiple effects on T helper type 1 (Th1) but not on Th2 cells. Eur J Immunol **1993**;23:1762-70.

76. Afonso LC, Scharton TM, Vieira LQ, Wysocka M, Trinchieri G, Scott P. The adjuvant effect of interleukin-12 in a vaccine against Leishmania major. Science **1994**;263:235-7.

77. Mahon BP, Ryan MS, Griffin F, Mills KH. Interleukin-12 is produced by macrophages in response to live or killed Bordetella pertussis and enhances the efficacy of an acellular pertussis vaccine by promoting induction of Th1 cells. Infect Immun **1996**;64:5295-301.

78. Lindblad EB, Elhay MJ, Silva R, Appelberg R, Andersen P. Adjuvant modulation of immune responses to tuberculosis subunit vaccines. Infect Immun **1997**;65:623-9.

79. Miller MA, Skeen MJ, Ziegler HK. Nonviable bacterial antigens administered with IL-12 generate antigen-specific T cell responses and protective immunity against Listeria monocytogenes. J Immunol **1995**;155:4817-28.

80. Germann T, Bongartz M, Dlugonska H, et al. Interleukin-12 profoundly up-regulates the synthesis of antigen-specific complement-fixing IgG2a, IgG2b and IgG3 antibody subclasses in vivo. Eur J Immunol **1995**;25:823-9.

81. Buchanan JM, Vogel LA, Van Cleave VH, Metzger DW. Interleukin 12 alters the isotype-restricted antibody response of mice to hen eggwhite lysozyme. Int Immunol **1995**;7:1519-28.

82. Rakhmilevich AL, Timmins JG, Janssen K, Pohlmann EL, Sheehy MJ, Yang NS. Gene gun-mediated IL-12 gene therapy induces antitumor effects in the absence of toxicity: a direct comparison with systemic IL-12 protein therapy. J Immunother **1999**;22:135-44.

83. Rakhmilevich AL, Janssen K, Turner J, Culp J, Yang NS. Cytokine gene therapy of cancer using gene gun technology: superior antitumor activity of interleukin-12. Hum Gene Ther **1997**;8:1303-11.

84. Rakhmilevich AL, Janssen K, Hao Z, Sondel PM, Yang NS. Interleukin-12 gene therapy of a weakly immunogenic mouse mammary carcinoma results in reduction of spontaneous lung metastases via a T-cell-independent mechanism. Cancer Gene Ther **2000**;7:826-38.

85. Weber SM, Shi F, Heise C, Warner T, Mahvi DM. Interleukin-12 gene transfer results in CD8-dependent regression of murine CT26 liver tumors. Ann Surg Oncol **1999**;6:186-94.

86. Boyer JD, Cohen AD, Ugen KE, et al. Therapeutic immunization of HIV-infected chimpanzees using HIV-1 plasmid antigens and interleukin-12 expressing plasmids. AIDS **2000**;14:1515-22.

87. Yoshida S, Kashiwamura SI, Hosoya Y, et al. Direct immunization of malaria DNA vaccine into the liver by gene gun protects against lethal challenge of Plasmodium berghei sporozoite. Biochem Biophys Res Commun **2000**;271:107-15.

88. Shan M, Liu K, Fang H. [DNA vaccination of the induction of immune responses by codelivery of IL-12 expression vector with hepatitis C structural antigens]. Zhonghua Gan Zang Bing Za Zhi **1999**;7:236-9.

89. Operschall E, Schuh T, Heinzerling L, Pavlovic J, Moelling K. Enhanced protection against viral infection by co-administration of plasmid DNA coding for viral antigen and cytokines in mice. J Clin Virol **1999**;13:17-27.

90. Hanlon L, Argyle D, Bain D, et al. Feline leukemia virus DNA vaccine efficacy is enhanced by coadministration with interleukin-12 (IL-12) and IL-18 expression vectors. J Virol **2001**;75:8424-33.

91. Noormohammadi AH, Hochrein H, Curtis JM, Baldwin TM, Handman E. Paradoxical effects of IL-12 in leishmaniasis in the presence and absence of vaccinating antigen. Vaccine **2001**;19:4043-52.

92. Iwasaki A, Dela Cruz CS, Young AR, Barber BH. Epitope-specific cytotoxic T lymphocyte induction by minigene DNA immunization. Vaccine **1999**;17:2081-8.

93. Tellez I, Sabbaj S, Bansal A, Goepfert P, Evans TG, Graham BS, Ginsberg R, Weiner D, Corey L, Weinhold K, Mulligan M. HIV-specific helper and cytotoxic T cell responses in seronegative volunteers immunized with a high dose HIV-1 gag-pol DNA vaccine [abstract 656]. 7th conference on retroviruses and opportunistic infections (San Francisco), **2000**.

94. Tellez I. HIV-specific T-cell responses in seronegative volunteers immunized with an HIV-1 gag-pol DNA vaccine. 7th conference on retroviruses and opportunistic infections, **2000**.

95. Gilbert PB, Chiu YL, Allen M, et al. Long-term safety analysis of preventive HIV-1 vaccines evaluated in AIDS vaccine evaluation group NIAID-sponsored Phase I and II clinical trials. Vaccine **2003**;21:2933-47.

96. Pachuk CJ, Ciccarelli RB, Samuel M, et al. Characterization of a new class of DNA delivery complexes formed by the local anesthetic bupivacaine. Biochim Biophys Acta **2000**;1468:20-30.

97. Cohen GB, Islam SA, Noble MS, et al. Clonotype tracking of TCR repertoires during chronic virus infections. Virology **2002**;304:474-84.

Protocol history

The Protocol Team may modify the original version of the protocol. Modifications are made to HVTN protocols via clarification memos, letters of amendment, or full protocol amendments. HVTN protocols are modified and distributed according to the standard HVTN procedures as described in the HVTN MOP (Organization and Policy>Vaccine Selection and Protocol Development).

The table below describes the version history of, and modifications to, Protocol HVTN 063.

Protocol history and modifications

Date	Protocol version	Protocol modification	Summary of modifications
11-Apr-06	Version 2	Full Protocol Amendment 1	Item 1 Eliminated from Part B: Group 6; only Groups 5 and 7 to enroll<br>Item 2 Revised (per Item 1): Sample informed consent form for Part B<br>Item 3 Reduced: Specimen collection volumes<br>Item 4 Added: Sample addendum to informed consent for Part A (Appendix G)<br>Item 5 Deleted: All references to HVTN 061<br>Item 6 Incorporated: Minor corrections throughout<br>Item 7 Updated: Protocol Team contact list<br>Item 8 Incorporated: Protocol modifications to Version 1 into Version 2
14-Feb-06	Version 1	Clarification Memo 3	Item 1 Discontinued: Specimen collection of blood plasma at baseline for HIV-1 RNA PCR<br>Item 2 Replaced: Viral and Rickettsial Disease Laboratory (Richmond, CA) by University of Washington Virology Specialty Laboratory (Seattle, WA)<br>Item 3 Changed: Tube type for HIV RNA PCR specimens from PPT to EDTA
20-Oct-05	Version 1	Clarification Memo 2	Item 1 Discontinued: Specimen collection for HIV DNA PCR testing
15-Jul-05	Version 1	Clarification Memo 1	Item 1 Corrected: Sites to instruct participants to contact site daily during reactogenicity assessment period<br>Item 2 Revised: Active syphilis infection may be assessed by locally appropriate test<br>Item 3 Clarified: Social impact assessment to be performed<br>Item 4 Assigned: IND number<br>Item 5 Updated: Team member contact information<br>Item 6 Corrected: Minor typos
25-Apr-05	Version 1	Original protocol	Original protocol

Protocol team

Information on protocol team member designation and responsibilities and on the protocol development process can be found in the HVTN MOP.

Contact information for protocol team members, HVTUs, and labs can be found in the *HVTN 063 Study Specific Procedures*.

Chair	Lindsey Baden<br>Harvard University<br>617-732-6801<br>lbaden@partners.org
Co-chair	Xia Jin<br>U. of Rochester Med. Ctr.<br>585-275-6515<br>xia_jin@urmc.rochester.edu
Clinical trials physicians	Marnie Elizaga<br>HVTN Core<br>650-854-4984<br>melizaga@hvtn.org
Vaccine developer representatives	Michael Lubeck<br>Wyeth Vaccines Research<br>845-602-1275<br>lubeckm@wyeth.com
	John Eldridge<br>Wyeth Vaccines Research<br>845-602-4940<br>eldridj@wyeth.com
	Dagna Laufer<br>Wyeth Vaccines Research<br>484-865-2011<br>lauferd@wyeth.com
Laboratory program representative	Cristine Cooper<br>HVTN Laboratory Operations<br>206-667-5812<br>cjcooper@hvtn.org
Medical officer	Massimo Cardinali<br>NIAID Vaccine Clinical Research Branch<br>301-451-2736<br>mcardinali@niaid.nih.gov
Pharmacist	Scharla Estep<br>PAB, DAIDS, NIAID<br>301-435-3746<br>sr72v@nih.gov
Statisticians	Barbara Metch<br>SCHARP<br>206-667-4656<br>bmetch@scharp.org
	Maggie Wang<br>SCHARP<br>206-667-7451<br>maggie@scharp.org
Clinic coordinator	Lizanne Noble<br>Brigham and Women's Hospital Division of Infectious Disease<br>617-732-5394<br>lnoble@partners.org
Community Advisory Board members	Paul Williams<br>SLU CAB<br>314-533-4189<br>williamspa@sbcglobal.net
Regulatory affairs	Renée Holt<br>SCHARP<br>206-667-7505<br>renee@scharp.org
Clinical affairs	Hsiu-Ying Huang<br>SCHARP<br>206-667-5055<br>hyhuang@scharp.org
Protocol operations manager	Drienna Holman<br>SCHARP<br>206-667-6695<br>dholman@scharp.org
Protocol specialist	Shelly Mahilum<br>SCHARP<br>206-667-6476<br>shelly@scharp.org
Community education	Gail Broder<br>HVTN Core<br>206-667-7348<br>gbroder@hvtn.org
Protocol development coordinator	Richard Newman<br>HVTN Core<br>206-667-5687<br>rnewman@hvtn.org

This page is intentionally blank

APPENDICES

Appendix A: Sample informed consent form (Part A)

Title: A Phase I clinical trial to evaluate the safety and immunogenicity of HIV-1 *gag* DNA vaccine alone or with *IL-15* DNA, boosted with HIV-1 *gag* DNA + *IL-15* DNA, or HIV-1 *gag* DNA + *IL-12* DNA, in healthy, HIV-1 uninfected adult participants

Short title: A safety study of HIV-1 *gag* DNA vaccine and *IL-15* DNA adjuvant

Thank you for your interest in this study.

The HIV Vaccine Trials Network (HVTN) and [site] are conducting a research study (an experiment).[1]

Site: Footnotes connecting template language to CFR and ICH guidelines are intended for writers, reviewers, and IRBs/IECs. Delete before giving consent form to participant unless your site favors inclusion

This study has 2 parts (A and B). You are being asked to join Part A, which is testing an experimental vaccine against HIV, the virus that causes AIDS. Vaccines are given to prevent infection or fight disease. Part A of this study is also testing an experimental adjuvant. An adjuvant is a substance that helps the body respond to a vaccine. Part B, and the study as a whole, will be testing two experimental vaccines and four experimental adjuvants.

We are testing the vaccines and adjuvants to see if they are safe to give to people and well tolerated. We are also testing to see how your immune system responds to them. The immune system protects your body against infections.[2]

This study is paid for by the US National Institutes of Health (NIH). The researcher in charge of this study at this clinic is
[Insert name of the site PI].

Participation in this study is voluntary. You do not have to join.[3] If you join the study and stay in it, you will be in it for about 12 months.[4] About 156 people will take part in this study ([#] at this clinic), 48 in Part A and 108 in Part B.[5]

This is an *informed consent* form. It answers these questions:

1. What is being tested?
2. How do I join this study?
3. What will happen during clinic visits?

[1] **21 CFR 50.25.a.1** A statement that this study involves research… **ICH 4.8.10.a** That the trial involves research.

[2] **21 CFR 50.25.a.1** …an explanation of the purposes of the research… **ICH 4.8.10.b** The purpose of the trial.

[3] **21 CFR 50.25.a.8** A statement that participation is voluntary…. **ICH 4.8.10.m** That the subject's participation in the trial is voluntary….

[4] **21 CFR 50.25.a.1** …and the expected duration of the subject's participation… **ICH 4.8.10.s** The expected duration of the subject's participation in the trial.

[5] **21 CFR 50.25.b.6** The approximate number of subjects involved in this study. **ICH 4.8.10.t** The approximate number of subjects involved in the trial.

4. What will happen to my blood samples?
5. What are the risks and inconveniences?
6. What are the benefits?
7. What are the alternatives to participating?
8. What are my responsibilities?
9. Can the researchers stop injections or take me out of this study?
10. What if I get HIV during this study?
11. What if I choose to leave this study?
12. Who makes sure this study is done correctly?
13. How will my private information be protected?
14. What if the experimental vaccine injures me or makes me sick?
15. What if the researchers learn new information during this study?
16. Will I have to pay?
17. Will I be paid?
18. Who should I call if I have questions or problems?

Read this consent form carefully. Please ask questions about anything you do not understand. The clinic staff will talk with you about the information in this form, and test your understanding. We encourage you to ask questions about this study at any time.

Site: Add the following paragraph (or one like it) if appropriate:

You may want to talk to others (such as family, friends, or your doctor) before you decide whether to join this study.

We will ask you to sign this form. Signing means:

- you have read the form (or had it explained to you),
- you understand it, and
- you agree to join this study.

We will give you a copy of this form.

1. What is being tested?[6]

The vaccine is called HIV-1 *gag* DNA vaccine, which will be called *gag* DNA vaccine from now on. It was developed by Wyeth Vaccines Research.

The adjuvant is called *IL-15* DNA.

The vaccine and adjuvant in this study are made in a laboratory. The vaccine is not made from live HIV or from HIV-infected cells. It does not contain live or killed HIV. ***It is impossible to get HIV infection or AIDS from these experimental vaccines.***

The vaccine and adjuvant are experimental. They have not been approved for treating or preventing HIV infection. The US Food and Drug Administration (FDA) allows their use in research only.

[6] **ICH 4.8.10.c** The trial treatment(s) and the probability for random assignment to each treatment.

gag *DNA vaccine*

This experimental vaccine contains a piece of DNA made in the laboratory. DNA is a natural substance in the body that tells the body to make proteins. Proteins are natural substances that the body uses to build and maintain itself as well as protect itself against disease. The DNA tells the body to make only part of a protein called Gag that is found in HIV. Your body's immune system may respond to this protein by making cells that recognize and fight against this type of HIV protein.

IL-15 *DNA adjuvant*

The *IL-15* DNA adjuvant is DNA that will tell your body to make IL-15, a normal protein in the body. IL-15 can help the immune system keep a "memory" of how to fight certain germs, so it will be ready to fight those germs again later.

Placebo

Not everyone in this study will get an experimental vaccine. Some people will get a placebo, an inactive substance that does not contain vaccine. In this study, the placebo is sterile salt water. We give placebo to some people, and compare the results from the people who got the experimental vaccines with the results from people who got the placebo. This helps us measure the effects of the experimental vaccines.

Being assigned to a group

You will be assigned to get experimental vaccine or placebo at random, like the toss of a coin. You have an 83% (5 in 6) chance of getting an experimental vaccine. You have a 17% (1 in 6) chance of getting placebo. This is a double-blind study. That means that neither you nor the researchers at your clinic know which product (experimental vaccine or placebo) you are getting until after the study is over.

Part A will test the *gag* DNA vaccine alone and with the *IL-15* DNA adjuvant. There will be 4 groups. In each group, 10 people will receive the experimental vaccine and 2 people will receive the placebo. These groups test the vaccine with different strengths (doses) of the adjuvant. Each time a higher dose is used, we will first see that there were no serious safety concerns at the lower doses. Clinic staff at this site can tell you which of the groups is currently enrolling.

In Group 1, people will receive 3 shots of the *gag* DNA vaccine alone, or placebo.

Group 2 will receive 3 shots of the *gag* DNA vaccine with a low dose of the *IL-15* DNA adjuvant, or placebo.

Group 3 will receive 3 shots of the *gag* DNA vaccine with a medium dose of the *IL-15* DNA adjuvant (5 times higher than the dose given to Group 2), or placebo.

Group 4 will receive 3 shots of the *gag* DNA vaccine with a high dose of the *IL-15* DNA adjuvant (15 times higher than the dose given to Group 2), or placebo.

The following table shows the groups and the study products they get.

	Number of people	First injection	Number of months after first injection: 1	Number of months after first injection: 3
Group 1	10	*gag* DNA	*gag* DNA	*gag* DNA
	2	placebo	placebo	placebo
Group 2	10	*gag* DNA + low dose *IL-15* DNA	*gag* DNA + low dose *IL-15* DNA	*gag* DNA + low dose *IL-15* DNA
	2	placebo	placebo	placebo
Group 3	10	*gag* DNA + medium dose *IL-15* DNA	*gag* DNA + medium dose *IL-15* DNA	*gag* DNA + medium dose *IL-15* DNA
	2	placebo	placebo	placebo
Group 4	10	*gag* DNA + high dose *IL-15* DNA	*gag* DNA + high dose *IL-15* DNA	*gag* DNA + high dose *IL-15* DNA
	2	placebo	placebo	placebo

2. How do I join this study?[7]

To see if you can take part in this study, you will have some screening procedures. Screening will include:

- questions about your medical history
- personal questions about your sexual behavior and any drug use
- physical exam

Site: In the following item, revise units of measure as appropriate

- blood tests to check for diseases such as HIV, syphilis, and hepatitis, and to check your general health; about 50 mL, or 3 tablespoons, of your blood will be drawn
- urine sample
- pregnancy test (for women)

You may have already signed a consent form for screening. If not, you will need to sign this consent form before we can do the screening.

Information from the screening can only be used for 56 days (8 weeks). If you are not enrolled into the study by then, you may need to be screened again.

[7] **21 CFR 50.25.a.1** …a description of the procedures to be followed… **ICH 4.8.10.d** The trial procedures to be followed, including all invasive procedures.

The results of the screening tests may show that you cannot join this study. We will explain the results to you, and tell you about places where you can get support and medical care if you need it.

If you are pregnant or breastfeeding, you cannot join this study.

If you have an allergy to bupivacaine (Marcaine) or other local anesthetics such as lidocaine (Xylocaine), mepivicaine (Polocaine/Carbocaine), etidocaine (Duranest), or prilocaine, you cannot join this study.

If you are HIV positive, you cannot join this study. The clinic staff will counsel you about your HIV infection and about telling your partner(s). The clinic staff will tell you about places where you can get support and medical care, and about other studies you may want to join.

3. What will happen during clinic visits?[7]

Site: Give number of visits and range of visit lengths for Part A only

You will visit the clinic about [#] times. The length of visits will vary from [#] to [#] hours.

If necessary, we may ask you to return to the clinic for more visits and/or lab tests.

You will be tested for HIV regularly. You will be counseled about the test and your results. You will also get regular counseling on how to reduce your risk of getting HIV.

At some visits, we will ask you questions to see if you have experienced personal problems or discrimination because of being in an HIV vaccine study. You can tell us about these problems at any time. We will also ask you personal questions about your sexual behavior and any drug use.

The following table shows what will happen at each study visit.

Procedure	Screening visit	1st injection visit	Time after 1st injection visit (in months)							
			½	1	1½	3	3½	6	9	12
Injection		√		√		√				
Medical history	√									
Complete physical	√									√
Brief physical		√	√	√	√	√	√	√	√	
Urine test	√		√		√		√			
Blood drawn	√	√	√		√		√	√	√	√
Pregnancy test (women)	√	√		√		√		√		
HIV testing/counseling	√						√	√	√	√
Interview/questionnaire	√	√	√	√	√	√	√	√	√	√

Not shown in this table is a time after all study participants have completed their last scheduled visit when you can find out what products you received.

At each visit, we will check for any changes in your health. We will ask you how you are feeling, and if you are taking any medication. At some visits we will examine you, and ask you to give urine or blood samples. We will *not* test your blood or urine for illegal drugs.

After all participants have had their final clinic visit (or sooner if necessary), we will tell you whether you got the experimental vaccine or placebo. To do so, we may ask you to come to the clinic one more time. Not all participants join this study at the same time, so you may have to wait as much as a year after your final clinic visit to learn what you got.

Injections

You will get an injection (shot) at 3 of your clinic visits. If you are a woman, you will have a pregnancy test before each injection. The injections will be given in the muscle of your upper arm by syringe and needle. Usually the injections are given in the same arm.

After each injection, you will stay in the clinic for at least 25 to 45 minutes. Clinic staff will watch you for possible reactions to the injection.

You will be asked to record your temperature and other side effects on a symptom log the evening of the injection and for the following 3 days, or longer if necessary. You will be asked to contact the clinic daily to report these symptoms. The clinic staff may ask you to return to the clinic if necessary. It is very important to stay in touch with the clinic staff.

If you have serious reactions, we may decide that you should not get any more injections. If that happens, we will ask you to return for other visits and tests, to check your health and to look for an immune response to any injections you got earlier.

Blood samples

Site: In this subsection, revise units of measure as appropriate

At some visits, we will take samples of your blood. The amount will depend on the lab tests we need to do. It will be some amount between 15 mL and 365 mL (3 tablespoons to 1½ cups). The total amount of blood taken from you during this study will be no more than 1540 mL (about 6½ cups). To compare, people who donate blood can give about 500 mL (about 2 cups) every 8 weeks.

4. What will happen to my blood samples?

Use in this study

We will use some of your blood for safety testing, to check your health and see if you have side effects. We will tell you the results of lab tests at your next visit, or sooner if necessary.

We will use some of your blood to test your immune response to the experimental vaccine or placebo. We may also test your immune response

to other vaccines you may have received, or infectious agents, such as viruses or bacteria, that you may have been exposed to in the past.

In addition, a genetic test called *HLA typing* may be performed. HLA stands for human leukocyte antigen—a tiny marker on your cells that helps protect the body from infections. You inherited your HLA type from your father and mother. We think that people with different HLA types may respond differently to the experimental vaccine. If HLA typing is done on your blood, this will not affect your participation in this study.

Tests of immune response are for HIV-related or vaccine-related research only (not to check your health), so we will not tell you or the clinic the results.

Site: Per HVTN policy, the following passage must be retained verbatim:

Storage and future testing

We will store other samples of your blood for future research that is not a part of this study. This may include genetic testing other than HLA typing. Your samples would be used for HIV-related or vaccine-related research only. An Institutional Review Board or Independent Ethics Committee, which watches over the safety and rights of research participants, must approve any research studies using your samples. Your samples will not be sold.

Your samples may contribute to a new invention or discovery. There is no plan for you to share in any money or other benefits resulting from this invention or discovery.

Your samples will be stored indefinitely. You cannot be in this study if you do not wish to have your samples stored for future research.

The researchers do not plan to contact you or your health care provider with results from future studies using your blood. This is because the procedures in research are often experimental. If the researchers decide that a specific test result would provide important information for your health, we will try to contact you. If you want this information, tell the clinic staff. Always let the study clinic know if you change your address and/or phone number.

5. What are the risks and inconveniences?[8]

Being in this study may harm you. It also keeps you from doing some things. You may find these restrictions inconvenient.

This section describes the risks and restrictions we know about. There may be unknown risks, even serious ones. These unknown risks could affect

[8] **21 CFR 50.25.a.2** A description of any reasonably foreseeable risks or discomforts to the subject. **21 CFR 50.25.b.1** A statement that the particular treatment or procedure may involve risks to the subject (or to the embryo or fetus, if the subject is or may become pregnant) which are currently unforeseeable. **ICH 4.8.10.g** The reasonably foreseeable risks or inconveniences to the subject and, when applicable, to an embryo, fetus, or nursing infant.

you, or your fetus if you become pregnant. If we learn about new risks during this study, we will tell you.[9]

Risks of injections

Injections can cause pain, soreness, redness, and swelling on the part of your body where you got the injection. On rare occasions, they may cause bacterial infection at the part of your body where you got the injection.

Risks of vaccination

Vaccines can cause fever, chills, rash, aches and pains, muscle aches, muscle damage, nausea, headache, dizziness, fatigue, and feeling generally unwell.

We do not know if these experimental vaccines will change your response to an approved HIV vaccine if you receive one in the future. Currently, there is no approved and licensed HIV vaccine. If such a vaccine becomes available in the future, we do not know whether getting the experimental vaccines in this study will cause your body to respond differently to a licensed vaccine, changing your body's ability to prevent HIV infection and disease. Your body's ability to prevent HIV infection and AIDS may become better or worse, or stay the same.

Allergic reaction

After receiving any of the study products, you could have an allergic reaction, like a rash, hives, or even difficulty breathing. *Allergic reactions can be life threatening.* The clinic staff will watch you for at least 25 to 45 minutes after each injection (the time when most allergic reactions occur) and give you treatment if you need it. People with a known allergy to bupivacaine (Marcaine) or related local anesthetics cannot participate in this study.

General risks of DNA vaccines

Possible risks related to DNA vaccines include: muscle damage at the site of the injection, the production of antibodies which might react with normal body tissues and cause an autoimmune disease (a disease in which the body attacks its own cells), and insertion of the vaccine DNA into the body's DNA. This could lead to cancer or unknown side effects. Although these risks are possible, only muscle damage at the site of injection has been seen so far in animals or people. Other experimental DNA vaccines have also been given to thousands of people since 1995. These vaccines have not caused serious side effects, although we do not yet have long-term safety information about the people in these studies.

[9] **ICH 4.8.10.p** That the subject or the subject's legally acceptable representative will be informed in a timely manner if information becomes available that may be relevant to the subject's willingness to continue participation in the trial. **21 CFR 50.25.b.5** A statement that significant new findings developed during the course of the research which may relate to the subject's willingness to continue participation will be provided to the subject.

Risks of gag *DNA vaccine*

The *gag* DNA vaccine in this study has not been given to people before, so we do not know all of the risks or side effects. However, DNA *gag* vaccines similar to this one have been tested in several hundred people with no known serious side effects. The *gag* DNA vaccine in this study was tested in mice and rabbits with no serious side effects. However, animal tests may not show what will happen in people. The *gag* DNA vaccine is being tested in people in the HVTN 060 study , and those results will be reviewed to make sure that there are no serious safety concerns before they are given in this study.

Risks of IL-15 DNA *adjuvant*

The *IL-15* DNA adjuvant in this study has not been given to people before, so we do not know all the risks or side effects. A similar *IL-15* DNA vaccine adjuvant has been tested in animals with no serious side effects. A few rabbits developed a purple area at the injection site for a few days, which was not serious.

Your body makes IL-15 protein naturally. This *IL-15* DNA adjuvant will cause your body to make a little bit more of this protein. High amounts of IL-15 protein are seen in some people with autoimmune diseases (diseases where the body attacks its own cells). The IL-15 protein alone may not cause autoimmune disease, but it may play a part in getting such a disease. In theory, having your body make more IL-15 protein than it usually does may put you at greater risk for autoimmune disease. We think the risk of this happening is low, because of the small amount of extra IL-15 protein your body would make after getting the adjuvant.

Risks of bupivacaine

The *gag* DNA vaccine and the *IL-15* DNA adjuvant contain bupivacaine. Bupivacaine helps the DNA get into the muscle. It is an anesthetic, similar to the numbing medicine used by dentists. Bupivacaine, like all medicines, can have certain side effects. When bupivacaine was given to people at a strength 50 times higher than what we will use in this study, some people had serious health problems. The side effects were rare, but included nervous system or heart problems or even death due to high levels of bupivacaine in the blood. This may happen as a result of accidental injection into the bloodstream, overdose, or slow breakdown of the drug. Nervous system side effects can include confusion, anxiety, dizziness, blurred vision, shaking, or seizures. Heart side effects can include decreased heart pumping, fast heart rate, low blood pressure, or abnormal heartbeats. Some less serious side effects include nausea, vomiting, or chills. Study nurses will be careful to avoid injecting bupivacaine into the bloodstream, but even with their best efforts it could possibly happen. Because you will be getting a much lower dose of bupivacaine if you get the *gag* DNA vaccine or *IL-15* DNA adjuvant, and it is not likely that you

will get an injection directly into your bloodstream, we think the chances of your experiencing these side effects are low.

Risks of gag *DNA and* IL-15 *DNA adjuvant given together*

Since this combination of *gag* DNA vaccine and *IL-15* DNA has not been give to people before this study, all possible risks or side effects are currently not known. Animals have had the vaccine and adjuvant together in doses similar to or larger than those planned to be given in this study without problems. However, animal tests may not show what will happen in people.

Blood drawing

Drawing blood may cause pain and bruising. On rare occasions, it may cause bacterial infection at the part of your body where the blood is taken. Sometimes, drawing blood causes people to feel lightheaded or to faint. Some people, especially women, may become anemic (have a low red blood cell count).

Personal problems

Some participants in other HIV vaccine studies have reported experiencing personal problems because of their participation. Spouses, other family members, or sexual partners have sometimes reacted by:

- becoming angry when a participant joined a study without consulting them
- worrying that the test vaccine would be harmful
- assuming that the participant was infected with HIV and shunning them
- assuming that the participant is engaging in certain sexual activities or drug use, and treating them unfairly

On rare occasions, a participant has reported losing a job because of being in an HIV vaccine study. This was either because the study took too much time away from work, or because the employer thought the participant was HIV infected or at high risk for HIV.

If genetic testing (such as HLA typing) is performed on your blood, there is a very small chance that the results will cause personal problems. Information from this test may suggest you are at risk for certain diseases. This does not mean you will get a disease, but if your test results were known, you could have trouble getting insurance or a job. This risk is extremely small, because the test results do not identify you by name. They do not become part of your medical records.

To help avoid these problems, talk with the study staff if you have to get HIV testing done outside this study. You can get an ID card that shows

you joined the study. The card also lists a toll-free number you can call for help or information.

Clinic staff will help you with personal problems you may experience because of being in this study.

HIV exposure

If you are exposed to HIV at some time after getting an experimental vaccine, we do not know what will happen. The experimental vaccine could increase or decrease, or have no effect on:

- your risk of becoming infected with HIV if exposed
- the time it takes to develop AIDS after being infected
- the course of HIV infection

We do not know if getting the experimental vaccine will protect you from HIV. This study will not answer that question. In the past, some people have become infected with HIV even though they got an experimental vaccine. The experimental vaccine did not cause the HIV infection, but did not prevent infection in these cases. Because we do not know the effect of the experimental vaccine, *we ask you not to do anything that may expose you to HIV, like having unprotected sex or sharing needles or injection equipment.*

False positive HIV test

Standard HIV tests look for antibodies (made by cells in your immune system) that recognize HIV. The experimental vaccine may cause your body to produce these antibodies. In this case, the standard HIV test could show a positive result. This does not mean you are infected with HIV—the test result could be a *false positive*.

If this happens, we will do further tests to confirm that you are not infected with HIV. If the experimental vaccine caused the false positive result, we do not know how long the HIV test will stay positive. We will offer retesting free of charge as long as the positive HIV test is due to the experimental vaccine.

If you are tested for HIV outside this study, a false positive result may cause you trouble. You may have trouble with:

- insurance
- medical/dental care
- travel to other countries
- employment
- military service

Blood banks and medical institutions know that an experimental vaccine may give a false positive result. Still, if you continue to have a false

positive result after the study you will have trouble donating blood, body fluids, body tissues, or organs. You may even be permanently banned from donating.

To help with these situations, or to prevent discrimination, we can talk to insurance companies, employers, and others to explain that you are in a study. We would do this only at your request and with your written permission. You can also get an ID card that shows you joined the study. The card also lists a toll-free number you can call for help or information.

Restrictions

While you are in this study, there are things you cannot do.

- Because of the risk of a false positive HIV test, you should get your HIV testing done only at the clinic. If you have to be tested for HIV outside this study, please talk to the clinic staff.
- You must not donate blood, body tissues, body fluids, or organs.
- You may not be able to join other medical research studies. If you are thinking about joining another study, please talk to the clinic staff.
- After you are finished with this study, you may not be able to join other HIV vaccine studies.

We ask you not to do anything that may expose you to HIV, like having unprotected sex or sharing needles or injection equipment.

Pregnancy risks

Women should not become pregnant during this study (about 12 months), because we do not know how the experimental vaccines or adjuvants could affect the fetus. If you are a woman having sex that could lead to pregnancy, you must agree to use effective birth control starting at least 21 days prior to enrollment and continuing until after your last clinic visit. This means using any of the following methods:

- birth control drugs that prevent pregnancy—given by pills, shots, patches, vaginal rings, or inserts under the skin
- male or female condoms, with or without a cream or gel that kills sperm
- diaphragm or cervical cap with a cream or gel that kills sperm
- intrauterine device (IUD)
- any other contraceptive method approved by the researchers

6. What are the benefits?

This study may not benefit you personally. Being in this study may help in the search for an HIV vaccine.[10]

Site: Add information about other benefits (health care, tests, etc.) as appropriate

7. What are the alternatives to participating?

You may choose not to join this study. Other services you receive at this institution will not be affected.[11]

If you choose not to join this study, you may join a different experimental HIV vaccine study, if one is available and you are eligible.[12]

8. What are my responsibilities?[13]

If you join this study, you will be asked to:

- come to all clinic visits
- record your temperature and other side effects on the symptom log , and report these results to the clinic staff, the evening of the injection and for the following 3 days, or longer if necessary
- tell clinic staff about any symptoms or side effects you have
- tell clinic staff about any medications you are taking
- tell clinic staff before getting any other vaccines, such as a flu shot
- follow instructions from the clinic staff
- stay in touch with the clinic staff; tell them if you have moved or if you want to leave the study.
- get your HIV testing done only at the clinic
- women: avoid pregnancy until after your last clinic visit

The clinic staff will share the HVTN Participant's Bill of Rights and Responsibilities with you. That document tells more about your rights and responsibilities.

9. Can the researchers stop injections or take me out of this study?

Your injections may be stopped if:

[10] **21 CFR 50.25.a.3** A description of any benefits to the subject or to others which may reasonably be expected from the research. **ICH 4.8.10.h** The reasonably expected benefits. When there is no intended clinical benefit to the subject, the subject should be made aware of this.

[11] **21 CFR 50.25.a.8** …that refusal to participate will involve no penalty or loss of benefits to which the subject is otherwise entitled…. **ICH 4.8.10.m** …that the subject may refuse to participate…without penalty or loss of benefits to which the subject is otherwise entitled.

[12] **21 CFR 50.25.a.4** A disclosure of appropriate alternative procedures or courses of treatment, if any, that might be advantageous to the subject. **ICH 4.8.10.i** The alternative procedure(s) or course(s) of treatment that may be available to the subject, and their important potential benefits and risks.

[13] **ICH 4.8.10.e** The subject's responsibilities.

- getting injections would be harmful to you
- you become pregnant
- you need a treatment, and the treatment and the experimental vaccine might interfere with each other
- the experimental vaccine or placebo is no longer available

If you must stop getting injections before this study is over, we will ask you to come back to the clinic to check your health and your immune response.

You may be taken out of this study entirely if:

- you cannot or do not attend the study visits
- you do not follow instructions
- you get infected with HIV
- the study is canceled.[14]

10. What if I get HIV during this study?

If you get infected with HIV during this study, the clinic staff will do additional HIV testing to confirm the infection and learn more about it. You will not be able to stay in this study.

Site: Include (as needed) required reporting information, and modify (as appropriate) information about provision of treatment

The clinic staff will counsel you about your HIV infection and about telling your partner(s). Medical care and treatment for HIV infection are not a part of this study. The clinic staff will tell you about places where you can get support and medical care, and about other studies you may want to join.

11. What if I choose to leave this study?[15]

If you join this study, you can leave it at any time. If you leave this study, you will not lose any benefits or rights you would normally have or be disadvantaged in any way.[16]

If you decide to leave this study, please tell the clinic staff. We will ask you to come back to the clinic at least once to check your health and look for an immune response to study injections.

[14] **21 CFR 50.25.b.2** Anticipated circumstances under which the subject's participation may be terminated by the investigator without regard to the subject's consent. **ICH 4.8.10.r** The foreseeable circumstances and/or reasons under which the subject's participation in the trial may be terminated.

[15] **21 CFR 50.25.b.4** The consequences of a subject's decision to withdraw from the research and procedures for orderly termination of participation by the subject.

[16] **21 CFR 50.25.a.8** …that the subject may discontinue participation at any time without penalty or loss of benefits to which the subject is otherwise entitled. **ICH 4.8.10.m** …the subject may…withdraw from the trial, at any time, without penalty or loss of benefits to which the subject is otherwise entitled.

Like everyone else in this study, you will have to wait until all participants complete their final study visit to find out whether you got the experimental vaccine or the placebo.

12. Who makes sure this study is done correctly?

Several groups watch over this study to see that your rights are protected and that the researchers are following this study plan.[17] These groups include:

Site: Modify list for non-US monitors and IBC as appropriate

- the US National Institutes of Health (NIH)
- the US Food and Drug Administration (FDA)
- a local Institutional Review Board or Independent Ethics Committee
- Wyeth Vaccines Research and people who work for them
- the HIV Vaccine Trials Network and people who work for them
- [Insert name of local regulatory authority as appropriate]

A local Community Advisory Board is also involved in this study. Community Advisory Boards assist scientists in developing research studies and review these studies for issues important to the community. The Community Advisory Board will not have access to medical information that can identify you.

13. How will my private information be protected?[18]

US sites: Check HIPAA authorization for conflicts with this section

We will do our best to protect your private information. Your records are kept in locked files at the clinic. On most records, we use a participant ID number, not your name.

The results of this study may be published. Any publication will not use your name or identify you personally.

Most of the groups who watch over this study may review your medical records. Your records may also be reviewed by clinic staff, and by the vaccine developer or people who work for them. Reviewers will keep your records private.

[17] **ICH 4.8.10.n** That the monitor(s), the auditor(s), the IRB/IEC, and the regulatory authority(ies) will be granted direct access to the subject's original medical records for verification of clinical trial procedures and/or data, without violating the confidentiality of the subject, to the extent permitted by the applicable laws and regulations and that, by signing a written informed consent form, the subject or the subject's legally acceptable representative is authorizing such access.

[18] **21 CFR 50.25.a.5** A statement describing the extent, if any, to which confidentiality of records identifying the subject will be maintained and that notes the possibility that the Food and Drug Administration may inspect the records. **ICH 4.8.10.o** That records identifying the subject will be kept confidential and, to the extent permitted by the applicable laws and/or regulations, will not be made publicly available. If the results of the trial are published, the subject's identity will remain confidential.

Samples of your blood are stored in a secure central storage site (not the clinic). Your name is not on the samples. The label on each sample tube contains only 4 pieces of information: a participant ID number, the substance in the tube, a visit number, and a visit date.

The results of tests for immune response, including genetic tests, are confidential. They do not identify you by name. They are not part of your medical records.

We cannot guarantee absolute privacy. Information about you may be released if required by law.

Site: If this study is being done at a US site where a Certificate of Confidentiality does apply, include the following 3 paragraphs verbatim. Bulleted examples should include all appropriate cases (reportable communicable disease, risk of harm to self or others, etc.)

To help us protect your privacy, the US government has given us a Certificate of Confidentiality.[19] The certificate means that the researchers cannot be forced to tell people who are not connected with this study that you are in it. If you would like to read the certificate, ask the clinic staff. We will use the certificate to refuse to give information that may identify you, even in court proceedings.

Sometimes the certificate cannot be used. For example, if someone from the US government wants to review projects that the government pays for, we cannot withhold information. We also must cooperate to meet the requirements of the US Food and Drug Administration (FDA).

Sometimes we may have to release information about you without your permission. For example, we may do this if:

- you have a disease that we must report to the health department, such as certain sexually transmitted infections
- we suspect that you may be harming yourself or others or planning to do so

14. What if my participation in the study makes me sick or injures me?[20]

If you get sick or injured, tell the clinic staff immediately. The clinic staff will treat you for study-related problems or tell you where to get the treatment you need.

[19] **http://grants.nih.gov/grants/policy/coc/background.htm** Under section 301(d) of the Public Health Service Act (42 U.S.C. 241(d)) the Secretary of Health and Human Services may authorize persons engaged in biomedical, behavioral, clinical, or other research to protect the privacy of individuals who are the subjects of that research. This authority has been delegated to the National Institutes of Health (NIH). Persons authorized by the NIH to protect the privacy of research subjects may not be compelled in any Federal, State, or local civil, criminal, administrative, legislative, or other proceedings to identify them by name or other identifying characteristic.

[20] **21 CFR 50.25.a.6** For research involving more than minimal risk, an explanation as to whether any compensation and an explanation as to whether any medical treatments are available if injury occurs and, if so, what they consist of, or where further information may be obtained. **ICH 4.8.10.j** The compensation and/or treatment available to the subject in the event of trial-related injury.

The cost of treatment of problems related to receiving vaccine (or placebo) will be covered by Wyeth Vaccines Research (the vaccine developer) or by the clinical trial site from the funds that support this clinical trial.

No funds are available from the clinical trial sites, the US NIH, or the HVTN to provide compensation for nonphysical injury such as lost work or pain and suffering. You and/or your health insurance carrier will continue to be responsible for medical costs incurred outside this study or for medical expenses determined not directly related to study procedures or agents. You will not be giving up any of your legal rights by signing this consent form.

15. What if the researchers learn new information during this study?

Results of this study or other scientific research may affect your willingness to continue to participate in this study.[21] If we learn new information of this kind, we will share it with you.

16. Will I have to pay?

You do not have to pay for the study products, research clinic visits, examinations, or laboratory tests that are part of this study.[22]

17. Will I be paid?

Site: Explain what is paid for. Example:

You will receive $[#] for each visit you complete, to cover the cost of [Insert text].[23]

18. Who should I call if I have questions or problems?[24]

If you have questions about this study, contact
[name and telephone number of the investigator or other study staff].

If you have any symptoms that you think may be related to this study, contact
[name and telephone number of the investigator or other study staff].

If you have questions about your rights as a research participant, or problems or concerns about how you are being treated in this study,

[21] **21 CFR 50.25.b.5** A statement that significant new findings developed during the course of the research which may relate to the subject's willingness to continue participation will be provided to the subject. **ICH 4.8.10.p** That the subject or the subject's legally acceptable representative will be informed in a timely manner if information becomes available that may be relevant to the subject's willingness to continue participation in the trial.

[22] **21 CFR 50.25.b.3** Any additional costs to the subject that may result from participation in the research. **ICH 4.8.10.l** The anticipated expenses, if any, to the subject for participating in the trial.

[23] **ICH 4.8.10.k** The anticipated prorated payment, if any, to the subject for participating in the trial.

[24] **21 CFR 50.25.a.7** An explanation of whom to contact for answers to pertinent questions about the research and research subjects' rights, and whom to contact in the event of a research-related injury to the subject. **ICH 4.8.10.q** The person(s) to contact for further information regarding the trial and the rights of trial subjects, and whom to contact in the event of trial-related injury.

contact
[name/title/phone of person on IRB or other appropriate organization].

If you want to leave this study, contact
[name and telephone number of the investigator or other study staff].

If you have read this consent form (or had it explained to you), understand it, and agree to take part in this study, please sign your name below.

Participant's name (print)	Participant's signature and date	Time (if signed on date of enrollment)
Study staff conducting consent discussion (print)	Study staff signature and date	Time (if signed on date of enrollment)

Site: For participants unable to read or write, substitute the signature block below:

Participant's name (print)	Participant's mark	Date	Time (if signed on date of enrollment)
Study staff conducting consent discussion (print)	Study staff signature and date		Time (if signed on date of enrollment)
Witness's name (print)	Witness's signature and date		Time (if signed on date of enrollment)

Appendix B: Sample informed consent form (Part B)

Title: A Phase I clinical trial to evaluate the safety and immunogenicity of HIV-1 *gag* DNA vaccine alone or with *IL-15* DNA, boosted with HIV-1 *gag* DNA + *IL-15* DNA, or HIV-1 *gag* DNA + *IL-12* DNA, in healthy, HIV-1 uninfected adult participants

Short title: A safety study of HIV-1 *gag* DNA vaccine and *IL-15* DNA adjuvant with 2 different booster injections

Thank you for your interest in this study.

The HIV Vaccine Trials Network (HVTN) and [site] are conducting a research study (an experiment).[1]

Site: Footnotes connecting template language to CFR and ICH guidelines are intended for writers, reviewers, and IRBs/IECs. Delete before giving consent form to participant unless your site favors inclusion

This study has 2 parts (A and B). You are being asked to join Part B, which is testing two experimental vaccines against HIV, the virus that causes AIDS. Vaccines are given to prevent infection or fight disease. Part B of this study is also testing four experimental adjuvants. An adjuvant is a substance that helps the body respond to a vaccine. Part A tests one experimental vaccine and one experimental adjuvant, which are also tested in Part B. People in Part B will participate only if there were no serious safety concerns seen in Part A.

We are testing the vaccines and adjuvants to see if they are safe to give to people and well tolerated. We are also testing to see how your immune system responds to them. The immune system protects your body against infections.[2]

This study is paid for by the US National Institutes of Health (NIH). The researcher in charge of this study at this clinic is
[Insert name of the site PI].

Participation in this study is voluntary. You do not have to join.[3] If you join the study and stay in it, you will be in it for about 18 months.[4] About 120 people will take part in this study ([#] at this clinic), 48 in Part A and 72 in Part B.[5]

This is an *informed consent* form. It answers these questions:

1. What is being tested?
2. How do I join this study?

[1] **21 CFR 50.25.a.1** A statement that this study involves research… **ICH 4.8.10.a** That the trial involves research.

[2] **21 CFR 50.25.a.1** …an explanation of the purposes of the research… **ICH 4.8.10.b** The purpose of the trial.

[3] **21 CFR 50.25.a.8** A statement that participation is voluntary…. **ICH 4.8.10.m** That the subject's participation in the trial is voluntary….

[4] **21 CFR 50.25.a.1** …and the expected duration of the subject's participation… **ICH 4.8.10.s** The expected duration of the subject's participation in the trial.

[5] **21 CFR 50.25.b.6** The approximate number of subjects involved in this study. **ICH 4.8.10.t** The approximate number of subjects involved in the trial.

3. What will happen during clinic visits?
4. What will happen to my blood samples?
5. What are the risks and inconveniences?
6. What are the benefits?
7. What are the alternatives to participating?
8. What are my responsibilities?
9. Can the researchers stop injections or take me out of this study?
10. What if I get HIV during this study?
11. What if I choose to leave this study?
12. Who makes sure this study is done correctly?
13. How will my private information be protected?
14. What if the experimental vaccine injures me or makes me sick?
15. What if the researchers learn new information during this study?
16. Will I have to pay?
17. Will I be paid?
18. Who should I call if I have questions or problems?

Read this consent form carefully. Please ask questions about anything you do not understand. The clinic staff will talk with you about the information in this form, and test your understanding. We encourage you to ask questions about this study at any time.

Site: Add the following paragraph (or one like it) if appropriate:

You may want to talk to others (such as family, friends, or your doctor) before you decide whether to join this study.

We will ask you to sign this form. Signing means:

- you have read the form (or had it explained to you),
- you understand it, and
- you agree to join this study.

We will give you a copy of this form.

1. What is being tested?[6]

The vaccine is called HIV-1 *gag* DNA vaccine, which will be called gag DNA vaccine from now on. It was developed by Wyeth Vaccines Research.

The 2 adjuvants are called *IL-15* DNA and *IL-12* DNA.

The vaccines and adjuvants in this study are made in a laboratory. The vaccines are not made from live HIV or from HIV-infected cells. They do not contain live or killed HIV. ***It is impossible to get HIV infection or AIDS from these experimental vaccines.***

All the vaccines and adjuvants are experimental. They have not been approved for treating or preventing HIV infection. The US Food and Drug Administration (FDA) allows their use in research only.

[6] **ICH 4.8.10.c** The trial treatment(s) and the probability for random assignment to each treatment.

gag *DNA vaccine*

This experimental vaccine contains a piece of DNA made in the laboratory. DNA is a natural substance in the body that instructs the body to make proteins. Proteins are natural substances that the body uses to build and maintain itself as well as protect itself against disease. The DNA tells the body to make only part of a protein called Gag that is found in HIV. Your body's immune system may respond to this protein by making cells that recognize and fight against this type of HIV protein.

IL-15 *DNA adjuvant*

The *IL-15* DNA adjuvant is DNA that will tell your body to make IL-15, a normal protein in the body. IL-15 can help the immune system keep a "memory" of how to fight certain germs, so it will be ready to fight those germs again later.

IL-12 *DNA adjuvant*

The *IL-12* DNA adjuvant is DNA that will tell your body to make IL-12, a normal protein in the body that helps immune cells work together.

Placebo

Not everyone in this study will get an experimental vaccine. Some people will get a placebo, an inactive substance that does not contain vaccine. In this study, the placebo is sterile salt water. We give placebo to some people, and compare the results from the people who got the experimental vaccines with the results from people who got the placebo. This helps us measure the effects of the experimental vaccines.

Being assigned to a group

You will be assigned to one of 2 groups and to an experimental vaccine or placebo at random, like the toss of a coin. You have equal chances to be in any of these 2 groups. You will not know which group you are in. You have an 83% (5 in 6) chance of getting an experimental vaccine. You have a 17% (1 in 6) chance of getting placebo. This is a double-blind study. That means that neither you nor the researchers at your clinic know which product (experimental vaccine or placebo) you are getting until after the study is over.

Part B will test the *gag* DNA vaccine with the *IL-15* DNA adjuvant.

Part B will also test different kinds of booster vaccines. Booster vaccines can be the same or different products that scientists think will improve the body's response to the *gag* DNA vaccine with *IL-15* DNA adjuvant. The 2 booster vaccines are:

- *gag* DNA vaccine with *IL-15* DNA adjuvant
- *gag* DNA vaccine with *IL-12* DNA adjuvant

We will review the results of current studies with the *gag* DNA vaccine and *IL-12* DNA adjuvant to see that there are no serious safety concerns before they are tested on people in this study.

In each of the 2 groups of Part B, 30 people will receive the experimental vaccine and 6 people will receive the placebo. All people in Part B will be scheduled to receive an injection (shot) at 5 separate visits.

In Group 5, 30 people will receive 5 shots of the *gag* DNA vaccine with a high dose of the *IL-15* DNA adjuvant and 6 people will receive 5 shots of placebo.

There is no longer a Group 6 in the trial, because Wyeth and the HVTN have changed plans.

In Group 7, 30 people will receive 3 shots of *gag* DNA vaccine with a high dose of the *IL-15* DNA adjuvant and then they will get 2 shots of *gag* DNA vaccine with *IL-12* DNA adjuvant. 6 people will receive 5 shots of placebo.

The following table shows the groups and the study products they get.

	Number of people	First injection	Number of months after first injection			
			1	3	6	9
Group 5	30	*gag* DNA + high dose *IL-15* DNA	*gag* DNA + high dose *IL-15* DNA	*gag* DNA + high dose *IL-15* DNA	*gag* DNA + high dose *IL-15* DNA	*gag* DNA + high dose *IL-15* DNA
	6	placebo	placebo	placebo	placebo	placebo
Group 7	30	*gag* DNA + high dose *IL-15* DNA	*gag* DNA + high dose *IL-15* DNA	*gag* DNA + high dose *IL-15* DNA	*gag* DNA + *IL-12* DNA	*gag* DNA + *IL-12* DNA
	6	placebo	placebo	placebo	placebo	placebo

2. How do I join this study?[7]

To see if you can take part in this study, you will have some screening procedures. Screening will include:

- questions about your medical history
- personal questions about your sexual behavior and any drug use
- physical exam

Site: In the following item, revise units of measure as appropriate

- blood tests to check for diseases such as HIV, syphilis, and hepatitis, and to check your general health; about 50 mL, or 3 tablespoons, of your blood will be drawn
- urine sample
- pregnancy test (for women)

[7] **21 CFR 50.25.a.1** …a description of the procedures to be followed… **ICH 4.8.10.d** The trial procedures to be followed, including all invasive procedures.

You may have already signed a consent form for screening. If not, you will need to sign this consent form before we can do the screening.

Information from the screening can only be used for 56 days (8 weeks). If you are not enrolled into the study by then, you may need to be screened again.

The results of the screening tests may show that you cannot join this study. We will explain the results to you, and tell you about places where you can get support and medical care if you need it.

If you are pregnant or breastfeeding, you cannot join this study.

If you have allergies to bupivacaine (Marcaine) or other local anesthetics such as lidocaine (Xylocaine), mepivicaine (Polocaine/Carbocaine), etidocaine (Duranest), or prilocaine, you cannot join this study.

If you are HIV positive, you cannot join this study. The clinic staff will counsel you about your HIV infection and about telling your partner(s). The clinic staff will tell you about places where you can get support and medical care, and about other studies you may want to join.

3. What will happen during clinic visits?[7]

Site: Give number of visits and range of visit lengths for Part B only

You will visit the clinic about [#] times. The length of visits will vary from [#] to [#] hours.

If necessary, we may ask you to return to the clinic for more visits and/or lab tests.

You will be tested for HIV regularly. You will be counseled about the test and your results. You will also get regular counseling on how to reduce your risk of getting HIV.

At some visits, we will ask you questions to see if you have experienced personal problems or discrimination because of being in an HIV vaccine study. You can tell us about these problems at any time. We will also ask you personal questions about your sexual behavior and any drug use.

The following table shows what will happen at each study visit.

Procedure	Screening visit	1st injection visit	Time after 1st injection visit (in months)											
			½	1	1½	3	3½	6	6½	9	9½	12	15	18
Injection		√		√		√		√		√				
Medical history	√													
Complete physical	√													√
Brief physical		√	√	√	√	√	√	√	√	√	√	√	√	
Urine test	√		√		√		√		√		√			
Blood drawn	√	√	√		√		√		√		√	√	√	√
Pregnancy test (women)	√	√		√		√		√		√		√		
HIV testing/counseling	√						√		√		√	√	√	√
Interview/questionnaire	√	√	√	√	√	√	√	√	√	√	√	√	√	√

Not shown in this table is a time after all study participants have completed their last scheduled visit when you can find out what products you received.

At each visit, we will check for any changes in your health. We will ask you how you are feeling, and if you are taking any medication. At some visits we will examine you, and ask you to give urine or blood samples. We will *not* test your blood or urine for illegal drugs.

After all participants have had their final clinic visit (or sooner if necessary), we will tell you whether you got the experimental vaccine or placebo. To do so, we may ask you to come to the clinic one more time. Not all participants join this study at the same time, so you may have to wait as much as a year after your final clinic visit to learn what you got.

Injections

You will get an injection at 5 of your clinic visits. If you are a woman, you will have a pregnancy test before each injection. The injections will be given in the muscle of your upper arm by syringe and needle. Usually the injections are given in the same arm.

After each injection, you will stay in the clinic for at least 25 to 45 minutes. Clinic staff will watch you for possible reactions to the injection.

You will be asked to record your temperature and other side effects on a symptom log the evening of the injection and for the following 3 days, or longer if necessary. You will be asked to contact the clinic daily to report these symptoms. The clinic staff may ask you to return to the clinic if necessary. It is very important to stay in touch with the clinic staff.

If you have serious reactions, we may decide that you should not get any more injections. If that happens, we will ask you to return for other visits and tests, to check your health and to look for an immune response to any injections you got earlier.

Blood samples

Site: In this subsection, revise units of measure as appropriate

At some visits, we will take samples of your blood. The amount will depend on the lab tests we need to do. It will be some amount between 15 mL and 300 mL (1 tablespoon to about 1.25 cups). The total amount of blood taken from you during this study will be no more than 1750 mL (about 7.5 cups). To compare, people who donate blood can give about 500 mL (about 2 cups) every 8 weeks.

4. What will happen to my blood samples?

Use in this study

We will use some of your blood for safety testing, to check your health and see if you have side effects. We will tell you the results of lab tests at your next visit, or sooner if necessary.

We will use some of your blood to test your immune response to the experimental vaccine or placebo. We may also test your immune response to other vaccines you may have received, or infectious agents, such as viruses or bacteria, that you may have been exposed to in the past.

In addition, a genetic test called *HLA typing* may be performed. HLA stands for human leukocyte antigen—a tiny marker on your cells that helps protect the body from infections. You inherited your HLA type from your father and mother. We think that people with different HLA types may respond differently to the experimental vaccine. If HLA typing is done on your blood, this will not affect your participation in this study.

Tests of immune response are for HIV-related or vaccine-related research only (not to check your health), so we will not tell you or the clinic the results.

Site: Per HVTN policy, the following passage must be retained verbatim:

Storage and future testing

We will store other samples of your blood for future research that is not a part of this study. This may include genetic testing other than HLA typing. Your samples would be used for HIV-related or vaccine-related research only. An Institutional Review Board or Independent Ethics Committee, which watches over the safety and rights of research participants, must approve any research studies using your samples. Your samples will not be sold.

Your samples may contribute to a new invention or discovery. There is no plan for you to share in any money or other benefits resulting from this invention or discovery.

Your samples will be stored indefinitely. You cannot be in this study if you do not wish to have your samples stored for future research.

The researchers do not plan to contact you or your health care provider with results from future studies using your blood. This is because the procedures in research are often experimental. If the researchers decide that a specific test result would provide important information for your health, we will try to contact you. If you want this information, tell the clinic staff. Always let the study clinic know if you change your address and/or phone number.

5. What are the risks and inconveniences?[8]

Being in this study may harm you. It also keeps you from doing some things. You may find the restrictions inconvenient.

This section describes the risks and inconveniences we know about. There may be unknown risks, even serious ones. These unknown risks could affect you, or your fetus if you become pregnant. If we learn about new risks during this study, we will tell you.[9]

Risks of injections

Injections can cause pain, soreness, redness, and swelling on the part of your body where you got the injection. On rare occasions, they may cause bacterial infection at the part of your body where you got the injection.

Risks of vaccination

Vaccines can cause fever, chills, rash, aches and pains, muscle aches, muscle damage, nausea, headache, dizziness, fatigue, and feeling generally unwell.

We do not know if these experimental vaccines will change your response to an approved HIV vaccine if you receive one in the future. Currently, there is no approved and licensed HIV vaccine. If such a vaccine becomes available in the future, we do not know whether getting the experimental vaccines in this study will cause your body to respond differently to a licensed vaccine, changing your body's ability to prevent HIV infection and disease. Your body's ability to prevent HIV infection and AIDS may become better or worse, or stay the same.

Allergic reaction

After receiving any of the study products, you could have an allergic reaction, like a rash, hives, or even difficulty breathing. *Allergic reactions can be life threatening.* The clinic staff will watch you for at least 25 to 45 minutes after each injection (the time when most allergic reactions occur)

[8] **21 CFR 50.25.a.2** A description of any reasonably foreseeable risks or discomforts to the subject. **21 CFR 50.25.b.1** A statement that the particular treatment or procedure may involve risks to the subject (or to the embryo or fetus, if the subject is or may become pregnant) which are currently unforeseeable. **ICH 4.8.10.g** The reasonably foreseeable risks or inconveniences to the subject and, when applicable, to an embryo, fetus, or nursing infant.

[9] **ICH 4.8.10.p** That the subject or the subject's legally acceptable representative will be informed in a timely manner if information becomes available that may be relevant to the subject's willingness to continue participation in the trial. **21 CFR 50.25.b.5** A statement that significant new findings developed during the course of the research which may relate to the subject's willingness to continue participation will be provided to the subject.

and give you treatment if you need it. People with a known allergy to bupivacaine (Marcaine) or related local anesthetics cannot participate in this study.

General risks of DNA vaccines

Possible risks related to DNA vaccines include: muscle damage at the site of the injection, the production of antibodies which might react with normal body tissues and cause an autoimmune disease, and insertion of the vaccine DNA into the body's DNA. This could lead to cancer or unknown side effects. Although these risks are possible, only muscle damage at the site of injection has been seen so far in animals or people. Other experimental DNA vaccines have also been given to thousands of people since 1995. These vaccines have not caused serious side effects, although we do not yet have long-term safety information about the people in these studies.

Risks of gag *DNA vaccine*

The *gag* DNA vaccine in this study has been given to 40 people in Part A of this study, with no serious safety concerns so far. However, DNA *gag* vaccines similar to this one have been tested in several hundred people with no known serious side effects. The *gag* DNA vaccine in this study was tested in mice and rabbits with no serious side effects. However, animal tests may not show what will happen in people. The *gag* DNA vaccine is being tested in people in the HVTN 060 study , and those results will be reviewed to make sure that there are no serious safety concerns before they are given in this study.

Risks of IL-15 *DNA adjuvant*

The *IL-15* DNA adjuvant has been given to 30 people in Part A of this study with no serious safety concerns so far. A similar *IL-15* DNA adjuvant has been tested in animals with no serious side effects. A few rabbits developed a purple area at the injection site for a few days, which was not serious.

Your body makes IL-15 protein naturally. This *IL-15* DNA adjuvant will cause your body to make a little bit more of this protein. High amounts of IL-15 protein are seen in some people with autoimmune diseases (diseases where the body attacks its own cells). The IL-15 protein alone may not cause autoimmune disease, but it may play a part in getting such a disease. In theory, having your body make more IL-15 protein than it usually does may put you at greater risk for autoimmune disease. We think the risk of this happening is low, because of the small amount of extra IL-15 protein your body would make after getting the adjuvant.

Risks of IL-12 *DNA adjuvant*

The *IL-12* DNA adjuvant in this study has been given to 40 people in another study without any serious safety concerns so far. A similar *IL-12*

DNA adjuvant has been tested in animals with no serious side effects. However, animal tests may not show what will happen in people.

IL-12, in the form of a protein known as recombinant human IL-12, has been tested in many studies with both healthy people and patients with infectious diseases. In healthy people, the most frequently reported side effects included fever, headache, muscle aches, nausea, chills, weakness, fatigue, and reactions at the injection site. Some patients had changes in test results for blood sugar, liver activity, and white blood cell count (cells that fight infection). These changes were temporary and test results returned to normal without treatment. On rare occasions, after receiving high doses of recombinant human IL-12, people have reported anxiety, confusion, depression, gastrointestinal bleeding, kidney problems, an abnormal feeling of burning or tingling, and increased blood pressure. The recombinant human IL-12 protein is not the same as the *IL-12* DNA adjuvant used in this study. *IL-12* DNA leads to the production of a very limited amount of IL-12 protein in the body.

Risks of bupivacaine

The *gag* DNA vaccine, the *IL-15* DNA adjuvant, and the *IL-12* DNA adjuvant contain bupivacaine. Bupivacaine helps the DNA get into the muscle. It is an anesthetic, similar to the numbing medicine used by dentists. Bupivacaine, like all medicines, can have certain side effects. When bupivacaine was given to people at a strength 50 times higher than what we will use in this study, some people had serious health problems. The side effects were rare, but included nervous system or heart problems or even death due to high levels of bupivacaine in the blood. This may happen as a result of accidental injection into the bloodstream, overdose, or slow breakdown of the drug. Nervous system side effects can include confusion, anxiety, dizziness, blurred vision, shaking, or seizures. Heart side effects can include decreased heart pumping, fast heart rate, low blood pressure, or abnormal heartbeats. Some less serious side effects include nausea, vomiting, or chills. Study nurses will be careful to avoid injecting bupivacaine into the bloodstream, but even with their best efforts it could possibly happen. Because you will be getting a much lower dose of bupivacaine if you get the *gag* DNA vaccine, the *IL-15* DNA adjuvant or the *IL-12* DNA adjuvant, and it is not likely that you will get an injection directly into your bloodstream, we think the chances of your experiencing these side effects are low.

Risks of gag *DNA and* IL-15 *DNA adjuvant given together*

Since this combination of *gag* DNA vaccine and *IL-15* DNA has been given for the first time to 40 people in Part A of this study so far, all possible risks or side effects are currently not known. Animals have had the vaccine and adjuvant together in doses similar to or larger than those planned to be given in this study without serious problems. However, animal tests may not show what will happen in people.

Risks of gag *DNA and* IL-12 *DNA adjuvant given together*

Since this combination of *gag* DNA vaccine and *IL-12* DNA has been given for the first time to 40 people so far, all possible risks or side effects are currently not known. The *gag* DNA vaccine and *IL-12* DNA are being tested in people in the HVTN 060 study before this study opens, and those results will be reviewed to make sure that there are no serious safety concerns before they are given in this study.

In this study, it is the first time that *gag* DNA and *IL-12* DNA adjuvant will be given as a booster vaccination to people who also received *gag* DNA and *IL-15* DNA adjuvant. This combination has not been studied in animals before, so all the risks are not known. This study will test if these products are safe when given together.

Blood drawing

Drawing blood may cause pain and bruising. On rare occasions, it may cause bacterial infection at the part of your body where the blood is taken. Sometimes, drawing blood causes people to feel lightheaded or to faint. Some people, especially women, may become anemic (have a low red blood cell count).

Personal problems

Some participants in other HIV vaccine studies have reported experiencing personal problems because of their participation. Spouses, other family members, or sexual partners have sometimes reacted by:

- becoming angry when a participant joined a study without consulting them
- worrying that the test vaccine would be harmful
- assuming that the participant was infected with HIV and shunning them
- assuming that the participant is engaging in certain sexual activities or drug use, and treating them unfairly

On rare occasions, a participant has reported losing a job because of being in an HIV vaccine study. This was either because the study took too much time away from work, or because the employer thought the participant was HIV infected or at high risk for HIV.

If genetic testing (such as HLA typing) is performed on your blood, there is a very small chance that the results will cause personal problems. Information from this test may suggest you are at risk for certain diseases. This does not mean you will get a disease, but if your test results were known, you could have trouble getting insurance or a job. This risk is extremely small, because the test results do not identify you by name. They do not become part of your medical records.

To help avoid these problems, talk with the study staff if you have to get HIV testing done outside this study. You can get an ID card that shows you joined the study. The card also lists a toll-free number you can call for help or information.

Clinic staff will help you with personal problems you may experience because of being in this study.

HIV exposure

If you are exposed to HIV at some time after getting an experimental vaccine, we do not know what will happen. The experimental vaccine could increase or decrease, or have no effect on:

- your risk of becoming infected with HIV if exposed
- the time it takes to develop AIDS after being infected
- the course of HIV infection

We do not know if getting the experimental vaccine will protect you from HIV. This study will not answer that question. In the past, some people have become infected with HIV even though they got an experimental vaccine. The experimental vaccine did not cause the HIV infection, but did not prevent infection in these cases. Because we do not know the effect of the experimental vaccine, *we ask you not to do anything that may expose you to HIV, like having unprotected sex or sharing needles or injection equipment.*

False positive HIV test

Standard HIV tests look for antibodies (made by cells in your immune system) that recognize HIV. The experimental vaccine may cause your body to produce these antibodies. In this case, the standard HIV test could show a positive result. This does not mean you are infected with HIV—the test result could be a *false positive*.

If this happens, we will do further tests to confirm that you are not infected with HIV. If the experimental vaccine caused the false positive result, we do not know how long the HIV test will stay positive. We will offer retesting free of charge as long as the positive HIV test is due to the experimental vaccine.

If you are tested for HIV outside this study, a false positive result may cause you trouble. You may have trouble with:

- insurance
- medical/dental care
- travel to other countries
- employment
- military service

Blood banks and medical institutions know that an experimental vaccine may give a false positive result. Still, if you continue to have a false positive result after the study you will have trouble donating blood, body fluids, body tissues, or organs. You may even be permanently banned from donating.

To help with these situations, or to prevent discrimination, we can talk to insurance companies, employers, and others to explain that you are in a study. We would do this only at your request and with your written permission. You can also get an ID card that shows you joined the study. The card also lists a toll-free number you can call for help or information.

Restrictions

While you are in this study, there are things you cannot do.

- Because of the risk of a false positive HIV test, you should get your HIV testing done only at the clinic. If you have to be tested for HIV outside this study, please talk to the clinic staff.
- You must not donate blood, body tissues, body fluids, or organs.
- You may not be able to join other medical research studies. If you are thinking about joining another study, please talk to the clinic staff.
- After you are finished with this study, you may not be able to join other HIV vaccine studies.

We ask you not to do anything that may expose you to HIV, like having unprotected sex or sharing needles or injection equipment.

Pregnancy risks

Women should not become pregnant during this study (about 18 months), because we do not know how the experimental vaccines or adjuvants could affect the fetus. If you are a woman having sex that could lead to pregnancy, you must agree to use effective birth control starting at least 21 days prior to enrollment and continuing until after your last clinic visit. This means using any of the following methods:

- birth control drugs that prevent pregnancy—given by pills, shots, patches, vaginal rings, or inserts under the skin
- male or female condoms, with or without a cream or gel that kills sperm
- diaphragm or cervical cap with a cream or gel that kills sperm
- intrauterine device (IUD)
- any other contraceptive method approved by the researchers

6. What are the benefits?

This study may not benefit you personally. Being in this study may help in the search for an HIV vaccine.[10]

Site: Add information about other benefits (health care, tests, etc.) as appropriate

7. What are the alternatives to participating?

You may choose not to join this study. Other services you receive at this institution will not be affected.[11]

If you choose not to join this study, you may join a different experimental HIV vaccine study, if one is available and you are eligible.[12]

8. What are my responsibilities?[13]

If you join this study, you will be asked to:

- come to all clinic visits
- record your temperature and other side effects on the symptom log , and report these results to the clinic staff, the evening of the injection and for the following 3 days, or longer if necessary
- tell clinic staff about any symptoms or side effects you have
- tell clinic staff about any medications you are taking
- tell clinic staff before getting any other vaccines, such as a flu shot
- follow instructions from the clinic staff
- stay in touch with the clinic staff; tell them if you have moved or if you want to leave the study.
- get your HIV testing done only at the clinic
- women: avoid pregnancy until after your last clinic visit

The clinic staff will share the HVTN Participant's Bill of Rights and Responsibilities with you. That document tells more about your rights and responsibilities.

9. Can the researchers stop injections or take me out of this study?

Your injections may be stopped if:

[10] **21 CFR 50.25.a.3** A description of any benefits to the subject or to others which may reasonably be expected from the research. **ICH 4.8.10.h** The reasonably expected benefits. When there is no intended clinical benefit to the subject, the subject should be made aware of this.

[11] **21 CFR 50.25.a.8** …that refusal to participate will involve no penalty or loss of benefits to which the subject is otherwise entitled…. **ICH 4.8.10.m** …that the subject may refuse to participate…without penalty or loss of benefits to which the subject is otherwise entitled.

[12] **21 CFR 50.25.a.4** A disclosure of appropriate alternative procedures or courses of treatment, if any, that might be advantageous to the subject. **ICH 4.8.10.i** The alternative procedure(s) or course(s) of treatment that may be available to the subject, and their important potential benefits and risks.

[13] **ICH 4.8.10.e** The subject's responsibilities.

- getting injections would be harmful to you
- you become pregnant
- you need a treatment, and the treatment and the experimental vaccine might interfere with each other
- the experimental vaccine or placebo is no longer available

If you must stop getting injections before this study is over, we will ask you to come back to the clinic to check your health and your immune response.

You may be taken out of this study entirely if:

- you cannot or do not attend the study visits
- you do not follow instructions
- you get infected with HIV
- the study is canceled.[14]

10. What if I get HIV during this study?

If you get infected with HIV during this study, the clinic staff will do additional HIV testing to confirm the infection and learn more about it. You will not be able to stay in this study.

Site: Include (as needed) required reporting information, and modify (as appropriate) information about provision of treatment

The clinic staff will counsel you about your HIV infection and about telling your partner(s). Medical care and treatment for HIV infection are not a part of this study. The clinic staff will tell you about places where you can get support and medical care, and about other studies you may want to join.

11. What if I choose to leave this study?[15]

If you join this study, you can leave it at any time. If you leave this study, you will not lose any benefits or rights you would normally have or be disadvantaged in any way.[16]

If you decide to leave this study, please tell the clinic staff. We will ask you to come back to the clinic at least once to check your health and look for an immune response to study injections.

[14] **21 CFR 50.25.b.2** Anticipated circumstances under which the subject's participation may be terminated by the investigator without regard to the subject's consent. **ICH 4.8.10.r** The foreseeable circumstances and/or reasons under which the subject's participation in the trial may be terminated.

[15] **21 CFR 50.25.b.4** The consequences of a subject's decision to withdraw from the research and procedures for orderly termination of participation by the subject.

[16] **21 CFR 50.25.a.8** …that the subject may discontinue participation at any time without penalty or loss of benefits to which the subject is otherwise entitled. **ICH 4.8.10.m** …the subject may…withdraw from the trial, at any time, without penalty or loss of benefits to which the subject is otherwise entitled.

Like everyone else in this study, you will have to wait until all participants complete their final study visit to find out whether you got the experimental vaccine or the placebo.

12. Who makes sure this study is done correctly?

Several groups watch over this study to see that your rights are protected and that the researchers are following this study plan.[17] These groups include:

Site: Modify list for non-US monitors and IBC as appropriate

- the US National Institutes of Health (NIH)
- the US Food and Drug Administration (FDA)
- a local Institutional Review Board or Independent Ethics Committee
- Wyeth Vaccines Research and people who work for them
- the HIV Vaccine Trials Network and people who work for them
- [Insert name of local regulatory authority as appropriate]

A local Community Advisory Board is also involved in this study. Community Advisory Boards assist scientists in developing research studies and review these studies for issues important to the community. The Community Advisory Board will not have access to medical information that can identify you.

13. How will my private information be protected?[18]

US sites: Check HIPAA authorization for conflicts with this section

We will do our best to protect your private information. Your records are kept in locked files at the clinic. On most records, we use a participant ID number, not your name.

The results of this study may be published. Any publication will not use your name or identify you personally.

Most of the groups who watch over this study may review your medical records. Your records may also be reviewed by clinic staff, and by the vaccine developer or people who work for them. Reviewers will keep your records private.

[17] **ICH 4.8.10.n** That the monitor(s), the auditor(s), the IRB/IEC, and the regulatory authority(ies) will be granted direct access to the subject's original medical records for verification of clinical trial procedures and/or data, without violating the confidentiality of the subject, to the extent permitted by the applicable laws and regulations and that, by signing a written informed consent form, the subject or the subject's legally acceptable representative is authorizing such access.

[18] **21 CFR 50.25.a.5** A statement describing the extent, if any, to which confidentiality of records identifying the subject will be maintained and that notes the possibility that the Food and Drug Administration may inspect the records. **ICH 4.8.10.o** That records identifying the subject will be kept confidential and, to the extent permitted by the applicable laws and/or regulations, will not be made publicly available. If the results of the trial are published, the subject's identity will remain confidential.

Samples of your blood are stored in a secure central storage site (not the clinic). Your name is not on the samples. The label on each sample tube contains only 4 pieces of information: a participant ID number, the substance in the tube, a visit number, and a visit date.

The results of tests for immune response, including genetic tests, are confidential. They do not identify you by name. They are not part of your medical records.

We cannot guarantee absolute privacy. Information about you may be released if required by law.

Site: If this study is being done at a US site where a Certificate of Confidentiality does apply, include the following 3 paragraphs verbatim. Bulleted examples should include all appropriate cases (reportable communicable disease, risk of harm to self or others, etc.)

To help us protect your privacy, the US government has given us a Certificate of Confidentiality.[19] The certificate means that the researchers cannot be forced to tell people who are not connected with this study that you are in it. If you would like to read the certificate, ask the clinic staff. We will use the certificate to refuse to give information that may identify you, even in court proceedings.

Sometimes the certificate cannot be used. For example, if someone from the US government wants to review projects that the government pays for, we cannot withhold information. We also must cooperate to meet the requirements of the US Food and Drug Administration (FDA).

Sometimes we may have to release information about you without your permission. For example, we may do this if:

- you have a disease that we must report to the health department, such as certain sexually transmitted infections
- we suspect that you may be harming yourself or others or planning to do so

14. What if my participation in the study makes me sick or injures me?[20]

If you get sick or injured, tell the clinic staff immediately. The clinic staff will treat you for study-related problems or tell you where to get the treatment you need.

[19] **http://grants.nih.gov/grants/policy/coc/background.htm** Under section 301(d) of the Public Health Service Act (42 U.S.C. 241(d)) the Secretary of Health and Human Services may authorize persons engaged in biomedical, behavioral, clinical, or other research to protect the privacy of individuals who are the subjects of that research. This authority has been delegated to the National Institutes of Health (NIH). Persons authorized by the NIH to protect the privacy of research subjects may not be compelled in any Federal, State, or local civil, criminal, administrative, legislative, or other proceedings to identify them by name or other identifying characteristic.

[20] **21 CFR 50.25.a.6** For research involving more than minimal risk, an explanation as to whether any compensation and an explanation as to whether any medical treatments are available if injury occurs and, if so, what they consist of, or where further information may be obtained. **ICH 4.8.10.j** The compensation and/or treatment available to the subject in the event of trial-related injury.

The cost of treatment of problems related to receiving vaccine (or placebo) will be covered by Wyeth Vaccines Research (the vaccine developer) or by the clinical trial site from the funds that support this clinical trial.

No funds are available from the clinical trial sites, the US NIH, or the HVTN to provide compensation for nonphysical injury such as lost work or pain and suffering. You and/or your health insurance carrier will continue to be responsible for medical costs incurred outside this study or for medical expenses determined not directly related to study procedures or agents. You will not be giving up any of your legal rights by signing this consent form.

15. What if the researchers learn new information during this study?

Results of this study or other scientific research may affect your willingness to continue to participate in this study.[21] If we learn new information of this kind, we will share it with you.

16. Will I have to pay?

You do not have to pay for the study products, research clinic visits, examinations, or laboratory tests that are part of this study.[22]

17. Will I be paid?

Site: Explain what is paid for. Example:

You will receive $[#] for each visit you complete, to cover the cost of [Insert text].[23]

18. Who should I call if I have questions or problems?[24]

If you have questions about this study, contact
[name and telephone number of the investigator or other study staff].

If you have any symptoms that you think may be related to this study, contact
[name and telephone number of the investigator or other study staff].

If you have questions about your rights as a research participant, or problems or concerns about how you are being treated in this study,

[21] **21 CFR 50.25.b.5** A statement that significant new findings developed during the course of the research which may relate to the subject's willingness to continue participation will be provided to the subject. **ICH 4.8.10.p** That the subject or the subject's legally acceptable representative will be informed in a timely manner if information becomes available that may be relevant to the subject's willingness to continue participation in the trial.

[22] **21 CFR 50.25.b.3** Any additional costs to the subject that may result from participation in the research. **ICH 4.8.10.l** The anticipated expenses, if any, to the subject for participating in the trial.

[23] **ICH 4.8.10.k** The anticipated prorated payment, if any, to the subject for participating in the trial.

[24] **21 CFR 50.25.a.7** An explanation of whom to contact for answers to pertinent questions about the research and research subjects' rights, and whom to contact in the event of a research-related injury to the subject. **ICH 4.8.10.q** The person(s) to contact for further information regarding the trial and the rights of trial subjects, and whom to contact in the event of trial-related injury.

contact
[name/title/phone of person on IRB or other appropriate organization].

If you want to leave this study, contact
[name and telephone number of the investigator or other study staff].

If you have read this consent form (or had it explained to you), understand it, and agree to take part in this study, please sign your name below.

Participant's name (print)	Participant's signature and date	Time (if signed on date of enrollment)
Study staff conducting consent discussion (print)	Study staff signature and date	Time (if signed on date of enrollment)

Site: For participants unable to read or write, substitute the signature block below:

Participant's name (print)	Participant's mark	Date	Time (if signed on date of enrollment)
Study staff conducting consent discussion (print)	Study staff signature and date		Time (if signed on date of enrollment)
Witness's name (print)	Witness's signature and date		Time (if signed on date of enrollment)

Appendix C: Laboratory procedures for Part A

					Tube volume (mL)												
				Visit:	**1**	**2**	**3**	**4**	**5**	**6**	**7**	**8**	**9**	**10**	**11**	**12**	
				Day:	Screening visit	D0	D14	D28	D42	D84	D98	D168	D182	D273	D287	D364	
				Month:		M0	M0.5	M1	M1.5	M3	M3.5	M6	M6.5	M9	M9.5	M12	
Procedure	Ship to	Assay location	Tube			**VAC1**		**VAC2**		**VAC3**							**Total**
Blood Collection																	
Screening or diagnostic assays																	
Screening HIV test	Local Lab	Local Lab	SST		5	—	—	—	—	—	—	—	—	—	—	—	**5**
HIV diagnostic ELISA	UW-VSL/Local lab	UW-VSL/Local lab	SST		—	—	—	—	—	—	5	5	—	5	—	5	**20**
HIV RNA PCR	UW-VSL/Local lab	UW-VSL/Local lab	EDTA		—	—	—	—	—	—	5	5	—	5	—	5	**20**
HBsAg/anti-HCV/Syphilis	Local Lab	Local Lab	SST		5	—	—	—	—	—	—	—	—	—	—	—	**5**
Safety labs																	
CBC/ Diff/ platelets	Local lab	Local lab	EDTA		5	—	5	—	5	—	5	5	—	5	—	5	**35**
Chemistry panel	Local lab	Local lab	SST		5	—	5	—	5	—	5	5	—	5	—	5	**35**
T cell subsets	Local lab	Local lab	EDTA		5	—	5	—	—	—	5	—	—	—	—	5	**20**
Immunogenicity assays													—				
HLA typing	CSR	Duke	ACD		—	20	—	—	—	—	—	—	—	—	—	—	**20**
Humoral assay															—		
HIV Binding ELISA	CSR	Duke	SST		—	5	—	—	—	—	*5*	—	—	—	—	5	**15**
Ab to IL-15	CSR	Wyeth	SST		—	5	5	—	5	—	*5*	—	—	5	—	5	**30**
Cellular assays													—				
ELISpot	CSR	Duke	Na Hep		—	60		—	—	—	*60*	—	—	60	—	60	**240**
ICS	CSR	FHCRC	Na Hep		—	40	—	—	—	—	*40*	—	—	40	—	40	**160**
Tetramer	CSR	Wyeth	Na Hep		—	60	—	—	—	—	60	—	—	60	—	60	**240**
Specimen storage																	
PBMC	CSR		Na Hep		—	80	—	—	—	—	60	—	—	60	—	60	**260**
Serum	CSR		SST		—	10	—	—	—	—	10	—	—	10	—	10	**40**
**Total**					**25**	**280**	**20**	**0**	**15**	**0**	**265**	**20**	**0**	**255**	**0**	**265**	**1145**
**56-Day total**					**25**	**305**	**325**	**325**	**340**	**15**	**280**	**20**	**20**	**255**	**255**	**265**	
Urine Collection																	
Urinalysis					X	—	X	—	X	—	X	—	—	—	—	—	
Pregnancy test					X	X	—	X	—	X	—	X	—	—	—	—	

CSR = Central Specimen Repository

- HVTN Laboratory Program includes endpoint laboratories at UW-VSL, Duke, FHCRC, and SAIL-NICD. UW-VSL = University of Washington Virology Specialty Laboratory (Seattle, Washington, USA); Duke = Duke University Medical Center (Durham, North Carolina, USA); FHCRC/UW = Fred Hutchinson Cancer Research Center/University of Washington (Seattle, Washington, USA); SAIL-NICD = South African Immunology Laboratory–National Institute for Communicable Diseases (Johannesburg, South Africa)
- Non-HVTN laboratory: Wyeth
- Screening may occur over the course of several contacts/visits up to and including Day 0 prior to vaccination. Additional tests performed at screening include syphilis test, HepB and HepC serologies using serum samples.
- Diagnostic HIV Western blot will be performed at UW-VSL as indicated from samples sent for diagnostic ELISA per the HVTN algorithm for diagnosis of HIV infections.
- Local labs may perform the HIV diagnostic algorithm (following HVTN SOP) upon approval from the HVTN Laboratory Operations Division.
- For viral assays, samples are sent to UW-VSL; test to be performed if clinically indicated. Non-US sites may use local labs with pre-approval from the HVTN Laboratory Operations Division.
- Local labs may assign appropriate alternative tube types for locally performed tests.
- Chemistry panels are defined in Table 12-1 (pre-enrollment) and Table 12-2 (post-enrollment).
- Based on the number of responders observed at the primary immunogenicity timepoint (Visit 7), lab assays may be performed on all participants for humoral and cellular responses at Visits 10 and 12.
- Based on the number of participants showing anti-IL-15 antibody responses at Visits 2 and 7, this assay may be performed on specimens from Visits 3, 5, 10 and 12.

Appendix D: Laboratory procedures for Part B

					Tube volume (mL)														
				Visit:	1	2	3	4	5	6	7	8	9	10	11	12	13	14	
				Day:	Screening visit	D0	D14	D28	D42	D84	D98	D168	D182	D273	D287	D364	D455	D546	
				Month:		M0	M0.5	M1	M1.5	M3	M3.5	M6	M6.5	M9	M9.5	M12	M15	M18	
Procedure	Ship to	Assay location	Tube			**VAC1**		**VAC2**		**VAC3**		**VAC4**		**VAC5**					**Total**
Blood Collection																			
Screening or diagnostic assays																			
Screening HIV test	Local	Local	SST		5	—	—	—	—	—	—	—	—	—	—	—	—	—	**5**
HIV diagnostic ELISA	UW-VSL/Local lab	UW-VSL/Local lab	SST		—	—	—	—	—	—	5	—	5	—	5	5	5	5	**30**
HIV RNA PCR	UW-VSL/Local lab	UW-VSL/Local lab	EDTA		—	—	—	—	—	—	5	—	5	—	5	5	5	5	**30**
HBsAg/anti-HCV/Syphilis	Local	Local	SST		5	—	—	—	—	—	—	—	—	—	—	—	—	—	**5**
Safety labs																			
CBC/ Diff/ platelets	Local	Local	EDTA		5	—	5	—	5	—	5	—	5	—	5	5	5	5	**45**
Chemistry panel	Local	Local	SST		5	—	5	—	5	—	5	—	5	—	5	5	5	5	**45**
T cell subsets	Local	Local	EDTA		5	—	5	—	—	—	5	—	—	—	5	—	—	—	**20**
Immunogenicity assays																			
HLA typing	CSR	Duke	ACD		—	20	—	—	—	—	—	—	—	—	—	—	—	—	**20**
Humoral assay																			
HIV Binding ELISA	CSR	Duke	SST		—	5	—	—	—	—	5	—	5	—	*5*	—	5	5	**30**
Ab to GM-CSF	CSR	Wyeth	SST		—	5	—	—	—	—	—	—	5	—	*5*	—	5	5	**25**
Ab to IL-12	CSR	Wyeth	SST		—	5	—	—	—	—	—	—	5	—	*5*	—	5	5	**25**
Ab to IL-15	CSR	Wyeth	SST		—	5	5	—	5	—	5	—	5	—	*5*	—	5	5	**40**
Cellular assays																			
ELISpot	CSR	Duke	Na Hep		—	60		—	—	—	60	—	—	—	*60*	—	60	60	**300**
ICS	CSR	FHCRC	Na Hep		—	40	—	—	—	—	40	—	—	—	*40*	—	40	40	**200**
Tetramer	CSR	Wyeth	Na Hep		—	60	—	—	—	—	60	—	—	—	60	—	60	60	**300**
TCR repertoire	CSR	Vanderbilt	Na Hep		—	—	—	—	—	—	—	—	50	—	—	50	—	—	**100**
Specimen storage																			
PBMC	CSR		Na Hep		—	60	—	—	—	—	60	—	50	—	60	50	60	60	**400**
Serum	CSR		SST		—	10	—	—	—	—	10	—	10	—	10	10	10	10	**70**
**Total**					**25**	**270**	**20**	**0**	**15**	**0**	**265**	**0**	**150**	**0**	**275**	**130**	**270**	**270**	**1690**
**56-Day total**					**25**	**295**	**315**	**315**	**330**	**15**	**280**	**0**	**150**	**0**	**275**	**130**	**270**	**270**	
Urine Collection																			
Urinalysis					X	—	X	—	X	—	X	—	X	—	X	—	—	—	
Pregnancy test					X	X	—	X	—	X	—	X	—	X	—	X	—	—	

- CSR = Central Specimen Repository
- HVTN Laboratory Program includes endpoint laboratories at UW-VSL, Duke, FHCRC, and SAIL-NICD. UW-VSL = University of Washington Virology Specialty Laboratory (Seattle, Washington, USA); Duke = Duke University Medical Center (Durham, North Carolina, USA); FHCRC/UW = Fred Hutchinson Cancer Research Center/University of Washington (Seattle, Washington, USA); SAIL-NICD = South African Immunology Laboratory–National Institute for Communicable Diseases (Johannesburg, South Africa)
- Non-HVTN laboratory: Wyeth
- Screening may occur over the course of several contacts/visits up to and including Day 0 prior to vaccination. Additional tests performed at screening include syphilis test, HepB and HepC serologies using serum samples.
- Diagnostic HIV Western blot will be performed at UW-VSL as indicated from samples sent for diagnostic ELISA per the HVTN algorithm for diagnosis of HIV infections.
- Local labs may perform the HIV diagnostic algorithm (following HVTN SOP) upon approval from the HVTN Laboratory Operations Division.
- For viral assays, samples are sent to UW-VSL; test to be performed if clinically indicated. Non-US sites may use local labs with pre-approval from the HVTN Laboratory Operations Division.
- Local labs may assign appropriate alternative tube types for locally performed tests.
- Chemistry panels are defined in Table 12-1 (pre-enrollment) and Table 12-2 (post-enrollment).
- Based on the number of responders observed at the primary immunogenicity timepoint (Visit 11), lab assays may be performed on all participants for humoral and cellular responses at Visits 7, 8, 9, 13 and 14.
- Based on the number of participants showing anti-IL-15 antibody responses at Visit 11, this assay may be performed on specimens from Visits 3, 5, 7, 9, 13 and 14.

Appendix E: Procedures at HVTU for Part A

	Visit												
Visit:	01[a]	02	03	04	05	06	07	08	09[b]	10	11[b]	12	Post
Day:		D0	D14	D28	D42	D84	D98	D168	D182	D273	D287	D364	
Month:		M0	M.5	M1	M1.5	M3	M3.5	M6	M6.5	M9	M9.5	M12	
Procedure	Scr.	VAC1		VAC2		VAC3							
**Study procedures**													
Signed screening consent (if used)	X	—	—	—	—	—	—	—	—	—	—	—	—
Assessment of understanding	X	—	—	—	—	—	—	—	—	—	—	—	—
Signed protocol consent	X	—	—	—	—	—	—	—	—	—	—	—	—
Medical history	X	—	—	—	—	—	—	—	—	—	—	—	—
Complete physical exam	X	—	—	—	—	—	—	—	—	—	—	X	—
Abbreviated physical exam	—	X	X	X	X	X	X	X	—	X	—	—	—
Risk reduction/pregnancy prevention counseling	X	X	X	X	X	X	X	X	—	X	—	—	—
Behavioral risk assessment	X	—	—	—	—	—	—	—	—	—	—	—	—
Confirm eligibility, obtain demographics, randomize participant	X	—	—	—	—	—	—	—	—	—	—	—	—
Social impact assessment	—	—	—	—	—	X	—	X	—	—	—	X	—
Outside testing and belief questionnaire	—	—	—	—	—	—	—	X	—	—	—	X	—
Concomitant medications	X	X	X	X	X	X	X	X	—	X	—	X	—
Intercurrent illness / adverse experience	—	X	X	X	X	X	X	X	—	X	—	X	—
HIV infection assessment/results[c]	X	—	—	—	—	—	X	X	—	X	—	X	—
Autoimmune questionnaire	X	X	X	X	X	X	X	X	—	X	—	X	—
**Local lab assessment**													
Urine dipstick	X	—	X	—	X	—	X	—	—	—	—	—	—
Pregnancy (urine or serum HCG)[d]	X	X	—	X	—	X	—	X	—	—	—	—	—
T-cell subsets	X	—	X	—	—	—	X	—	—	—	—	X	—
CBC, differential, platelet[e]	X	—	X	—	X	—	X	X	—	X	—	X	—
Chemistry panel (see Table 12-1 and Table 12-2)[e]	X	—	X	—	X	—	X	X	—	X	—	X	—
SYPHILIS	X	—	—	—	—	—	—	—	—	—	—	—	—
Hepatitis B, Hepatitis C	X	—	—	—	—	—	—	—	—	—	—	—	—
**Vaccination procedures**													
Vaccination	—	X	—	X	—	X	—	—	—	—	—	—	—
Reactogenicity assessments[f]	—	X	—	X	—	X	—	—	—	—	—	—	—
**Post-study**													
Unblind participant	—	—	—	—	—	—	—	—	—	—	—	—	X

[a] Screening may occur over the course of several contacts/visits up to and including Day 0 prior to vaccination.

[b] Visits 9 and 11 not required for participants of Part A.

[c] Includes pre- and post-test counseling and follow-up contact to report results to participant.

[d] For female participants, pregnancy test must be performed at each vaccination visit prior to vaccination. Pregnancy test to determine eligibility may be performed at screening but must be performed on Day 0 prior to first vaccination. Serum pregnancy tests may be used to confirm the results of, or substitute for, a urine pregnancy test.

[e] Blood draws required at post-enrollment vaccination visits must be performed prior to administration of study agent; however, it is not necessary to have results prior to administration. Lab tests may be drawn within the 3 days prior to vaccination.

[f] Reactogenicity assessments performed daily for up to 3 days post-vaccination (see Section 14.1).

Appendix F: Procedures at HVTU for Part B

	Visit														
Visit:	**01[a]**	**02**	**03**	**04**	**05**	**06**	**07**	**08**	**09**	**10**	**11**	**12**	**13**	**14**	**Post**
Day:		D0	D14	D28	D42	D84	D98	D168	D182	D273	D287	D364	D455		
Month:		M0	M.5	M1	M1.5	M3	M3.5	M6	M6.5	M9	M9.5	M12	M15	M18	
Procedure	Scr.	**VAC1**		**VAC2**		**VAC3**		**VAC4**		**VAC5**					
**Study procedures**															
Signed screening consent (if used)	X	—	—	—	—	—	—	—	—	—	—	—	—	—	—
Assessment of understanding	X	—	—	—	—	—	—	—	—	—	—	—	—	—	—
Signed protocol consent	X	—	—	—	—	—	—	—	—	—	—	—	—	—	—
Medical history	X	—	—	—	—	—	—	—	—	—	—	—	—	—	—
Complete physical exam	X	—	—	—	—	—	—	—	—	—	—	—	—	X	—
Abbreviated physical exam	—	X	X	X	X	X	X	X	X	X	X	X	X	—	—
Risk reduction/pregnancy prevention counseling	X	X	X	X	X	X	X	X	X	X	X	X	X	—	—
Behavioral risk assessment	X	—	—	—	—	—	—	—	—	—	—	—	—	—	—
Confirm eligibility, obtain demographics, randomize participant	X	—	—	—	—	—	—	—	—	—	—	—	—	—	—
Social impact assessment	—	—	—	—	—	X	—	X	—	—	—	—		X	—
Outside testing and belief questionnaire	—	—	—	—	—	—	—	X	—	—	—	—	—	X	—
Concomitant medications	X	X	X	X	X	X	X	X	X	X	X	X	X	X	—
Intercurrent illness / adverse experience	—	X	X	X	X	X	X	X	X	X	X	X	X	X	—
HIV infection assessment/results[b]	X	—	—	—	—	—	X	—	X	—	X	X	X	X	—
Autoimmune questionnaire	X	X	X	X	X	X	X	X	X	X	X	X	X	X	—
**Local lab assessment**															
Urine dipstick	X	—	X	—	X	—	X	—	X	—	X	—	—	—	—
Pregnancy (urine or serum HCG)[c]	X	X	—	X	—	X	—	X	—	X	—	X	—	—	—
T-cell subsets	X	—	X	—	—	—	X	—	—	—	X	—	—	—	—
CBC, differential, platelet[d]	X	—	X	—	X	—	X	—	X	—	X	X	X	X	—
Chemistry panel (see Table 12-1 and Table 12-2)[d]	X	—	X	—	X	—	X	—	X	—	X	X	X	X	—
SYPHILIS	X	—	—	—	—	—	—	—	—	—	—	—	—	—	—
Hepatitis B, Hepatitis C	X	—	—	—	—	—	—	—	—	—	—	—	—	—	—
**Vaccination procedures**															
Vaccination	—	X	—	X	—	X	—	X	—	X	—	—	—	—	—
Reactogenicity assessments[e]	—	X	—	X	—	X	—	X	—	X	—	—	—	—	—
**Post-study**															
Unblind participant	—	—	—	—	—	—	—	—	—	—	—	—	—	—	X

[a] Screening may occur over the course of several contacts/visits up to and including Day 0 prior to vaccination.

[b] Includes pre- and post-test counseling and follow-up contact to report results to participant.

[c] For female participants, pregnancy test must be performed at each vaccination visit prior to vaccination. Pregnancy test to determine eligibility may be performed at screening but must be performed on Day 0 prior to first vaccination. Serum pregnancy tests may be used to confirm the results of, or substitute for, a urine pregnancy test.

[d] Blood draws required at post-enrollment vaccination visits must be performed prior to administration of study agent; however, it is not necessary to have results prior to administration. Lab tests may be drawn within the 3 days prior to vaccination.

[e] Reactogenicity assessments performed daily for up to 3 days post-vaccination (see Section 14.1).

Appendix G: Sample Addendum to Informed Consent Form for PART A

Title: A Phase I clinical trial to evaluate the safety and immunogenicity of HIV-1 *gag* DNA vaccine alone or with *IL-15* DNA, boosted with HIV-1 *gag* DNA + *IL-15* DNA, or HIV-1 *gag* DNA + *IL-12* DNA, in healthy, HIV-1 uninfected adult participants

Short title: A safety study of HIV-1 *gag* DNA vaccine and *IL-15* DNA adjuvant with 2 different booster injections

1. Review of Study Information

You are a participant in a study called HVTN 063. This study has 2 parts (A and B). You are participating in Part A, which is testing the safety of an experimental vaccine against HIV, the virus that causes AIDS. Part A of this study is also testing an experimental adjuvant. An adjuvant is a substance that helps the body respond to a vaccine. Due to some changes in the study design, we have additional information to share with you.

Please review this form carefully. The study staff will talk with you about the information in it. You are free to ask questions about this study at any time. You will be asked to sign this form to show that you have been given this information. You will get a copy to keep.

To review, the vaccine is called HIV-1 *gag* DNA vaccine, which will be called gag DNA vaccine from now on. The adjuvant is called *IL-15* DNA. These study products have been developed by Wyeth Vaccines Research.

2. New study plans

The study design for Part B is being changed so that the study will have 36 fewer participants. As a result of this change, 48 people will take part in Part A and 72 people will take part in Part B, for a total of 120 participants. Part B has also been changed so that the study as a whole will test only one HIV vaccine and two adjuvants. This change does not have anything to do with the safety of the study vaccine.

Your schedule of study visits will not change. You will be told whether you received vaccine or placebo after all of the participants in the study have completed their study visits. Not all participants join this study at the same time. Since you are in Part A, you may have to wait as much as two year**s** after your final clinic visit to learn what you got. This is longer than you were originally told.

Due to changes in the study, we will now take less blood for lab tests at some study visits.

The amount of blood taken at each visit will depend on the lab tests we need to do. It will be some amount between 15 mL and 300 mL (1 tablespoon to about 1.25 cups). The total amount of blood taken from you during this study will be no more than 1200 mL (about 5 cups). To compare, people who donate blood can give about 500 mL (about 2 cups) every 8 weeks.

3. Study progress and safety Information

As of March 2006, 36 participants have been enrolled in Part A of the study to receive the HIV-1 gag DNA vaccine, or placebo vaccines only. Twenty-four of these participants have received all 3 vaccinations.

The HIV-1 *gag* DNA vaccine and the *IL-15* DNA adjuvant have been safe and well tolerated. No severe reactions or illnesses related to the vaccines have been reported.

If you have questions about the changes in the study or your participation in the study, please ask the study staff. They can answer your questions, and can provide you with another copy of the consent form which you signed earlier if you need one. All the information in your original consent form is still in effect.

We thank you for your involvement in this trial.

4. Signature

If you have read this addendum to the consent form (or had it explained to you), understand it, and agree to remain in this study, please sign your name below.

Participant's name (print)	Participant's signature and date	Time (if signed on date of enrollment)
Study staff conducting consent discussion (print)	Study staff signature and date	Time (if signed on date of enrollment)

Site: For participants unable to read or write, substitute the signature block below:

Participant's name (print)	Participant's mark	Date	Time (if signed on date of enrollment)
Study staff conducting consent discussion (print)	Study staff signature and date		Time (if signed on date of enrollment)
Witness's name (print)	Witness's signature and date		Time (if signed on date of enrollment)
